# Supplementary material for: Understanding the implementation, impact and sustainable use of an electronic pharmacy referral service at hospital discharge: A qualitative evaluation from a sociotechnical perspective
Source: PLoS One. 2021 Dec 22;16(12):e0261153. doi: 10.1371/journal.pone.0261153 (PMC8694480; doi:10.1371/journal.pone.0261153)
Supplement: S1 Checklist — (DOCX) [file pone.0261153.s001.docx]

**Understanding the implementation, impact and sustainable use of an electronic pharmacy referral service at hospital discharge: A qualitative evaluation using Strong Structuration Theory**

**COREQ checklist**

Note: in order to minimize the length of the manuscript, some of the details on the checklist (marked ‘*’) are not included in the manuscript.

| *Guide question* | | *Response* | *Page number in main manuscript* |
| --- | --- | --- | --- |
| 1 | Interviewer/facilitator | MJ conducted the interviews. | P11 |
| 2 | Credentials | MJ holds an PhD in Pharmacy Practice | * |
| 3 | Occupation | MJ : Research Associate in medication safety; | * |
| 4 | Gender | MJ male | * |
| 5 | Experience and training | MJ has previous experience of undertaking qualitative research in healthcare at PhD and postdoctoral level | P10 |
| 6 | Relationship established | The researchers were not known to the participants prior to the study | * |
| 7 | Participant knowledge of the researcher | Participants were made aware of the reasons for doing the research via the information which was given to the participant prior to the interview | Sampling and recruitment P9; Ethics statement P11 |
| 8 | Researcher characteristics | The researchers had identified the study topic as part of larger programmes of work in their research groups, medication safety in primary care. | * |
| 9 | Methodological orientation and theory | The analysis was thematic and informed by sociotechnical theory | P6, P11-12 |
| 10 | Sampling | Sampling and recruitment.  Participants were opportunistically sampled from those health professionals working in secondary and primary care (community pharmacists, hospital pharmacists, hospital pharmacy technicians, general practice-based pharmacists and neighbourhood leads) who were actively involved in the implementation or use of the pharmacy e-referral TCAM system. The aim here was to find a range of different professionals to explore a range of views and perspectives of the service. Additionally, patients and carers for whom referrals had been made during their hospital admission were opportunistically sampled. The health professionals were approached through a range of professional pharmacy networks known to the researchers through the National Institute for Health research (NIHR) Greater Manchester Patient Safety Translational Research Centre (GM PSTRC) Community Pharmacy Patient Safety Collaborative and Salford Royal NHS Foundation Trust (SRFT). Participants returned consent-to-contact forms if they wished to take part. They were then approached by telephone, email, letter or face-to-face by a member of the research team (HB or MJ). Three health professionals declined to take part or did not return consent to contact forms. Those who declined cited workload and time commitments as the reason for their non-participation.  Patients or carers were identified through hospital pharmacists involved in the patients’ care and approached whilst in the hospital. Potential patient participants were provided with written information about the study alongside being provided information about their TCAM referral. Patients were not approached if they were confused, unconscious particularly unwell or frail and therefore in the opinion of the pharmacist unable to give informed consent. If during the approach by the hospital pharmacist, the patient indicated they would prefer their carer to take part they would then be asked to forward the information to their carer. Carers who agreed to take part returned consent-to-contact forms to the research team. All potential participants were given at least 24 hours to consider study information. Each participant in the study was assigned an identification number. This number, rather than the participant's personal details, was used to identify any interview data associated with the participant. | P9-10 |
| 11 | Method of approach | Participants were approached by directly face to face, telephone or email | P9-10 |
| 12 | Sample size | 27 participants | Results section P12.Table 1 |
| 13 | Non-participation | A number of possible participants were approached but declined to participate. For health professionals this was predominantly this was for reasons of time, workload or lack of use of the system. A number of patients approached could not take part due to ill-health or frailty | Discussion P33 |
| 14 | Setting of data collection | Interviews were conducted by MJ at the local NHS hospital trust or pharmacy where the participant was working, on university premises, or in the case of patients and carers at the participant’s home. | P10-11 |
| 15 | Presence of non-participants | No non-participants were present | * |
| 16 | Description of sample | Twenty-three interviews were conducted with health care professionals (n=18 participants, 18 interviews) and patients and carers (n=9 participants, 5 interviews). Two interviews with patients were conducted one-to-one with the remaining three as group interviews (patient/carer; carer/carer; patient/carer/carer). | Methods P10-11, Results P12 |
| 17 | Interview guide | The interviews were designed to elicit views and perceptions of using the electronic TCAM system. This included the benefits and drawbacks of the service; how organisational norms, work practices, workflow and conventions interacted and impacted upon the ongoing use of the service; and the communication and collaborations between health professionals. This would help us to understand the factors that might influence the acceptability, implementation, sustainable use and potential impact of the service. | P10-11, supplementary file |
| 18 | Repeat interviews | No repeat interviews were conducted | * |
| 19 | Audio/visual recording | Audio recording only, with consent from the participant | * |
| 20 | Field notes | None | * |
| 21 | Duration | The interviews lasted between 22 and 59 mins. | P10 and table 1 |
| 22 | Data saturation | Data collection continued until saturation was reached and no new themes emerged from the interviews and focus groups. | * |
| 23 | Transcripts returned | No transcripts were returned to participants | * |
| 24 | Number of data coders | MJ coded the data but regular discussions codes were held with co-authors | P11 |
| 25 | Description of the coding tree | A coding tree description is not given. Coding themes were developed from the first transcripts and then developed through discussions amongst the authors | P11-12 |
| 26 | Derivation of themes | Emerging thematic codes were applied to the data and new themes emerged from the data. This is described in the analysis section | P10 |
| 27 | Software | QSRNvivo 12 software was utilised to manage the data | P11 |
| 28 | Participant checking | No | * |
| 29 | Quotations presented | Please see the results section of the manuscript | P12-P28 |
| 30 | Data and findings consistent |  | P12-P28 |
| 31 | Clarity of major themes |  | P12-P28 |
| 32 | Clarity of minor themes |  | P12-P28 |
