# Supplementary material for: Understanding the implementation, impact and sustainable use of an electronic pharmacy referral service at hospital discharge: A qualitative evaluation from a sociotechnical perspective
Source: PLoS One. 2021 Dec 22;16(12):e0261153. doi: 10.1371/journal.pone.0261153 (PMC8694480; doi:10.1371/journal.pone.0261153)
Supplement: S1 Appendix — (DOCX) [file pone.0261153.s002.docx]

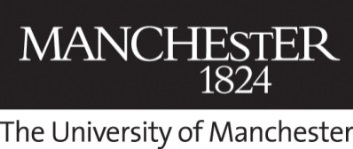


**Understanding the implementation, use and sustainability of an electronic pharmacy referral service at hospital discharge**

**INTERVIEW SCHEDULES**

**Part A - Health Professionals only**

**Interviewer introduction:**

I am [….] and I am a researcher at The University of Manchester. We are carrying out a project looking at how the pharmacy electronic referral service in Salford is introduced and used over time in hospital and in community pharmacy settings. We are particularly interested in the role of this referral service on improving the transfer of patients from secondary to primary care and whether the service has an impact on medication safety. We would like to find out more about the experiences of secondary and primary care staff, managers and other health professionals who have worked with the electronic referral service in some way.

This interview will last for up to one hour. During our time together I’d like to discuss your experiences of the pharmacy referral service in practice, with a focus on medication safety practices. I should remind you that the interviews are confidential. I’d like to audio record the discussion if that is okay with you; this is simply to help me capture all of the information that comes out of it. If you prefer, I can make written notes instead. You can ask for the audio-recorder to be switched off at any point during the interview. The recordings will be destroyed as soon as they are transcribed, the transcripts will be kept in a secure location for five years after the study is complete and then they will be destroyed.

Before we begin, I’d like to provide some ground rules for the discussion:

- You are being digitally recorded, so speak clearly;

- We will anonymise the transcript so that nobody can be identified by name. However, please try to avoid naming specific people or locations;

- Everything discussed here is confidential. However, if you were to reveal anything that would be considered unlawful or anything that would place you orsomebody else at risk of harm, we may have to report this to a clinical supervisor or manager. Unless you have any questions for me, then we can begin.

**Questions chosen will depend upon the role of the participant and their experience of using the referral service. Questions are designed to explore the contextual background, the socio-technical network involved in the service, the dispositions and actions of those involved and potential or actual outcomes and consequences.**

1. *Why do you think the pharmacy referral service was introduced?*
   1. What is it there for? What is the purpose of the service? What is it trying to achieve?
   2. What was there before? How was discharge information, particularly concerning patient medicines and for those on multi-dosage systems aids, communicated before? How good was the previous service?
   3. What resources were allocated? Was there training/support for the service– who, what, when?
2. *Can you describe how the pharmacy referral service was introduced into your workplace?*
   1. What went well when the service was introduced? What could be done better?
   2. Who took a lead on this? Who drove things forward and how, did they involve others?
   3. Did you contribute to the introduction of the service? In what ways? If not -Why not? Would you have liked to?
   4. Do some people know more about the service than others? What is the impact of this?
3. *Who uses the pharmacy referral service?*
   1. What do you know about the different people who use the service?
   2. Who are the important players? Why are they important?
   3. Who should, in your opinion, be using it? Why do you think that?
   4. Are some users different? Do your colleagues share your views?
   5. What do you think are the different implications of the use of the service for staff versus patients?

1. *Please describe how the referral service is used by you as part of your work.*
   1. What are your duties/roles? What do you do? Please describe exactly how you use the service.
   2. Initial views/expectations versus reality of use
   3. How have you contributed to the service?
   4. How often are you using it (sending or receiving referrals, counselling patients, involved with patients who have been part of the service etc)?
2. *Can you tell me about how the referral service ‘fits in’ with your existing work?*
   1. Has the service changed the way you or the team works? How has it impacted upon your work?
   2. Has it helped, hindered or constrained your work? What challenges have there been?
   3. Have you or others adapted your work to suit the service or vice versa?
   4. Has the service become an integral part of daily work? How was this achieved
   5. Do you think that using the tool should be part of your role/duties and why?
   6. Do you think that the service should be used routinely in discharge? Should it be used just for MDS or for discharges more generally? Why do you think that?
3. *What has happened as a consequence of the introduction of the referral service?*
   1. Do you think that the service has actual or potential value / benefits for your work?
   2. Is the discharge information provided through the service timely, relevant and useful?
   3. What impact has the service had upon your community pharmacy in terms of medicine supply? (e.g. things like waste, and time saved making up blister packs/MDS)
   4. Are there any problems with how well the service works and how are these dealt with, e.g. technical issues, interface issues
   5. Has the service disrupted working relationships?
   6. Is any information collected to find out how well the service is working for you and the team? Who is responsible and how is this information used
   7. Do you have any ways in which the team get together to talk about the service and how well it is working for you? Who is responsible for driving/organising this and what have been the outputs of such activity
   8. What changes (if any) would you make?
   9. Have the potential benefits of the service been realised? Do you think that the service can/will/will not/has already improve care?

**Interviewer conclusion:**

Concluding questions

*- Is there anything that you would like to talk about?*

*- Is there anything that you would like to go back and talk about?*

Switch off tape recorder

Many thanks for taking the time to help us with this study. Your contribution has been extremely valuable. If you wish we can send you a copy of your interview transcript, and you can also request to receive a summary of the findings of this research study, just ask us. In the meantime please feel free to contact either myself or the other researcher(s) involved if you have questions in future.

**Part B – Patients only**

**Interviewer introduction:**

I am [….] and I am a researcher at The University of Manchester. We are carrying out a project looking at how a new service designed to allow pharmacy staff in Salford hospital to electronically send information about patients’ medicines to their pharmacist when the patient leaves hospital. We want to know how the referral service is introduced and used over time by people involved with it, including patients and their carers. We are particularly interested in the role of this referral service in keeping patients safe as they move from hospital to the community and how the service might help the health care system to use medicines more safely. We would like to find out more about the experiences of patients and carers who have recently been discharged from hospital and who have received some kind of follow-up from their community pharmacist as a result of the new pharmacy referral service or from the hospital pharmacist in preparation for their leaving hospital.

This interview will last for about 30 mins (and no longer than one hour). During our time together I’d like to discuss your experiences of the pharmacy referral service, about your medicines and medicine taking and about any conversations you may have had about your medicines either in hospital or during a visit to the community pharmacist. I should remind you that others will not be able to identify you in any reports which contain quotes from your interview. I’d like to audio record the discussion if that is okay with you; this is simply to help me capture all of the information that comes out of it. If you prefer, I can make written notes instead. You can ask for the audio-recorder to be switched off at any point during the interview. The recordings will be destroyed as soon as they are typed up, the typed interview records will be kept in a secure location for five years after the study is complete and then they will be destroyed.

Before we begin, I’d like to provide some ground rules for the discussion:

- You are being digitally recorded, so speak clearly;

- We will make sure the typed up record of the interview has any identifiable information removed, so nobody or nowhere can be identified by name. However, please try to avoid naming specific people or locations;

- Any discussions that take place during the study are confidential. However, if you were to tell us something new that could put you or someone else at risk of harm we will ask you to contact an appropriate person about this, such as your GP, care team, other health professional or a family member or, if we consider it to be serious, we may have to report this. In no other circumstances will we discuss with your GP, care team, other health professional what we have talked about in the interview or contact them on your behalf.

Unless you have any questions for me, then we can begin

1. *Firstly I’d like to talk about your medicine taking. Could you please tell me about the medicines that you take?*
   1. What do you take them for? How long have they been prescribed? Who prescribed them?
   2. Thinking about medicines you were taking **before** you went into hospital recently. What information were you given about those medicines when they were prescribed to you? Did you talk to anyone else about those medicines apart from the doctor who prescribed them?
   3. Thinking about medicines you were taking **before** you went into hospital recently again. Did you have any problems with those medicines? For instance did you always get your medicines on time? Did any problems get resolved? Who helped you with those problems?
2. *Thinking about your recent visit to hospital. Did anyone talk to you about your medicines in hospital? Who talked to you? What was that conversation like?*
   1. Were any of your medicines changed? Who made those changes? Do you know why they were changed?
   2. What information were you given in hospital about your medicines? Were any changes to your medicines explained to you? Who explained that?
   3. How did you feel about the ways those explanations were given? Do you think you got enough information about your medicines?
   4. Since leaving hospital have there been any problems getting your medicines? If so how were these resolved? Who helped with this?
3. *Thinking about your recent visit to hospital. Can you tell me how/when you were told about the pharmacy referral service?*
   1. What were you told about the referral to the community pharmacy service? How was it explained to you? What were you told? Who explained it? Were you happy with that explanation?
   2. What reasons were given to you for the referral? Why do you think you were referred? What do you think is the purpose of the referral to the community pharmacy?
   3. How did you feel about the way the pharmacy referral service was explained to you?
   4. What do you think your role is in the service? How do you think the referral service could be helped by patients?
4. *Have you spoken to a community pharmacist or a pharmacist at your GP practice since leaving hospital?*
   1. If so what happened? How did the meeting go? If not would you have liked to have talked to someone? Why? How do you think that would have helped?
   2. What did you expect was going to happen? Was the meeting similar to your what you expected or different? In what ways?
   3. Why do you think the community pharmacist wanted to see you? Was this explained by them? How did they explain that? Were you happy with that explanation?
   4. What sorts of things were discussed with the community pharmacist? What did you talk about?
   5. Did you have an opportunity to ask questions? What questions did you ask? How where these answered? Were your concerns addressed in the way you wanted?
   6. Do you think visiting the pharmacist has helped you understand your medicines? In what ways? Could/did the service have addressed your concerns with medicines information needs, changes to medicines etc? (link back to any comments they made earlier)
   7. Do you think visiting the pharmacist has helped with your medicine taking? In what ways?

**Interviewer conclusion:**

Concluding questions

*- Is there anything that you would like to talk about?*

*- Is there anything that you would like to go back and talk about?*

Switch off tape recorder

Many thanks for taking the time to help us with this study. Your contribution has been extremely valuable. If you wish we can send you a copy of your interview transcript, and you can also request to receive a summary of the findings of this research study, just ask us. In the meantime please feel free to contact either myself or the other researcher(s) involved if you have questions in future.

**Part C –Carers only**

**Interviewer introduction:**

I am [….] and I am a researcher at The University of Manchester. We are carrying out a project looking at how a new service designed to allow pharmacy staff in Salford hospital to electronically send information about patients’ medicines to their pharmacist when the patient leaves hospital. We want to know how the referral service is introduced and used over time by people involved with it, including patients and their carers. We are particularly interested in the role of this referral service in keeping patients safe as they move from hospital to the community and how the service might help the health care system to use medicines more safely. We would like to find out more about the experiences of patients and carers who have recently been discharged from hospital and who have received some kind of follow-up from their community pharmacist as a result of the new pharmacy referral service.

This interview will last for about 30 mins (and no longer than one hour). During our time together I’d like to discuss your experiences of the pharmacy referral service, about the medicines and medicine taking of the person you care for and about any conversations you may have had about their medicines either in hospital or during a visit to the community pharmacist. I should remind you that others will not be able to identify you in any reports which contain quotes from your interview. I’d like to audio record the discussion if that is okay with you; this is simply to help me capture all of the information that comes out of it. If you prefer, I can make written notes instead. You can ask for the audio-recorder to be switched off at any point during the interview. The recordings will be destroyed as soon as they are typed up, the typed interview records will be kept in a secure location for five years after the study is complete and then they will be destroyed.

Before we begin, I’d like to provide some ground rules for the discussion:

- You are being digitally recorded, so speak clearly;

- We will make sure the typed up record of the interview has any identifiable information removed, so nobody or nowhere can be identified by name. However, please try to avoid naming specific people or locations;

- Any discussions that take place during the study are confidential. However, if you were to tell us something new that could put you or someone else at risk of harm we will ask you to contact an appropriate person about this, such as your GP, care team, other health professional or a family member or, if we consider it to be serious, we may have to report this. In no other circumstances will we discuss with a GP, care team, other health professional what we have talked about in the interview or contact them on your behalf.

Unless you have any questions for me, then we can begin

1. *Firstly I’d like to talk about the person you care for and their medicine taking.*
   1. In what ways do you help the person you care for with their medicine taking/ Do you collect their medicines for them? Do you talk to them about their medicines? Do you help them take their medicines? In what other ways do you help?
   2. Thinking about medicines they were taking **before** they went into hospital recently. What information were they/you given about those medicines when they were prescribed? Did you talk to anyone else about those medicines apart from the doctor who prescribed them?
   3. Thinking about medicines they were taking **before** they went into hospital recently again. Were there any problems with those medicines? For instance did they always get their medicines on time? Did any problems get resolved? Who helped you with those problems?
2. *Thinking about the person you care for’s recent visit to hospital. Did anyone talk to them/you about their medicines in hospital? Who talked discussed those? What was that conversation like?*
   1. Where any of the medicines changed? Who made those changes? Do you know why they were changed?
   2. What information were you given in hospital about the medicines? What information was given to the person you care for? Were any changes to your medicines explained? Who explained that?
   3. How did you feel about the ways those explanations were given? Do you think you got enough information about the medicines?
   4. Since leaving hospital have there been any problems getting your medicines? If so how were these resolved? Who helped with this?
3. *Thinking about the person you care for’s recent visit to hospital. Can you tell me how you were told about the e-referral to the pharmacist service?*
   1. What were you told about the referral to the community pharmacy service? How was it explained to you? What were you told? Who explained it? Were you happy with that explanation?
   2. What reasons were given to you for the referral? Why do you think you were referred? What do you think is the purpose of the referral to the pharmacist?
   3. How did you feel about the way the pharmacy referral service was explained to you?
4. *Have you or the person you care for spoken to a community pharmacist or a pharmacist at your GP practice since leaving hospital?*
   1. If so what happened? How did the meeting go? If not would you have liked to have talked to someone? Why? How do you think that would have helped?
   2. What did you expect was going to happen? Was the meeting similar to your what you expected or different? In what ways?
   3. Why do you think the community pharmacist wanted to see you? Was this explained by them? How did they explain that? Were you happy with that explanation?
   4. What sorts of things were discussed with the community pharmacist? What did you talk about?
   5. Did you have an opportunity to ask questions? What questions did you ask? How where these answered? Were your concerns addressed in the way you wanted?
   6. Do you think visiting the pharmacist has helped you understand the medicines for the person you care for ? In what ways? Could/did the service have addressed your concerns with medicines information needs, changes to medicines etc? (link back to any comments they made earlier)
   7. Do you think visiting the pharmacist has helped with medicine taking or the person you care for? In what ways?

**Interviewer conclusion:**

Concluding questions

*- Is there anything that you would like to talk about?*

*- Is there anything that you would like to go back and talk about?*

Switch off tape recorder

Many thanks for taking the time to help us with this study. Your contribution has been extremely valuable. If you wish we can send you a copy of your interview transcript, and you can also request to receive a summary of the findings of this research study, just ask us. In the meantime please feel free to contact either myself or the other researcher(s) involved if you have questions in future.
