# Supplementary material for: Understanding the implementation, impact and sustainable use of an electronic pharmacy referral service at hospital discharge: A qualitative evaluation from a sociotechnical perspective
Source: PLoS One. 2021 Dec 22;16(12):e0261153. doi: 10.1371/journal.pone.0261153 (PMC8694480; doi:10.1371/journal.pone.0261153)
Supplement: S2 Appendix — (DOCX) [file pone.0261153.s003.docx]

**TCAM - First coding - Coding Framework - Sets of codes**

| **Set A. Communication between Health Professionals** | **Set B. Communication with patient** | **Set C. Dispensing, supply, availability and taking of medicines** | **Set D. Implementation and the processes of the system** |
| --- | --- | --- | --- |
| Communication between Health Professionals | Communication with patient | Dispensing, supply, availability and taking of medicines | Implementation and the processes of the system |
| Communication CP and Hosp | Changes in communication with patients | Changes to meds not reflected in supply | Getting used to the system; still learning |
| CP_GP communication | Communicating changes to medicines | Cost and Wastage | Implementation and the processes of the system\Implementation |
| Future - Possible referrals between GP-CP | Delivery of meds adds a barrier to communication | Delays in supply | Implementation -adaptations and changes and adapting to change |
| Informal communication | Formal communication - NMS or MUR | Errors in medicines dispensed | IT infrastructure |
| Information exchange | Informal - ad hoc | Medicines have been changed | Policy drivers |
| Initiation of contact between GP and CP |  | Patient or carer active role in meds | Resources for implementation and sustainability |
| Potential and benefit of three-way communication |  | Patient waiting for medicines | Training |
| Standardising and streamlining communication |  | Pharmacy supplying meds effectively | **Sub - Set D. ii** **Implementation and the processes of the system - Involvement of NIPPS and GP Pharmacists** |
| Systems for communication |  | Problems receiving discharge information and supplying medicines | Involvement of NIPPS and GP Pharmacists |
|  |  |  | Delays in NIPPS getting information |
|  |  |  | Having systems for practice based pharmacists |
|  |  |  | IT difficulties for NIPPS |
|  |  |  | NIPPs team not involved |
|  |  |  | Time saving if NIPPS were involved |
|  |  |  | Workload for NIPPS |

| **Set E. Knowledge and Information** | **Set F. Potential benefits and Capacity to initiate change and actions** | **Set G. Relationships** | **Set H. Responsibilities, goals and values** |
| --- | --- | --- | --- |
| Knowledge and Information | Potential benefits and Capacity to initiate change and actions | Relationships | Responsibilities, goals and values |
| Accuracy and timeliness of information being received at CP | Actions of Practice pharmacists | Building relationships as a consequence of the system | Patient or carer responsibility |
| ‘because a lot of the time if you don’t ask, you don’t get told.’ | Communication of changes in meds will help patients | Joined up communication | Patient wishes, goals and values |
| Carer or patient knowledge about meds | Future benefits |  | Peace of Mind |
| Distinction between Explanation, Information Communication and Knowledge | Patient not in receipt of incorrect medication |  | Responsibility for correct meds |
| Knowledge and information leads to safety | Patients feel that community pharmacy is more involved in their care |  | Shared responsibility |
| More detailed information in discharge summary for CPs | Patients who may benefit - patient characteristics |  | Transfer of responsibility |
| Patient or carer has information about meds | Value of community pharmacy |  |  |
| Patient trust in medicines |  |  |  |
| Power and control of information |  |  |  |
| Shared information and knowledge |  |  |  |
| Where does patient get information - How do they access information |  |  |  |

| **Set I. Structures** | **Set J. Systems** | **Set K. Transition from secondary to Primary** | **Set L. Workload and Workflow** |
| --- | --- | --- | --- |
| Structures | Systems | Transition from secondary to Primary | Workload and Workflow |
| Community pharmacy structures | Checklist and structure | Different actors in discharge process | Before and after - change |
| GP Structures | New system works smoothly | Discharge is a complex process | GPs are too busy |
| Hospital structures | People and roles involved in discharge process | Discharge process is not just e-referral its about accuracy of prescription and summary | Time saving |
|  | Problems with new system | Knowledge transfer during transition | Work processes |
|  | Processes on admission | Problems with transition |  |
|  | Several steps in previous way of working | Processes of discharge |  |
|  | Timing of receiving alerts |  |  |
