# Supplementary material for: Understanding the implementation, impact and sustainable use of an electronic pharmacy referral service at hospital discharge: A qualitative evaluation from a sociotechnical perspective
Source: PLoS One. 2021 Dec 22;16(12):e0261153. doi: 10.1371/journal.pone.0261153 (PMC8694480; doi:10.1371/journal.pone.0261153)
Supplement: S3 Appendix — (DOCX) [file pone.0261153.s004.docx]

**TCAM – Final coding framework**

| **Theme** | **Codes** |  |  |  |  |
| --- | --- | --- | --- | --- | --- |
| **1. Knowledge and Information - Quality of Information** | Power and control of information | Quality, detail, accuracy, and timeliness of information |  |  |  |
| **2. Availability and accessibility of information** | Accessibility availability and the sharing of knowledge and information. | Patient knowledge of e-referral service, discharge, and medicines |  |  |  |
| **3a. Adaptation of work processes to the availability of information** | Implementation process - Getting used to the system, learning, training, adapting and changing | Previous system and work processes |  |  |  |
| **3b. Adaptation of communication across the network** | Communication initiated by patient | How communication between health professionals works or might work. | Interrelationship between communication and relationships and the expectations around what that achieves or might achieve | Limitations in communication - barriers, limits, communication that doesn't happen, no MUR or NMS, information that doesn't get sent | Ways in which Health professionals currently or could communicate with patients, either because of service or other wise |
| **3c. Infrastructures - existing, adapted, and new.** | Changes in technology and technologies | Information Technology - benefits of, availability, access, use. | MDS - The processes and technology involved |  |  |
| **4a. Changes in meds safety work** | Admin time down - Clinical time up | Discharge process, structures, systems and new ways of working. Benefits of, or problems, with the new system | Medication safety - medicine related admissions, errors and delays in dispensing and supply of medicines - cost and wastage. | Timesaving, speed, efficiency - streamlining of communication | Which patients should it or could it be used for - MDS patients or other |
| **4b. Nature of the Network - agents and how it operates** | Completeness, fragmentation or incompleteness of the network | Different actors in the network and how workflow might be different for each of them. | Movement of information in the network - How is information exchanged between actors | Patient preference in relationships with health professionals | Relationships between different health professionals and what this achieves |
| **5. Perceived impact and potential benefits - Hoped for, anticipated realised and unintended changes** | Aspirations and expectations | Good communication leads to timeliness and promotes good relationships | Potential benefits - Capacity to change, capacity to improve care | Value, trust and confidence in health professionals | What changes should be made - How can the service be improved. |
