# Supplementary material for: Understanding the implementation, impact and sustainable use of an electronic pharmacy referral service at hospital discharge: A qualitative evaluation from a sociotechnical perspective
Source: PLoS One. 2021 Dec 22;16(12):e0261153. doi: 10.1371/journal.pone.0261153 (PMC8694480; doi:10.1371/journal.pone.0261153)
Supplement: S4 Appendix — (PDF) [file pone.0261153.s005.pdf]

ALL EXTRACTS FROM CODING OF TRANSCRIPTS

Theme 1. Knowledge and Information - Quality of Information

1.1 Code - Power and control of information

HP16

R: And I think also there's a lot of work that goes into preparing for patients who've been discharged, it's really not sung about or not recognised. So just a meds rec in itself is a routine thing that we do, but actually what about the discharge MUR, what about the advice on new medicines, they're looking at actually are they still compliant, do they have any issues with the medicines, is there anything we need to do that's different? So for us that's kind of a very, very big thing and also I think usually patients think of the GP in charge of discharge but actually for your blister pack patients we're probably a lot more in control of the medicines, the information, making sure things are done properly because from our end we need to know that's there to restart the MDS, for example. Or to make sure that the prescription's ordered on time for the next one, anything else that's needed that the patient has. And the other part I think people forget to recognise is that the GP deals with the patient, we deal with the family and the carers as well. And you can have family members coming in, carers coming in, all sorts of other things going, where's this, where's that, where's this, where's that? And you could get to a point on a Friday, for example, where this patient's been in hospital, we never knew about it, they need a blister pack now and what's happened? Well, we haven't had the information.

HP17

But I mean you've got summary care records, which is better now, but from past experience this new system of getting electronic referral is brilliant because you know instantly…you know when they go in hospital, you get an email for that and you can bring it up on PharmOutcomes, and you know when they come out, and you get…all the information that the doctors get you get, I'm assuming the same, or very similar, information. So, I've had problems in the past with one particular surgery, not mentioning any names, that wouldn't even give me a discharge sheet from the doctors, they refused, the practice manager refused to give me a discharge sheet, and this is what she said to me, not mentioning any names, was, 'well the doctor's done the prescription, it'll be right because the doctor's done it, and we're not releasing a discharge sheet.' But what happens if it's not right? What happens if the doctor's made a mistake? What happens if it's been transcribed wrong? It's my job as a pharmacist to check what they've come out of hospital matches what the GP's prescribed and I can't do that if I've not got a discharge sheet.

HP2

I: What happens once the patient has left is going to maybe have an impact or whether they come back at all.

R: If, you know…once that patient goes home, if somebody then can have contact with that patient, maybe in their own home, or the patient goes into the GP surgery and speaks to pharmacists or technicians in there, just to keep up-to-date with that patient to say, are you fine with your blister pack? How can we help you? Are you finding any problems? That kind of thing could work in the community and I don't know if people are doing that kind of thing now. So, I don't know how it can help…like the hospital pharmacy, once we've done that referral and they've left, I don't know what benefit we can…

I: When you do the referral, do ever put an action on for the…right.

R: No. I don't.

I: So, you only tell them what's happened.

R: Yeah. We let them know that the patient's in or they've gone, and then they'll receive the discharge summary, which has…the doctor will write why the patient has been in, what actions they've taken, medication they came in on, if anything was added, if anything was stopped. Yeah. So, that's it then, we've…it's finished from the pharmacy department then […] Then it is up to the community […] to deal with medications, but then maybe social issues, that brought the patient in, whether it is medication-wise or not, I don't know. If it could help, it could benefit somebody in that way. Another team.

HP5

R: Sometimes they can go to a facility where they are dealt with by…where the facility deals with another chemist, and therefore I have to be aware that I'm not sending information to the wrong source. To another, to someone who doesn't need it, you know, it's a breach of data protection again, so I have to make sure I'm in line with that myself.

I: Yes, precisely. So, thinking of those things and keeping that confidentiality there as well, so…and did you say some of your patients would then, would they go in to nursing homes or care homes or…

R: Yes, they can go to all the care facilities and that, those care facilities they are just another pharmacy that deals with them. And again, we've got the permission from the patients that if they don't mind the pharmacy being informed of their medications.

HP5

I: When…before it happened, what were your sort of expectations of what, what the service might look like, compared to what, you know?

R: I did, I must admit I thought the community pharmacy referral would just isolate the medications that the patient is on and send that to the pharmacy. But my understanding is the discharge summary actually goes along with them, the list of jobs to the chemist. That aspect is important in letting the community…letting the patient know that the community pharmacy is receiving all this information.

I: Right, why is that important?

R: Because they may have information…people's imagination of a community pharmacist that all they should have is drug information, but they don't see them as having any other... I've worked in HIV and infectious diseases before, there is certain information that hospital

will have that perhaps you might not want a community pharmacy to have which regards the status of patients, are sort of like treated the same way. There has got to be other information that perhaps people may feel the community pharmacy doesn't need to know. It might be inclusive on there, ie if they are an alcoholic or if they abuse drugs or if they whatever, community pharmacy doesn't necessarily know the background to that patient or certain aspects of a patient. So, they need to know that everything is being sent via the community, e-referral to the patient.

P2

I: That's interesting because…I mean in what way would you like to be told about that? Would you…who do you…or firstly, how do you think it would help you to know what you're on? Do you think it would help you to know why you're on those medicines?

R: Well I'd like, yeah…I mean me, daughter's more taken control because I forget so much, and this is the problem and I do, you know, because she said to me write it down mum, but I find that at the hospital and the GP, they're just changing it, and of course they haven't got time to explain everything to you because you're only allowed so many minutes with your GP and at the hospital all they do, is just discuss it amongst themselves, and they don't discuss it with the individual.

P3

I: Who do you think is best at helping you with your medicine taking, who gives the best advice and best help around what you need to do with your medicines?

R: That's a difficult one, that, because a lot of the time if you don't ask, you don't get told, you know.

I: Yeah. So you feel a lot of that is down to you then therefore…?

R: Yeah, yeah, if I don't ask, I don't get to know, I don't know why, perhaps it's your old medical profession thing where a little information can be too much, you know, I don't know, I don't know because I love the NHS the way everything is going, the informality of it and the friendliness of it it's all first names, you know, I think it's brilliant and it puts people at their ease. But as I say, there may be that little secrecy over the meds and…

I: Yeah, and passing information to you, yeah.

R: Yeah, yeah, I know and I'm not telling you, you know, it keeps them in a little position of power if you like, yeah, control I should say.

1.2 Code - Quality, detail, accuracy, and timeliness of information

HP1

I: Yes, because there's a lag in the system isn't there, because they're getting the information, they may have already made changes, by the time you know, things could have already been resolved.

R: Have done, or will be getting done, yeah. 'Cause obviously there's a lag in us receiving the email, I mean I only work part time, I don't work on Mondays, so I would only look at this on Tuesday, and then I'd disseminate it to my pharmacy or myself. Now, is that particular pharmacist in that particular…is there a pharmacist in that practice on that particular day? So, sometimes there are delays. And that again, goes back to the importance of us having the system, 'cause if we have the system, we're looking at it all the time, and yeah.

HP1

I: Yeah. So, a lot of pharmacists would be in the position anyway, it wouldn't be an unfamiliar system to…presumably. So, if you had that three, if you were seeing as the discharge or e-referral went to the community pharmacist, if it came to the practice pharmacist at the same time, how do you think that would be of benefit then?

R: I mean, it depends on what needs changing. If it was something to change on a repeat, we'd just do it there and then. I think also, the issue is that the community pharmacists, there's been two occasions where they've said something, and actually it didn't need reporting, because it would have been removed anyway. So, if it was a, they had a repeat, and they said, it's still on the repeat, it was still on the repeat because that's an old prescription, an old repeat. So, sometimes I think that the selection of boxes, they also need to understand what that would look like for us, so I think the importance of the community pharmacy selecting the right boxes, and the hospital pharmacy selecting the right boxes, because if they select the wrong box, it just means something different to us. So, for example, I'll try to make sense of what I've just said, if it was about, like if we take this example, so, medicines stopped in hospital, still on repeat. This is what the pharmacy had told us. And again, it's difficult, 'cause I haven't seen the system, I don't even know how they do it. But, do they know that that is actually the warning for us, the issue that's been highlighted for us to action, or are they just ticking this, assuming that it's just information, just saying that the hospital's stopped this, and it's on repeat, but it's fine, it's nothing we want anyone to do anything about it, we're just stating a fact. But I don't think that they realise that that, to us, is, okay, it's stopped in hospital, but it's still on repeat, there's a problem, why is it still on repeat? But then going back to the community pharmacy, when I called them, they said, oh it's fine, it's just that it had been stopped, but I don't think they realised that that was something that's been highlighted.

HP10.

I: There's more quality information?

R: Yeah, yeah, where before it was handwritten very often and sent via the fax, it got even worse in terms of quality, you couldn't really read properly what that information was. I've got a feeling this digital we get now and it's even more...there's more in it, there's more information. I don't think it's the same as the doctors get, I think they get an even bigger one, but we, for example, now can find out about what the patient was admitted with. I always scan through it, I always find time to, and I read, oh, this and that and the other, I read the little history. I'm not an expert, I'm not a GP, I'm not a doctor, so I don't... I mean, I understand most of it but I don't understand everything, but it's helpful to know that, say... I'll

give an example. If someone was...it's happening quite often, I think 20, even 30, per cent of my patients will be admitted due to falls, they'll be falling, they fell in the house, they hurt themselves, taken to hospital. And I'm thinking, oh God, looking at what are they on. Oh, they take alendronic, they take this, this and this, and I'm thinking, Christ, how did I not pick this up earlier? Oh gosh, so many strong painkillers, bloody hell. Maybe we should be more conscious about it. You've got to get a better and bigger picture. You can understand this patient, oh, hold on a minute, oh Christ, I should maybe make sure that she... Doctors often don't think about those things, they will issue another painkiller that could make them drowsy, and I'm thinking, hold on a second, she was admitted to hospital with a fall not so long ago. Just think about that. Not so long ago, where a lady was on very strong pain relief and their doctor must have given...went for a home visit. So, I just picked it up thinking, oh, Christ, she's given her this. Oh, no, no, she shouldn't be on it, she's already on this, she had this fall. And I just rang: listen, this and this happened not so long ago. Oh, yeah, yeah, thanks (name) I will change it, so that was good.

HP11
I: Was there a difference between what information was then or…to what you get now?

R: I think we didn't always get the full…sometimes we would just get the medicines, whereas now, we get this patient was admitted on…and sometimes we did. I think more recently we did but in the past you'd just get the sheet with the medication on. Whereas now it says…

I: So these are the new medicines ….

R: Yeah, whereas now it says why they went in, where they've been discharged to, how long they were in hospital, what changes they've made and where they've gone home to. Where they've gone to, because they don't always go home. Sometimes they go […] into an intermediate care or…whereas we've got the full information, it's the full information that the GPs get. And then we can then…I think the only problem that we've found is that it's the pharmacist at the practice who actions that medication, that discharge and I don't think he…he's saying he doesn't always get it. He doesn't see that email. So whether it's just being sent to a general practice, an email for the practice and it's never getting forwarded to him…I mean, it doesn't really matter because we're sending it to him anyway. But he's not always seeing it before we're saying, (name), have you done this script?

HP12
I: Talking about the pharmacy referral service then, can we talk about when it was first introduced. Why do you think it was brought in, what's it there for?
R: I think to just improve communications between the two. That's what's worked best for us. The fact that we actually get told when one of our patients goes into hospital, is a big thing. Because quite often they'd ring and say I'm out of hospital, they've changed my medicines, we didn't even know you were in, we've still been sending you medication every week, where's that been going, you know. And to get the discharge information as well. 'Cause sometimes we would get that faxed through, sometimes we wouldn't. They went through a spell where they wouldn't fax it, trying to get it off the doctors sometimes is difficult. So, it just makes the whole process so much easier because we are getting the information quickly and the accurate information.

HP12
I: Yeah. What about the discharge summaries, how useful are they then.
R: Really useful because you've got all the information there about the medication. And it's very straightforward how it's presented and it's easy to compare that against our control charts that we use.
I: So, before you just got a list and now you get everything about that patient.
R: Yeah. So, obviously we get the list of medications which is easy to check against ours but there is also a section where it says, what's stopped, what's started, I don't know whether to say it's interesting or nosey to know why they were admitted, but sometimes it might be something that we can prevent happening in the future, so I don't know, if they have gone in with acute kidney injury because they've had sickness and diarrhoea and they've carried on with all the medications, or anything like that really; or they've taken too many of something and they've ended up in.

HP13.
I: Yeah, and how much information was in that fax?
R: Not as much as in this, no, it didn't tell you what date they went in to hospital which it does in this. Well, it did tell you the date of discharge, I suppose, but there just wasn't as much information, it was simply the medicines. And this tells you which…if they're going back to their normal home, it tells you now. It didn't tell you that, you didn't know that before 'cause that's relevant, they could be going off to a care home. And if that's the case, then it's a whole different ball game 'cause they're probably changing GP.

HP13.
R: Yeah, go through it. I like the PharmOutcomes thing because this actually makes you go through it in a systematic way. See, because when you've got the discharge now, you have to actually say what you've done and tick boxes, you see, which is marvellous because before, I did it but I wasn't acknowledging to myself or anyone else that I did it.You know and it's saying, the first question is have you reconciled all the medicines and that's the first thing you actually do which we always did but now it's making you do it, it's making you declare that you did it and it's asking you have you got a repeat prescription, which is good. You have to look and see have you and then they ask you is it correct. And then they ask you have you had to refer anything back to the GP which again, if I do, which I often do, then I have to obviously press a yes.
I: Is that all within PharmOutcomes?
R: Yeah, it's all in one page, it's excellent and there's notes to say…you can put what exactly you had to refer to the GP and why you had to and what the GPs done about it. This is obviously marvellous.

HP13.
I: Yeah, there might be something within the service that needs to be thought around, that, sort of, thing. I was thinking, there was a question about disrupting working relationships and actually that's something, you know, it's around use of community pharmacy and the rest of it. If you were told you were in, now, in charge of the whole thing as of tomorrow morning, what would you do, what would you change or would you change anything, what would you do to…for that service?

R: I think there was a little bit on the form that I thought could’ve been more clear because I didn’t know ’til the second meeting about. I mean, it’s there but I didnt’ see it and it’s quite an important little bit. And it was only at the second meeting that one of the ladies from Hope Hospital actually said, oh look, this is when they went in, this is where they’re going, which residence they’re going to and this is very important. I just think that could be highlighted more, maybe, in case other people haven’t noticed it ’cause I’m sure they haven’t.
I: Is knowing where they’re going really important?
R: Well, it is really because if they’re going to residential care, I’m not doing their prescription anymore and the GP needs to know that too because often their GP changes so they won’t be doing it either and something needs to be sorted out for them and that, kind of, thing. Or you might want to be saying to the GP, shall we do a couple of weeks ’til they get sorted or whatever, I don’t know.
I: Yeah, or if they’re going into intermediate care or whatever, yeah.
R: Yeah, just…there’s things like that. What else, really. No, there’s plenty of information there. There’s a little bit of quite interesting background where if they’ve got dementia or this, kind of, thing that you might not have known, we might not have known that.

HP13
R: Yeah, it would be important because you might explain things differently to somebody, knowing what condition that they had or that, kind of, thing, you know. Or even maybe what they were in for, I think that was always on it but it’s just a bit more detailed.You know, you might give them extra advice if you know that they’ve been in ’cause they’ve had pneumonia, you might tell them about having the pneumonia jab and all that when they’re better, there’s various little things like that, that’s good. But I don’t know what else could be improved, really. With this PharmAlarm, I don’t think…that’s just excellent, really.

HP15
I: Good. We are recording. We are talking about…thinking about the introduction of the pharmacy e-referral service. What do you think it is there for? What do you think the purpose of having that e-referral is?
R: So, it gives a clearer discharge letter to us first of all. So, the handwritten ones, sometimes they are not very clear and it causes confusion. But when it’s electronic, it’s easier for us to go through and compare to the old medication and see if there are any changes. So, it’s quicker for those purposes. Also, because it is electronic, we get to know quicker than we would usually if the patient has been admitted to the hospital or is discharged. Because sometimes we wouldn’t even know if they had been admitted, until they come out and they ask for their medicine. So, we wouldn’t know, yeah. But it’s better when it’s electronic now. So, we get to know that they are in the hospital now.

HP15
I: Yeah. So, you were getting informal conversations from people to let people know, but not before. And you said that the discharge was handwritten beforehand, and it wasn’t electronic. What information did you get from that before?
R: It’s just the medication. So, what they were discharged with. What medication they were discharged with. So, you usually used to get faxes. So, they call us first to say that, what’s your fax number? And they’ll fax over the discharge letter. But sometimes when they do that, we still don’t get the discharge letter until days after. And then the patient…at that point because it’s not straight, it’s not the same day they are discharged. It is probably two days after they’ve been discharged. So, there’s a delay.
I: Yeah, they are already in the community.
R: Yeah. And then what happens the patient will need the medication. They usually get discharged with seven day’s supply. So, if you have not received it by the fourth day…I mean when the doctors haven’t received the discharge at that point, there’s a big delay because they don’t know, they can’t do the prescriptions until they’ve seen the new discharge letter and everything.
I: And then you’ve only got three days left before.
R: Yeah. So, what happens we keep…we are continuously chasing up…can we get the prescription for…when they haven’t got the discharge either. So, we usually get the discharge on the fifth day. So, it just leaves two days for them to look at everything, arrange a prescription. And the patient is getting very worried and saying, well I don’t have medicine, I don’t have medicine. Even though they have two days left but they’re scared. So, they keep chasing us, that can I have the medicines?

HP16
R: The admission…I ...the admission and getting that notification.
R: It is and we never used to get that, well, I don't think never, sorry, but hardly, we used to hardly get notified that somebody was in because a lot of the time you have some revolving door patients who'll go in for the day, come out, no changes. But even that for us is useful to know because there's an impact on potentially, if there is any impact on the clinical side of it, so actually they may have gone in with an infection but actually they could have gone in with thinking it was an AKI. It may not have turned out but actually that's a prompt for us to say, here's a conversation for to be had with the patient.
I: Yes, precisely.
R: And I think also there's a lot of work that goes into preparing for patients who've been discharged, it's really not sung about or not recognised. So just a meds rec in itself is a routine thing that we do, but actually what about the discharge MUR, what about the advice on new medicines, they're looking at actually are they still compliant, do they have any issues with the medicines, is there anything we need to do that's different? So for us that's kind of a very, very big thing and also I think usually patients think of the GP in charge of discharge but actually for your blister pack patients we're probably a lot more in control of the medicines, the information, making sure things are done properly because from our end we need to know that's there to restart the MDS, for example. Or to make sure that the prescription's ordered on time for the next one, anything else that's needed that the patient has. And the other part I think people forget to recognise is that the GP deals with the patient, we deal with the family and the carers as well. And you can have family members coming in, carers coming in, all sorts of other things going, where's this, where's that, where's this, where's that? And you could get to a point on a Friday, for example, where this patient's been in hospital, we never knew about it, they need a blister pack now and what's happened? Well, we haven't had the information.

HP16
I: And you get more information on that discharge than you were getting?
R: Yeah, so it used to be we just used to get the medicines copy and, if we were lucky, we'd get the medical stuff, but now we're getting a copy of the full discharge and the medical stuff. And I think, give (name of hospital) credit, they have actually worked to make the discharge more pharmacy friendly as well, so actually encouraging more information to be put on.

HP16

I: So you now know why that patient's gone in, what's happened, what changes have happened, what's been resolved what [voices overlap 05:25].
R: Yeah, I mean it's down to the quality of the discharge, the discharges do vary in quality from being very, very detailed to being the bare basics, but we still have more information than we did to help that patient. And the key parts are it's usually medicines started and stopped because that stuff is really key for our role in that transfer of care. That's the whole point of it is actually has it been stopped, has it not, has it been missed? We don't know and the GP practice might spend a few days doing that but we don't know that they're spending those times chasing it up or getting the information, all we see is this is what's going on. And then the other part of it is it allows us to be a bit more proactive in getting the new prescription. So if a patient comes in and they've been discharged we know we have a window of opportunity to get any of the issues sorted, anything new started, do we need to order it in, do we need to do anything different, do we need to adjust the timings of the medicines? All those sorts of things, it allows us to plan for.

HP17
R: …in the past…I mean I've been a pharmacist for quite a few years, 15/16 years; previously you might get a discharge sheet, you might not get a discharge sheet. There's a lack of information, clarity from…There's a lack of, or there's a loss, or you don't get any data. So, I've seen so many problems. So, here's the hospital, patient comes out of hospital, they then turn up at the chemist and say, where's my tray, or, I've got no medicines, and you don't even know they've come out of hospital, you don't know anything, you don't know if their medicines have changed, if they've not changed, have the hospital discharged them with a week, or not? So…and then it causes massive problems because when you get to the stage when they actually turn up in the chemist and they've got nothing then you need to sort that very quickly and you can't always do that and you can't give stuff out without a prescription, unless you do an emergency supply, and then you've got to bend some rules, whether you want to do that or don't do that, and… But I mean you've got summary care records, which is better now, but from past experience this new system of getting electronic referral is brilliant because you know instantly…you know when they go in hospital, you get an email for that and you can bring it up on PharmOutcomes, and you know when they come out, and you get…all the information that the doctors get you get, I'm assuming the same, or very similar, information. So, I've had problems in the past with one particular surgery, not mentioning any names, that wouldn't even give me a discharge sheet from the doctors, they refused, the practice manager refused to give me a discharge sheet, and this is what she said to me, not mentioning any names, was, 'well the doctor's done the prescription, it'll be right because the doctor's done it, and we're not releasing a discharge sheet.' But what happens if it's not right? What happens if the doctor's made a mistake? What happens if it's been transcribed wrong? It's my job as a pharmacist to check what they've come out of hospital matches what the GP's prescribed and I can't do that if I've not got a discharge sheet.

HP17
I: A lot of that stuff is around getting that information. Now that you're getting the information, via PharmOutcomes, for those 200 people on trays, that's a lot, isn't it, are you…what's the benefit of having not just the list of medicines but all the other stuff that comes with it?
R: So, you get the full picture. So, you'll read the discharge sheet. Ideally, assuming time allowing, I'll try and have a quick skim through the whole discharge sheet, and you get the full picture of the patient, you get to look into it more, you get more detail. Just a list of meds is okay, so you can match the meds against the scripts or chase it up or look for what's going on, but when you've got the full picture of any contraindications, medical history, any medicines that were stopped and started, any that were in hospital but have then been stopped, you've got all the extra detail on, well continue this [inaudible] for so many weeks, or, this is a dose reduction. So, there're all sorts that you're getting off the discharge sheet to find out more about the patient which you're not…which you're instantly getting and you're not…there's no guesswork. You know what's gone on when they've been in hospital, you know what's been stopped straightaway, assuming the discharge sheet's been written clearly and there's nothing ambiguous on it where maybe they've not recorded a dose reduction perfectly, or maybe in hospital they've got their own formulary, or you've got to watch like where if they've doubled up on something because maybe they didn't have it in stock. So, look a bit more in detail at if they've doubled up on, for example, Lamictal, or maybe they didn't have Spironolactone of this strength in stock, so they've doubled up on that one, or maybe they've halved something. But overall it's the same, it doesn't matter as long as you're balancing the same drugs, same strength. But it's great, I like it.

HP17
R: Sometimes on the discharge…sometimes you might get a discharge…there's a care home near us here called (name of care home) and sometimes it'll say on the discharge, back in community, or it might…I mean they could do with being a bit clearer on the discharges. This is the bit that's a bit vague. So, sometimes it might say on the discharge sheet, back in community, or sometimes it might mention the word (name of care home) and then we know, well they've gone in (name of care home) but are there still in (name of care home) or have they moved from (name of care home) and then…and they're in respite and then they're then going back into community, into their actual residential home? So, it's a bit ambiguous there when that happens and that, when you've got to get on to the surgery, start asking more questions, or even possibly ring up (name of care home), but we practically never do that, but then it becomes guesswork, and then you're chasing, and then you ring the surgery, ring (name of care home), look at the discharge sheet again, get the surgery to chase up what's going on. But there's only a limited amount of time because we're under huge amounts of pressure.

HP17
I: Yeah, we were saying about communication with the GPs. Has it…having all this information has it improved the communication with the GPs, or has it made it more difficult, has it made it…or is it the same, or what? What changes have happened there, if any?
R: So, what I would say is it's reduced my stress to the point that you're not stressing about getting the information, so you're not mithering the GP as much. So, we're not…I wouldn't say wasting the GP's time but they could be doing more important things, dealing with their patients rather than us asking them to send this email. Fax machines are being phased out. Previously we would get stuff faxed or picking up the physical paper. So, there's less mither for them. So, we're not…it frees up their time because we're not on the phone chasing discharge sheets. It improves the data transfer. So, you know, it's…it just streamlines the whole thing really, it simplifies things.

HP17
We've got the same data that they've got. We're protecting the GP in a way because we now have the full information rather than bits of it. So, we can get…because we're getting this full picture you're not missing stuff. So, you can look at the full picture of the patient, look…think in your head as a pharmacist, does it look right, is it okay, is there any risk here, are there any clinical issues to discuss, do I need to query this with the doctor? Whereas before we might get no information at all. So, overall there's less risk to the patient, less risk to the doctor of prescribing something wrong, or, say…no, say he does prescribe something wrong, or miss something, which is exactly what happened a few days ago.

HP2
I: But is there a benefit of using the PharmOutcomes system as relative to email?
R: Yeah. It's quicker. Yeah. It's much quicker and you know…because you've got the little picture on the PharmOutcomes you know where you're actually sending it to, with the address on there, the name and the postcode. So, you know it's going direct…well hopefully, going direct to that chemist.

HP2
I: So, in terms of timeliness then, if the previous service wasn't very timely… they didn't get that information very quickly. Why is having the information quicker, better?
R: For us or for the chemist?
I: For both.
R: Well, for us you know that…well hopefully, that the chemist is getting it there and then. They are receiving it quickly, therefore they are saving money, they are saving time, and they basically know that patient is in the hospital. So, they are waiting for them…for us to let them know that they are back out with any changes or…
I: So, presumably when the patient is in the hospital, they can just… […]
R: Yeah. Because if there are changes then that blister pack is just thrown away wasted.

HP4
I: So that communication, how can that help with that safety or how is that going to help…?
R: Because inevitably I see it all the time when patients are discharged from hospital, we see so many problems with mistakes, errors, prescribing errors, when patients go from one setting to another, when they go from primary to secondary, secondary to primary, you're constantly facing that battle of mistakes in that transition, and the aim really is to make sure it's communicated reliably. So if the chemist are getting an electronic discharge of like a snapshot of exactly what they were discharged with, that takes out that kind of element of, you know, they've got the information there so they can then hopefully act on that quickly, much more quickly than relying on a fax two days, three days later where there used to be quite a significant delay, things can get changed quicker, so if it's blister packs they can get their new blister pack up and running, they can nag the GP to get the new prescriptions through. So hopefully the next time the blister pack comes out, it will have the correct medication in, not medication from like preadmission.
I: So it's about having that correct…making sure the patient is on the correct medication …
R: Yeah, exactly, yeah, making sure changes have been communicated accurately.

HP5
I: When…before it happened, what were your sort of expectations of what, what the service might look like, compared to what, you know?
R: I did, I must admit I thought the community pharmacy referral would just isolate the medications that the patient is on and send that to the pharmacy. But my understanding is the discharge summary actually goes along with them, the list of jobs to the chemist. That aspect is important in letting the community…letting the patient know that the community pharmacy is receiving all this information.
I: Right, why is that important?
R: Because they may have information…people's imagination of a community pharmacist that all they should have is drug information, but they don't see them as having any other... I've worked in HIV and infectious diseases before, there is certain information that hospital will have that perhaps you might not want a community pharmacy to have which regards the status of patients, are sort of like treated the same way. There has got to be other information that perhaps people may feel the community pharmacy doesn't need to know. It might be inclusive on there, ie if they are an alcoholic or if they abuse drugs or if they whatever, community pharmacy doesn't necessarily know the background to that patient or certain aspects of a patient. So, they need to know that everything is being sent via the community, e-referral to the patient.

HP5
I: Yes, that's really interesting actually and you talk there a lot about that continuity of care, because of actually one…and you also talked there about the…in terms of that, in terms of patients because one of the questions there is, you know, do you think the service can improve care, will it improve care?
R: Yes, definitely.
I: In what, why will it do that?
R: Because the information received is accurate. It really is accurate; everybody is getting the same information.
I: Yes, precisely.
R: And you've not got a break in information. Because a patient, a scenario we've had in the past is a patient, we might send a summary to a patient, they might go to community pharmacy to get a supply of the drugs, community pharmacy isn't aware they've been sent into the hospital and gives them something, a new blister pack. Perhaps they might have had a couple of prescriptions left from prior to the admission and then they get drugs that have been, that has been stopped that caused hospital admission.
I: And could have caused a hospital admission.
R: Exactly, so yes, so definitely because there is communication between all services involved and you've got now exactly the same information moving between all of them. The risk of having errors like that made is minimised.

HP7
I: And, does anything happen on discharge?
R: So, discharge is very important because they'll get an electronic copy. So, you need to make sure that everything that was prescribed at the times the patient was admitted. So, you know, for example, if they had aspirin in the morning, you need to make sure that on discharge it's prescribed as aspirin in the morning, because they will just get that copy, it will have no written annotations.
I: Yes.
R: So, you need to annotate, if it's in the blister pack electronically, if it's not in the blister pack, you need to say what you've supplied, you need to tell them about…yeah, so that's quite…I think discharge is very important that you do it correctly, yeah.
I: Why?
R: Because they will just get it automatically and it's not out of your hands to be honest, what they get, unless you have checked it beforehand.

I: Yes. So, has having the referral made…
R: It makes it more…you've sort of got a bit more to check at discharge. You need to be quite sort of…
I: You need to be more accurate.
R: Accurate, yeah, than you could have been. And, that's more accurate as in, on the electronic system it needs to be accurate, whereas beforehand you could just annotate it with pen.

HP7
R: Yeah, 'cause we get a lot of practice pharmacies that call up and don't know why we've done stuff.
I: What's the communication…?
R: Because sometimes discharge summaries are really incomplete and this way, doing this on e-referral means that, we double check that it's complete.
I: Yeah. I mean, that's the thing that we are really interested in.
R: Yeah, so we sometimes re-write certain or write things that aren't there. It's like things have been changed but it's not got documented so we'll say, we will fill in the gaps. Sometimes those gaps can't always be filled so then things get sent to GPs and they don't know what's happened and they'll call up here and find out.

HP7
R: I think it's had quite a lot of benefits. Because it saves time ringing pharmacies, it's made us be a bit more careful; add more information, a bit more accurate on discharge. We've been able to communicate in a more timely manner to community pharmacies particularly out of hours.

HP9
R: Yeah, mistakes. Communication is the biggest one. I think they usually don't either tell you on the discharge summary that something's changed or stopped or started. And then you compare the medication list, then it just…obviously, there's one that's on your repeat that's not on the discharge or there's something new on the discharge but they've not told you that they've started it or anything, so you have to…I dig through it because I've got time to actually look through it. That's part of my job but the GPs – some of them – don't have the time or they just look at this part that says medication changes and they go, oh, no changes. And they might just leave it and just mark it as complete. But then actually it's not matching. But I dig through it. I just have to go back to…because I can access the (name of place) system, you know the EPR system, so I can check to see what's happened there. So that's what I usually do, and then I'd have to submit an incident report because…which I've had to do a few recently. So this is the main thing that I see.

Theme 2. Availability and accessibility of information
2.1. Code - Accessibility, availability and the sharing of knowledge and information

C2_C3
R1: Yeah, they've not been bad. Like I say, they have explained things. The pharmacists at the hospital, they've been really good. They've not just like done it and then not told you, they've explained. The day before discharge she phoned me up twice. She phoned me up and then explained about the meds she was on.
R2: It's because it's controlled, because it's a controlled drug.
R1: And then she phoned me up later on to explain that she didn't have this particular tablet, they didn't have it, but she has been on this other one before. One time the chemist didn't have it and the doctor prescribed this other sleeping tablet, zopiclone, so they're pretty good at explaining.

HP1
R: Okay, so I hate the notification, 'cause the first time I had it, and I'm quite computer savvy, my mum was a computer…anyway, we used to do everything on computer from the day we were born, so I know how to use computers. But, when I looked at it, I thought, oh God, what the hell is this? So, what it is, is they send you an Excel, I'm trying to open it, but it's not really going to be of benefit, so I can describe it, so they send you an Excel spreadsheet, with…this has actually been, so I've actually changed it so you can see the words, but initially when I got it, there were just codes on the Excel spreadsheet. So, you have to then change the format to work like Word, so text, and then you'd see it. So it's quite simple but someone that doesn't really know, they'd kind of be looking at it for about ten minutes, thinking, what is this, I don't really know what this is?

So, I think that's now been fixed, because I raised it with (name), and I think (name) now will change it, before she sends it out. And that was my initial experience. The other thing was, there's no NHS number, so what happens in general practice is, I receive this email via my NHS email, and then I then, there is a column that said, where I eventually found, there is a column that says what is the issue. And so, for example, the first one I got, the issue was medicines stopped in hospital, still on repeat. I don't actually know what the medicines were, that's it.
I: It's not told you which of them, or all of them?
R: Not told me which, yeah, but I just guessed that another reason, going back to having training, it would be nice to actually see how it looks on the hospital side, so we have an idea what that has come from. Was it actually someone free texting it, or was it just a tick box?
I: Yes, a drop tick box.
R: If it was a drop tick box, then it would make me think, okay, it might just be a general thing, it might not even be an issue. So, that just helps with our thoughts. So, I knew that there was an issue, but I didn't actually know, there was only a patient ID. Now, in EMIS, and Vision, you can't get the patient's records via their hospital ID, that's only for the hospital system. So, what I had to do, was log on to Sunrise, now you wouldn't appreciate it if you haven't done it, it is easier now, but some practices still have some block on the computer, so sometimes it's difficult to log onto sunrise from a practice, which is the hospital EPR. So, what I had to do, was log on to sunrise, type in the hospital ID, find the patient, go onto the patient information to find out what the NHS number is, so I've got the NHS number, go back to the practice system, find out who…and this is just to find out who the patient is.

HP1
R: So, the first day I got this, I couldn't log onto EPR, which is sods law but they had updated the system at the practice, and I couldn't log on to EPR. So, it was very stressful for me, because I knew that something wasn't right, because we were issuing something on the repeat, but it had been stopped in hospital. I didn't know whether it had just been stopped, or is this like, has the patient been having it for about two weeks? And I didn't know any other information. I didn't know who the patient was. I didn't know what to do. Didn't know what pharmacy it was, because there's no information about who the pharmacy is, which pharmacy it is, so I had to guess and just called, yeah, there's no information on what

pharmacy it's come from. So, I didn't like it. And, I did speak to (name) about it, and said, why isn't the NHS number in there, just so it's fast? And she said, that the way that it's sent via the PharmOutcomes, is they can't generate the NHS number, so it can only be this way for some reason, I don't know. So, yeah, in fact, it had got a pharmacy number, (name of pharmacy), but there's hundreds of (name of pharmacy), pharmacies in (place), so I didn't know which, so I just called the one next door, and luckily it was them. So, that's how it looks like in practice.

HP1

I: And also, then, having to do further things, as you…I mean, do you think, what's the sort of, how's this going to move forward then, in terms of, is this going to stay the same like this, or is this going to be, do you think this'll be…?

R: I mean, I think that there's a lot of things that don't need to be in this report that we get sent. I mean, this is assuming that we're not going to be in the e-referral system, and it's going to be a two-way, if it was a three-way, it would be a different story, 'cause we could actually see the system, and yeah, but currently, as it stands, we're just getting these reports as and when we need to. And the reports are really, there's information that we don't really need, like DISWA B3, ah, that's probably Discharge Ward.

I: Discharge Ward, yeah.

R: I mean, yeah, that's useful, because if we wanted to call the ward, and we know where they got discharged, yeah. But there are things that, I think there's information that we don't really need, and the information that we do need, probably needs highlighting more. We also definitely need an NHS number, or an easier way to get to know who the patient is, because not everyone can access EPR unfortunately, which I think they should be able to, but even if they can access it, it takes time. And the way that we access EPR in general practice, we have to log on via [Cytrix 00:22:52] and then we have to go on to our hospital system, and then we have to select Sunrise, and then we have to log onto Sunrise, so already there's three log ons, yeah, just to get onto the system, so it's not really that easy to do, yeah, and it takes time.

HP10.

I: There's more quality information?

R: Yeah, yeah, where before it was handwritten very often and sent via the fax, it got even worse in terms of quality, you couldn't really read properly what that information was. I've got a feeling this digital we get now and it's even more...there's more in it, there's more information. I don't think it's the same as the doctors get, I think they get an even bigger one, but we, for example, now can find out about what the patient was admitted with. I always scan through it, I always find time to, and I read, oh, this and that and the other, I read the little history. I'm not an expert, I'm not a GP, I'm not a doctor, so I don't... I mean, I understand most of it but I don't understand everything, but it's helpful to know that, say... I'll give an example. If someone was...it's happening quite often, I think 20, even 30, per cent of my patients will be admitted due to falls, they'll be falling, they fell in the house, they hurt themselves, taken to hospital. And I'm thinking, oh God, looking at what are they on. Oh, they take alendronic, they take this, this and this, and I'm thinking, Christ, how did I not pick this up earlier? Oh gosh, so many strong painkillers, bloody hell. Maybe we should be more conscious about it. You've got to get a better and bigger picture. You can understand this patient, oh, hold on a minute, oh Christ, I should maybe make sure that she... Doctors often don't think about those things, they will issue another painkiller that could make them drowsy, and I'm thinking, hold on a second, she was admitted to hospital with a fall not so long ago. Just think about that. Not so long ago, where a lady was on very strong pain relief and their doctor must have given...went for a home visit. So I just picked it up thinking, oh, Christ, she's given her this. Oh, no, no, she shouldn't be on it, she's already on this, she had this fall. And I just rang: listen, this and this happened not so long ago. Oh, yeah, yeah, thanks (name) I will change it, so that was good.

HP11

I: Was there a difference between what information was then or…to what you get now?

R: I think we didn't always get the full…sometimes we would just get the medicines, whereas now, we get this patient was admitted on…and sometimes we did. I think more recently we did but in the past you'd just get the sheet with the medication on. Whereas now it says…

I: So these are the new medicines ….

R: Yeah, whereas now it says why they went in, where they've been discharged to, how long they were in hospital, what changes they've made and where they've gone home to. Where they've gone to, because they don't always go home. Sometimes they go…

I: No, 'cause sometimes it's…

R: …into an intermediate care or…

I: …or to care homes, yeah.

R: …whereas we've got the full information, it's the full information that the GPs get. And then we can then…I think the only problem that we've found is that it's the pharmacist at the practice who actions that medication, that discharge and I don't think he…he's saying he doesn't always get it. He doesn't see that email. So whether it's just being sent to a general practice, an email for the practice and it's never getting forwarded to him…I mean, it doesn't really matter because we're sending it to him anyway. But he's not always seeing it before we're saying, (name), have you done this script?

HP12

I: Talking about the pharmacy referral service then, can we talk about when it was first introduced. Why do you think it was brought in, what's it there for?

R: I think to just improve communications between the two. That's what's worked best for us. The fact that we actually get told when one of our patients goes into hospital, is a big thing. Because quite often they'd ring and say I'm out of hospital, they've changed my medicines, we didn't even know you were in, we've still been sending you medication every week, where's that been going, you know. And to get the discharge information as well. 'Cause sometimes we would get that faxed through, sometimes we wouldn't. They went through a spell where they wouldn't fax it, trying to get it off the doctors sometimes is difficult. So, it just makes the whole process so much easier, because we are getting the information quickly and the accurate information.

HP16

I: So you now know why that patient's gone in, what's happened, what changes have happened, what's been resolved what [voices overlap 05:25].

R: Yeah, I mean it's down to the quality of the discharge, the discharges do vary in quality from being very, very detailed to being the bare basics, but we still have more information than we did to help that patient. And the key parts are it's usually medicines started and stopped because that stuff is really key for our role in that transfer of care. That's the whole point of it is actually has it been stopped, has it not, has it been missed? We don't know and the GP practice might spend a few days doing that but we don't know that they're spending those times chasing it up or getting the information, all we see is this is what's going on. And then the other part of it is it allows us to be a bit more proactive in getting

the new prescription. So if a patient comes in and they've been discharged we know we have a window of opportunity to get any of the issues sorted, anything new started, do we need to order it in, do we need to do anything different, do we need to adjust the timings of the medicines? All those sorts of things, it allows us to plan for.

HP16

R: They need to be actioning those things as well. And I think the training has very much attended by and focussed on, the actual pharmacist, owner, manager, whoever that is, and although the teams were encouraged I think the pharmacies didn't get that the teams are going to be doing a lot more than the actual pharmacist will.

HP17

I: A lot of that stuff is around getting that information. Now that you're getting the information, via PharmOutcomes, for those 200 people on trays, that's a lot, isn't it, are you…what's the benefit of having not just the list of medicines but all the other stuff that comes with it?

R: So, you get the full picture. So, you'll read the discharge sheet. Ideally, assuming time allowing, I'll try and have a quick skim through the whole discharge sheet, and you get the full picture of the patient, you get to look into it more, you get more detail. Just a list of meds is okay, so you can match the meds against the scripts or chase it up or look for what's going on, but when you've got the full picture of any contraindications, medical history, any medicines that were stopped and started, any that were in hospital but have then been stopped, you've got all the extra detail on, well continue this [inaudible] for so many weeks, or, this is a dose reduction. So, there're all sorts that you're getting off the discharge sheet to find out more about the patient which you're not…which you're instantly getting and you're not…there's no guesswork. You know what's gone on when they've been in hospital, you know what's been stopped straightaway, assuming the discharge sheet's been written clearly and there's nothing ambiguous on it where maybe they've not recorded a dose reduction perfectly, or maybe in hospital they've got their own formulary, or you've got to watch like where if they've doubled up on something because maybe they didn't have it in stock. So, look a bit more in detail at if they've doubled up on, for example, Lamictal, or maybe they didn't have Spironolactone of this strength in stock, so they've doubled up on that one, or maybe they've halved something. But overall it's the same, it doesn't matter as long as you're balancing the same drugs, same strength. But it's great, I like it.

HP17

R: So, the sooner we get that information then you're reducing the delays to the patient getting their medication in community. The limitation of this system is the surgery. So, even though we get the discharge sheets I still can't do anything until the surgery accept the information at their end, process the discharge sheets, pass it to the GP, get the GP to then do new prescriptions, whether they be monthly, whether they be weekly depending on the risk, and then those prescriptions have got to come to us. Some of the surgeries don't do anything. They'll just sit on that information, it'll just sit on their computer, and they'll do nothing, and then the patient…

I: Yeah. So, you're getting that information, which you didn't get before, but you can't act upon it until you've got the script, can you?

R: We can provisionally act upon it. We can make sure we've got the drugs in stock, we can put the patient on a sort of chasing up shelf and have the…make sure…see what's changed, look to see if we've got any existing scripts. So, if they've put on the discharge sheet, no changes, okay, no problem, we'll just continue the trays as they were. We might use existing scripts, we might request new scripts if we need them, but if the surgery…or we can…it prompts us then to phone the surgery and say, wait a minute, we've had notification this patient's come out of hospital, we need new prescriptions for everything, why have you not done it? Oh, it's sat in the doctor's room, or, oh, sorry about that, we've had it a week but we've not done anything.

HP17

R: So, how many patients have I personally prevented from dying, being re-hospitalised by GP prescribing errors before versus after the new electronic notification system? So, I mean I'm getting old now, I've got some [inaudible 00:00:28]…I don't need to tell you that, but I've been doing it 15/16 years but… Yeah, so getting the full information is great, it reduces prescribing errors.

I: Because you have more information to check for the GP?

R: Yeah. So, something might have been stopped but because the GP's maybe not noticed or was rushing and he's stressed in his job, or her job, and they've not noticed that this has been stopped, or that's been started. So, you could get multiple…that one that I just told you about was two diuretics together. You could get all sorts of different changes, it could be anything, and you could get something stopped that the GP's still continuing. So, anything I notice that doesn't match the discharge sheet I'll query. But my dispenser here, when I came, she was a bit reluctant to query and go against the GP and say, well why's this on, or, why's that not on, because once the GP went mad at her and that then changed her attitude. So, he said…this GP said, 'why are you questioning me, I'm the GP, I prescribe this?' Well it's not on there.

HP17

R: Yeah. So, I disagree. The more information the better.The more information we have then we can reduce the risk to the patients. Yes, you've got to log on to PharmOutcomes, yes, you've got to spend how many minutes accepting it and printing it out, but overall you've got the information. So, the time that you're spending downloading the information, printing it out, yes, it takes a few minutes, maybe five minutes, but that time you spend is valuable time which will then benefit you because of the knowledge that you've gained.

I: About that patient?

R: About the patient and the time that it's saving so that you're not in a situation where somebody just walks in and says they've got no medication because you've already pre-empted that because you've had this all planned out since the day they got discharged.

HP3

R: I think it would be a good thing, so they're aware. I'm not too sure how...like what the roles are of the practice pharmacists, but I think it would still be helpful, especially on discharge. I think it would be more helpful on discharge, probably, if they had that.

I: [Voices overlap 0:22:26]

R: Yeah, if they had that information.

I: What would they be able to do with that information?

R: So, again, I think they can follow up with counselling for the patient. If something new has been started, they can prepare, I guess, for when the patient gets back and make appointments. I think it's just really good transfer of care, pretty much.

I: Yeah, and having that seamless thing so that, as you said before, everybody knows what's going on.

R: Because I don't think patients will ever say... Well, I don't know why a patient wouldn't want other healthcare professionals that are looking after them not to know what is happening. Because if I were a patient, I would want everyone to know...well, all the healthcare

professionals to know this is going on, because they're looking after me. I don't think there's anything that I'd want them not to know. Especially because they understand that we're healthcare professionals, we're registered, we have to abide by rules, so it's not like we're going to be talking to random people about what's going on.

HP4

I: One of the things when health professionals say to me that that saves time or this only takes so much time and now we've saved some time, one of the things I often say is then, so what do you do with that time?
R: Yeah. Well, we see more patients, which is good, or you have more time to do clinical things rather than just kind of, you know, desk…
I: So it takes away some of this administrative work…
R: Yeah, exactly.
I: …and brings you back into those patients.
R: Yeah, that's right, which helps, you know, so we can then have more time to correct mistakes and prescribe on the ward and things like that. So yeah, I would say definitely saves time. I still the odd time, the only downfall with having the e-referral sometimes is you still end up having to ring the chemist on admission to clarify certain things. So if for example like a patient doesn't have their medicines with them from home, you might be able to see from their GP records who their nominated pharmacy is, but you might still have to ring that pharmacy just to check, do you do blister pack for them or do they not, it looks like you do, but I always kind of ring just to check, because I think it's easier just to sort it out then than for someone to get a decline in like two days' time or whatever. So I kind of ring them or sometimes might want to clarify the timings of the drugs, so if it's not clear, if they're on like 20 things and they're all white and you can't work out which one is at night-time, which one is teatime, you might just need to go through the blister pack with them over the phone and say, is that night-time, or if there's a mistake sometimes as well in the dispensing, in the actual blister pack you might say, is that a mistake, the label is not there but looks like it's in or… So there's still times where we have to ring them, but I would say overall it definitely saves time.

HP8

R: Definitely, yeah. It doesn't take me a lot of time out of my day. It's fairly straightforward what I have to do with it and then when the NIPPS team chase it up they get quite quick answers about what's happened.
I: I think on that note, unless you've got anything...?
R: Just that in setting up the generic NIPPS email, that has then opened up more communication between the hospital and the NIPPS team, so (name) circulated the NIPPS email and said that you can send us non-urgent patient queries. So, again, I triage those and it's literally just this is the generic NIPPS email send something through. But we have had urgent patient queries sent through on them. So we need to work on our messaging.
I: Yeah, because if you're get anything urgent, because you're not looking at it regularly enough for...
R: I am looking at it regularly but we don't have the team in place that are in the patient surgeries. As I've said every...
I: Every day, no…yes, precisely.
R: ...like there's not a nominated pharmacy. It might be a week before a NIPPS team member works in a surgery due to...

P1_C1

I: Yes, okay. So, when these medicines were first prescribed, what sort of information were you given about them, who did that, who talked to you about that?

R2: In all fairness, there wasn't that much information given.
I: Right. Interesting.

R2: So, it wasn't given as in what might cause side effects. And, with it coming in a venapack; there is no information as to what side effects may be caused.
I: Yes. So, when you get the Venalink pack, you don't get the little patient information leaflet.

R2: No information at all.

P1_C1

R2: So, like if you get it individually in packs like that. You get your information.
I: Yeah.

R2: But even sometimes with these, unless it's in, like mum gets these, or she did do and she used to get them weekly but there was no information in the box.
I: Right.

R2: From the chemist either.
I: Yes.

R2: So, you don't actually know what your side effects are or what you have got to look out for.
I: Or what you have got to look out for or what you might need to avoid taking as well.

R2: Yes. So, certain food types. Now, one of them does say on it, avoid grapefruit.
I: Right.

R2: But that's the only bit of information you get, like not drinking alcohol.

P1_C1

I: So, how do you feel about that, the when you were in hospital, those changes were being made, what do you feel about that, the explanations that were or weren't given?

R2: Well, there wasn't much in the explanation as to why things were changed other than the salt levels needed to be brought back up. But some of the tablets that mum's on I've never heard of and, again, they weren't explained as to why they were being used.
I: Right, yeah.
R2: So, I don't know. And, of course, there is no information to say, you need to watch out for this or avoid this.
I: Yes.
R1: But I take 17 tablets a day.
I: Right, yeah.
R1: And, in my own mind, I don't need all them.
I: Right, yeah. R`: You know.
I: But has anyone talked you though and said why you need to be on those 17 tablets?
R1: No.
R2: We know about the bipolar tablets and the reason why that is, because it's mood levels and making sure that you are not overactive.
I: Yeah.
R2: But a lot of the meds that mum's on she has been on for many many years. So, you know.

P1_C1
R2: That's why they wanted to put us on the new drug, which is the apixaban.
I: Right, okay.
R2: Which again, wasn't really explained about, it was just saying that you didn't have to go for as many blood checks.
I: Yeah, precisely.
R2: But they couldn't tell us what possible side effects were because it's a newish drug in terms of medicine.
I: Yeah.
R2: At the time, I think it had only been around ten years.
I: Yeah.
R2: But the people that they'd worked with and dealt with, patients they said they've had nothing that they could say was really serious and they may need to revert back to the warfarin.

P1_C1
R1: But the GP, Dr (name) she explains everything. She is really good.
R2: Well she does and she doesn't. She doesn't explain what the tablets actually do.
I: All do.
R2: And what interacts with them.
I: Yeah.
R2: She's good in the fact that she's aware of what mum's conditions are and why the tablets have been given.
I: So, basically you would like, more information would be better?
R2: Yeah. Even with the venapack, even if, obviously if it was going to be regularly changed, then that would defeat the object, but if you've got regular doses and you are on them for however many, even for 12 months, if one piece of information, like a piece of paper of the all the drugs that's on and what the interactions would be, what to avoid, what not to avoid, if you got that information, with the pack, just the once, so you've got them here. And then when it's changed, that information changes with it.

P2
I: Have you talked to the chemist at all about it, or has anyone talked to the chemist?
R: No, because you see, I used to get it from…because my doctors is at (name of surgery), and I used to get me medication from there, but when they put it in the blister pack they moved me to (name of pharmacy). I don't even know where (name of pharmacy) is, I haven't got a clue where it is, so they're just sending these now from there and I don't know…
I: (name of pharmacy)?
R: I've no idea where it is. You know, I haven't got a clue. So, it just comes from (name of pharmacy), and that's all I know.
I: Right, okay.
R: You know, I don't know anything else. You know, I mean, before it came from (name of surgery) and I knew and if it was right, I could go in and get the prescription.
I: Right, so you don't even know where it's…
R: No, where now they just give me this because of the way that I am forgetful. And I just said, well you know, I can't remember what I'm taking so they said we'll give you the blister pack.

P3
R: Well, I said to a couple of the pharmacists, they have different ones, and I said, why have I got these headaches, is there anything in these meds to do it, and he looked through and he said, no, there's nothing there that should do it, but the surgery kept changing the strength of the Ranexa and I've started off at 375 on that a few years ago, then it was increased to 500 and I've been on that for quite a while. And one of the tablets that had come out the very first time in May was an angina tablet and that's what we put the headaches down to on the day I was released, and after a few days of that I stopped taking it hoping it would go away, and then they suddenly said, right, we'll up the Ranexa to 750. So after a week I rang them back, I said, it's not making any difference, the headaches are the same, so they dropped it to 500, again, and then they dropped it to 375, and nothing was making any difference with my head, so eventually they stopped it altogether, and I don't know if it was that after a couple of days in hospital when it started to ease this time. But it's been something that…it's like the shortness of breath, they've not been able to put their finger on what the problem is, and it's the same with the headaches, the medication changes has done nothing…

P3
But now it's different, I mean, I don't know if you know Dr (name) at all, he's a consultant at (name of hospital) and he's one of the cardiac consultants, great bloke, yeah, it's first name, have a laugh, the last time I saw him he said something and my wife said, oh, he's on his computer playing games, so he said, oh, what do you play, so I said, I play World at War, oh, ruddy hell, he said, I used to spend hours on that playing on the Xbox. And the guy in urology is putting the camera down and all that and he's showing me the pictures, he's telling me, oh, that one looks alright, you know. They just make – well, 90 per cent - make everything as simple, relaxing and easy as they can. So I'm quite happy with the way things

go. I mean, okay, it would be nice…I have been going to the cardiac rehab and they do talks on new medication, you know, but they don't always break it down, it's in groups, ACE inhibitors and…

I: Yes.

R: Yeah, so they talk about it in groups, but it would be nice to know properly what each one does, you know. Yeah, it would be nice, but I won't put pressure on anybody if…I mean, the GPs are run off their feet, aren't they? The pharmacists are no better, you know, hospital under-staffed everywhere. So no, if they want to tell me, I'm sure they will tell me, you know, but it's usually just when there's changes, they just tell me what's changed and always say why, yeah.2b. Patient knowledge of e-referral service, discharge and medicines

2.2 Code - Patient Knowledge of e-referral service, discharge and medicines

C2_C3

I: When we talked about the referral to the pharmacy, did anyone talk to you apart from (name) when she explained the study? Did anyone talk to you about the referral to the pharmacy, did anyone tell you about the pharmacy would know your mum was in hospital and stuff?

R1: No, but I assumed that, because it's happened before. They'd phone me up sometimes and said is your mother out of hospital now. In the past, the chemist. Not this chemist, the previous chemist, because my mum had been under him for years. So I've had a couple of times where they've phoned me up and said I'm just checking is your mother home yet and I've said…because just checking she's home with her about meds and that. So, yeah, seems to be pretty…

I: But they didn't do that this time perhaps because they…

R1: No, I've not heard off them, no, but having said that the chemist…

I: They should have actually now known that that was the case with the new service.

R1: That's it, but there again it's a different chemist. The old one was really, really on the ball.

HP1

I: In terms of patients then, what do you think the benefits are going to be to patients? Are those benefits already being realised perhaps?

R: I don't think by the patients, I don't think they have any idea. I don't think. But some do say, oh right thanks, some are surprised that you knew something had happened in hospital, but I don't think they know how we know and how we're told, yeah.

HP5

R: Less, you have less, right you get less admissions, hospital admissions due to drugs for a start. Patient educated as well, patient education, they'll know exactly what they are supposed to be taking now because they get the same information from all the disciplines involved. Patients can get confused if they get one information from here and another set of information from here, so it's less confusion with regards to their care. And they are less, obviously they are less distraught and traumatised by people not talking to each other.

I: No, knowing what that…yes.

R: About what they should be getting.

HP8

R: Yeah, exactly, it can be very confusing for patients I think what they are supposed to be taking when, especially when they've got lots of different drugs. So, yeah, I think community pharmacy have a role in helping patients to understand what they're taking and why.

HP9

I: Yeah, 'cause in terms of patients on Venalinks, I mean, do they know why…what they're taking?

R: The Venalink medication?

I: Yeah.

R: A lot of them don't. There are a proportion of them that do actually, are really clued on and know exactly what they take, but a lot of them because it is in a blister pack, they don't tend to know what's in there or what's changed. They know maybe what tablets are in there, they don't know the doses and what…because they've changed so much and they don't get told what's actually changed, so that's what you tend to find with them.

P1_C1

I: About your medicines, about your medicine taking, which is interesting. So, let's move on to the recent visit to (name of hospital) and talk about that. When you were in the hospital, did any medicines get changed?

R2: Yes. In fact the, my mum's on flucloxacillin because we thought there was an infection which was penicillin based.

I: Right.

R2: Unfortunately, she had a reaction to that, which didn't start 'til the second lot of medication kicked in.

I: Right.

R2: So, we were looking for the signs and symptoms on the first…which we did get the information in the pack, none of that related to what was happening to mum because nothing happened in that first prescription of five days' worth. The second lot of prescription when we went to the GP, so we were looking for the signs and symptoms but a rash appeared which wasn't part of…

I: So, this is before going into hospital.

R2: Yes. And, then we ended up going into hospital.

I: Right.

R2: And, then the antibiotics were stopped because mum was diagnosed with heart failure.

I: Right.

R2: Because of low salt levels, so her liver wasn't doing the job that it needed to be doing, which impacted on the kidney's which impacted on her heart.

I: Yeah.

R2: So, then the Simvastatin, which I think is the statin was stopped, whilst she was in hospital. The omeprazole which is the gastric was stopped the flucloxacillin was stopped. And, the water tablets, the flumethiazide which also can cause low salt levels.

I: Right.

R2: This is what we found out later, can cause the problems that affected mum why she went into hospital. So, then they then had to increase the water tablet to help reduce the fluid.

I: Right, yeah.
R2: And, then but monitor that to a point where it was safe to take her off that to put her on a lower dosage. So, that's what the change was.

P1_C1
R1: But I've been on water tablets for 54 years. 1965 I was first prescribed them, when I had my fifth child, which was (name). And, they don't do a blessed thing.
I: Right.
R1: They don't take…
R2: Well they kind of do, but I know what mum's saying she doesn't see the effect.
I: Yeah.
R2: But if she wasn't on them.

P1_C1
I: So, how do you feel about that, the when you were in hospital, those changes were being made, what do you feel about that, the explanations that were or weren't given?
R2: Well, there wasn't much in the explanation as to why things were changed other than the salt levels needed to be brought back up. But some of the tablets that mum's on I've never heard of and, again, they weren't explained as to why they were being used.
I: Right, yeah.
R2: So, I don't know. And, of course, there is no information to say, you need to watch out for this or avoid this.
I: Yes.
R1: But I take 17 tablets a day.
I: Right, yeah.
R1: And, in my own mind, I don't need all them.
I: Right, yeah. R`: You know.
I: But has anyone talked you though and said why you need to be on those 17 tablets?
R1: No.
R2: We know about the bipolar tablets and the reason why that is, because it's mood levels and making sure that you are not overactive.
I: Yeah.
R2: But a lot of the meds that mum's on she has been on for many many years. So, you know.

P2
I: So, if we could talk about the medicines you take to start with, can you just tell me about those, so what medicines do you take, and what for? You know, what are you taking?
R: I don't know what I take them for.
I: You don't know.
R: I just take them, you know, and I don't know what they're for.

P2
R: I mean, I take all these, and I haven't got a clue what they're for.

P2
R: Yeah, and then they just keep…when I go and I've got a problem, oh we'll just change it.
I: But no one explains to you why?
R: And nobody explains why they're changing it, so of course they just change it and they don't have the time to sit down and talk to you, this is the problem. You know, you go and oh it's if you go to your GP, they haven't got time to discuss everything, so of course, I'm on a lot of medication but I don't know what it's for.

P2
I: Have you talked to the chemist at all about it, or has anyone talked to the chemist?
R: No, because you see, I used to get it from…because my doctors is at (name of surgery), and I used to get me medication from there, but when they put it in the blister pack they moved me to (name of pharmacy). I don't even know where (name of pharmacy) is, I haven't got a clue where it is, so they're just sending these now from there and I don't know…
I: (name of pharmacy)?
R: I've no idea where it is. You know, I haven't got a clue. So, it just comes from (name of pharmacy), and that's all I know.

P2
I: Do you think it's better having the blister pack then?
R: Yeah, because…but I just take it, I don't know what it's for, but I just take it.
I: Keep taking the tablets.
R: I sort of think well, it's up to them to make sure the doctors and the chemist, to make sure that they're giving me the right medication. I don't know what I'm taking.
I: Do you think they are…do you think…how…are you sure they are giving you the right stuff, I mean is that…or do you worry that they are giving you the right things?
R: I don't worry about it because as far as I'm concerned, they should know what I need. I don't know what I need but now…
I: So, you don't worry about that?
R: I don't worry about it, because to me, it's not my responsibility to take the right medication, its up to the doctors and the chemist to do the right thing, and I just take it and I think, oh well, and then I look at all them and I think, what are they all for. You know, I mean it's got the names on.
I: But the names don't mean much to you.
R: The names don't mean anything to me. You know, oh swallow it whole, take it in the morning, you know, so that's what I do.

P2
I: You said when you went to (name of hospital) recently, this last visit just, you were discharged well, 9 days ago, or a week ago, you came out of (name of hospital) last time? Was it last week you came out?
R: I went in, yeah.
I: Yeah, so you said that some of the medicines were changed then, do you know who made those changes, who decided on those changes?
R: The doctor at the hospital.
I: The doctor, yeah. And do you know…but you don't know why those changes were made?
R: No. Oh well that dosage isn't right.
I: Ah right, so they told you the dosage wasn't right.
R: So, we'll try a new dosage and see how you go. You know, although, but they don't actually speak to you, and face to face they don't speak to you, they speak to the medical team and, oh well, we'll change that to such a thing, but they don't actually speak to you face to face. And to me that's important, you know, but they don't and of course I think, oh well, they know what they're talking about. I think, well they should know what they're talking about, but do they? They're just changing it to see if it works, and that's all. They're not sure whether it will work.

P3
R: There is a bit of a confusion with it at the moment…
I: Oh, well that could be really interesting then, yeah.
R: It may look very confusing…
I: Oh, because you've got some in boxes and some in blister packs.
R: Yeah. Yeah. That's my current one, which is wrong.
I: Oh right, right.
R: This is the ones that I came out of hospital with…

P3
R: Yeah. Now, with those, I had to take…that pink one, I have to take that out.
I: Oh right.
R: Right, and I have to put these two in, and that statin, I have to break it in half.
I: So currently this has been made up by…
R: My local chemist.
I: Your chemist.
R: Yeah.
I: And this is…so currently some of these bits and pieces are wrong.
R: It's pretty much as it was before I went in hospital.
I: Oh, so they haven't made the changes yet?
R: When I left hospital, the day after I went in to the pharmacy and asked them if they got all the information and they said no, they said, we've had a phone call from the hospital pharmacy and they have told us that there's some changes, so I said, so you've not had the discharge papers, so they said no, I said, have you had the new list of medication, no, have you heard from the surgery, no. So I said, right, I'll ring them, she said, well I'll ring them and have a word. So when I rang the surgery said, we've already sorted it out with (name of pharmacy), it's all done and everything, so I thought, right, that's fine, showed (name of pharmacy) the blister pack that I'd got from the hospital and they said that we haven't had time to get you one ready, your next one, she said, so the one that we've got ready has got all your old meds on and there's no changes, so we'll have to give you that and the boxes of the additional tablets and you'll have to take the Ranexa out and break the statin in half because they'd reduced…
I: They'd reduced the dose, yeah.
R: …dose. So that's the point I'm at at the moment, and my pharmacy said…I said, well I always used to pick it up on a Thursday because my first dose was Friday morning on the new one, so now I have to pick it up on Monday because they've rehashed everything, so I have to pick my next one up next Monday and that should be up-to-date…

P3
But now it's different, I mean, I don't know if you know Dr (name) at all, he's a consultant at (name of hospital) and he's one of the cardiac consultants, great bloke, yeah, it's first name, have a laugh, the last time I saw him he said something and my wife said, oh, he's on his computer playing games, so he said, oh, what do you play, so I said, I play World at War, oh, ruddy hell, he said, I used to spend hours on that playing on the Xbox. And the guy in urology is putting the camera down and all that and he's showing me the pictures, he's telling me, oh, that one looks alright, you know. They just make – well, 90 per cent - make everything as simple, relaxing and easy as they can. So I'm quite happy with the way things go. I mean, okay, it would be nice…I have been going to the cardiac rehab and they do talks on new medication, you know, but they don't always break it down, it's in groups, ACE inhibitors and…
I: Yes.
R: Yeah, so they talk about it in groups, but it would be nice to know properly what each one does, you know. Yeah, it would be nice, but I won't put pressure on anybody if…I mean, the GPs are run off their feet, aren't they? The pharmacists are no better, you know, hospital under-staffed everywhere. So no, if they want to tell me, I'm sure they will tell me, you know, but it's usually just when there's changes, they just tell me what's changed and always say why, yeah.

P4_C4_C5
R2: Very hard. We got there, and he said, what did he say to you? He didn't take blood, he said there was an infection in there, so he gives him more antibiotics.
I: Right, so when was this, is this since…?
R2: This was Monday just gone.
I: This is since you've come out of hospital?
R2: Yeah, Monday just gone.
I: Yes.

R2: So, he'd had antibiotics from the hospital, he's had antibiotics again, and I asked about all these antibiotics, is it doing him any harm?
I: Right, yeah.
R2: So, the doctor said, well, he said, no, because it's better giving him a cure than waiting while it takes over.
I: Right, okay. So further antibiotics have been prescribed since you've come out of hospital?
R2: Yeah.
R1: Yeah, (I'm) still on them.

P4_C4_C5
R2: Most of it is the same, except for a few isn't it?
R1: Yeah.
I: Yeah.
R2: Most of it's the same, so I can look at this list, what I've got, and it'll tell me what they're called
R1: I think, the old saying is (name) could give them a run for their money. As of, identifying tablets, they haven't come and told us what they are, this, that and the other, and she's had the pack from my old pack, and she's looked at them, and she's said, they've changed that.

P4_C4_C5
I: When you get those conversations about the medicines, in what way are they helpful, how does that help with things, or does it help, knowing [inaudible 00:28:32]?
R2: Yeah, because I want to know what I'm giving him.
I: Right.
R3: My mam knows exactly the tablets and what they're for in that pack, don't you?
R2: Yeah.
R1: That's what I was trying to put over before, [inaudible 00:28:43].
I: I suppose that also, that helps you know when there wasn't one there? Yeah?
R2: Yeah. When I thingy, I'll go through one pack, then the other pack.
I: Right.
R2: And then I know there's one missing.

P4_C4_C5
R3: So we was here, and giving him the packs, I was looking at the, you have the pack, when you used to get it, you had the pack, and it identified or try to identify the tablet, and I noticed on what (name of community pharmacist) had handwritten, it didn't always fill out what shape and style the tablet was. So, in some instances, it was difficult to pick out what that tablet was.
I: Right.
R3: So, where you've got quite a few in the blister, you'd have a white one, or a white oval, and there might be two white oval ones in there, which one is large oval, small…?
R2: [inaudible 00:29:59].
I: Do you…is it useful having the blister pack, do you think?
R2: Oh yeah.
I: Does that help?
R2: Yeah.
I: In what way does that help?
R2: Well, I'd be opening boxes all the time.
I: Yeah, because it's all organised, it's…four time a day isn't it?

Theme 3a. Adaptation of work processes to the availability of information

3a.1 Code - Implementation process - Getting used to the system, learning, training, adapting and changing

HP1
R: The starting up of the actual e-referral?
I: Yeah.
R: I don't know. I know about a year, maybe a year and a half, a while ago, we were told that there was going to be an e-referral project, and myself and someone, (name), who's now left the team, were nominated to be part of it. I think I was probably nominated 'cause I had the experience in community and practice, and it just kind of, nothing happened, and then all of a sudden, the meetings were happening and the discussions. I didn't attend much of the meetings, because it was usually on a Monday when I didn't work, but in our NIPPS team there wasn't much conversation about it, just that something was going on, they'll let us know once we need to know, yeah, so there wasn't much information, until the go-live date when we were told, we will receive notification via email, so look out for these, that was it. But, we were aware of it, we all were aware of it, yeah.
I: But, in some respects, because it's sort of like, you're getting information, but you're not sort of getting the action on [inaudible 00:07:16] referral as they are in community pharmacy, it's slightly different isn't it, yeah?
R: Yeah. I think in hindsight, it might have been good to have, because don't forget I'm one of the leads, and I told my band 7s, that I don't think that many band 7s know, they probably just get an email saying, can you just find out what's going on with this patient? I don't think they actually know the background to it, and there is a system and…so in hindsight, it might have been better to have, maybe, a really short, kind of PPD session, maybe (name) could have done a session on certain cases, like scenarios, how it would work, and how we would be affected, and how we'd be able to help. I think that might have been good to do.
I: In what ways would that have helped then?
R: Because people would have been aware, so if for example, my band 7, assuming they didn't really know much about it, I sent her an email and said that this has been sent to me, and it's in one of your practices that you're working in, can you sort it out? Yes, she'll sort it out, but without knowing the background, really, if she knew the background behind it, she may have learned that, oh, actually, maybe this person in the practice was involved in this, so let me just have a discussion with her, to prevent it in the future. I don't know, just having a background just helps understand why and just helps with the system as well, I think, so…

I: Yeah, precisely.
R: So, yeah, I think that would have probably been better.

HP10.
I: That's really useful, thank you, that's great. When the e-referral was introduced, I mean, there was a period of time, wasn't there, at the back end of last year when you perhaps first heard about it, and perhaps if we start there before... So before it went live and it started kicking out referrals and stuff, when did you first hear about it?
R: I think I might have heard it from my area manager, to be honest with you. He pops in every now and then and we always have a chat about all kinds of things and I think he said something about this being rolled out and he said will I attend training? And then head office must have sent some information about the training that wasn't mandatory, so I don't think I could attend that. And they said they will facilitate some information if we can't attend so we can learn about it and there will be some guidance. So that pretty much was the initial kind of...
I: So you didn't go to any of those training sessions?
R: No, I did not. Funnily enough, I had one case where I liaised with the lady, I can't remember her name, a pharmacist from (NHS Trust) who was nice enough to send me some basic information, and that was quite useful. And again, my head office facilitated some stuff, some info, some basic, like a briefing of what this is all about, and then I just went on PharmOutcomes and worked it out myself, to be honest with you, which is the way of looking at it, but anyway, I wasn't at any training.

HP10.
R: Was it? Okay, fair enough. So maybe it was February that it kicked off. I basically just remember we had a date, we knew from various people, whoever that was. Sorry, I can't remember who communicated it to me but I knew and I had it in my calendar, such and such a date, this thing is kicking off and I need to check PharmOutcomes for some potential referrals coming through. And I already had an understanding of how this thing will work overall, so that's what it was. And then I think pretty much a few days later, five days later, we got a first initial information about someone being inpatients, and then I can look at that, what to do, accept, yeah. You know, I just learnt through that first patient how that works. It was pretty straightforward and I didn't have any... I can't recall any particular issues with the system at all, in terms of understanding what it's all about, in my view.

HP10.
R: To be honest with you, I honestly, genuinely struggle to think about negatives. I'm a positive guy anyway, you see, but there was nothing before and this is there now and it's just great. Maybe if there was a different system or two different systems that we were looking at, maybe I could then compare it and say, oh, this is better than the other, but because there was nothing there, which I've told you already was making our work a nightmare at times and making patients' meds, whatever that event, very unsafe, where now it's just so much better. It's just a structured system in place that there wasn't one. Honestly, I genuinely cannot think of any problem. I've had one case where a lady was... I think it was a mistake, the lady said she was receiving meds from us and I think whatever she said to someone at the hospital, they were under the impression she gets a blister pack, but she didn't. So we got all the information and we told them, I had written down, she is not the recipient of the blister pack, guys, and blahdy-blah. But then they rang me back straightaway: hi, is it (name)? Can I speak to you please? Yeah. They said what it was, they couldn't... And I said, that's fine, guys, thank you very much, and I spoke to the patient, spoke to the patient's husband, saying, listen, we've got this information, but to be honest with you, it's your responsibility. Oh yeah, don't worry, I'm already acting there, the surgery, I know that it's happening. Are you happy to dispense it? Yeah, I said, as normal, don't worry, we will dispense all the meds the way they should be. So, you know, we're quite busy, so I didn't feel I was obliged to actually manage that patient and I left them to it. You know what I mean? I am a big fan of the patient needs to take ownership of their own medicine life itself. Do you see what I mean?

HP11
I: …the start of this service. What did you know about what happened? Can you describe what happened here when it was introduced? How did you know about it and so on, or what…?
R: Well, we went to the original training thing that said it was going to happen. And obviously we…familiar with PharmOutcomes so we thought that's great, we'll…yeah. So…
I: What was that training like?
R: Well, I think we went to the very first one and there were IT issues. But yeah, it was fine. There was a guy from PharmOutcomes there who was…were they on the (name of place)? Yeah, so it was a bit, yeah, disorganised and not loads of people went but I think it was just things on the day. But the actual information was there and there was two pharmacists from…(name), wasn't there. Who was the tall, slim guy there? I can't remember his name. But yeah, no, so it was useful for them to be there and (name), was there who we know anyway.

HP12
I: When it started, was there any training and support that you had?
R: Yeah. So we went to a training evening before it started explaining what the service was going to be and showing us how it was going to work. When we got the invite to the training meeting, we had no idea what it even was. It was just kind of like, well we do venalinks, we'd better go and find out what's happening. And when we went to that we came back thinking, that's a much better idea, it's going to work much better than things do now.
I: Right, okay. And that training did it just explain the system or did explain what you needed to do?
R: It explained the system; they had a mock-up of how the PharmOutcomes would work, so they talked us through that. Obviously a few things have changed in the meantime; it's kind of been been fiddled with a bit to make it better.

HP12
I: What sort of changes have happened then?
R: So, there is now options where we can reject something that's sent to us and give a reason why we've rejected it.
I: Right, okay.
R: Or it is return, I can't remember what the buttons say, there is an accept, but there is one now we can put a reason why we're rejecting it, so it's come to the wrong pharmacy, we need some more information it's not a venalink patient, what have you. So, we've got chance to put a reason for it instead of just accept, reject, kind of thing. So, it does give a bit more two-way communication.
I: Yes, so you are sending some information back to them as well.

R: We don't have to do it very often. A vast majority of them we don't but that option is there. That's one of the things they have fiddled with that's made a difference.
I: When did they do that change?
R: I don't know whether that came in before it even started as a result of the initial training meetings we went to.
I: Right.
R: I can't honestly remember. But when we went to the training meeting and they were showing up the mock-up, it was just, accept, reject. And, obviously, because it's just the venalink patients, you are not getting huge amounts of them, sometimes (name) will deal with them, sometimes I'll deal with them, so you don't always see them all. So, I'm not sure when it actually came in.
I: Can we talk about when it…so you have had that bit of training, so can you describe when it was introduced here, when the first ones started coming through, what happened?
R: I got quite excited when we got the first email, it was like, oh, we've got one. So, to be fair, as I know the first one we got we all huddled round the computer together and it was like let's work out how to do this, how to see it, let's everybody look at it.

HP12
I: So, when you first got that first one, you are all looking at it, then what happened in terms of that first notification – one of your patients is in hospital presumably?
R: I can't remember whether the first one was one of those or a discharge, I can't remember. But we went through together so that everybody saw the process of how to accept it. I think it was an inpatient one and then about two days later they actually came out again so we could all see the process through quite quickly. Because obviously you get people going in and they don't come out for weeks.
I: Yes quite.
R: So, that one was quite a quick one so we could all see and we could show the staff and the pre-registration pharmacist how to view the discharge letter, how to get that information, and what to do with it once we'd got it.
I: And once you'd seen that was that, you know, was it pretty straightforward?
R: It was fairly self-explanatory, I don't think, if we hadn't have gone to the training meeting, I don't think it would have been quite as straightforward to follow, knowing where to look for the information and what have you. I think the inpatient ones are very straightforward. It basically says, this patient has been admitted, accept or whatever.

HP12
I: So, there was a second training event, when was that?
R: That was only a few weeks ago.
I: Right.
R: That was at (name of hospital). Very few people there, very disappointing. There were very few at the first one.
I: Few, in terms of community pharmacists?
R: Yeah. Very poorly attended considering it's quite a big project. So, I don't know why that was. But the first one there weren't many at the first one.
I: Yeah. I suppose [voices overlap 14:05].
R: We got a lot of good information at that second one.
I: Right. Such as?
R: Such as, things like at one point, even if we didn't reject it, we could send a message back. And she said when the service first started she was actively checking every single message back, but as its progressed and obviously there is a lot more, that they don't get checked unless it's a rejection so there is no point writing a little message in, just ring and just clarify it over the phone. Because initially if we had any queries, if we put a message in there you'd get a response back to it, whereas she said, don't do that now 'cause there is that many to go through, it might get missed. Which makes sense. You kind of thing, I'll send a message rather than ring on the phone, but you don't realise there is only one person dealing with all of those, so they won't get dealt with in a timely manner necessarily. Things like they showed us where to – 'cause we've got a discharge at one point and we were trying to work out why there was no medications on it, and then in the end we sussed out that it did say destination, deceased.
I: Right, yes.
R: But it's things like that, it's taught us where to look to get all the relevant bits of information off the discharge summary. Because there is a lot of information on there, and especially for staff, a lot of it's not relevant and it's over the top of the head kind of thing. So, it's knowing where to look to get all the different bits and pieces.

HP12
I: So, in what ways, has it helped, hindered or constrained maybe, the way you work? What challenges have there been?
R: I don't think there is any challenges particularly in the way that we worked. I think the main thing we have is obviously, there's us and there is another branch (name of pharmacy and place) and that is a branch that does basically just blister packs, nothing else. So, we have had quite a few referrals to us that should have been for them. But I mean it's not a big thing because we will just get the referral, it's not our patient, reject, it's not our patient, and then it's done. And then the lady at the training event, the second one, was saying the staff have to hover over a map and if they don't zoom in, they will just pick the nearest thing. But I don't think really it's had any hindrances for us, any negative things, I think it's probably all been positive.
I: Yes. And has it become an integrate part of the way you work at all?
R: Yeah, definitely. And I think, so the more we are getting and the more used to it, and we love the inpatient notification, that's fantastic.
I: Why is that so useful?
R: Because we prepare them all in advance and they are all ready to go out for delivery and normally if you don't find out for however many days, that pack could have gone out for delivery, the delivery drivers might have delivered it, left it with a neighbour, husband, wife and then there is a danger that that is still sat at that patient's house when they come home, with old medication in. Whereas as soon as we get the inpatient notification now, it's like, right remove all of that patient; put them away in a separate area. We leave it all up there until we know that they are back out. So, that's a definite plus for us.

HP12
I: Yes, but you are still empowered with that information to have that…

R: Yeah, because we got the initial information, we spoke to them, sent them the information so he's then rung and said, I've dealt with it all but I've not done this because of…you know.
I: Yeah, precisely. Is there any communication, apart from the little notification you can put in there as to why you are accepting or rejecting, is there any communication back and forward to the hospital?
R: Not an awful lot. We do find if we are ringing up now it's much easier to say, I've had a discharge summary from such a person, and this is…we did have some referrals the other day, so they were discharge referrals, no they weren't they were inpatient referrals, but they were patients we didn't do a blister pack for. So, we sent it back saying, we rejected it, we don't do a blister pack for this patient, and then somebody from the hospital rang us and said, they had been assessed, we feel they need a blister pack that's why we've referred them to you.
I: Okay.
R: And then when I did look at it more closely it did sort of say, referred for new compliance aid. So, it's all still a learning process. We kind of, just looked at the name and went, no we don't do her, reject, you've picked the wrong pharmacy kind of thing.
I: Yeah.
R: So, it's all still learning and the fact that we'd had that phone call, now makes me think, right when we get one that I don't know, I'll check that it doesn't say.

HP13.
R: Well, the first one wasn't very well attended, to be fair. I think there was only about me and two or three other people there. The second was a bit better attended but they're very good events, they were run well, I just thought it was rather disappointing that not many people were…
I: From community pharmacy.
R: Engaging with it, yeah, you know. But I didn't like the faxing that they did before, anyway. I didn't like all these phone calls and faxes. I'm very technological-oriented, so this appealed to me, that's why I went, you know, 'cause it appealed to me. And it's even better than I thought because I like this feedback thing that I've got to do, this thing about going through logically what I've done.
I: A checklist….
R: Yeah, the checklist, yeah and that's there as a formal record, anyway, of what you really did do.

HP13.
I: We were talking about the training event there, you were saying it wasn't very well attended. Do you think people across community pharmacy are appreciating…are engaged with the service or do you think…why do you think that might've been that there wasn't that many people?
R: I think there's a few things there, isn't there, really. Somebody like myself that owns their own business is more likely to go because you want to be up to date with things, right, there's that kind of person or somebody who owns a few shops, they're going to want to go. And then if you're into technology, you're going to want to go.
I: Yeah…..
R: Yeah, whereas if you work for (name of pharmacy) or somewhere, you're less likely to want to go because you're not getting paid more for doing all this extra work. You probably think, I can't really be bothered and a lot of them are half…you know, maybe part-time, pharmacies don't really work very many hours, they're not very technological-orientated anyway, they're not going to want to go, that's what I think it is, myself.

HP15
R: So, I didn't know much about it before until (name), he just explained to me that they'll be sending the referrals electronically. So, when you get the request first, we just have to accept it and then, that gives…that sends a notification to the hospital that we have accepted it. So, they know that actually we are in contact and we know everything that's happening. Yeah, that's all I knew about it beforehand.

HP15
I: So, you just basically…though it's not about, so much as improving communication, it's just they don't need to contact you as much.
R: Yeah. Yeah. No, they don't need to contact us as much. They just check they have received the referrals and things. That was initially because we weren't sure how it was working, but now they don't even need to check. Because they can see all on their end to see if we've accepted or not accepted.

HP16
R: So I think for me it's slightly different because I was involved in it from very, very early on, so we've been scoping this for a couple of years now but the issue was getting the information and the IT bit behind. Now being involving with some of seamless care work we know that (name of place) opted to do the test bed for (name of metropolitan area) , that meant the process had to be quite rigorous because they would need to make sure that it suits any trust's system potentially, it is interoperable with what other people would need from very, very basic paper records all the way up to what (name of place) have, which is an integrated system. So it's that kind of mix so that's where some of the delays came in so I've known about it for quite a while.
I: So as that planning was going forward do you think that was the...what went well with that in the way that it was planned and before? I mean the question here is what went well when the service was introduced but I'm just going to say what went well in the planning to get to that point of introducing?
R: I think it was quite rigorous and we got to a provider which was interoperable, which was the key bit so that the referrals were quite seamless, they became part of everyday workflow, or they could become part of everyday workflow. So what we wanted was something like that if the system in the hospital could handle it. But also if the hospital...

HP16
R: No, that's it exactly, and one of the key things was if that had to be the case that had to be the case but what it was from the hospital perspective was it was like doing anything else. There wasn't much training needed on how to do it or it's not a shock to the system because it's just like generating another order for something else. And that was the beauty of it, so they wanted that level of interoperability where it just became seamless as part of normal practice, the same for pharmacy, we wanted that just the same as normal practice.The key bit for us, I think, was that actually we wanted a system which doesn't have too much of any impact on workflow, it's not onerous, because when it becomes onerous it doesn't get used. But the system has the capability of being used at a very basic level where, say, a hospital doesn't have...you know

we've got varying levels of record across (name of place) completely, to people have separate PAS and EPR system, to people who have half paper, half electronic records even, but the system can work on all of those.

HP16
R: I think that the good thing was it was actually quite seamless and because there's been test bed done it's been tested quite rigorously before roll out it wasn't too much of a shock for it coming through. And a lot of pharmacies were already dealing with referrals so some of it was new, some of it wasn't, but actually dealing with the referral, for us here, dealing with the referral wasn't very difficult because we already receive the referrals from 111 out of hours and things like that, you see, so we already get those things. It literally was just the same as something popping up and just dealing with it basically. And I think we had a few vocal pharmacies as well who were actually instrumental in its development ongoing, so actually these are the issues we're facing, they were very quick to say these are the issues and can we resolve them? And the whole team worked to get resolution. So it wasn't like actually, oh my god, this is a big barrier, it's going to take ages for things to sort out, all done very, very quickly.

HP16
R: So those little things, it was teething issues but actually we put a box in about meds rec very quickly that we recognised that people weren't associating what they were doing with the medicines reconciliation so actually lining up for this repeat they thought that was just normal practice, but actually that is a medicines reconciliation, that's not...you are doing that correctly. So they didn't make the link so actually initially we didn't see very many meds rec being done, we thought, well, actually that can't be right because obviously you'll be checking your discharge against your first prescription. So those sorts of things and there's other things about terminology and clarification.

HP16
R: And I think (name of hospital) have really been quite good at communicating with the pharmacies around if there's any issues, (name of hospital pharmacist) been brilliant at doing that and getting on the phone and saying, actually I've seen something come up which doesn't make sense. And she would always call or email me anyway if she saw something that she didn't understand, so she'd always get on the phone and say, actually I've had a this from the pharmacy, is this normal? So we were working quite closely together to sort stuff out at the very beginning.

HP16
R: There was three for community pharmacy and then whatever it was for the hospital
I: ...the hospital ones, yeah.
R: Yes, so the community pharmacy ones I think people didn't necessarily realise because when we'd asked for the coms we'd asked them to be done drip fed so that the booking links would live for ages and then there's information about the service and then people can realise, oh, actually this can have an impact, and get them onboard. But it got to the point where we had to get people to just ring around the pharmacies to get them to come, to make sure they're aware of it so that they could attend. So we found the last one was very well attended, the other two were medium attendance.

HP17
I: So, it's not much, much later. Were you given any sort of…when you got here was there any training, was there any…were you told anything about it, or…?
R: All I was told was that we get electronic discharge sheets and it comes on PharmOutcomes, and then my supervisor will print it off, but I've now made sure that the main dispenser that does the trays that she knows how to print it off and look at it. I mean we monitor emails multiple times a day. We're on PharmOutcomes multiple times a day. So, we're constantly looking and as soon as the…it comes through, you know, we can see. If you're bored you can even monitor it on home at the weekend if you want to and log in and say, oh, so and so's been in hospital, and then you're prepared for Monday then, you know?

HP2
I: Wasted. Yeah. Yeah. Precisely. When it was introduced, what, sort of, training and support and [voices overlap 05:54]?
R: We had training by two of the pharmacists within the hospital. And so, they were brought in…we were brought into a seminar room and they went through the whole system with us, explained what, you know, was going to happen, what were we to do if we had any problems, to contact one of them too. And, yeah. When it went live…the Monday morning when it went live, we were all prepared, we all knew what to expect. So, yeah, I think it ran quite well, you know. I think EAU were the first people to use it on that morning, so...

HP2
I: Yeah. Oh, that's actually brought us perfectly seamlessly on to our next question, which is about how it was introduced into your workplace. So, what, sort of, things went well, when that went live, 17 February wasn't it? I think.
R: I think it was, yes. Well, it was just so easy to follow. Just the whole process was easy to follow. You know, you clicked on one thing and the next screen came up. So, it felt too easy. Yeah, it felt too easy to be right, but because we did that, we knew the people in the dispensary every evening who were faxing, faxing, faxing, had all that spare time to do something else. So, it took time off people down in the dispensary…a busy time of night, when they are faxing all of these prescriptions. So, they could carry on with something else, help out other staff, so…
I: Yeah. So, it freed up people.
R: It freed up people. Yeah.
I: Who had been, sort of, like, clogged down with this.
R: Yeah. Who were standing at the fax machine, just faxing for however long.

HP2
I: When you started using it, you talked about how you did it and how it's used as part of your work. Was that…how did the ways you used it, fit with your initial expectations, or expectations of what it should be like, or your initial views of it?
R: I thought there would be more involved to it. Rather than just putting a couple of things of information in and then it had gone and it's done, it's that, it's finished.
I: How long does it take per patient?
R: Five seconds maybe. Once you know that the pharmacy is part of the scheme, then five seconds.

HP2

I: Yeah. Stuff like that. Yeah. Yeah. Precisely. And in terms of the way you, you know, using it as part of your work, how often are you sending out those referrals or how often do you go into the system?

R: The e-referral system. Every time a patient who has a blister pack, so, it depends how many we've got on the ward at any one time. So, maybe…I think I saw two patients with blister packs yesterday, but there are other people doing medicines reconciliations, so, then you do…they are probably using it as well. So, I couldn't say day-to-day. It's constant, if, you know…

I: If you've got those patients.

R: If you've got people coming in with a blister pack or a Venalink, then, yeah.

HP2

I: And do you think it should be…is there a potential to extend it beyond just MDS or beyond dosette boxes, beyond Venalinks to discharge more generally.

R: Every patient? I think it would be a really good idea. Yeah. For us it would be a really good idea just to let the chemist know when a particular patient is in. But for the chemists it will be…basically we are passing our work on to them. And I've had this discussion with a particular pharmacy here in (place), who said to me, I don't like this system at all. So, I said, why? It's great, do you know, she said, yes, but you've passed your duties on to us. Because now they have to go to the computer every morning to look, to see if they've got an e-referral…a referral and it will say, oh, we're coming to have a look to see who's in hospital. Oh, right, sort all their…what we're going to do there…blister packs today, no, so should we do that. She said, but then all through the day, we are constantly looking. So, it's taking time out for our staff to keep looking to see if there's been any referral to say somebody is in. So, we don't have to do the blister pack. Whereas beforehand, we were waiting, until the end of the day to fax them, they got hard copies, so, they didn't have to keep going looking at the computer. So, they were relying on the paper appearing on a fax, now they have to keep going to look at the computer, to logon, to put their code in or their password. So, it's causing them more time.

HP2

I: Yeah. Precisely. If you were to…well firstly do you think…is there…have you discussed amongst the team, how the referrals work? Have you had any…I mean, you had that bit of training before, have you then…?

R: Yeah. But we've not had any meetings.

I: There's not been any.

R: Not that I have attended.

I: But there's been informal discussions.

R: Yeah. There's been informal discussions, twice a week we have a departmental meeting, so, just before work starts on a Monday and a Friday. And (name) who has run this will say different things, you know, just make sure that you're putting the right postcode in, make sure that you're going to the right chemist. If you can't get through to anyone in particular, if it's not on the map, let me know. Things like that. So, that's…in those meetings, (name) …

I: Yes. So, dealing with those little glitches around that.

R: The little glitches, (name) will pass on, yeah.

HP3

I: What sort of training and support did you get when the service...? Because are we now? We're two months in, aren't we, from when it started, or before it started?

R: Yeah. I think we had several sessions. I think there were two or three sessions maybe where (name) went over what was going to happen and what it actually looks like. I think, for me, I'm the type of person that actually needs to see it myself to actually understand. So when it did go live, I still didn't really understand what was going on, but I'm the type of person that actually needs to see it myself and play around with it to fully understand. But she did do several training sessions.

I: So you had some support to do it. But you say you particularly wanted to actually get in there and see it hands-on before actually...

R: Yeah, just because seeing it in theory, like seeing just pictures of it, I'm not...and especially because it was quite a few months before it actually went live, so...

I: Yeah, because that training was, what, back in, before...?

R: It was last year, I think.

I: Before Christmas, wasn't it?

R: Yeah.

I: Yes.

R: And so I was thinking, I'm not going to remember any of this information. So I think having those sessions, maybe like the week before even it went live, that would have been helpful.

I: Yeah, I think they wanted to go live sooner than they did.

R: But I mean, still, she put through the training sessions which I thought were good.

HP3

R: So I think it took all of us a bit of time to fully understand what we're doing now. I think it started on a Monday, and so we did have our team huddle and (name) did explain again, 'guys, it's going live, make sure you write it down as the electronic referral.' So before, when we'd send down the blister packs and we'd have the fax sheet in front of it, we all knew that it was a blister pack, but now, (name) said there's no need for the fax sheet anymore, just write the letter e on top of it, saying that it's Venalink, and so that should be fine by itself. And with that she said you don't even have to put what goes in which slot, because usually, when we do the TTOs, we'd write on the top that it's a Venalink.

I: TTOs are the...?

R: The discharge medication.

I: Medication, yeah.

R: Yeah. And so on that we would write, this is a Venalink blister pack, so when they're dispensing it they know that...

I: They know how to put it and make it up.

R: Make it up. But (name), when we first started it, she said, oh, that's not needed anymore, because if you put the letter e, they should just know that this is an e-referral, so they should know what should go in the blister pack. But I think there was confusion, because there were some that went through and they were just putting them in boxes, in separate boxes, instead of actually putting them in the Venalink. So, I think there

were some discharge meds that went through that weren't blister packs, which I think it just confused the dispensary staff, but then I don't know if they'd had...

HP3
R: Were a bit confused by what to know, what to put inside of the blister pack, or even if it was a blister pack. And so I think we had a meeting, it was like the next huddle, we just said, we'll just continue writing that it is a blister pack just to make everyone aware that it is.
I: Yeah, so that they were aware. So could that have been done better then, that introduction?
R: Slightly. I don't think it was terrible, though, but I think it could have been done slightly better, but I think for what it was, it was fine. I think we were just a bit slow getting used to using...like finding pharmacies... I think that was another problem, actually, doing the community pharmacy search. I think some people struggled finding where the pharmacies were, just because it shows up with the actual google maps, and so you have to pick which pharmacy it is and search it. I think you can either search by pharmacy name or the actual location, but with pharmacy name, because there are so many (name of pharmacy), it's like how are you meant to find which (name of pharmacy)?

HP3
R: I didn't really have any expectations, to be honest. I think because I didn't really fully understand... I didn't understand what was going to happen. All I knew was...because (name) said it will just make things a lot more efficient. I remember at one of the meetings that I went to some of the other pharmacists weren't really happy about the whole e-referral situation. I think for them... But then I think it was mainly the senior pharmacists that weren't happy about it. I'm not sure if it's because they're so used to the system that we had before, but I know one of them said it's because the pharmacies will now be aware of why the patient's in and what's happened and it's too much information. But I feel like the more information the better, to be honest.
I: Why do you think that?
R: I feel like it's just better for them to understand why this patient's been in. If they're left in the dark, it will just be... I don't know what the word is. I think them having more information will help them with their work as much as it helps with us knowing fully what's happening in the community for the patient. So it will help them understand, okay, this patient obviously hasn't been adherent with their medication and maybe we can do something better to help them be more compliant, I guess. Because, say, it's a case of their blood sugars are ridiculously high because they haven't been taking any of their medication, if the pharmacy's not aware of that they're just going to keep doing the same thing because they think nothing's wrong.

HP3
I: So have there been any hindrances or constraints to your work? Have there been any challenges and difficulties with the service?
R: I'd say probably when we first started using it, but then that was because we were just still learning how to use it, so it felt like, oh, this is just taking so much longer and why can't we just fax it? But now that we understand how it works and how to use it, it's fine. I can't really think of a bad thing about it, to be honest.

HP4
R: …so she did a lot of kind of…she came up to the ward because obviously because we're in the admissions team, we do an awful lot of blister packs and things, so we probably use it the most, so she came up to speak to me and my colleagues on the ward to run through how it worked and talk us through, and we got an email with all the instructions with how it works, launch date, all that kind of thing. And then she's been really proactive at getting feedback on issues we've had in the rolling out process, so we've spoken to her quite a few times just to query things or check how we should be doing things or… But yeah, I think it was rolled out really well actually…
I: Yeah, I mean, that's…
R: …I think basically, I mean, the thing is, it's fairly straightforward. There was lots to read through when we got the initial documentation, but actually in reality it's a very straightforward process.
I: So in terms of that introduction, was there anything that could have been done better you think or was that…?
R: I don't think so, I think it was pretty well rolled out to be honest, yeah. I think it was self-explanatory, I don't think we needed kind of talking through it necessarily.

HP4
I: And how has it fitted in then with the way you work?
R: Really well, yeah. The only…and kind of the very first day where we used it, we weren't too sure how to use the search function properly, we finally figured out that the easiest way to search was by postcode, it's just kind of the way it works, so we tend to use that. So it works really well I think, we've all taken to it quite quickly.
I: And is there anything that's hindered or constrained your work, made it difficult at all or any challenges?
R: Only sometimes, like if there's a couple of community pharmacies really close together, sometimes we've had difficulty trying to identify which or pick…it's easy to pick the wrong flag sometimes if they're really close together. But overall, not really, once I did have an error message, a couple of times I think on our ward we've had an error message pop up which meant that we couldn't do the e-referral, which I did speak to her about, just to kind of try and find out what that error was. She did want us to screenshot it for her next time it came up. I've not had it for a while to be honest, I can't 'even remember what she said she thought the reason was. So once I couldn't do the e-referral and I had to leave it as a kind of outstanding flag, and I just rang the community pharmacy instead.

HP5
I: Yes, so when the e-referrals started here…well perhaps we'll move on actually because the next bit, and I'll come back to that part…the next bit is about how it was introduced here. Can you remember those first occasions when [voices overlap 0.07.55] went through?
R: Yes, we had it as an email because I'm off site. We had it, I had it as an email that was going to start an e-referral system. Then I had to read what the process, what was involved in the process of sending email, of sending an e-referral to a patient, what this involved with regards to this, and to come to the hospital if I was uncertain as to whether or not I knew how to do it and so on. So, I met up with one of the pharmacists here, the pharmacist who is heading it and I had a little chat with her about, you know, in what circumstances do I use this et cetera. And to be honest the minute I read about the e-referral I was, like, I want to do it now, if that makes sense. Because of the circumstances under which I work, obviously because I'm off site I've had a lot of challenges with chemists phoning days later to say we didn't know this patient was discharged for example. Even though I would say that I've sent the e-referral, I've not seen the fax, or maybe they sent me say several faxes that day and it was probably not the information that they have received and it's been chucked away. You know, so it's the minute I saw e-referral I thought, oh

thank goodness for that. And the other thing, of course, in my head that was going on was that when it was explained that once you put an e-referral on at the point of discharge you get a discharge summary sent to the GP, but you refer, the minute you refer it is sent to the chemist. I thought thank goodness for that.

HP5

R: It shows a list of drugs and there's a cut off line. And then below that we've got [inaudible 0.15.11] and then community pharmacy referral. So, it tells me, once a community pharmacy, because there is an order… so once that order is made, I know that community pharmacy referral has been sent.

I: Right, yes.

R: And all it's waiting for is for the discharge now.

I: And then it will automatically…

R: And it will automatically go to the community pharmacy.

HP5

I: So, but it's moving from here to community pharmacy, is there any transfer of information back from the community pharmacy to yourselves, or to here?

R: No, no.

I: Do you think there should be?

R: It would be helpful. I suppose the community pharmacist will deal with us on a need to know basis. I mean at the end of the day if you want information from the community pharmacy, we will have to initiate it, you know. So, it would be great if the community pharmacy could let us know, have a complete history of what a patient has had from them, but it's never necessarily complete because patients are not necessarily loyal to community pharmacy these days either. So, the best source of history of medication will be via the GP surgery, where we already have access to the SCR anyway as to what has been dispensed, and we can just confirm that information with the community pharmacy. Having said that telephone systems are prone to being busy, a lot of people are using telephones to contact pharmacy et cetera. Whereas, if you can get an electronic confirmation of information safely that you need, then that could save time.

I: Do you know if it's been received and when you, when you send that through do you know that community pharmacist has gone oh yes, that's my patient.

R: Yes, because I phone them.

I: Oh right.

R: What I did, I'm not one for using a system and then assuming that it's worked perfectly.

HP5

R: Yes, I know, I know. I had to, for my own peace of mind the first, in fact the first at least a dozen e-referrals that are sent I actually phoned to make sure that they received them and what they've received as well in a pharmacy outcome. And there's another, there's a community pharmacy that I deal with that's actually next door to the facility where I work and I've actually walked, gone into there and I've said have you received. Oh yes, yes, yes, we have, we have. So, I, sort of, like, I've made sure. I've needed to be satisfied that it's working as I said it would. And I've had 100 per cent hit, hit with regards to the telephone call follow up of whether or not…So, I'm quite happy that it works as it said it would.

HP5

I: So, that, in so much as now they know that, has that changed what you do in terms of, you know…you talked about not doing faxes and stuff…has it changed things, how you then, you know, the ways you operate, or is it just the…?

R: No, no, I keep the way, I still stick to the way I operate, I only add those additional questions with regards to the e-referral.

I: Yes, so you are only doing…?

R: I'm very mindful of it, but the basic job is still there.

HP6

I: Right, that's really interesting, so basically if when the e-referral goes, the pharmacist rejects it, what do you then do?

R1: What I do, every day, as often as I can, morning time, before I start doing other things, I have a look on the PharmOutcomes, I log-in, see if there's an e-referrals, at nine or quarter past nine, at that time, probably there is none but later on, they start appearing on the system, on PharmOutcomes. So, when I see one of these rejections…do you want the details of what I do?

I: Yes, go on, why not.

R1: So they prompt out, there's a list for all the e-referrals that are pending on PharmOutcomes but the ones that are rejected, they go on the top of the list. So it could be, one, two, three, I've seen three the other day. So I go on each one individually, so I press the one that I need to see what's the reason behind the rejection and try to deal with it appropriately, accordingly, find out the reason. There's like a record as well, so I also try to sort out the problem, which is the most important thing, we need to sort it out, we need to get it sent to the right place.

I: Right, because sometimes it will be that it's gone to the wrong pharmacy?

R1: Exactly, well yes most of the time, I think, so far, it was directed to a wrong pharmacy. There are many reasons, if you want me to mention them now.

I: Yes.

R1: So let's start in order, so once I open that rejected Venalink prescription, I find out the reason, I write it on a log and that log goes to (name), the pharmacist whose in charge, to follow up and change something on the EPR, which I'm not allowed to, I'm not authorised to. Once I write it on that log, I try to follow the steps, if the patient is not with that pharmacy, he doesn't deal with the pharmacy, he or she, then I try to find out what the right pharmacy is. So sometimes, what happens, they pick up, on the system, the wrong pharmacy, so because the map, it's not very, very clear and especially under work pressure, people tend to, when they see the area and see that sign of a pharmacy, they click on it, while there is one very close to it, carrying the same name but in a different lot and then mistakes happen. So I try to just make sure, go on EPR, check the right address, check the right telephone number, try to speak to them if there's any need for that and this is one of the easiest things to do, actually. So I just click on the right pharmacy and say that it's done. Well if it's the wrong pharmacy on the EPR, then I have to report that to (name) or the person who is dealing with the EPR thing, who have authorisation to go and change things over EPR. As I mentioned, I don't have that authorisation.

HP6
Then when the time approached, to get it implemented, to get it in place, we went through a small seminar with (name) and (name). So they explained the whole system and how it worked, which pharmacies are going to be covered by this system. For an hour we talked about the advantages and the potential disadvantages but so far, I haven't seen any disadvantages. Well the disadvantages could be due to human mistakes, not the system, not the idea itself. The idea and the system is brilliant but there's nothing perfect because there is humans involved in that. When a pharmacist, for example, choose the wrong pharmacy or when a pharmacists forgot to do the e-referral, because they don't do it for all the discharge because they are discharged without Venalink, they don't need that.The discharge with Venalink, so sometimes they got distracted under work pressure and they forget to put the e-referral on the system. So then something happens and we get involved to sort it out.

HP6 Yes and read everything and she explained how we do the e-referrals. I have spent hours with (name) talking about how the system works and because there are lots of bits of information that aren't important, it's not like I'm sat in front of the computer and see them. We need to understand what to do and we have to make it as a workshop and try and sort out some rejections and learn…I learnt from them, (name) let me learn from these experiences and day by day, I am getting more knowledge about it, how to do it.

HP6
R1: Yes and I need to know, sometimes, it's like a piece of a whole picture, a bigger picture, if you need to understand that piece, you have to know what's around that piece, you have to see the whole picture, sometimes to understand how the work goes.

HP7
I: Yeah. When this was introduced, what happened, can you just talk me through, if we go back to when you first heard that there was going to be an e referral system.
R: They have a few…they gave us sessions about it. So, like what pharmacies it would include, how it would work, and it was more like an information, we could ask questions and it was with the medicine safety and the EPR pharmacist. So, the man who is in charge of the prescribing electronically basically.
I: Yeah.
R: There was quite a few sessions so if you couldn't attend one you could attend another one. And then there was a lot of talk about it and the day it was, sort of, released we were told about it and then if we had any questions we could speak to somebody, if we had any problems.
I: And, was that training and support, was that useful?
R: Yeah, it was useful.
I: In what ways?
R: Just knowing how it was going to work. So, the map and knowing that it's not going to include all pharmacies and that if it's not on the map you can't send it. That sort of thing. So, yeah, I thought it was really useful.

HP8
I: You said earlier actually about your team, did you give your team any training around e-referral?
R: No. So they were invited to come to the community pharmacy, the sessions that were put on for...
I: The sessions that were put on by…yes…
R: ...community pharmacy, but I don't think any of our NIPPS team went. I think one of our NIPPS team might have gone to one of those. And then there was also one at the hospital, which I think two of our NIPPS team attended aside from me.

HP9
I: Are you seeing many of them?
R: So far I've only been highlighted one that (name) has sent over to me, but the pharmacy had already rang the GP practice the day before. I think because of that system, they'd rang the practice the day before and let me know that they'd got a problem with this discharge, so I had a look at it. So when (name) sent me over this, I was like, oh, okay, yeah, I've had a look at it already, so they must have come across it because of that system and then flagged it up the next day and then…but I'd already sorted it 'cause [inaudible 03:47]…
I: Yeah, it's interesting, isn't it, because possibly then, because of the service, pharmacists are actually triggered to do something about it earlier. And though when you're getting it it's a bit later, yeah.
R: Yeah, so because (name) I think gets a lot of requests so it's basically she'll send them over but by the time she's sent them over it's probably…it's been done or by the time it flags up maybe on the system, it's already been dealt with. But this is so far what I've come across, that it, yeah, they've been flagging it I would say more often, 'cause usually I would do the discharge and then I would call the chemist but I think there's been quite a few instances where they've called me about a discharge and they've said, well, we've got this discharge from the hospital and the medications don't match, so can you have a look at it? Which is really good that they're being a bit proactive about it because…

HP9
I: Yes, we'll come onto that actually because having that proximity of them is quite interesting, isn't it? When the e-referral service started, how were you told about it? How was it introduced? What did you know about it to start with?
R: I think there was emails from (name) and from maybe (name) in the past that they've just told us about it. I think it might have been discussed in meetings, just briefly what it's about. That it's basically to try and improve the three-way communication because at a certain point I think the chemist will be able to refer to the GP other problems, not just…maybe that's my idea of it, that there'll be three-way communication or I can refer to the chemist through that service. I don't know if that's…this is like, future. I know for now I think it's to communicate with the chemist that the patient's been discharged and give them the discharge summary. And then they will obviously modify it on the system. I'm not really seeing it in my own eyes to say…I've seen the table, the spreadsheet and I know they can…if it's pending or if it's been received, if it's been dealt with and things like that. So I think that's for the time being but I think they've got more plans for it in the future as far as I have heard. But this is just like the trial, isn't it? There to see how it's going.

HP9_
I: …yes, or on the wrong ones. When it was introduced, there was some training, wasn't there, at the hospital? Did you go to any of the training?

R:	I didn't go to the training, no because…I can't remember why, I think I was either off…I think I was probably off at that time or I can't remember if I…yeah, there was a reason why I didn't go, I'm pretty sure it's because I was off that I didn't go to the training, but yeah, I did plan to go but I couldn't go that time. There was definitely a reason.
I:	I mean, I know other people did go to that. Do you think that it would have been helpful for the NIPPS people to have seen what was going on?
R:	Yeah. I think it would have been helpful, yeah, 'cause when I got the referrals and I looked at this big spreadsheet, I was like, I don't really know what's all these fields, there's loads of them and I was like, does that mean it's been done? 'Cause it said…I think on the status bit, said something like, that's been completed, but then (name) was like, no, I think that means that the chemists completed their side of it. I was like, oh, that's really confusing, 'cause I was like…and that makes it look like that has been done and sorted but…
I:	Yeah, 'cause you're saying it was a spreadsheet…
R:	Yeah.
I:	…whereas…I mean, they're seeing it through PharmaOutcomes which is completely different.

HP9_
I:	They need the new one from the pharmacy. Has there only been any sort of – in this last part really – wanted to look around your work, which we've touched a lot on and we've talked a lot about, but has there been any sort of impact in…has it helped? Has it hindered your work? Has it changed your work at all?
R:	No, I mean, it's not hindered my…the e-referral we're talking about?
I:	Yeah.
R:	It's not hindered my work at all. I think it helped. It's definitely helped because like I say, more things are getting flagged up and it makes me look…I think a few of them, I'd not even received a discharge and it did come through the system itself but I think because the discharge went to the chemist they flagged things up for me. And I managed to sort it in time.

HP9_
R:	…that's what's been happening, yeah, it's really useful because they want to make sure the patient gets the medication on time as well and want to sort out their blister pack early. So they seem to be flagging it up. When they come across any problems they just flag it up to us and we can sort it. I guess again, that relies on the people who are getting it, how pro-active they are. But yeah, I would say it's definitely not hindered my work. It's helped more than anything to facilitate that process and improve it, yeah.

3a. 2 Code - Previous system and work processes

HP1
I:	What was there before this, in terms of any communication?
R:	Nothing. A telephone, yeah. So, I know, 'cause I've worked in community, and obviously I'm working in practice now. I've never worked in hospital. But I know that, when I was in community, we would get the discharge faxed from the practice, and working in practice, I mean, it's varied depending on what practice you work at, for example, (name of practice), we're quite active, we're always picking up the phone, we know who the pharmacy is on the other end, they know who we are, and there's plenty of exchange, documents and things.
I:	Yes, but that's fairly informal exchange presumably?
R:	Informal yeah. No formal software or anything like that. There's nothing, yeah. So it was give and miss. So, for example, if I wasn't here, or the practice pharmacist, wasn't here, there's no guarantee that that would continue, so definitely, there was nothing formal, yeah.

HP10.
I:	What was there before? What did they used to do?
R:	They used to fax things over to us but that wasn't always working. So it used to be, I don't know, it was a bit wild, to be fair. It was either nothing before. I've been working in (name of place) for over four years now, in this one setting, yeah, pretty much four years. So, initially, there was either no communication, we used to just, by accident or find out or receive the random prescription with a substantial amount of changes to this blister pack patient, surprise, what the heck is going on? Ring the doctor, blah de blah, blah de blah, changes, speak to the hospital, this, that and the other. It was sometimes like this. It was gradually improving, so the hospital wasn't letting us know about the patient being admitted, but we used to get information about the patient being discharged. We used to be told they are given a week's worth of meds, they will run out on a given day, we fax over the discharge. So that's been implemented sort of three years ago, to my understanding, to my kind of experience. And that was happening but not always in such a great way. So I used to try then to communicate that with the next door doctors, letting them know I'm waiting for those scripts to come. So that was the way, yeah, so that was the way.

HP10.
I:	So they faxed it but then there was also some informal communication then between you and the GPs?
R:	Yes, it was difficult. I think ever since the GP surgeries took on, they developed... Like the surgery next door to me, they developed this, what they call prescribing team. They will have a pharmacist or two pharmacists in charge of running it, they will have prescription clerks, all these people are doing all activities with regards to prescribing prescriptions, discharges, you name it, all the safety of meds. Where before, it was really... The next door doctors, unfortunately, it was all over the place, in my view. There was usually a doctor leading this, signing, but there was no one really taking full charge of every single person was issuing. It was utter chaos. Again, I'm talking about this particular surgery next door to my practice that we got 90 per cent of prescriptions from, so it wasn't very professional, it wasn't very sustainable, it was just chaos. So ever since that has been introduced, they very much...you know. We used to engage with whoever was answering the phone in the past. Now there are people that we can relate to, exactly. We can either ask for (name) or this that or the other. So this is the way now. In the past, it was just...

HP10.
I:	Yeah, that's the sense of the journey through, because that's really interesting, because you sort of like go from the note, putting it on hold, through to the checking of the discharge summary. But what I'm really interested in there is that communication with the GP. Is that something that's new or has that always been like that? Or is that the sort of because you've now got this extra information coming with the discharge summary through the e-referral system?
R:	I think it's a number of things. I definitely think this discharge has improved it enormously. We've got this meaningful... It's kind of... You know, on a discharge sent through the fax, you could sometimes hardly see what's on it. It was hand-written very often, it was just things coming

through the fax. There was, like, ink covering certain bits, the information wasn't very good. If you had to pass that on to someone else, fax it again, or scan it and send it via the email, it wasn't very clear, where this is I've got a PDF, so I can easily just save it on my PC and send it as an email to my doctors, which I do, but they prefer the fax. For some reason they prefer that, they pick it up quicker than an email, but I'm talking about next door. But, overall, this has improved it. The other thing I would say is the fact that I've been there for a bit, I know all the main doctors, I know everybody there, I'm quite a proactive guy, I engage a lot with them. I used to attend more often the meetings, so we know each other well, so they don't fob me off, they respect me, they know I'm not wasting their time, they know there is some meaning there. I think we've got...

HP10.

I: Has it changed the way you do things then, the e-referral service?

R: Yes, it did. I mean, again, always when someone was going into hospital, coming out of hospital, it was always a problem. Believe me, it was always, I felt, Christ, a crisis issue, there was always that kind of label to stick to that kind of event. Someone who was a recipient of a blister pack was going to hospital, we never even knew about it, anecdotally we sometimes found out through the family who was conscious enough to let us know about it. Honestly, there was... So it was always an issue...

I: So often, beforehand, you wouldn't have stopped...?

R: No, that's what I mean, that's the thing. I'm telling you the truth, that's what it was. So sometimes things were being despatched to go out to the patient but they didn't have a clue. Sometimes our drivers will have an access to a key to someone's house and they will just open the door themselves, leave it in the porch or leave it somewhere, the parcel, because we know the patient is in a wheelchair, is upstairs and can't come down, those arrangements are made with carers. And then stuff was there and no one really knew anything. Where now, as I said to you, we will straight away look, oh, it's sitting on a shelf, [inaudible 0:21:30] it's with the patient, fair enough, okay, we'll see what happens, we will make sure that comes back to us for disposal if there are some changes.

HP11

I: What happened before we had the e-referral, 'cause…well, we had…? Yeah.

R: Well, we used to get a fax from the hospital. So we didn't know the patients had…unless the family let us know they were in hospital, we often didn't know they were in hospital 'til we got a fax or a phone call to say we're sending them home.

I: Yes, so on admission you got nothing?

R: We had…no, we had nothing, unless we just…or we would send a pack out and it'd come back and the driver would say, oh, the next door neighbour says they're in hospital.

HP11

I: That's great. That's brilliant. So in terms of work, has it made any differences to how you work? Has it changed things, hindered things, made it more complicated? Made it easier or made it better, or what?

R: I think it's changed massively. I think it's probably helped because we have all the information. But the actual process isn't much different because we were still getting the discharges from the hospital.

I: Yes, but just by fax.

R: Just by fax.

I: Yeah. So the processes…

R: We're still getting the same information now, just more reliably and immediately I think as well because I know (name), was saying as soon as that's generated at the hospital, that gets sent to us.

I: Yeah, I think it's two hours.

R: Yeah. Whereas before, you know, it might have been a day or so, mightn't it?

HP11

I: Have there been any difficulties or challenges involved in it?

R: No, I don't think so. Apart from the odd one that we've got that isn't ours. But no. I mean, that would have happened before they were just…that would have…in the past we've had stuff faxed which isn't our patient, it's for MDS or whatever, so at least it's secure and you're not having to think, this shouldn't have come to us.

I: So on a…where it's not your patient, you just send it back and then it's gone to the…it's been redirected to…

R: Yeah, we then just click on a thing that says, refused, or…

I: I suppose with now, you can highlight that as soon as you get it?

R: Yeah, whereas before we would have probably phoned MDS and said, is this your patient? And they would have said, yeah, after five minutes, when they'd looked. And then we would have then faxed it onto them really. Rather than probably…and if it was nothing to do with (name of pharmacy chain), we would probably then phone the hospital and tell them. So that's an improvement, isn't it, from the confidentiality point of view.

HP12

I: Yeah. What happened before?

R: So, sometimes we would get a copy of the discharge summary faxed through to us. It tended to just be the medicines page, so there was none of the history, why they were admitted, stopped and start, it was just basically the list of the medications.

I: The new medications that they are on?

R: Yeah, the discharged medications. Sometimes we wouldn't get anything, we would just get the patient ringing up saying, I've come home, I've got one day left, when am I getting my next ones. So, then we'd go to the surgery and say, we believe this patient's been discharged, have you got a discharge summary, they then would have to find a discharge summary, get scripts sorted out, get everything done and it was sort of a very urgent situation really rather than now, it all seems much more controlled and much more…

I: Right. So, there wasn't time to do things before you say …?

R: Sometimes there was. It depended on, sometimes the patient would ring us and say I've just come out of hospital today, in which case we knew, we were looking out for it. Sometimes the pharmacy at the hospital would ring and say, we are discharging this patient, we will fax you over the discharge summary. It was just a bit of a mishmash of everything. There was on structure to it really.

I: Yeah. And if you got discharge summaries, they were faxed to you then?

R: Yes, they were faxed over to us.

HP12

I: So, in terms of how good that was, what were the issues in that then?

R: It was mainly, a couple of things really, the fact that there was no sort of continuity to it. So, it wasn't the same, you couldn't rely on getting a discharge summary faxed, you couldn't rely on somebody ringing you or what have you. The fact, like I mentioned before, that sometimes you wouldn't know about it until the patient had no medication left, which then means everything, is being done super quick, which I think gives more room for errors to happen and that kind of thing.

HP12

R: Such as, things like at one point, even if we didn't reject it, we could send a message back. And she said when the service first started she was actively checking every single message back, but as its progressed and obviously there is a lot more, that they don't get checked unless it's a rejection so there is no point writing a little message in, just ring and just clarify it over the phone. Because initially if we had any queries, if we put a message in there you'd get a response back to it, whereas she said, don't do that now 'cause there is that many to go through, it might get missed. Which makes sense. You kind of thing, I'll send a message rather than ring on the phone, but you don't realise there is only one person dealing with all of those, so they won't get dealt with in a timely manner necessarily. Things like they showed us where to – 'cause we've got a discharge at one point and we were trying to work out why there was no medications on it, and then in the end we sussed out that it did say destination, deceased.

I: Right, yes.

R: But it's things like that, it's taught us where to look to get all the relevant bits of information off the discharge summary. Because there is a lot of information on there, and especially for staff, a lot of it's not relevant and it's over the top of the head kind of thing. So, it's knowing where to look to get all the different bits and pieces.

I: And also knowing that you are getting notification to know that the patient has passed away earlier than you would have done.

R: Yeah.

I: Because before that you wouldn't have heard anything.

R: We wouldn't have heard anything, no. It would only be like every so often we'd think like I've got all these up in hospital, let's check with the surgery whether they are still there, have they gone in nursing homes, have they died.

I: Yeah. So, you would also get on that discharge summary, you would know if they have gone into intermediary care.

R: Yes. So, on that destination bit it would say where they've gone to. So, it would either say, home address or intermediate care or wherever.

HP13

I: Yes, right. What happened on discharge of…'cause we've got, obviously we're talking about patients on blister packs or venalinks aren't we? What happened on discharge before?

R: Well, they would simply do it by phone, you know. Well, for a start, we didn't know when they went into hospital at all. Sometimes we did if we had the phones but usually not. And that was a problem because then we'd be getting a Venalink ready for them, not knowing they were in hospital, where at least now we know. Then they used to phone us on discharge or a couple of days before on the discharge, they used to phone us and then they used to fax through the discharge and that was kind of about it. Well, I suppose that's similar but that was about it. Sometimes the fax, you didn't get it, occasionally it didn't happen.

HP15

I: Yeah. Yeah. And you said you are getting a notification on admission and you get the discharge summary…the full discharge summary now, when they leave hospital. What was happening before the service was put in place?

R: So, usually the patient's carer or someone, whoever is with the patient will let us know. Or most likely, when the hospital, the pharmacist or someone, when they go through the medication, they'll call us to let us know. But it doesn't always happen. So, sometimes we don't get informed here.

HP15

I: Yeah. So, you were getting informal conversations from people to let people know, but not before. And you said that the discharge was handwritten beforehand and it wasn't electronic. What information did you get from that before?

R: It's just the medication. So, what they were discharged with. What medication they were discharged with. So, you usually used to get faxes. So, they call us first to say that, what's your fax number? And they'll fax over the discharge letter. But sometimes when they do that, we still don't get the discharge letter until days after. And then the patient…at that point because it's not straight, it's not the same day they are discharged. It is probably two days after they've been discharged. So, there's a delay.

I: Yeah, they are already in the community.

R: Yeah. And then what happens the patient will need the medication. They usually get discharged with seven day's supply. So, if you have not received it by the fourth day…I mean when the doctors haven't received the discharge at that point, there's a big delay because they don't know, they can't do the prescriptions until they've seen the new discharge letter and everything.

I: And then you've only got three days left before.

R: Yeah. So, what happens we keep…we are continuously chasing up…can we get the prescription for…when they haven't got the discharge either. So, we usually get the discharge on the fifth day. So, it just leaves two days for them to look at everything, arrange a prescription. And the patient is getting very worried and saying, well I don't have medicine, I don't have medicine. Even though they have two days left but they're scared. So, they keep chasing us, that can I have the medicines?

HP16

R: For me it's about embedding pharmacy in the discharge pathway, so historically we had a lot of issues with this about patients and not getting faxes on time, not receiving faxes at all, not knowing patients were in hospital, and that causes us massive delays. There's a bit impact as well so if we deliver something that's possibly incorrect the patient comes out they may think it's correct and just take it again, so there's a risk of a readmission from there. The other part of it is waste of resources because that's waste medicines plus also our driver time, pharmacy time that goes into preparing for that patient, but also it's inaccurate information. So what the e-referral aim in my head is, is that actually it gives us a copy of the discharge at the same time as the GP which allows us to plan for discharge. But before that it's let us know that the patient's in hospital and to just cease dispensing for that patient until we hear otherwise.

HP16

I: Yeah, absolutely. What happened before the e-referral service [voices overlap 03:53].

R: So before if a patient was admitted we should have got a phone call to say they're admitted and then we'd go through all the medicines and the rigmarole that was there. And then on discharge we may get a fax, we may not and we may get a phone call. So the aim was, the aim before was everyone a phone call that the patient's in, the blister pack, and then once they're discharged a copy of the discharge comes through and we're phoned that they've gone home with X number of days medicines. Now that information was sporadic, it wasn't always communicated through, the issues that we had with faxes was if you've got a five, six page discharge you may get page three, page three might not be visible, you may get half of it and the fax gets stuck and nobody's recognised that, all those things, legibility, transcription, all those issues that came in. So at the moment now with the e-referral service we get a copy of the discharge as is as a PDF.

HP17

R: …in the past…I mean I've been a pharmacist for quite a few years, 15/16 years; previously you might get a discharge sheet, you might not get a discharge sheet. There's a lack of information, clarity from…There's a lack of, or there's a loss, or you don't get any data. So, I've seen so many problems. So, here's the hospital, patient comes out of hospital, they then turn up at the chemist and say, where's my tray, or, I've got no medicines, and you don't even know they've come out of hospital, you don't know anything, you don't know if their medicines have changed, if they've not changed, have the hospital discharged them with a week, or not? So…and then it causes massive problems because when you get to the stage when they actually turn up in the chemist and they've got nothing then you need to sort that very quickly and you can't always do that and you can't give stuff out without a prescription, unless you do an emergency supply, and then you've got to bend some rules, whether you want to do that or don't do that, and… But I mean you've got summary care records, which is better now, but from past experience this new system of getting electronic referral is brilliant because you know instantly…you know when they go in hospital, you get an email for that and you can bring it up on PharmOutcomes, and you know when they come out, and you get…all the information that the doctors get you get, I'm assuming the same, or very similar, information.

HP17

And before this…this was…this chemist, I've been here since 8 May and it's the first time that I've seen this electronic discharge sheet through PharmOutcomes. So, before, if I go back to when I used to work in the area of (name of place) way, dealing with the surgeries in (name of place), or looking, when I was at my other branch for a few years…because I was in (name of place) for ten years and then in (name of place) for two and a bit years and dealing with surgeries in (name of place) and round there, all those surgeries, and you might get no discharge sheet. You might get a discharge sheet. You might get a call from the hospital saying they're coming out but then a discharge sheet doesn't come. You might get no information at all and the patient just turns up and they've run out. And we're constantly trying to educate the patients, if you go in hospital tell us straightaway, or as soon as you come out of hospital don't leave it until you've run out, tell us the day you come out that you've come out, bring your discharge sheet in to show us, bring the tray in to show us if you've not got a discharge sheet. And then on the other side of the coin we've got to liaise with the surgery and say, can we have a copy of the discharge sheet? Okay. In previous cases they might have faxed it. Some cases you might have to physically pick up a copy from the surgery. But the latest is if you don't get it through PharmOutcomes will be, have you got a secure email address? So, with the advent of NHS Mail they'll now email us the discharge sheet, usually, but you might get some surgeries that are difficult, like the one that I dealt with in the area of (name of place). That was when the practice manager was very difficult, refused to release any information, and that was what really annoyed me at the time where she said, well the doctor's done the prescription, it will be right. How do I know?

HP17

R: So, the surgery will practically never tell you that a patient goes in hospital. It would be very unlikely that the surgery will communicate that. One thing that may happen is the surgery…you'll request prescriptions for a patient and the tray's going out later in the week, and then the prescriptions don't come back. So, then that prompts you to then get on to the surgery, well they've had 48 hours to do the script, why is the prescription not back? So, then you'll ring the surgery and they'll say, oh, so and so's in hospital, you don't need the prescriptions. Oh, right, okay, thanks for the information, thanks for telling us, we'll now put that tray on the hospital shelf. So…or…

I: But if that comes back with changes is that tray then…?

R: So, if the tray…so in theory you won't make the trays up until you've got the prescriptions, unless you want to get ahead and make the trays up off the backing sheets from the previous month, you know, copying the previous information, but then obviously a pharmacist isn't going to check it until they've been matched against the prescriptions and you've checked there're no changes. But one thing could happen is if the patient goes in hospital when you've already got the scripts. So, surgery's done the scripts, you've ordered the scripts maybe a week/week and a half early, you've got the prescriptions, now the tray's been made up, pharmacist has checked it, it's gone on delivery, driver gets to the house and there's no answer at the house. So, then you'll try and deliver it again the next day. There's still no answer at the house. So, then it might get put downstairs as a collection because it's failed twice for delivery, and then it could just sit there. So, yeah, so then it'll just go downstairs as a collection, and then it'll only stay downstairs for a while but you don't know that they're in hospital. And then it takes time. You've got a whole…(name) has got two shelves full of patients that are in hospital, because we deal with 200 trays, and every so often she'll ring up and have a chat with the clinical manager or the secretary at the surgery and say, what's going on with this patient, what's going on with that patient? So, she…the other day, actually yesterday, she was chasing that, say, oh, this patient's moved to this chemist. But they don't tell us they're moving chemist. Or…

HP2

I: Yeah. What happened before?

R: Beforehand, if the patient was admitted into hospital, we would find out via the patient or any other information, the patient, the GP, or our computer systems, if the patient had been before, who their chemist was. So, first it was sourcing who the chemist was. Going to speak to the patient to see if they had bought medication in, which had the label on to say who the chemist was. And then we would give them a ring and inform them that way. So, that's what we would do and then…so, we would let the chemist know when the patient went home, as soon as the discharge summary was written or the TTO, the discharge prescription, we would put a covering letter on the top of that, which was mainly for our staff, and we would have a member of staff in the dispensary who would fax. So, sometimes it could take a few hours, if the fax didn't go, or the next day the fax didn't arrive. So, it wasn't really timely and if the fax number was incorrect, there was a lot of things that held it back.

HP3

I: And what happened before the service was introduced?

R: So before... Which, I mean, I guess, I didn't really consider it bad because starting out, this is all I knew pretty much and it wasn't like it was anything crazy. So before, it was when we would do a med rec either the patient would have the compliance aid with them, so we would copy everything down and then just ring their pharmacy, or we'd ask the patient if we could ring the pharmacy and let them know that they're currently in. And then we would write in our med spec document saying we've contacted the pharmacy, these are their details, and we'd usually document whether it was weekly or monthly delivery, what day they would deliver it, if it was collected, and then we'd put their telephone and fax number. That's like a really thorough... Most people wouldn't put everything but we tried to put everything, all of that information. And if the patient had any more at home, or if the pharmacy had any left, or if they were going to send some more out later that week, or when the next lot was due, and if they didn't have a compliance aid, then we would usually ring them anyway just to find out what was in the compliance aid. And then on discharge, we'd always try to contact them, but most people don't have time to contact them about any changes. So in most cases, we would contact them just to see if they did have one ready, especially if there were no changes. If there were changes, we would try and contact them to say, these things have changed. A lot of cases, we don't have the time really to contact them, so we would just fax them through a discharge summary, and so that would be after we've dispensed everything and set up the medications, and we usually put it in... There's like a slot in dispensary where we've got a file where things are to be faxed to the pharmacy. I think sometimes the problem with that, though, is if the fax doesn't go through. There have been times where we've kept trying, it's the wrong number, or they've given the correct number and they're just not getting it through, so we just have to tell them over the phone, these are the things that have changed, so they just don't get any record, pretty much. I mean it was rare that happened but it did happen.

HP3
I: And what do you think in terms of the previous way of doing things, was that good or is this an improvement now with the...or not an improvement?
R: I would say e-referral is an improvement. The way before wasn't bad, but it was time-consuming sometimes, but it wasn't a terrible way of doing things, it was just... Yeah, it was fine.

HP4
I: Right. And thinking about the e-referral service and the introduction of it couple of months ago back in February, just want to talk about how you perceive that, how you think about that to start with. What do you think it's there for, what do you think the purpose of the service is?
R: For e-referrals?
I: Mm.
R: To kind of I guess standardise communication, to make it more reliable, because before it relied on people kind of, you know, faxing, ringing, too many chains in the…too many links in the chain where it fell down routinely because we'd constantly get phone calls with problems where it didn't happen. And so safety I guess overall, I think the aim is probably to do with patient safety, improving communication from secondary care to primary care in terms of medication changes in hospital.

HP4
I: …which is exactly what you said there. So what happened before, what happened before we had it?
R: Very unreliable process I think, resulting in lots of errors. It relied on human…kind of humans, basically, you know, staff doing lots of things. So basically the first step would be ringing the pharmacy, as part of the medicines reconciliation document we do on admission, we identify if a patient has a blister pack or not, so we record it in significant events as like a compliance aid section. So once we know they're a blister pack, when we get the discharge summary, then we would basically ring the chemist and say, Mr Bloggs is going home and kind of communicate over the phone what we were doing, if we were dispensing a new blister pack or if we were just doing a separate box, all that kind of stuff, and then we would fax a copy of the discharge summary over. So sometimes we'd fax from the ward directly, if we had time or for some reason the community pharmacy asked for it there and then, we'd fax it over directly from the ward, particularly from EAU we used to do that quite a bit because we had a fax machine in the office and it was just there and we knew it had been done then. If it went down to the dispensary for example or if it was from a weekend or Bank Holiday, then it would basically just go through the process in the dispensary which relied on an ATO picking up the discharge summary, noticing it was a blister pack one, relying on the fact that the pharmacist had filled in the paper form that went with it with the fax details and communication details on, and then that would get faxed from the dispensary to the chemist. And I think they would ring to check they'd received the fax. The difficulty sometimes, we found errors for example, sometimes you'd come in after like a week off on annual leave and you'd find a prescription sheet in your pigeon hole with, unable to fax, for whatever reason, query incorrect number, and that would have just been sat there for a week, so then you'd have to kind of pick it up and ring the chemist and say, have you got notification of this, no, the blister pack has already gone out with the wrong stuff in, lots of things like that really, you know, pharmacists forgetting to do the coversheets before they send it down, then the technicians having to ring up and chase it up, so just time-consuming, really longwinded, time-consuming and not reliable.
I: Yeah, and a lot of processes.
R: And delays in…I think that was the thing, there'd often be delays, particularly if a blister pack was processed on a Friday for example, then it wouldn't necessarily get…and I know a lot of community pharmacy aren't open at weekends, but some are, and that process, if it wasn't picked up till say Monday afternoon and they'd gone home on Friday, they're nearly running out of the next blister pack, aren't they, if we've only sent them home with a week. So then you're kind of chasing that up and you're relying on somebody picking up a fax, the fax working. That's the other problem we had on our ward, that our fax actually was really unreliable and used to break constantly, so used to take us forever to try and fax these things through and you had to hold the paper in a certain way. So just time-consuming really.

HP4
I: No, no, I mean, and I haven't either yet, so yeah. So what happens then, so that's on admission…
R: Yeah, so that order stays on their current inpatient chart throughout their admission, then my understanding is on discharge basically that order will automatically ping off…
I: Right. So when you then…do you have to physically do anything?
R: You don't have to do anything – yeah, my understanding is once you've put that referral on, that electronic order, when they are discharged, that will automatically notify the community pharmacy that they've gone home and they'll get a copy of the discharge summary.
I: So you only ever go into the system once, really.
R: To the order, yes, to the referral. So on discharge it's a slightly different process, there are certain things we have to do on discharge, like in the [QRG or 15:56] the guidelines they kind of set up with this, we have to annotate the discharge summary with documentation to say who's clinically checked the discharge summary, the time, the fact that we've asked the patient's consent if they're able to, and details of what we've

supplied. And as part of that we check that the discharge summary was accurate in terms of the stop-started amended medication. So we do annotate the discharge summary on discharge to make it clear to the community pharmacy what we've actually done. Because before I guess you would have ticked on the coversheet that we used to use, the paper sheet, we would have ticked on there, seven day supply, Venalink dispensed, 14 day supply, you know, it would have been in black and white on paper, so this is kind of another way of…
I: With the cover letter with the discharge summary handwritten just…
R: Yeah, basically, yeah, yeah.
I: Faxed.
R: Yeah.
I: So it's quite a change…

HP4
R: Just as we've said, probably time, the saving the time, knowing that it's going to happen which was always kind of in the back of your mind that it might not happen, so reliability. It's just easier, we're not all running around trying to do faxes and stuff in the afternoon and cursing the fax machine when it doesn't work. I would say it's probably too early to really see if it's…it's difficult, I would say I've not really been able to assess yet the impact it's had to see for patients coming back in, because it's still quite early days. It will be interesting to watch over time and see the e-referral patients who then come back in and see maybe…it'll be quite an interesting thing to do actually, wouldn't it, just to see…if we had the baseline data actually, it would be nice to see if we had less problems, difficult to pick up because there's so many factors, but I can only imagine it's going to make our life easier in the long-term. But only if the discharge summary is right.

HP5
I: Right, okay. And what do you know about the pharmacy e-referral service that's been introduced here, what do you think it's there for?
R: Well, I'll tell you what, I was very excited when I first heard of e-referrals. Only because within the unit where I work, the units, if a patient is discharged or if they arrive, one, is I don't know if they've been contacted by the hospital prior to transfer to my facility, that's the first thing. That information is not necessarily in the medical history, the medicines history or medicines reconciliation, that's the first one. And the second thing is, is because I work between units my contact with the community pharmacy that if they had a blister pack, for example, would be via fax, via fax. But quite often they would get discharged and I wouldn't be at that facility and I missed the opportunity to actually inform the community pharmacy of what the patient has actually been discharged with. So, the beauty of the e-referral, I'm not being funny, but the beauty of it, is that firstly we get to inform the community pharmacy that the patient is admitted into hospital, they get to know that electronically. And the second thing is whether or not I'm at the facility, once an e-referral has been setup they get a discharge summary once the patient goes. So, it takes the pressure off of me, trying to remember whether or not we've informed community pharmacists, et cetera. I've got a visualisation, I can see now whether or not a patient has had an e-referral, and I know that's going to go once they get discharged, so that takes the pressure off of me, I love it, I absolutely love it.

HP5
R: Right, now when a patient…when we are doing medicine reconciliations or drug history, on arrival we would see if a patient, for example, uses compliance aid. Now, then we'd have to find out where they get the compliance aid from. Then it means phoning the pharmacy to let them be aware of the admission of that patient into the hospital, before sending those drugs. Now several factors, quite often the telephone is engaged, some of them are not open all day, you know, especially if a patient comes at night, for example, you know, it's out of hours, so you are not going to be able to phone or contact anyone to inform them of an admission. So, that's what we do when they first come in. Now, when it comes to the next day you are doing more work, so that patient could slip through the net and quite often they did, because they'd moved on to another ward perhaps or something like that and hopefully somebody else picks them up. But, the documentation as to whether or not one has contacted the community pharmacy on admission was…I mean we did our best, it was a high percentage, there's no doubt about that, we were very good at doing this…but it did take more time, because then you are doing yesterday's work on the next day as well, to try and follow up a patient if that make sense.

HP5
R: Yes, yes, you are doing two days work into one because you'll filter into the next day, so, that was a pressure. And, of course, the other time that you get involved in supplying blister pack is at point of discharge. Of course, during the stay of the patient in the hospital there will probably be changes made, so the community pharmacy would need to know of such changes, and then one would have to fax that information. The community pharmacy also quite often wanted to make sure it was a secure fax that you had. Quite often faxes didn't go because some faxes shared the telephone line as well. So, you didn't know, so quite often when you think it's gone it's not gone. So, the information that the community pharmacy should be receiving they never receive, because of the type of system that we are actually using. Fax is an archaic system, it was good in its day, but the information highway, there's a lot more patients now using blister packs and so on. So, you know, if you were to fax information, the fax needed to be free, whereas they tend to be a shared line, I don't know because of costs whatever. So, the exchange of information could be halted by just the type of equipment we were using, and the circumstances under which we use them.
I: Yes, I know.
R: And faxes broke, faxes didn't always work and faxes weren't on every ward. That's the other thing as well, so if you had a fax on the EAU but if you went to another ward like on the L block et cetera, maybe one out of six wards had a fax machine. So, you know, it all impacted on an individual time wise.

HP6
R1: Yes, so faxing is still there but the benefit out of it, is I don't like…what I want to say is, it goes like that, we don't even think about it, as long as the right information and everything is on the system properly, it goes by the system. The system does everything, which is really good, it makes a great, great difference and gives me chance to save more money.
I: Yes, to do the other bits of your job, rather than just standing by a fax machine.
R1: Two hours out of eight, is like 25 per cent of my work time…
I: Was doing that.
R1: Yes, a significant amount of savings, which means I can do different things, even if I walked a patient, directed him to his clinic or anything could be beneficial, more than stood next to the fax and chase up the…it's a nightmare actually. Nobody liked it but I volunteered to do it, it's not easy, nobody liked it, as I mentioned and it used to be my line manager's job and he was always bubbling under, because of that job. It's

really stressful because you need to do it, several times for certain pharmacies, you still have some of those pharmacies that they don't like it, the fax, you need to send it more than one time. Sometimes five or six times. Two days ago, I had one of these nightmares and it went in the end.

HP7

I: What was done before that? You were here before…
R: Faxing.
I: Yeah, presumably.
R: So, a lot of time we are faxing, a lot of paper, what you would do if their fax machine wasn't working. So, like yesterday I had a patient who was discharged out of area.
I: Right.
R: So, I couldn't do an e referral and I got a phone call the next day to say that their fax machine wasn't working and they couldn't/they never received my fax. It was an error. So, I obviously had to speak to them over the phone and it's just not the best way of communicating. Thankfully, there weren't any changes to the medicines, but if there had been changes, it wouldn't have received a copy because their fax machine wasn't working.

HP7

I: So, discharge information before was sent by fax. And, how would changes for people on MDS or Venalink how would they have been communicated before, would that have been by fax?
R: Yeah. You mean a change?
I: Yeah.
R: They would just get a copy through a fax machine of the medicines we have supplied, and it would be annotated. What we put in the blister pack and what we didn't.
I: Yes.
R: Whereas now it's electronic it's a bit more better, it's not scribbled and writing. And, then they'll get a copy of…and, also with the electronic, they get an electronic copy of the full discharge summary.

HP7

R: I mean, we were supposed to ring…when I went to speak to patients when they came in and they told me they were on a blister pack, we were supposed to ring and inform them and sometimes they don't answer the phone. Because they can be very very busy, if it's like a Monday morning, it's just busy; the lines are busy and busy.
I: Yeah, the pharmacists are busy checking…
R: So, we're trying to ring them back but sometimes you just don't have the time.
I: Yeah.
R: Whereas with an electronic referral you don't have to ring them, you can just do it electronically and you don't have to worry about whether they are answering the phone or not. No matter what they'll know, they are in hospital.

HP9_

I: …on the discharge summary. And they might not match up. The discharge summary list should have been sent to the community pharmacist. What I'm trying to think of there is then if these things are happening, in what ways then is the referral going to help that?
R: Help it. Well, because I think the issue that…from working in (name of place) and now, the issue is with the discharges being sent to the chemist, it doesn't always happen because it is done by fax and it relies on the pharmacist actually calling the chemist, which they don't always, but their number could be busy for hours and hours. It doesn't sometimes…either the discharge doesn't arrive via the fax because there's a problem with the fax machine.
I: So you get discharges here by fax?
R: No, electronically…
I: Electronically.
R: …but it used to…
I: …it used to be by fax, yeah.
R: …it used to be…to the chemist it…
I: …by fax.
R: …used to be by fax until this new system came, and that used to be a nightmare. I know from working in the hospital and here, it just…it's a nightmare for both ends because it doesn't…fax is not really reliable and doesn't always work and the number's busy and sometimes they don't receive the full fax. So that causes a lot of issues and they always used to complain to the hospital that they'd not received it but then it's not always the hospital because they might have done their part but it's just not got through, so it's just completely not a reliable way of sending something that contains confidential information as well on the patient.
I: Yeah, precisely.
R: And to ensure it gets there on time and the chemist is [inaudible 22:38] I think that was just the wrong system to use. It just didn't work for anybody to be…

Theme 3b. Adaptation of communication across the network

3b 1 Code - Communication initiated by patient

C2_C3

I: Going back before that time, in the past generally if you like, has there ever been any problems with the medicines?
R1: The only problem we've had is the chemist that previously used to do them, next to the doctor's, for some unknown reason they changed over to the one on the road and we've had a few issues with them not delivering them on time. One time they ran out the injection, you had to go running up to get them. That was a bit with that, but before that everything was fine. With the other chemist it was spot on.
R2: There were that time when she was in hospital, the one time when they changed the tablets and she came home and you had to phone the doctor to get her to put her back on the tablets.
R1: Oh, that's right, yeah.

R2: I think that might have been the second time.
R1: They took her off the tablets, sleeping tablets.
R2: She [voices overlap 05:39] these tablets but didn't let us know.e-referral C2_C3 (
R1: That was then, that was just the once. But other than that, apart from these teething problems with this other chemist it's usually pretty not bad really.
I: And who helped to resolve that then? Did you have to phone…
R1: Just one doctor. Well, he's retired now, but I phoned my doctor…
I: Oh, he said…
R1: Yeah, he's really good. Well, he was, he's gone now, he's retired, so I've not had that situation occur since. So I don't know what would happen now it's all changed, he took retirement, the one who was in charge.

C2_C3
I: How did those problems get sorted out then? Were you…
R1: I just phoned them up.
I: You phoned…
R1: And then said to them, like, this has been ordered, it should have been sent.
I: So are you pretty aware of what your mum's on and what she needs to have?
R1: Yeah, because I've been doing it coming up to three years now, so you get used to it.
R2: Been doing it longer than that, before the stroke. Not the same medication, but you're quite aware of everything.
R1: My mum's had bad arthritis for years anyway, haven't you, mum, so I've kind of been calling down before work and doing things and then my mum's like done the meals when she was…so when I'm work and that, so I've been more or less calling here every day anyway before the stroke.

P1_C1
I: Yes. Have you had a review of those medicines with the local pharmacist at all? Have you ever talked to them?
R2: No. Not to the local pharmacist, no.
I: At the general practice?
R2: I questioned a couple of them with the pharmacist and that's only recently..
I: Right.
R2: …to say, can we not reduce one of the particular tablets that mum's on. The doctor had said that she was going to put them up before we went into hospital, that she was going to up her water tablet to 40mg, that never got sent through to the pharmacy, for whatever reason, there was a communication…
I: Right.
R2: It wouldn't have made any difference in all fairness, but again there was a difference in that.

P1_C1
R2: Which I did speak to the district nurse that came round to say, right, are these the same because again, the appearance is different.
I: Yeah.
R2: So, depending on what the meds are being used by the local pharmacist is different to what's obviously being given in hospital.
I: Yeah.
R2: Whereas if, somebody like mum, she wouldn't know to check that, she'd just think, yeah, that's right. So, it can be a bit…

P1_C1
I: I was thinking perhaps more broadly around that. I know what it was, so you said you've not had reviews with community pharmacists or anybody, the only reviews in terms of, let's look at all the different medicines you are taking, has been done at the GP practice. Is that done regularly at the GP practice?
R2: No. They review it.
I: But only when things happen?
R2: But they don't speak to us about it.
I: Oh right, yes.
R2: It's only because I got in contact and said can we change any particular meds.
I: Yes, right.
R2: So, it was only my instigating it.
I: Right, I'm with you.
R2: So, I know that they do do regular but they don't engage.

P4_C4_C5
R2: When he comes out now, I phone (name of pharmacist), that's our pharmacist, and I ask her if she's got the prescription, and she'll say, no, there's nothing come through.
I: Right, okay.
R2: So, then I phoned the doctor. Oh, we've got nothing. I did it this time, when he came out on the Friday I phoned (name of pharmacist), because…
R3: That was after speaking with (name of pharmacist) wasn't it?
R2: Yeah, 'cause she said it goes from here, the day he comes out.
I: Yes, I mean it should be, yes.
R2: So, I phoned her, on the [Saturday 00:08:40] I phoned her, I said, right, there should be a prescription for Monday comes through, or it's going to the doctor, and you should get it Tuesday.
I: Yes.
R2: So, I phoned, she said, well, I've got nothing, so I phoned on the Tuesday, she'd still heard nothing.
I: Right.
R2: So, I phoned the doctor, but you get the receptionist, oh, there's nothing here.

P4_C4_C5

I: But, do you feel therefore that they're not quite so keen to just tell would tell you, you have to do that?
R3: You have to find, yeah.
R2: Find out yourself.
R1: Well, if you didn't, you wouldn't know.
I: Right. Yeah.
R2: No one would come and, well your husband's in for this, you've got to go and ask.
I: Right.
R2: Because over the phone you get nothing.

P4_C4_C5

R2: Most of it's the same, so I can look at this list, what I've got, and it'll tell me what they're called
R1: I think, the old saying is (name) could give them a run for their money. As of, identifying tablets, they haven't come and told us what they are, this, that and the other, and she's had the pack from my old pack, and she's looked at them, and she's said, they've changed that.
R2: And what about when they left one out?
R1: And they left one out.
I: Right, so what happened when they left one out, what was that, was that recent or was that…?
R2: No, it's a bit ago.
I: A bit ago?
R1: You went chasing it up didn't you?
R2: I went chasing it, and said to (name of pharmacist), how come there's none in this?
I: So, there was something wrong with the actual prescrip…it was one not in there?
R2: Yeah, she said if you ever see that, you must phone me, she said, you must phone me right away.
I: Yeah.
R2: And when he comes out of hospital, she says, phone me and let me know he's out.

3b .2 Code How communication between health professionals works or might work.

C2_C3

R1: At this moment, like I say, from when she was in hospital, they came round and explained the situation with that, and like they explained about the injections. And then the nurses came and…she has quite a regular nurse, apart from when she's off, and she's really good, the community nurse, if there's any problems I'll just have a word with her and she'll explain that as well, so kind of got a good network really.
I: Yeah, that's interesting, because the community nurse is coming in, what?
R2: Every day.
R1: She's involved in our practice and she comes every day and if my mum's…right away she noticed, like the other week she came in your thing had come out, hadn't it, mum, she said, you'd pulled it down a bit more and your lip's a bit…right away she noticed that her lips were a little blue, so she put it…sort it out. But she's good, you can ask her anything and like she'll explain, how she'll say, right, she's going to ask about these injections, she said, because my mum's getting quite bruised now with the injections, she said, I want to have a word with doctor when I come back off holiday, which is this week, just to see if there's any alternative. So she's really on the ball, she's quite good.
I: So the community nurse is really helpful.
R1: Yeah, so it's kind of all…they all interlock.
R2: Communicate with each other.

HP1

I: Yeah, precisely, yeah. So, some limited information coming through, information is difficult to unpick, unravel, difficult to act upon. How is that then fitting into the rest of what you're doing, in terms of your work? Is it slowing the rest of your work down, is it changing the way you work, or what?
R: I mean, the numbers that we're receiving, it's not really changing the way that I work, it's just something, as and when I get a notification, I just need to deal with it. I think that is part of my job role, so I don't think it's affecting me. I'm quite chatty and just speak to anyone, and anything anyway, but some pharmacists, some of our inexperienced band 7s, might be less chatty, so I think it's good, in that it promotes them to pick up the phone and call their local pharmacy and say, oh hi, I'm the pharmacist, what's happened, I've just received this notification? Which is the whole point in the whole three way communication, so I think it is good to have these every now and then. It's just, if it was a little bit more slick, it would be nicer.
I: Yeah. So, the information you're getting through is the issue, or how it's presented to you.
R: Yeah, how it's presented, yeah. And whether it needed to be presented, because if it has been dealt with, or if it was something that, one occasion as well, because the pharmacy, and again I raised this with (name), the community pharmacy get it after 72 hours, and we receive a discharge later, maybe within the week, so they might have sent us something, and by the time we've looked at it, we probably are coming to it, so sometimes it's a bit of, I feel, it maybe was just unnecessary, maybe just highlight the things that needed doing asap.

HP1

I: What do you think the impact upon, well a couple of things, the impact upon working relationships across those transitions, between yourselves and community pharmacists, yourselves and hospital pharmacists. What sort of changes do you think the service is going to make to those sorts of working relationships?
R: I think it will help with knowing who's who. 'Cause there's so many of us now, and all over the place, we're like ants. So, I think it will help us know who is in what team, and what their role is. And it will also, it's more streamlining, sending information across, so again, assuming it's three way communication, we could then just send a quick notification via pharm outcomes, to the community pharmacy, and they will then send one back, instead of being on the phone for endless hours waiting for someone to pick up, or…I think definitely, and also it's a formal way of communicating. Sometimes, there have been occasions where it's been, oh you didn't tell me that, oh I did tell you, why didn't you…you know? So, having it written down, formally, is also safer and a better way of communicating I think. And also, the hospital, definitely, it's useful knowing what ward they're in, and kind of…

I: Why does that help?
R: Because, sometimes, if there was a query, so on the discharge, they might not have put something, and we just wanted to know, it's easier to go straight to the ward, and say, do you remember this patient that you've just discharged? Yeah, so that was useful. Yeah.

HP10.
So we try to make sure that the next door pharmacist, doctors, whoever is managing the discharge, will know exactly when do we need that set of prescriptions, see if there are any changes for. And then we're looking out for scripts. Recently, it has become better and better. We will sometimes obviously chase things up or we will receive things: oh, there is an error here, there is an error there, we will question things, but sometimes realising they don't always necessarily always agree, the GPs, or with what's on that discharge, they will make their own changes, they will make their own little implementations, you know, whatever they do. We, ultimately, do what we're told, but at the same time, we watch their hands, we're making sure that this is going to be right. So we will be in the habit of still double-checking. We receive something that is in contrary with what the district was saying and we'll say, guys, is that definitely right, that furosemide, is it definitely...? It says there 40 and you just dropped it to 20. Oh yeah, because she's doing a blood test in a week's time so we'll then... So we always have that verbal communication, and once everything is cross-checked, then the preparation will start. We'll just prepare it. I always do an in initial check against the discharge. So, to be honest with you, my, (name) who does the blister packs, she's really good, she's so good that she always... I check but it's hardly ever that I pick up any errors or discrepancies, but I do it anyway, I'll always check the information on the script against the discharge initially, and then once I'm happy with that, then I will do whatever checks need doing, and then it's going out. So, yeah, I think that's the best way I can describe it really, giving you that kind of journey of that.

HP10.
I: Is that widening of general practice isn't it
R: Yeah, exactly, so pharmacists are very much now an essential part of the surgery, I think, there's no exception that this one is, I think it's overall. And they all engage with... Again, to me, my understanding is they engage with all kinds of activities to do with prescribing, every single thing I'll be talking through pharmacists. Very rarely these days I will speak to the GP directly unless they've got something, you know.
I: Well, that in itself is quite interesting, isn't it, that you use them...that's their role in some respects...
R: Yeah.
I: ...in that sort of middle ground between yourselves.
R: Yeah, like a bridge.
I: And the GP.
R: Bridging, yeah.

HP11
I: Yeah, that's fine, yeah, absolutely. No, that's perfect. So thinking about the pharmacy referral service, why do you think it was introduced? What do you think was the point of it, if you like?
R: Well, I think it's always been particularly good in (name of place) anyway because they've always let community pharmacists know directly, which a lot of places don't do. So (name of hospital) has always been a bit ahead of its time with that. And then they've always communicated directly with the GPs, where I know, like my in-laws live in the Midlands and they get a scrappy piece of paper when they come out of hospital, to take themselves to the GP. Presumably they then get a letter at some point but there isn't the direct contact that they have in (name of place). But obviously, before they were just faxing it, it's a bit hit and miss and sometimes they'd fax our phone number rather than our fax number and it's not secure, is it?

HP11
I: Yeah. So can you talk through what happens when you get that first…from the moment you get the admission notification?
R: So when we get the admission notification we'll just say, oh, this patient's in hospital, we're not sending any more deliveries, we're not expecting them to be coming and collecting them, put them to one side and then we'll just leave it at that, then 'til we hear something. And then…do you want me to move onto the discharge?
I: Yeah.
R: Yeah. So then when we get the discharge, we'll look at when they need it for. So we have a control sheet with everything written on and signed off and then any discharges from the hospital we keep in a folder for that patient. So then we would then look at what medication they were on before, what medication they're on now, any changes that have been made and then go and speak to the practice pharmacist, and then he would then generate the scripts and then we would then make those changes to our sheet. And it would be clinically checked by me or (name). And then we'd make the pack up and either deliver it or they would collect it.

HP11
R: Well, definitely because it needs to go to whoever is arranging the discharge prescriptions, doesn't it? There's no point in sending it to somebody who's not. So I imagined they were just sending it, he would then get that information, but he's not…
I: No.
R: …he's not doing…
I: It's not.
R: …so…but…
I: …he's the practice-based pharmacist, but yes…
R: Yeah, so what's the point of that, because he's then relying on us to go in with a bit of paper.
I: Yeah. That was a sort of …
R: So that is probably where it's falling down, if [five 28:02] didn't really know why he wasn't seeing that, whether they just hadn't allowed him access to the right email box or whether it wasn't being directed properly within the practice. But that's obviously…

HP12
I: Yeah, because you've already got it.
R: It sounds awful to say but if the surgery just do us a new lot of prescriptions, we prefer to check that against the discharge summary, just to make sure that everything is as it is on the discharge summary. I know the doctors have issued the prescription but we prefer, as a double check, to check it against the discharge summary. So, sometimes they were getting a bit…why don't you get one and blah blah…so, we've got that

information and like I say, it's the other way round now, we are taking it through to them saying, this patient has been discharged, here is the discharge summary, please can we have prescriptions.
I: Which is really really interesting.
R: Yeah. And we've got quite a good relationship with the practice pharmacist that's working at the surgery now, the previous one we didn't have, but that's another story! But this current one we've got a really good relationship and he's just like, yeah, if you can print me off an extra copy that's great, it's much quicker.

HP13
I: You could carry on supplying and not know.
R: Yeah, just, yeah, [inaudible 02:32], then I can inform the GP as well. The GPs…I think they're so busy that they didn't always pick up on the discharge or act on them, whereas I think we are doing that and we, kind of, did anyway. You know, it used to be that I would phone the GP once the hospital had phoned me to make sure that the prescription was coming and that they knew about about the changes. Half the time they hadn't read them, they hadn't got round to reading them, you know.

HP13
I: In terms of communication, you talked a little bit about the communication with the doctors there, what about the communication back and forward to the hospital, is there any communication there at all?
R: No, 'cause you can't really get through to (name of hospital) much, the pharmacy, you wouldn't be able to actually ring them up, no, you don't really need to with this, to be fair, 'cause once they've told you what the patient's medicines, it's all there so you wouldn't need to, I've never had to phone them up.
I: Right, yeah, 'cause one of the things that I was thinking is, you know, it's a bit linear, the information is coming out of the hospital, coming to you but actually, once they provide you with that information, that's all they need to really do, isn't it, unless there's any queries that you…
R: Well, if there's a query, to be fair, I think actually I would pass that on to the GP, if there was a real query. Because at the end of the day, I'm not the clinical person producing the prescriptions, so you know what I mean, if there was an actual, real thing that I thought, I'm not happy with this, I'd put on PharmOutcomes that such and such doesn't look right or whatever and inform the GP and then I'd phone the GP. I mean, I don't rely on just the email on PharmOutcomes, I do actually phone them if there's a problem, I would throw that back to the GP, not the hospital and I'd let the GP deal with the hospital, yeah, really.
I: There is that process of talking.
R: But we…I don't think many pharmacists would ever really try to get through to the pharmacy at (name of hospital) about anything, I've never had that arise, really, no.

HP13
I: One of the things that was thought of to start with, this would be a triangular communication between practice-based pharmacies, hospital pharmacies and community pharmacies. Do you think that would be…do you have anything to do with practice-based pharmacies at GPs?
R: Oh yes, yeah.
I: What are relationships like or do you talk to them about these?
R: They're not great, strangely, they're not great always, no. But yeah, we'll have to talk to them, yeah. Although I find actually a lot of them, the reason I'm saying it's not great at first, a lot of them are fine but I think now they won't talk to you directly, which I don't like.
I: That's interesting, yeah.
R: You have to pass the message to some receptionist. I don't like passing messages to someone to pass a message. If it's complicated I say no, I don't want to pass a message, just tell them I need to speak to them directly. I don't know why that is, whether they're busier or whether they feel that they don't want to be interrupted.

HP15
R: Yeah. So, if there are any changes, we will call the doctors and ask them if they've received the discharge letter. Otherwise we can fax it to them if they want.
I: Are they getting it after you get it?
R: No, the online one, they seem to get it as well, yeah.
I: They get it at the same time. Yeah.
R: Yeah. We've never had such issues where there have not received it, but if they haven't, then we would send it to them.

HP15
I: Just to confirm with you that they are right. That's good. There's another part as well. We talked there just about the extra communication with the patients. And we talked a bit earlier before about that communicating extra…you know, your communication with the GP practices and so on. In what ways has this improved, you know, communication with the hospital? Or has it improved in communication with the hospital? Has it not?
R: It has improved. So, they don't necessarily need to call us and tell us if there are not any major changes. We just read and…yeah. Unless there's something very confusing or something then, we will have to call them, but we've never had any such issues, once it started, yeah.
I: Yeah. Were there before? Were there times when the hospital were calling you or you always check back with the hospital about a patient or something?
R: There were a couple, yeah. So, what would happen sometimes, is the patient would say, oh I've been started on this one or my dose has been increased. But maybe a page has been missed out in the discharge they've sent to us. So, we have not seen that, yeah. So, we have to check with the GP surgery if they received a full discharge and was it in their discharge. So yeah, there was a lot of time that was used up in that chasing up to see what's happening. Or we ask the patient if they have a copy with them, they can bring it in and we can have a look. So, if a discharge has not been sent properly, we can at least use what they've been given by the hospital, and tell the doctors that we have seen the paper from here, and we take a copy and send it to them. Yeah. But that's reduced a lot now.

HP16
I: And if you've got someone in a big...where you've got lots and lots and you're dispensing loads of items and you've got someone who virtually that's their job nearly all the time it's a massive impact on them.

R:	It is and a lot of the time it is your support staff who deal with those patients first, so actually they're better off knowing that than we are half the time because they're the ones who are dealing with the ins and outs, they're the ones who are doing the coms on the sheets, they're the ones who are actioning all those phone calls and everything. And, yes, then we need to know about the clinical stuff, that's how it should be but actually and equally that good communication so, did you know...we'll always put, did you know X is in hospital? Yeah. Notification just come through, X is in hospital. And that is sometimes all we need to say that we're aware of it but actually then our team gets on with what they need to do. They do the note on the PMR, they'll do this, they'll do that, everything they will just sort out.

HP17
But I mean you've got summary care records, which is better now, but from past experience this new system of getting electronic referral is brilliant because you know instantly…you know when they go in hospital, you get an email for that and you can bring it up on PharmOutcomes, and you know when they come out, and you get…all the information that the doctors get you get, I'm assuming the same, or very similar, information. So, I've had problems in the past with one particular surgery, not mentioning any names, that wouldn't even give me a discharge sheet from the doctors, they refused, the practice manager refused to give me a discharge sheet, and this is what she said to me, not mentioning any names, was, 'well the doctor's done the prescription, it'll be right because the doctor's done it, and we're not releasing a discharge sheet.' But what happens if it's not right? What happens if the doctor's made a mistake? What happens if it's been transcribed wrong? It's my job as a pharmacist to check what they've come out of hospital matches what the GP's prescribed and I can't do that if I've not got a discharge sheet.

HP17
So, we're…one thing to tell you that is our branch here is probably a really good branch for you to pick for this study because we're a tray hub. We manage nearly 200 patients, or probably 200 plus the ones in hospital. So, it's a lot of pressure here. And we've got one girl, (name) that's spending…a full time member of staff, apart from…more or less full time, apart from on a Thursday, not that that's relevant, but she's constantly working on these trays, banging them out, constantly liaising, very good lines of communication with the surgery, with the hospital, with the Warfarin clinic. So, it's multidisciplinary, multiple different agencies, with the patients, with the carers, with the nursing homes. So, you've got to have good lines of communication.

HP17
R:	So, the sooner we get that information then you're reducing the delays to the patient getting their medication in community. The limitation of this system is the surgery. So, even though we get the discharge sheets I still can't do anything until the surgery accept the information at their end, process the discharge sheets, pass it to the GP, get the GP to then do new prescriptions, whether they be monthly, whether they be weekly depending on the risk, and then those prescriptions have got to come to us. Some of the surgeries don't do anything. They'll just sit on that information, it'll just sit on their computer, and they'll do nothing, and then the patient…
I:	Yeah. So, you're getting that information, which you didn't get before, but you can't act upon it until you've got the script, can you?
R:	We can provisionally act upon it. We can make sure we've got the drugs in stock, we can put the patient on a sort of chasing up shelf and have the…make sure…see what's changed, look to see if we've got any existing scripts. So, if they've put on the discharge sheet, no changes, okay, no problem, we'll just continue the trays as they were. We might use existing scripts, we might request new scripts if we need them, but if the surgery…or we can…it prompts us then to phone the surgery and say, wait a minute, we've had notification this patient's come out of hospital, we need new prescriptions for everything, why have you not done it? Oh, it's sat in the doctor's room, or, oh, sorry about that, we've had it a week but we've not done anything.

HP17
So, then this surgery manages their own prescriptions. So, then the other thing is…so one surgery, they do all their own prescriptions and we don't do any ordering, they have somebody that's employed full time just to do all the prescriptions and work out when they're due and print them all off. All we get is prescriptions and we just ask for a name. Not mentioning who this surgery is, of course, but I said, well if something's not there or something's not thingy you don't just assume it's right, you need to question everything; so why, for example, with this patient that was on 30 milligram of Citalopram and then all of a sudden it drops to 20, where's the extra 10 one that they'd only just started two weeks ago? Don't know, it's not arrived, they ordered the wrong prescription so maybe they've not done it. Well let's question it and let's ask them. Well we don't question this surgery. Yes, we do, we'll get back on the phone now and find out what's going on.

HP17
I:	And you're probably not doing quite so much of the ringing back and forward you said in the beginning, back and forward to the GP?
R:	No. That's less…so there's less mither. So, it's being more efficient, the system. So, you're moving the work around. Rather than being last minute you're doing some prep work before when it's worth the time…
I:	Yeah, precisely.
R:	…that's…the valuable time you put in to do that.

HP2
R:	The e-referral?
I:	Yeah.
R:	Yeah. So, that we can inform the community pharmacy as soon as possible, when a patient is admitted.
I:	Right.
R:	And basically…at the moment, we are just doing patients who are on blister packs, Venalinks. So, basically just to let them know in a timely manner that the patient's in and to withhold the blister pack.
I:	So, you tell them on admission.
R:	We tell them on admission. And then when that patient is discharged, the chemist will get a message…an e-referral message from the hospital and they will receive the discharge summary of any changes and if the doctor has filled that incorrectly then they will be told whether there is any new medication, any medication that's been removed, any changes.

HP2
I:	So, yeah, that's really, really interesting. In terms of you, what the consequences of the e-referral service might be, let's look at that in a few different ways. Firstly, do you think it, you know, has actual value or benefit for…you know, you talked there it might have some negative effects to the pharmacy. Has it benefited things here in the hospital?

R: The pharmacy department, yes, because of the time it saves us. I think it will benefit a patient if it carries on and it's up and running, because if the chemists and GPs surgery get together…if this patient is coming in and out, in and out all the time, they can look into that, you know, get that patient referred back to the doctors surgery and say, why is this happening? You know is there is somewhere that this patient…are we failing the patient somewhere where the medications are? Is it a medication issue or is it a social issue? If it's medication we can sort that out.
I: Yes. Precisely. And is it because then that information is being shared or….?
R: It's been shared. Yeah. It's being shared by everybody. So, we all know about this patient when they are discharged.

HP2
I: What happens once the patient has left is going to maybe have an impact or whether they come back at all.
R: If, you know…once that patient goes home, if somebody then can have contact with that patient, maybe in their own home, or the patient goes into the GP surgery and speaks to pharmacists or technicians in there, just to keep up-to-date with that patient to say, are you fine with your blister pack? How can we help you? Are you finding any problems? That kind of thing could work in the community and I don't know if people are doing that kind of thing now. So, I don't know how it can help…like the hospital pharmacy, once we've done that referral and they've left, I don't know what benefit we can…
I: When you do the referral, do ever put an action on for the…right.
R: No. I don't.
I: So, you only tell them what's happened.
R: Yeah. We let them know that the patient's in or they've gone, and then they'll receive the discharge summary, which has…the doctor will write why the patient has been in, what actions they've taken, medication they came in on, if anything was added, if anything was stopped. Yeah. So, that's it then, we've…it's finished from the pharmacy department then.
I: Yes, then so then it's…
R: Then it is up to the community…
I: To deal with…
R: …to deal with medications, but then maybe social issues, that brought the patient in, whether it is medication-wise or not, I don't know. If it could help, it could benefit somebody in that way. Another team.

HP2
I: Yeah. Precisely. And if you were to make any changes to the service, what would you do?
R: I don't know. I don't know if this…I don't know if there is a facility to send a message to the chemist when we are doing a referral, when we're letting them know they are in. You know, maybe…no, I don't know.
I: Are there? Yeah. Maybe. I don't know.
R: So, if there is…which I've not even looked. I suppose if there was…and I will check when I go back, we could, you know, mention to the chemist, the patient didn't bring any medication in. Said, that they don't have any at home. Can you confirm or whatever?
I: Yes. Yes. So, on admission you could ask for some feedback from the community pharmacist. Yeah.
R: From the chemist. Yeah. From the community pharmacy. Whereas at the moment, you're speaking to a patient, who is ill and so, you know, you might be asking them and they might not know. And in that moment in time, don't want to say. So, it's not the correct time really.
I: Yeah. Yeah. And it's obviously a difficult one, isn't it? Yeah
R: If there is a communication where you can ask a question or send written information to the chemist, rather than phoning them up. But we always phone them anyway. So, we do the referral but then when it's a patient on a Venalink, then we have to phone the chemist, just to confirm that there aren't any changes or…to the one that the patient had brought in or the one that they've most recently supplied.
I: Right. Because the patient might not have brought in the most recent one

HP3
I: And who are the important people in the using of the service? I mean, as well as thinking more broadly, who are the important...?
R: As in the hospital?
I: Yeah, in hospital or in the community or whatever.
R: I'd say it would be us, because when patients come in, we're the ones that would have to identify that they're on a blister pack and we would send the referral out to the pharmacy, it wouldn't be the community pharmacy letting us know that. So it would only be one way, I guess, the referral, yeah.
I: Yeah, so it's not... That's quite interesting, in so much as that this is purely you sending the information to the community pharmacy. Do you ever get anything coming back from them?
R: No.
I: Have you ever seen anything coming back from them?
R: No.
I: So it's only...
R: A one-way.
I: It's a one-way street.
R: Yeah. And so I guess if they did have a concern...
I: They'd phone up.
R: ...they'd have to, yeah. So if there was something in the discharge summary that pharmacies aren't sure about, they will just ring us, pretty much, and there isn't anything that they can send through. But, to be fair, if they did send something through, the patient would still have to be here, they'd have to be reviewed, because I guess there isn't a system in place to see patients that have been discharged if the community pharmacy has an issue with it. Because when the patients are discharged, we move on to the next patients that are currently here. So I guess it should only work as a one-way system, to be honest, and then they would ring us if they had any queries.

HP3
I: Because when you send that, you send the discharge, and have you sent any actions at all for the pharmacy to do?
R: Actually, yeah, that's another thing. So in the discharge summary, we usually write down, saying, we've asked for the patient consent and they're aware that we're sending this e-referral. We'll put if we've supplied seven days, if we've supplied 14 days of the blister pack, or if we've done nothing at all. And then we'll usually put the time that we've clinically checked the prescription, just so they know that at this time we've

checked everything, and if anything's added after that we weren't aware of it, so they would know that it was checked by me at this time on this day.
I: Yeah, but do you ever sort of say to them, oh, I think this patient needs an MUR or NMS or anything like that?
R: No, but that would be a good thing to use it for also, so yeah.
I: Yeah, to actually have that as an option.

HP3
R: Because before, what we had to do was put it in as an order, so it's similar to the orders where the doctors do the prescriptions, so we'd have to put it in as a community pharmacy referral and then... Which, I mean, it's not much longer but it's just easier when you're doing the medicines reconciliation, just click yes, and then it will take you straight to it.
I: It takes you straight to it. And then what happens?
R: So then it will come up with the pharmacy search, you put in the pharmacy, and then, at the bottom, it's like a consent, saying, I have told the patient, they've consented, or, I was unable to confirm with the patient if they're okay with it, and then you just confirm and then it sends, so it's just like an automatic...
I: So it then sends that the patient's been admitted and what's happened?
R: No, I don't think it sends that. I think it just sends that the patient's currently an inpatient, so I don't think there's any information of why they're in...
I: There's no information, just that they're in.
R: Yeah.
I: Yeah, they're here. So that if they're doing anything within the Venalink they can then stop it going out.

HP3
R: Yeah, so I think that's the main purpose, just so that they put whatever they've got there on hold. I suppose then when they're discharged, they'll get the discharge summary and then I guess that explains everything that's...
I: They're given that and now we can... Yeah, it just stops anything...
R: Yeah.
I: ...the gap. So when the patient is discharged, what happens then?
R: So nothing really happens with that referral, to be honest, it's just after we've clinically checked everything, then, like I said, we put in those messages saying... But that's in the discharge summary, saying that they've consented and they're aware that the pharmacy is aware, we've supplied a new blister pack, or we haven't supplied anything, and then I've clinically checked it at this time.

HP4
I: And who are the important people in using that, who are the important people in that?
R: Well, everyone I guess, because it's like a chain, isn't it, I guess, it's like if one part is not working and the rest isn't, so if you don't put the referral in the first place which would be the responsibility of whoever is doing that medicines reconciliation, so that would be the pharmacist or the technician, if you don't do the referral in the first place, community pharmacy is not going to know they're in hospital. But if they don't pick it up, if they're not proactive in picking up the referrals, then it's not going to work either. So I would say probably equally important.

HP4
R: Yeah, that's right, which helps, you know, so we can then have more time to correct mistakes and prescribe on the ward and things like that. So yeah, I would say definitely saves time. I still the odd time, the only downfall with having the e-referral sometimes is you still end up having to ring the chemist on admission to clarify certain things. So if for example like a patient doesn't have their medicines with them from home, you might be able to see from their GP records who their nominated pharmacy is, but you might still have to ring that pharmacy just to check, do you do blister pack for them or do they not, it looks like you do, but I always kind of ring just to check, because I think it's easier just to sort it out then than for someone to get a decline in like two days' time or whatever. So I kind of ring them or sometimes might want to clarify the timings of the drugs, so if it's not clear, if they're on like 20 things and they're all white and you can't work out which one is at night-time, which one is teatime, you might just need to go through the blister pack with them over the phone and say, is that night-time, or if there's a mistake sometimes as well in the dispensing, in the actual blister pack you might say, is that a mistake, the label is not there but looks like it's in or… So there's still times where we have to ring them, but I would say overall it definitely saves time.

HP4
I: And we were talking about MDS and just the thing I wanted to come back to and about Venalinks and blister packs, MDS, compliance aids, whatever you want to call it, currently that's the only group of patients that the service is being used…is focusing on. Do you think it should focus just on that or do you think it could be used for discharges more generally?
R: Yeah, it's interesting, isn't it, because that section of the discharge summary is so poorly documented at the moment in terms of stop-start, change medication, I actually think it is also really useful sometimes just to notify the community pharmacy of changes generally. Because you've still got the same situation, imagine you've got your 80-odd year old male who's on lots of medication, we change everything in hospital, then we rely on the GP stopping that prescription say for the aspirin, you know, once they get the discharge summary, then say a secretary looks at it then passes it on to the GP, the GP then puts it in their pile of things to look through. A couple of days later eventually, maybe they'll get round to stopping the aspirin off the repeat prescription, but by that point, say like another lot of prescriptions has gone out or they've got one at the chemist, by the time the chemist gets the next lot of prescriptions, they could easily have got another supply of medication stopped. And if the patient's not totally with it with their medication, which lots of people aren't, they'll just take whatever arrives, then it definitely would, you know, you can see how it could work there as well. I sometimes have used it for stuff like that, so I know technically we're not meant to, but I have used it a couple of times for patients who aren't blister pack patients, and I've used it for more like a communication tool almost. So I think the two I use it for are for nursing home patients, and I wasn't sure to begin with whether they had blister packs or not, I can't remember which nursing home it was, but I rang the chemist just to check and they said, they're not blister packs, but they said to us, if you make any changes, would you let us know because they're due another month's supply of medication to go out this week. And I was like, yeah, that's fine, so I put a referral on and I put like in the comments section, and b) this patient does not have blister packs, but this is just to highlight any changes on discharge. So in that situation, I've already kind of used it for that to some extent, because I don't see why you couldn't.

HP5

I: Yes, so do you think that the purpose of the service, what it's trying to achieve then is that sort of joined up in communication…?
R: It links everybody, it absolutely it links everybody, there's not a break in the care of the patient so to speak. The community pharmacy know that the patient is no longer under their care when an e-referral is sent, so it means that they will withhold any blister packs, for example, or sending them because changes could be made. So, if they withhold it that means there's not the risk of the patient going home, say they go home with a week, and then going back into a blister pack, perhaps with a drug that perhaps caused their admission, the admission in the first place, that they still have at home, that has been sent to us as they've been in hospital. So, it really does complete the circle of communication between all disciplines involved.

HP5
I: So, some of the, for some of your patients because they've come, they are in that sort of stepping stone out of hospital, some of them would have already had the e-referral happen in hospital.
R: Yes, the majority would have.
I: Right, but you check that that's the case.
R: Yes, but I check to make sure that that's the case.
I: That's interesting isn't it.
R: Because, you know, even though in their records, in the medicines reconciliation or drug history might have that they use a blister pack, there are occasions where whoever has done the meds rec previously might not have the opportunity to do community pharmacy referral. And the reason why that might happen is, because the patient might not know which chemist they actually get a compliance aid from or anything. So, it's got that they use it, but nobody, they might not know. Because some patients come in and obviously, they are confused at times or whatever and it's a traumatic event, they might not be able to give that information. But they are more relaxed by the time they get to my facility and therefore, I make sure that I get that information, or I get it from the family. And it's important to have that information because at the point of discharge not all of my patients go back to where they came from initially.

HP5
I: Yes, yes, that's really interesting. In fact, that brings us back to these other points about who you think should be involved in the e-referral service, who should be using it?
R: I really think pharmacists, pharmacy technicians, I'm not sure. I think primarily the two of them, only because the e-referral information is for the community pharmacy and most of the liaising will be with the community pharmacy. However, we do get instances where perhaps doctors, especially out of hours will need to know about certain information with regards to patients. And they are told when perhaps we've gone home that a chemist might be open a little bit later and they might need that information. If they are competent, they can put an e-referral on. But I do believe primarily the pharmacy staff because they are understanding of the e-referral system, how it works, so it's better.

HP5
I: So, but it's moving from here to community pharmacy, is there any transfer of information back from the community pharmacy to yourselves, or to here?
R: No, no.
I: Do you think there should be?
R: It would be helpful. I suppose the community pharmacist will deal with us on a need to know basis. I mean at the end of the day if you want information from the community pharmacy, we will have to initiate it, you know. So, it would be great if the community pharmacy could let us know, have a complete history of what a patient has had from them, but it's never necessarily complete because patients are not necessarily loyal to community pharmacy these days either. So, the best source of history of medication will be via the GP surgery, where we already have access to the SCR anyway as to what has been dispensed, and we can just confirm that information with the community pharmacy. Having said that telephone systems are prone to being busy, a lot of people are using telephones to contact pharmacy et cetera. Whereas, if you can get an electronic confirmation of information safely that you need, then that could save time.
I: Do you know if it's been received and when you, when you send that through do you know that community pharmacist has gone oh yes, that's my patient.
R: Yes, because I phone them.
I: Oh right.
R: What I did, I'm not one for using a system and then assuming that it's worked perfectly.

HP6
Actually, there shouldn't, if the faxing, if it goes from the first time, there shouldn't be a communication between myself and the pharmacy. So the e-referral does, there's no need for communication at all, it's just because we are trying to send the information in the right way, it has to be sent like that because other ways of sending information could be, not effective. Or could carry mistakes, could end up with mistakes, like with the drugs. Because some drugs carry very similar names, like promethazine or promazine, sometimes a spelling mistake might change something to something else. Because e-referral or faxing, send the information printed clearly, not every time by fax it goes clearly but most of the time, it goes clearly, with all the details and it ends up with zero mistakes, if it's sent properly. But the faxing is time-consuming, while the e-referral, it's not at all.

HP6
Well, starting from the pharmacist who is looking after the patient and maybe the nurses, so then the pharmacist and the pharmacist staff as well, who receive the information, who receive the e-referral, they are involved as well, myself or course and maybe some technician as well, ward technicians are involved as well. I don't know if the doctors take part in that, I'm not sure about it.

HP7
R: So, they will get an email from [Pharma 12:46] pharmacy outcomes to basically tell them that they are in hospital. I have actually also used it for patients who are on methadone.
I: Right, okay.
R: Because there was an incident where the patient was admitted and I just, you are supposed to ring the community pharmacy and tell them that the patient has been admitted to hospital when they are on methadone.
I: Yes.

R: And, the line was busy all day, I couldn't get through. So, I used the e referral to communicate to them. So, the next day when I rang them, they were like, yeah we received your e-referral. So, that was good.
I: Yeah. And, that's useful.
R: Yeah, it cannot just be for compliance aids it can be used for that.
I: Yeah, and that can be useful because they can…
R: They'll know no matter, yeah if they are not answering the line or the phone has gone or something.

HP7
R: Yeah, it would be good.
I: …more of a…
R: Better communication between GPs and community pharmacists as well need to happen.
I: Yeah.
R: A lot of, yeah, when I was working in admissions I noticed a lot of poor communication between GPs and community pharmacies.
I: And, what could happen?
R: Yeah, medicines get stopped in hospital. And, then weeks down the line we find out that, they were never stopped in the community and it's just been on them and nobody has realised it until they are back in again.

HP8
I: And the e-referral... Well, actually let's move on to the e-referral. Let's go back to, you came into your present role in November, so presumably that was when you first heard about the e-referral? What was the first thing you, take me back to that time when you first heard it?
R: So I met (name) at the hospital who was on the NIPPS team but went back to the hospital fulltime and she mentioned it, so that was the very first I'd heard about it. And she said that it was a way for the hospital to communicate with community pharmacy. So when a patient is discharged, their information about their medicines will be sent to their local pharmacy that they use, whether that's their (name of pharmacy) or their (name of pharmacy) or one that's at their GP surgery. And she just mentioned a brief sentence or two saying that the NIPPS team might then be a part of it at a later stage and it would be, like, a three way communication between the hospital, community pharmacy and the NIPPS team.

HP8
I: And then in some respects therefore, how does that then, that what that is for, fit in with what you're doing in your role?
R: So, yeah, it's that extra step that if there is a query about the patient's medicines, that doesn't have to go back to the hospital, that can go back to either the patient's GP or the NIPPS pharmacist who potentially has the time to do a proper in-depth examination of the patient's medicines and make sure that they are on the correct medicines. So, yeah, it's adding an extra layer of safety and an easier way to communicate, I think.

HP8
I: Why are you getting fewer than the community pharmacists?
R: So every patient that is discharged on a Venalink, I believe, their community pharmacy, if they have one, if it's in (name of place), will be notified. But then we only get one if there's a query that the community pharmacist decides, needs to be addressed in primary care.
I: So effectively it's a way of the community pharmacies communicating back to you.
R: To us, yeah.

HP8
R: So I think everyone that I've gotten so far has already been actioned by the time I've received the e-referral. I think there's a lag because (name) did tell me why, she explained it, but basically there's a little bit of a lag. So whenever I get a, it's usually something like should the patient be continuing on both of these medicines because they do the same thing, and we think one has been added in hospital by mistake. So then I contact the lead pharmacist in that area and ask them who is working at this patient's GP practice next. Or if no one is that day, can they call the practice and find out whether this has been actioned already and to my knowledge it already has been in a hundred percent of the cases.
I: So there's already...
R: There's already been another, kind of, communication, which I'm not sure how or when that would have happened. But, yeah, so it seems to be more of a safety net than anything else.

HP8
I: Yeah, precisely. I think, you know, having in some respects a more rounded service. I'm wondering because the broad question there is who should be using it, who are the important people in it, and it seems, how that... The problem, I suppose, would be how that would be achieved, how do you get that, you know?
R: It's the "c" word, isn't it, it's communication. It's knowing not only what you do as part of your role, so what the NIPPS team can do but then also where you can refer on to, who your lead contacts are and how they can help. So we're doing some work on pain on looking at patients on high dose opioids and where possible reducing that. So we've met with the pain team at the hospital. They've got a psychologist, they've got a physiotherapist, so we can refer patients to them. But, you know, we didn't know that before someone said, oh, well, I know this lady who is a pain specialist nurse and she's really friendly and we could go and have a chat with her. So, I suppose, it's communication. I think we have that quite well with the hospital because a lot of our staff came from the hospital and know that world.

HP8
I: And then they get on to resolving whatever the issue is. Within that how does that impact upon the GPs, is that creating extra work in general practice generally or?
R: I think so. I think when the community pharmacy communicates with the GP surgery outside of the e-referral system, they might say, oh, yeah, the patient needs to see the GP, so the GP will then make an appointment. Whereas, actually one of the NIPPS team might be even better placed to do that review than the GP.

HP9_
R: So far I've only been highlighted one that (name) has sent over to me, but the pharmacy had already rang the GP practice the day before. I think because of that system, they'd rang the practice the day before and let me know that they'd got a problem with this discharge, so I had a

look at it. So when (name) sent me over this, I was like, oh, okay, yeah, I've had a look at it already, so they must have come across it because of that system and then flagged it up the next day and then…but I'd already sorted it 'cause [inaudible 03:47]…

I: Yeah, it's interesting, isn't it, because possibly then, because of the service, pharmacists are actually triggered to do something about it earlier. And though when you're getting it it's a bit later, yeah.

R: Yeah, so because (name) I think gets a lot of requests so it's basically she'll send them over but by the time she's sent them over it's probably…it's been done or by the time it flags up maybe on the system, it's already been dealt with. But this is so far what I've come across, that it, yeah, they've been flagging it I would say more often, 'cause usually I would do the discharge and then I would call the chemist but I think there's been quite a few instances where they've called me about a discharge and they've said, well, we've got this discharge from the hospital and the medications don't match, so can you have a look at it? Which is really good that they're being a bit proactive about it because…

I: So the starting off…so…but this is one of the things, isn't it? It then becomes like, it's out. At the beginning, we were talking about improving communication. If it's just sending out a communication, is there any communication back? Is there any communication between you and the community pharmacy? Is that rounded, that communication?

R: Yeah. Like I say, I've got…I do get communication. I think it depends which chemist it is, like I say. This one contacts us a lot more frequently. I don't think…I've not been contacted by other chemists…other than this one because it's next door…

P3

I: When you went into (name of hospital) recently, who talked to you about those changes of the medicines?

R: It was one of the ward doctors, see, I went into A&E, went into the EAU, and from the EAU I went into the heart unit…

I: Oh right, so you got moved about a fair…

R: Yeah, yeah. So I got a bit conflicted information, there was one doctor said that – I don't know if he said artefacts on the ECG [voices overlap 19:53]. He said, we noticed something on the right side of your heart which is normally related to people who have lung problems. So I thought, oh well, perhaps we're getting to the breathing side of it. So he said, we're going to do a CT scan to see if there's any clots in your lungs. And then somebody came in and said, we've booked you in for an echocardiogram, so I said, oh right, I said, I have had one of them before a few years ago. Well, we want to see what's going on. So I said, well, I have had the MRI, so this doctor, oh, he said, I'll see if I can get the pictures off that, he said, it might be better than the echo. So the next thing, another consultant comes in the next day and he's got trainees with him and he's got like a big TV on a stand that they're moving around, there's another chap with him, and he's talking to the students about my condition, then he turns to me and he said, we're going to do this scan on your lungs, so he said to see if there's any clots. So I said, well, they did a blood test in the A&E for that to see if there's any blood clots and he said there wasn't any. Oh, don't worry about that, he said, we'll do it this way – it came back clear, all the X-rays I've had have come back clear, you know. And I mentioned the echo and he said, you don't need that, so I said, oh, fine, no problem, he said, you can't get better than the MRI, said, fair enough, I'm not bothered – I got a letter yesterday for my echo.

P3

I: Apart from (name of pharmacist), did any of the pharmacists at the hospital come and talk to you at all at any stage apart from (name of pharmacist) about it?

R: Yeah, they usually come up on the day before you're being discharged, yeah, and they'll tell you what you, you know, we'll have your blister pack ready, any changes that the doctors have made will be included in there, and as I say, they've put a list of all the tablets on the blister pack, so you know, what they all are, but, you know. There's a lot of information given now from the pharmacy. And as I say, it's always whatever they say it's going to be, whatever is going to be in it is in it, you know, it's pretty…

3b 3 Code - Interrelationship between communication and relationships and the expectations around what that achieves or might achieve

HP1

I: Yeah, precisely, yeah. So, some limited information coming through, information is difficult to unpick, unravel, difficult to act upon. How is that then fitting into the rest of what you're doing, in terms of your work? Is it slowing the rest of your work down, is it changing the way you work, or what?

R: I mean, the numbers that we're receiving, it's not really changing the way that I work, it's just something, as and when I get a notification, I just need to deal with it. I think that is part of my job role, so I don't think it's affecting me. I'm quite chatty and just speak to anyone, and anything anyway, but some pharmacists, some of our inexperienced band 7s, might be less chatty, so I think it's good, in that it promotes them to pick up the phone and call their local pharmacy and say, oh hi, I'm the pharmacist, what's happened, I've just received this notification? Which is the whole point in the whole three way communication, so I think it is good to have these every now and then. It's just, if it was a little bit more slick, it would be nicer.

HP13

I: And you're getting that discharge a couple of hours after the patient's left the hospital.

R: At that point the patient's got a week's worth of tablets, right, therefore you do it quickly and reconcile it when they need a prescription, you've at least got a week.

I: To get it sorted, to get a new prescription done?

R: To get it, you know, and we're getting…that's, you see, very helpful because with that phoning business that we did before, it might not have been as prompt when they actually managed to get through to and then you were waiting for a fax. And it could be two days almost had gone through for all you know and by then the person's beginning to run out of medicines, you see. This is much better for the patient, much better, definitely.

I: Because that patient's not then anxiously wondering where their medicines are coming soon.

R: Well, what patients tend to do, to be fair, anyway, they wait 'til day six or maybe even the morning of day seven and then they phone you up and say, I've got no medicines left, you know, I've been in hospital. And that's no good 'cause this still happens with the non-MDS ones, you see. It still happens because we don't know they'd gone in hospital and it's amazing how many people do that. I think they just think everything's wonderful and it's all going to work so they wait 'til they're nearly running out and then they tell you which is not good.

I: Yes, 'cause that's actually not giving you that block of time to do things.

R: No, not at all, whereas with these MDS it's good and it's a shame they can't extend it, hopefully one day they will.

HP16

R: You get told they're in hospital.

I: But you don't get told why until you see on the discharge summary.
R: Yes.
I: So, as you say, it could be a fall, it could be an MI, it could be anything.
R: Yeah, it could be, even if it's just a suspected cardiac event, respiratory event, even that in itself can help us impact on what we do at the other end.
I: Yeah, precisely.
R: If it's, for example, a respiratory event they might have inhaler counselling in hospital but actually there's nothing wrong with us doing the inhaler counselling when they're out again. And that's the kind of thing, you see, it's that stepped approach.

HP4
I: And who are the important people in using that, who are the important people in that?
R: Well, everyone I guess, because it's like a chain, isn't it, I guess, it's like if one part is not working and the rest isn't, so if you don't put the referral in the first place which would be the responsibility of whoever is doing that medicines reconciliation, so that would be the pharmacist or the technician, if you don't do the referral in the first place, community pharmacy is not going to know they're in hospital. But if they don't pick it up, if they're not proactive in picking up the referrals, then it's not going to work either. So I would say probably equally important.

HP5
I: Yes, so who, are there, do you think…I mean one of the things when this started this service there were some thoughts there would be a three-way communication between community pharmacy practice-based pharmacists, GP in GP land and the hospital. Do you think that's happening or do you think that would be a good thing if it did happen, or what?
R: I can see the idea working. The problem is…well no, there aren't problems there are challenges. The challenge is this, if you are going to have three sets of disciplines working there has to be a starting point and it's pretty obvious a starting point would have to be ourselves when the patient comes in. Now, at what point are we going to involve say like the GP surgery. We already or the GP pharmacists, because we already have the information we need via the SCR right, which all of us can access if you've got the card.

HP5
R: Peace of mind for those of us who are doing drugs history, definitely. Peace of mind for patients knowing that their chemist is now going to know of changes that have been made. It also will…I was going to say improve relationships, but the communication between GP surgery and pharmacy is going to be strengthened because they both receive the same information. So, a community pharmacy can phone a GP surgery, confident that the GP surgery has received the same information. Because there's no way if you do an e-referral that one or the other is going to be, both going to receive that, so, it strengthens that. Basically, what it does it strengthens the relationship between all the carers whether they do primary or secondary or whatever, between the community and the hospital. And for me personally I do feel that patients are getting better care as a result or continuity of care. I'm not, because of working between sites, I'm not going to go to another site and scratch my head and think did I send this, did I send that, this and that, or you know. So, you know, it's a job done here and I can concentrate on this other facility here without worrying about back pedalling as to what I've done here. We call it…if one is not concentrating on the job they are doing, somebody else is going to be affected by this.

HP5
I: Yes, that's really interesting actually and you talk there a lot about that continuity of care, because of actually one…and you also talked there about the…in terms of that, in terms of patients because one of the questions there is, you know, do you think the service can improve care, will it improve care?
R: Yes, definitely.
I: In what, why will it do that?
R: Because the information received is accurate. It really is accurate; everybody is getting the same information.
I: Yes, precisely.
R: And you've not got a break in information. Because a patient, a scenario we've had in the past is a patient, we might send a summary to a patient, they might go to community pharmacy to get a supply of the drugs, community pharmacy isn't aware they've been sent into the hospital and gives them something, a new blister pack. Perhaps they might have had a couple of prescriptions left from prior to the admission and then they get drugs that have been, that has been stopped that caused hospital admission.
I: And could have caused a hospital admission.
R: Exactly, so yes, so definitely because there is communication between all services involved and you've got now exactly the same information moving between all of them. The risk of having errors like that made is minimised.

3b 4 Code- Limitations in communication - barriers, limits, communication that doesn't happen, no MUR or NMS, information that doesn't get sent

HP11
R: So we do things like…we know we do MURs and NMS, so if they were sending… One of the categories of MUR is post-discharge, which we hardly ever do, because – especially for Venalink patients – 'cause they don't come in often.
I: No, 'cause…
R: Not to…
I: …things are delivered to them, yeah.
R: Yeah, or they're housebound and that's part of the reason why they're on a Venalink, isn't it?

HP12
I: So, in what ways, has it helped, hindered or constrained maybe, the way you work? What challenges have there been?
R: I don't think there is any challenges particularly in the way that we worked. I think the main thing we have is obviously, there's us and there is another branch (name of pharmacy and place) and that is a branch that does basically just blister packs, nothing else. So, we have had quite a few referrals to us that should have been for them. But I mean it's not a big thing because we will just get the referral, it's not our patient, reject, it's not our patient, and then it's done. And then the lady at the training event, the second one, was saying the staff have to hover over a

map and if they don't zoom in, they will just pick the nearest thing. But I don't think really it's had any hindrances for us, any negative things, I think it's probably all been positive.

HP12

I: Yes, precisely. Has there been anything, you know, 'cause one of the things that was initially thought was that this would lead to MURs, whilst they are still existing, NMSs. Has there been any of that as a consequence of this?

R: I don't think there has probably been that much of that because a vast majority of our patients that are on the venalinks, we deliver to.

I: So, they don't come here.

R: So, they don't come, so we can't – we do have quite a few that collect, so we do the new medicine service with them and give them something relevant. Although it's a bit of an odd one because they've already been on it in hospital, they could have been on it two or three weeks in the hospital before they come to us. But I think it does, not necessarily give you an official MUR or an NMS but it gives you that opportunity for a discussion with the patient, are they happy with how things are, do they understand what changes they've got, and things like that.

I: And having that extra information on the discharge summary, how useful is that around those conversations?

R: To be fair, probably the ones that we see personally and have the conversations with are probably not the ones that have gone in with medication related issues. So, probably not so much.

HP13

R: Yeah, and liaise with the doctor and make sure that they're aware of the whole thing as well, if you do need new prescriptions 'cause I think they get so many discharges in practice, there's so many patients, they're not looking at them promptly, you know, often.

HP13

I: One of the things that was thought of to start with, this would be a triangular communication between practice-based pharmacies, hospital pharmacies and community pharmacies. Do you think that would be…do you have anything to do with practice-based pharmacies at GPs?

R: Oh yes, yeah.

I: What are relationships like or do you talk to them about these?

R: They're not great, strangely, they're not great always, no. But yeah, we'll have to talk to them, yeah. Although I find actually a lot of them, the reason I'm saying it's not great at first, a lot of them are fine but I think now they won't talk to you directly, which I don't like.

I: That's interesting, yeah.

R: You have to pass the message to some receptionist. I don't like passing messages to someone to pass a message. If it's complicated I say no, I don't want to pass a message, just tell them I need to speak to them directly. I don't know why that is, whether they're busier or whether they feel that they don't want to be interrupted.

HP16

I: Yes, right, what specifically were some of those issues then?

R: One other thing was not being able to return an admission notification, so you could return the discharge but if you were sent the wrong admission notification you couldn't return it, you had to accept it or leave it. So that was changed very, very quickly because we recognised that actually that's really important because that allows the discharge to go to the right place as well.

HP16

I: So it's more efficient but have there been any challenges from using it, have there been any difficulties?

R: I think with any new system there's always going to be challenges, I think the key challenge for us is getting the whole team onboard to recognise things very quickly and making locum workforce aware that these things are coming through.

HP16

R: You get told they're in hospital.

I: But you don't get told why until you see on the discharge summary.

R: Yes.

I: So, as you say, it could be a fall, it could be an MI, it could be anything.

R: Yeah, it could be, even if it's just a suspected cardiac event, respiratory event, even that in itself can help us impact on what we do at the other end.

I: Yeah, precisely.

R: If it's, for example, a respiratory event they might have inhaler counselling in hospital but actually there's nothing wrong with us doing the inhaler counselling when they're out again. And that's the kind of thing, you see, it's that stepped approach.

HP16

R: I think we've not seen it in our area, our area's probably slightly unique in that, candidly, our GPs are quite archaic.

I: There's quite a few single-hander GPs here.

R: We've got a couple of single-handers but the big practices also don't...they're too worried about, well, we can't talk to one pharmacy and not the other and all these things, and we're getting into all this debate all the time, it's like, well, actually if I've come to you with a question...

I: About a patient.

R: Not even a patient but actually a service or something then we're doing it for the benefits of patients, that's our patient cohort, it doesn't need to be the whole of the...

I: Yes, it's the patient cohort.

R: It's the patient cohort that we serve and there are a significant number at your practice. So I think in other areas I've seen it work very, very well, for us probably not as much but it is getting better.

I: Yeah, because I know a while back, I think, there were some single handers hanging on, as it were, and near to retirement.

R: Yes, we've got three or four actually in this area who are single handed but the issue is that's all great but actually you've got a NIPPs network there but the NIPPs network aren't necessarily linking in with the pharmacies in this area essentially, it could be because we have so many practices, I don't know whether it's because they don't get around to the pharmacies or what but we've never had a NIPP come in here to say hello.

HP17

R: Sometimes on the discharge…sometimes you might get a discharge…there's a care home near us here called (name of care home) and sometimes it'll say on the discharge, back in community, or it might…I mean they could do with being a bit clearer on the discharges. This is the bit that's a bit vague. So, sometimes it might say on the discharge sheet, back in community, or sometimes it might mention the word (name of care home) and then we know, well they've gone in (name of care home) but are there still in (name of care home) or have they moved from (name of care home) and then…and they're in respite and then they're then going back into community, into their actual residential home? So, it's a bit ambiguous there when that happens and that, when you've got to get on to the surgery, start asking more questions, or even possibly ring up (name of care home), but we practically never do that, but then it becomes guesswork, and then you're chasing, and then you ring the surgery, ring (name of care home), look at the discharge sheet again, get the surgery to chase up what's going on. But there's only a limited amount of time because we're under huge amounts of pressure.

HP17

R: Here's another one for you. So, you've got the middle man, which is the respite, or the attached NHS facility which is doing respite, or the nursing home, which could be a temporary nursing home just to let them settle back in and recover and then they go in community. Now you're adding a second chemist in them. So, a second chemist that deals specifically with this separate nursing home, or residential care facility, has got a contract with that nursing home to do it. So, then they'll change the nomination from us to them, which happened with a patient very recently, in the last few weeks. And it was, oh, why is this patient now nominated to a chemist in (name of place)? Don't know, maybe they've gone in there, they've got some random script, some antibiotic. Oh, wait a minute, I thought this patient was in this residential facility. Well I thought they were in hospital. So, they've come out of hospital but we've not been told and now they're with this chemist. Okay, well they get written off the books because we don't know what's going on with them. And now the daughter then turns up, oh, have you got any trays? They're ringing up, have you got any trays for this patient? No, they're nominated to this chemist in (name of place), this independent chemist. Oh, right, okay, oh, why's that? I don't know, you tell me.

HP17

R: So, the sooner we get that information then you're reducing the delays to the patient getting their medication in community. The limitation of this system is the surgery. So, even though we get the discharge sheets I still can't do anything until the surgery accept the information at their end, process the discharge sheets, pass it to the GP, get the GP to then do new prescriptions, whether they be monthly, whether they be weekly depending on the risk, and then those prescriptions have got to come to us. Some of the surgeries don't do anything. They'll just sit on that information, it'll just sit on their computer, and they'll do nothing, and then the patient…

I: Yeah. So, you're getting that information, which you didn't get before, but you can't act upon it until you've got the script, can you?

R: We can provisionally act upon it. We can make sure we've got the drugs in stock, we can put the patient on a sort of chasing up shelf and have the…make sure…see what's changed, look to see if we've got any existing scripts. So, if they've put on the discharge sheet, no changes, okay, no problem, we'll just continue the trays as they were. We might use existing scripts, we might request new scripts if we need them, but if the surgery…or we can…it prompts us then to phone the surgery and say, wait a minute, we've had notification this patient's come out of hospital, we need new prescriptions for everything, why have you not done it? Oh, it's sat in the doctor's room, or, oh, sorry about that, we've had it a week but we've not done anything.

HP3

I: And who are the important people in the using of the service? I mean, as well as thinking more broadly, who are the important...?

R: As in the hospital?

I: Yeah, in hospital or in the community or whatever.

R: I'd say it would be us, because when patients come in, we're the ones that would have to identify that they're on a blister pack and we would send the referral out to the pharmacy, it wouldn't be the community pharmacy letting us know that. So it would only be one way, I guess, the referral, yeah.

I: Yeah, so it's not... That's quite interesting, in so much as that this is purely you sending the information to the community pharmacy. Do you ever get anything coming back from them?

R: No.

I: Have you ever seen anything coming back from them?

R: No.

I: So it's only...

R: A one-way.

I: It's a one-way street.

HP3

I: Because when you send that, you send the discharge, and have you sent any actions at all for the pharmacy to do?

R: Actually, yeah, that's another thing. So in the discharge summary, we usually write down, saying, we've asked for the patient consent and they're aware that we're sending this e-referral. We'll put if we've supplied seven days, if we've supplied 14 days of the blister pack, or if we've done nothing at all. And then we'll usually put the time that we've clinically checked the prescription, just so they know that at this time we've checked everything, and if anything's added after that we weren't aware of it, so they would know that it was checked by me at this time on this day.

HP3

R: I didn't really have any expectations, to be honest. I think because I didn't really fully understand... I didn't understand what was going to happen. All I knew was...because (name) said it will just make things a lot more efficient. I remember at one of the meetings that I went to some of the other pharmacists weren't really happy about the whole e-referral situation. I think for them... But then I think it was mainly the senior pharmacists that weren't happy about it. I'm not sure if it's because they're so used to the system that we had before, but I know one of them said it's because the pharmacies will now be aware of why the patient's in and what's happened and it's too much information. But I feel like the more information the better, to be honest.

I: Why do you think that?

R: I feel like it's just better for them to understand why this patient's been in. If they're left in the dark, it will just be... I don't know what the word is. I think them having more information will help them with their work as much as it helps with us knowing fully what's happening in the

community for the patient. So it will help them understand, okay, this patient obviously hasn't been adherent with their medication and maybe we can do something better to help them be more compliant, I guess. Because, say, it's a case of their blood sugars are ridiculously high because they haven't been taking any of their medication, if the pharmacy's not aware of that they're just going to keep doing the same thing because they think nothing's wrong.

HP3
I: Yeah, precisely, so they know it's not going to be collected, and so on, which is really important. Beyond then just those sorts of things, are there any other reasons why it would be good to have it used more widely, or are there any challenges in using it more widely, do you think?
R: I'm sure there are challenges using it more widely. I guess one of the challenges would be, because we're inputting into the system, this is the pharmacy, and then we send through the referral, if we send it to... Like I said before, there are so many (name of pharmacy). Say I send it to the (name of pharmacy) on the other side of town and they've got all of this patient's information. I'm not sure, I think on their side they have to accept or reject, they can accept or reject.
I: Yeah, I think there is something, but I think if they reject, I think they then get phoned up by somebody, or something.
R: Oh, okay.
I: Something happens; I'm not sure.
R: So I'm not sure how it works on their side but I guess it is a case of patient confidentiality, because I've sent it now to the wrong pharmacy, and is that an issue with that? Say it was as pharmacy that the patient used to be at but they no longer want to go to, and they don't want them knowing any of their business.

HP4
R: We see so many medicines-related admissions, you know, because of errors that occur in transition because things weren't accurately documented on discharge summaries, that if we'd stopped something it wasn't documented, so then it might not have been on the prescriptions but it wasn't written in the text, so then it's just continued in community. It's that kind of lack of communication that really causes harm.
I: Yeah. And you see medication-related…
R: Yeah, we see loads, so we have another order set for medicines-related admissions, and I would say we tend to do at least two, three a day. It might not be all due to prescribing errors, some of it might just be side-effects of medication, inevitably, but yeah, we do see I would say on a daily basis we see problems where people have recently been discharged and there's been some kind of error happened in transition between when they've gone home, what they should have been going home with and what they should have continued and then what's actually been continued by the GP. And that could be lack of communication with the patient or lack of documentation on discharge, or just mix-ups generally.

HP4
R: Are continued, yeah, that's right. The only…I guess the only downfall slightly is that our system doesn't necessarily show that it's had a pharmacist clinical check. So if a patient's discharged but the nurses or the doctors don't tell pharmacy about the discharge summary, that discharge will automatically still ping off to the community pharmacy, but it might not have had a clinical check, so there might still be errors on that. That's one of the big risks I think in our kind of system…
I: Because unless they make any changes, they don't need to involve you, is that what…?
R: So we try to encourage doctors and nurses to inform us of all discharges basically so we can cast our eye over it, because a lot of the time you do see doctors writing, no changes to medicines, or… We did a recent audit on EAU where we found we changed nine out of ten prescriptions in terms of documentation and prescribing. So if you imagine that and if you imagine that say out of hours overnight if pharmacy aren't open or if a nurse just says, oh, there's no changes, I'm not going to bother telling pharmacy, we'll just discharge the patient, and then that automatically goes off and those errors are continued.
I: Errors can carry on, yeah.
R: That is still a risk, but it's difficult to get around that, so other than encouraging staff to inform pharmacy of all discharges, problem is pharmacy aren't there 24/7, at the moment we just have 9:00-5:00 really, 9:00-7:00 on EAU, so that is still a risk. So the problem with this e-referral I guess is that you can't guarantee it's necessarily going to be correct. If a pharmacist has been involved, you'd hope it was correct, but you don't necessarily… So that's why we annotate I guess if we've seen it or not, if we've clinically checked it, it puts our electronic stamp on it to say, we have clinically checked this. If it doesn't have that and there's errors, I guess the community pharmacy could then ring and say, is this right, has anyone actually seen it from pharmacy, is it…
I: Because you do the order at admission, if you then don't get involved in the discharge, it goes without…
R: Yeah, that's it, it goes. It goes, yeah.
I: …you having anything…
R: That's right, yeah.
I: And it's about keeping people…
R: It really is, yeah, so there is still an element of communication and unreliability to some extent. It's just better than it was. Yeah.

HP5
So, before you briefly, sort of, like mentioned there about faxes and stuff, what was the process do you think before it came in?
R: Right, now when a patient…when we are doing medicine reconciliations or drug history, on arrival we would see if a patient, for example, uses compliance aid. Now, then we'd have to find out where they get the compliance aid from. Then it means phoning the pharmacy to let them be aware of the admission of that patient into the hospital, before sending those drugs. Now several factors, quite often the telephone is engaged, some of them are not open all day, you know, especially if a patient comes at night, for example, you know, it's out of hours, so you are not going to be able to phone or contact anyone to inform them of an admission. So, that's what we do when they first come in. Now, when it comes to the next day you are doing more work, so that patient could slip through the net and quite often they did, because they'd moved on to another ward perhaps or something like that and hopefully somebody else picks them up. But, the documentation as to whether or not one has contacted the community pharmacy on admission was…I mean we did our best, it was a high percentage, there's no doubt about that, we were very good at doing this…but it did take more time, because then you are doing yesterday's work on the next day as well, to try and follow up a patient if that make sense.

HP5
I: So, some of the, for some of your patients because they've come, they are in that sort of stepping stone out of hospital, some of them would have already had the e-referral happen in hospital.

R: Yes, the majority would have.
I: Right, but you check that that's the case.
R: Yes, but I check to make sure that that's the case.
I: That's interesting isn't it.
R: Because, you know, even though in their records, in the medicines reconciliation or drug history might have that they use a blister pack, there are occasions where whoever has done the meds rec previously might not have the opportunity to do community pharmacy referral. And the reason why that might happen is, because the patient might not know which chemist they actually get a compliance aid from or anything. So, it's got that they use it, but nobody, they might not know. Because some patients come in and obviously, they are confused at times or whatever and it's a traumatic event, they might not be able to give that information. But they are more relaxed by the time they get to my facility and therefore, I make sure that I get that information, or I get it from the family. And it's important to have that information because at the point of discharge not all of my patients go back to where they came from initially.

HP5
I: So, but it's moving from here to community pharmacy, is there any transfer of information back from the community pharmacy to yourselves, or to here?
R: No, no.
I: Do you think there should be?
R: It would be helpful. I suppose the community pharmacist will deal with us on a need to know basis. I mean at the end of the day if you want information from the community pharmacy, we will have to initiate it, you know. So, it would be great if the community pharmacy could let us know, have a complete history of what a patient has had from them, but it's never necessarily complete because patients are not necessarily loyal to community pharmacy these days either. So, the best source of history of medication will be via the GP surgery, where we already have access to the SCR anyway as to what has been dispensed, and we can just confirm that information with the community pharmacy. Having said that telephone systems are prone to being busy, a lot of people are using telephones to contact pharmacy et cetera. Whereas, if you can get an electronic confirmation of information safely that you need, then that could save time.
I: Do you know if it's been received and when you, when you send that through do you know that community pharmacist has gone oh yes, that's my patient.
R: Yes, because I phone them.
I: Oh right.
R: What I did, I'm not one for using a system and then assuming that it's worked perfectly.

HP8
R: And I'm not sure that we have that with community even though some of our pharmacists currently work as a split job in community pharmacy. But I certainly haven't met any community pharmacists since I started, which is bizarre when you think about it.
I: Yeah, there is communication but it's, sort of, like... And some of the communication may well be, sort of, ad hoc or informal, e-referral is trying to make that more...
R: Standardised, yeah. I don't know if I should say this because it's just an impression that I get, but some of the NIPPS team I think are a bit confused about why we're getting these from community pharmacy and it's been, you know... I've been sending this email saying, oh, can you just check on this patient, here's all the details that I've got. I go, okay, but I don't understand why, you know, where's this come from. Even though we've told them about the service many times and in the lead up to it and preparing them. And then obviously if I'm on annual leave or I'm sick, we've got a system in place for someone else checking the email, so everyone is aware of the system, but they just don't quite see the value. And I get the impression that that's because it's coming from community pharmacy.

P1_C1
I: So, when the blister pack, did that come from the local chemist?
R2: It comes from; it's a dispensing chemist, which is part of (name of community pharmacy).
I: Right, okay.
R2: And, then it gets sent to the local chemist, and then it's collected and then brought through.
I: Right. So, was there any conversation with the local chemist about…?
R2: No, because the main carers pick them up but even when I've picked them up there is no, it's just I go in and I say prescription for…and then they say, right, and that's it, there is no conversation.
I: No conversation. Have you ever had any conversation with, you know, a pharmacist or a chemist about your medicine.
R1: No, never

P1_C1
I: And, when you were in the hospital, did you talk to the pharmacist at all or anyone in the hospital?
R2: Not really, no.
I: No.
R1: There was this one lady that came.
R2: That (name) that came and talked to us about the research.
I: Yes.
R2: But the pharmacist didn't. So, when I got my blister pack for mum when she was discharged from hospital, there wasn't anything told about what needed to be done?
I: Right.
R2: Even while mum was in hospital, she was on restricted fluids.
I: Right.
R2: But that wasn't given over either to say, you need to continue this going forward, which I have addressed with the GP and said can you look into that.
I: Right. That's interesting isn't it? So, no one came…
R2: No, to talk through the meds.

P1_C1

R2: And, quite interesting while we were on the ward, 'cause mum has lamotrigine, which is part for the bipolar.
I: Yeah.
R2: Because the staff weren't aware of bipolar symptoms and the medication for bipolar, they were saying to mum, when she was saying to them, what am I taking these tablets for, they said, that's in case you have a seizure. And, I'm saying, but mum's never had a seizure, she doesn't have epilepsy.
I: Right, yes.
R2: And, because some drugs work with epilepsy as well, but because mum has, bipolar that's why she's on the lamotrigine.
I: Right, yeah.
R2: But the staff at the hospital weren't aware of that.

P1_C1
I: So, how do you feel about that, the when you were in hospital, those changes were being made, what do you feel about that, the explanations that were or weren't given?
R2: Well, there wasn't much in the explanation as to why things were changed other than the salt levels needed to be brought back up. But some of the tablets that mum's on I've never heard of and, again, they weren't explained as to why they were being used.
I: Right, yeah.
R2: So, I don't know. And, of course, there is no information to say, you need to watch out for this or avoid this.
I: Yes.
R1: But I take 17 tablets a day.
I: Right, yeah.
R1: And, in my own mind, I don't need all them.
I: Right, yeah. R`: You know.
I: But has anyone talked you though and said why you need to be on those 17 tablets?
R1: No.
R2: We know about the bipolar tablets and the reason why that is, because it's mood levels and making sure that you are not overactive.
I: Yeah.
R2: But a lot of the meds that mum's on she has been on for many many years. So, you know.

P1_C1
R2: That's why they wanted to put us on the new drug, which is the apixaban.
I: Right, okay.
R2: Which again, wasn't really explained about, it was just saying that you didn't have to go for as many blood checks.
I: Yeah, precisely.
R2: But they couldn't tell us what possible side effects were because it's a newish drug in terms of medicine.
I: Yeah.
R2: At the time, I think it had only been around ten years.
I: Yeah.
R2: But the people that they'd worked with and dealt with, patients they said they've had nothing that they could say was really serious and they may need to revert back to the warfarin.

P2
I: Do you think it's better having the blister pack then?
R: Yeah, because…but I just take it, I don't know what it's for, but I just take it.
I: Keep taking the tablets.
R: I sort of think well, it's up to them to make sure the doctors and the chemist, to make sure that they're giving me the right medication. I don't know what I'm taking.
I: Do you think they are…do you think…how…are you sure they are giving you the right stuff, I mean is that…or do you worry that they are giving you the right things?
R: I don't worry about it because as far as I'm concerned, they should know what I need. I don't know what I need but now…
I: So, you don't worry about that?
R: I don't worry about it, because to me, it's not my responsibility to take the right medication, its up to the doctors and the chemist to do the right thing, and I just take it and I think, oh well, and then I look at all them and I think, what are they all for. You know, I mean it's got the names on.
I: But the names don't mean much to you.
R: The names don't mean anything to me. You know, oh swallow it whole, take it in the morning, you know, so that's what I do.

P2
I: One of the things we've talked about a lot, and I'm going to wind up in a minute because I think we're coming up to half an hour or so, is about the fact that no one has talked to you about these medicines, which is really interesting. Would you like an explanation from people, who would you like to talk to? Who do you think would be the right person to talk to about them, your GP or the doctors at the hospital or who? Who do you think would be best placed? Or the chemist?
R: Maybe the chemist, like I understand the specialists at the hospital don't have the time, you see and that's what me daughter wants.
I: But now you're with the, I think from what you're saying, you're with the chemist, where they've got one chemist that deals with all the blister packs.
R: Maybe the chemist is the best to talk to.
I: But there's not…you haven't got your local…there's not one just around the corner is there, where you walk in and you go actually have a chat with them. Do you think having a chat with the chemist would be helpful, to go through what those medicines are?
R: Yeah, but you see me daughter, she would have liked to have sorted it before I came out of hospital, but it was just brushed under the carpet.
I: Yeah.
R: You know, and she personally asked, and the doctor said, yes, we'll sit down with your mum and that never happened.

I: Never happened.
R: You know, and it didn't happen.

P3
I: Who do you think is best at helping you with your medicine taking, who gives the best advice and best help around what you need to do with your medicines?
R: That's a difficult one, that, because a lot of the time if you don't ask, you don't get told, you know.
I: Yeah. So you feel a lot of that is down to you then therefore…?
R: Yeah, yeah, if I don't ask, I don't get to know, I don't know why, perhaps it's your old medical profession thing where a little information can be too much, you know, I don't know, I don't know because I love the NHS the way everything is going, the informality of it and the friendliness of it it's all first names, you know, I think it's brilliant and it puts people at their ease. But as I say, there may be that little secrecy over the meds and…
I: Yeah, and passing information to you, yeah.
R: Yeah, yeah, I know and I'm not telling you, you know, it keeps them in a little position of power if you like, yeah, control I should say.

P4_C4_C5
R3: The pharmacist come round.
R2: The pharmacist come round. […]
I: Right.
R3: He came round, but that's the first time I've, or you've ever seen him as well isn't it?
R1: Yeah.
I: Oh, this is from the GPs'?
R1: Yeah.
I: Oh right.
R3: He made a house call.
I: Oh right, okay.
R2: Because (name of patient) couldn't go out.
I: Right.
R3: But normally, you do not get a house call from my dad's GP, they do not do house calls.
R1: You're lucky if you get a phone call.
R3: They tell you to ring Out of Hours, no matter what time of the day you call. It's been known for only one…
R2: They're not on about that (name), they don't want to…
R3: No, I'm just saying.
I: Yeah.
R1: Let (name) have her say.
R3: They only have one doctor on, in some cases, so it was unusual to get the pharmacist out here
I: And he actually came out?
R2: Yeah.
R3: He came out on that one occasion.

P4_C4_C5
R2: When he comes out now, I phone (name of pharmacist), that's our pharmacist, and I ask her if she's got the prescription, and she'll say, no, there's nothing come through.
I: Right, okay.
R2: So, then I phoned the doctor. Oh, we've got nothing. I did it this time, when he came out on the Friday I phoned (name of pharmacist), because…
R3: That was after speaking with (name of pharmacist) wasn't it?
R2: Yeah, 'cause she said it goes from here, the day he comes out.
I: Yes, I mean it should be, yes.
R2: So, I phoned her, on the [Saturday 00:08:40] I phoned her, I said, right, there should be a prescription for Monday comes through, or it's going to the doctor, and you should get it Tuesday.
I: Yes.
R2: So, I phoned, she said, well, I've got nothing, so I phoned on the Tuesday, she'd still heard nothing.
I: Right.
R2: So, I phoned the doctor, but you get the receptionist, oh, there's nothing here.

P4_C4_C5
I: Yeah, I know which one it is. So the pharmacist should have known what the medicines were, but it's about getting the prescription from the doctors.
R2: The doctor, that's right, she said, I can't do anything.
I: They've actually got to write the script haven't they?
R3: Yeah. I have, in the past, had to go into the doctor with his discharge notes.
I: His discharge summary, yeah?
R3: Yeah, over a week past him being discharged.
I: Yeah, because you come out with a week's supply, and then when that's gone…
R1: We got some this morning.

P4_C4_C5
I: So, did they get sorted, those problems with those medicines?
R2: Well, I phoned and she said, what was it, I'm sorry there's nothing come through. I said, well I'll tell you what, I said, it better be through for Thursday, I said, or my husband's got no tablets.

I: Yeah.
R2: So she said, well just a minute. Oh yeah, they've found it.
I: Yeah.
R2: They've never passed it through. And I know he takes his time for signing them off.
R3: There has been one occasion, where I have been chasing a prescription from morning, that should have gone through to him, and I've waited and waited, to the point where I've gone down to the…I've phoned all morning, oh, he's in clinic now, he'll do it when he's finished clinic.
R1: Yeah.
R3: And I've gone into the surgery, and asked for him. Oh, he's on his lunch. And I've waited in the surgery, and he's walked through and I've gone, is that the doctor? Doctor, I forget his name now, but at the time I went, Dr (name) And he turned round, and he's going, and he's walking, and I went, Dr (name). And he's turned round, and I said, it's my…(name of patient) daughter, I said, have you got his prescription please, I said, only he's desperate for it? And he's looked at the girls and he's gone, oh has it come through yet? I said, yes, it come through at such a time this morning, and I said, he's urgent for it, the girls passed it through to you at such a time today. And he's just walked off, gone in his room and then sent it, I'm sat there, and he's sent it through to the pharmacy, and then the girls have said, oh, he's sent it through to the pharmacy…
R2: Won't come out.
R3: ..cause I was like that, I thought five more minutes, and I'm going to knock on his door, because I think they just…you was brought up with respect, my Mam and Dad always brought me up with respect, and now I have a few friends who are GPs and pharmacists and stuff like that, and they're ordinary people, but some of them get this chip on their shoulder, and you think, no, you treat people with respect. And to me, that GP, he's just taken on too much and can't cope, but he won't pay for the added locums and the extra staff to take on the workload he's got, and he's there for a service not to make money. If he's in that, he's in the wrong job.
I: Yeah, yeah.
R3: He really is. The run ins we've had with him, I'm sorry, that is why

P4_C4_C5
I: And do you think, sort of there and generally, and I know the way this might go, do you think you get enough information about your medicines generally, from people?
R3: I think what can happen sometimes, when the doctor does his rounds, I think they might sit and explain everything to my dad, but my dad…
R2: But he doesn't take it in.
R3: …at this moment in time, especially when he's got a fever, or when he's on his medication and that, his memory is not the very best, and he does not relay that information very well.
R2: He doesn't want to know, [inaudible 00:20:39], he just doesn't want to know.
R3: No, he doesn't retain the information.
R2: Doesn't register.
I: Yeah.
R3: And that does not get passed on to the staff, with the changeovers and everything like that, they don't take into account that my dad's memory is like it is, and you can tell staff on the ward certain things, they couldn't even read the instructions that TVN had left.
R2: For his legs.
R3: Because what we found, is the longer the patient's in, the longer the notes get, and they'll only read the last paragraph as opposed to, which is understandable, but…
I: Yeah.
R2: Well I mean, we know they're rushed off their feet, and they work to death.
R3: Absolutely. And you get staff that shine and staff that doesn't, but it's one of those things that…
R2: When it's your own dad or your husband…
R3: …that's where they're missing out, yeah.
R2: …you lose your rag.

P4_C4_C5
I: But, do you feel therefore that they're not quite so keen to just tell would tell you, you have to do that?
R3: You have to find, yeah.
R2: Find out yourself.
R1: Well, if you didn't, you wouldn't know.
I: Right. Yeah.
R2: No one would come and, well your husband's in for this, you've got to go and ask.
I: Right.
R2: Because over the phone you get nothing.

P4_C4_C5
I: Yeah, the chemist was going to know that you'd been in hospital and so on. So, was it just (name of hospital pharmacist) that talked to you about that, or did anyone else talk to you about that?
R2: Yeah, no, just (name of hospital pharmacist) wasn't it?
R3: On that occasion, it was just (name of hospital pharmacist). We have had occasions where other doctors, in the past, have mentioned changing medication, and what they're changing for. It depends on what ward you were on, and what doctor's there at the time.
I: Right, yes.
R3: When you get discharged, if he ever goes to the discharge lounge, they will sit there and go through the discharge notes with you won't they?
R2: Yeah.
R3: And go through the medication there, but they do say…
R2: But they won't tell you what it's for though.
R3: It depends on, I've been sat there with my dad in the discharge lounge, and sometimes the nurses will tell you what they're for, other times, again it's dependant on the member of staff who's dealing with you at the time.
I: Yeah, quite, yeah.

R3: You get some that'll just, you're out the door, and you get others that will sit and go through everything. It's a lottery

3b 5 Code - Ways in which Health professionals currently or could communicate with patients, either because of service or other wise

C2_C3

I: Right. Thinking about the medicines before that hospital, what sort of information was given about the medicines, who talked to you or to your mum about the medicines?

R1: Well, the last time she was in they noticed that the pregabalin, the one for the clot, wasn't working properly, so that's when they put my mum on the injections, so she has injections daily now for those. And then they informed us in the hospital that they'd be giving her injections from then on and she has the injections now instead of the tablet form. But they come round and…the chemist in the hospital pharmacy phoned us up and came to speak to us and went through all the meds and went through them with us and explained that they didn't have the sleeping tablet, the [inaudible 03:36], asked would I bring some down, the packet down while she was in, and then they just prescribed a slightly different one. Exactly the same sort of thing.

I: Right, so they did make some changes.

R1: Yeah, they changed the sleeping tablet, more or less the same sort, just a bit different manufacturer, and the injection she's on. She has injections now and she came out with some antibiotics for infection just to finish off, which she's stopped taking now. But they're pretty good actually at explaining and that. They phoned us up at home.

I: This is the pharmacy at the hospital?

R1: The pharmacy in the hospital, yeah. There's only the doctor really that changes anything when she's at home. The pharmacy just takes word off what they say. The doctor never has to come out when they want to give her something else or knock something down, they do…it all goes through the surgery then.

HP10.

I: I know also that when they refer, when they do the...and I think they do the referral at the point of admission and then the discharge summary automatically comes to you, but I think there is a potential for them to say, oh, by the way, would you like...can you look…counsel this patient, do an NMS, MUR, or something like that. Has any of that happened? Have you had sent …through the service?

R: Yes, it did, but it's not always that easy. So, normally, I will probably speak to the patient or my staff will speak, saying, how are you, are you out now? Okay, cool, what's happening? Have you got a week's worth? And we always kind of say, okay, cool, listen, we're just working on it so don't worry about it, or we speak to a family member. We will be trying our best, the surgery is working on your script now. When we get the script, we'll get it ready and we'll send it out. Oh, okay, thanks. So this is kind of happening, so the patients are reassured that someone is actually doing something about it.

I: Yeah, precisely.

R: So we normally do that. And then with one lady... You see, a lot of our blister pack patients are patients who wouldn't necessarily come down to see me. We don't have all this... It's really not feasible for us to go out to their houses and do and MUR there, a Medicine Use Review or any other service, but mainly that communication happens over the phone. I had one through that, one lady who was started with a new inhaler, because I do a service called Inhaler Technique Service and she came post-discharge with a new inhaler in her hands, she didn't have a clue what to do with it, which was just surprising. I think she was told, shown and told in the hospital, but I think she... I don't thinks he's diagnosed with dementia but she clearly had got some problems with cognition, she was saying, oh, I don't know what I'm doing, I don't know what I'm doing. So I said, yeah, come on in, have a look, this, that and the other, shown her again. I always give...I think it's called Safety Net. I told her, listen, any time you want, come back. So I kind of did another service, which we get paid a tenner for, showing them how to use their inhaler. And I think what I did as well, I've ordered her like a chamber to put...to get a new inhaler for her, she's not doing it right, she needs something else to make sure that works. So we'll always make sure or explore other avenues of communication: everything alright? You know, let me know if you experience any problems with it, and that's what happens really.

HP10

I: So is the e-referral service, therefore, having an impact upon that communication with patients? Is it increasing it or changing that communication?

R: Yes, most definitely. You've got those prompts there. You look at them, thinking, oh, right, I should maybe do this, I should maybe do that. That already helps. You're thinking, oh, maybe I could carry out a Medicine Use Review post-discharge. We did hardly any before. I don't think I did that many last year but it's not very often happening, or it wasn't the case. There is no system that doctors will think, oh, maybe I should send people to the community pharmacist so they can review their meds. You're picking on it yourself and you're thinking, oh, maybe I should let her know. And I kind of say it to them, listen, any problems with those new meds, let me know. But again, as I said to you, a lot of patients who we provide this with, they rely on the carers, they will be housebound, they will be suffering from dementia and the communication is really difficult. So there is, unfortunately, that element of it where there's no way you can really do much with those patients other than, like, speak to their carers or family members to reassure them that we are on the ball and we're getting it ready.

HP10

R: Yes, you're right. You're right, yes, it does. I'm not sure whether you are trying to refer to the fact that I've just said about communication with the patient. That's what you meant. Yeah, that's right. True, I didn't think about that initially, it just came out in the conversation. Yeah, you're right. It's kind of like you're looking at it, when you're dealing with that on PharmaOutcomes it says, what have you done? Commenced a blister pack, phone them post discharge, MUR, NMS, this, that and the other. So you're thinking, oh, maybe I should do that, you know, like even that or just... It's just basic communication and those people often, you know, they don't... You're not trying to patronise them in any way, but where I work, there are a lot of working class people, so they're just people that don't necessarily understand things very well but they appreciate, like, you know, like you look after them, you tell them, you... To be honest with you, if you ring them, give them a ring and reassure them, you save yourself phone calls later, oh, where's this, where's that? It's just you do it and you know everybody is happy, it's much better. And again, you know, as I said to you, maybe it's me, but I always enjoy doing clinical stuff, I always like to kind of have that broader picture overall. Communication makes them safe and makes the drugs they use safer, in my view.

HP12

I: Yes. What role have the patients and families got then?

R: It depends very much on the patient or family. But you will get some who will ring and say, my mum's come out of hospital and we can say, yes we know they've told us and they are like, great. And you get some that you don't hear anything from anyway, and just expect it to turn up as and when it turns up.
I: Yeah, precisely.
R: But it's quite reassuring I think, when patients or families ring up and say, just letting you know my mum's come out, yeah, we've got it and we've got the discharge, we are on with it, we are sorting it. Whereas before it was like, oh right, okay.

HP12
I: Yes, precisely. Has there been anything, you know, 'cause one of the things that was initially thought was that this would lead to MURs, whilst they are still existing, NMSs. Has there been any of that as a consequence of this?
R: I don't think there has probably been that much of that because a vast majority of our patients that are on the venalinks, we deliver to.
I: So, they don't come here.
R: So, they don't come, so we can't – we do have quite a few that collect, so we do the new medicine service with them and give them something relevant. Although it's a bit of an odd one because they've already been on it in hospital, they could have been on it two or three weeks in the hospital before they come to us. But I think it does, not necessarily give you an official MUR or an NMS but it gives you that opportunity for a discussion with the patient, are they happy with how things are, do they understand what changes they've got, and things like that.
I: And having that extra information on the discharge summary, how useful is that around those conversations?
R: To be fair, probably the ones that we see personally and have the conversations with are probably not the ones that have gone in with medication related issues. So, probably not so much.

HP13
R: Because I know I've got the discharge, I will then…I've got it early, I've now…because it's the PharmOutcomes, you have to tick things that you've done. One of the things I will do now which I wouldn't have actually done necessarily, I will phone the patient, I'll let them know that we know they've been in hospital, we've got a discharge, they're not to worry if they will be coming later in the week and blah de blah, you know, if it's the same I tell them. And then also when I go to the patient with it or whoever goes, it's me, usually, if they've been in hospital I will go and if there are changes I do a medicine review with them all about the changes to let them know.
I: In their home?
R: Yeah, in their home, yeah, what it is about and I would not have done that before. I didn't take it as…just because PharmOutcomes is very well designed 'cause it draws your attention to the fact that that's what you should be doing, so that's what I do. I've found that that's helpful, very helpful and I think the patients find it helpful, they've all been very grateful because maybe they haven't understood what the doctor had said to them, maybe they didn't know that their medication was changing or they weren't listening.
I: That new medicine or counselling bit that you're doing there, is that new or is that something….?
R: No, I really didn't do that much before. Now and again I would've done but not with every discharge at all whereas now I am doing, if it's changed. If it's not changed, I just phone them and say there are no changes, we'll be along with your medicines on Thursday as usual, end of story. But if it's changed, it's making me do things I would not have done.
I: That's fascinating, actually.
R: Whether everyone is, I don't know but I feel that it's definitely made a big impact that way.

HP13
R: I just feel I'm giving those patients a much better service because of this than I used to. I just feel that and I'm sure they feel that too, you know.
I: Because of that conversation?
R: Yeah, I really did used to think it was always the doctors' responsibility whereas now I had to be dragged into it and I prefer that. I feel as if it's a good…you're giving them a good service and making sure there's no errors, yeah.
I: And do you feel the patients value that?
R: Oh yeah they do, definitely, 'cause that's another thing, when I call at their house, I'll take the old packs away 'cause that's another issue if the medicines have changed, you don't want them picking up two weeks' ago, you don't know how long they've been in hospital, they might've been in hospital two months, they might have loads of packs there so I'll clear them out when I take the new pack. And I will write on the new pack so I know to tell them that this is the new, up-to-date pack and I get rid of all the others and take them away.
I: Right, yeah, and so the patient, yeah.
R: I didn't used to do that, I don't think I did used to do that, I just used to deliver it and say, that's your new medicines, you see. It's just a safer service, really, it's made it safer.

HP15
I: Right. Okay. Have you then…interestedly, have you had any sort of referrals where it's asked you to do an MUR or NMS or something like that?
R: So, for all of them, when we complete the request, first of all we have to accept when it comes to say that we have accepted the request. And there are a couple of questions at the end, that we have to fill in what we have done with the discharge. So, it doesn't really ask us to do an MUR but in every pharmacy…so once the patient has come out from the hospital, most likely we have to do an MUR on them, to make sure they understand the new medicines. Yeah. Yeah.
I: The new medicines. Yeah. Yeah.
R: Yeah. So, it's called a discharge MUR. So, we do do that for most patients, but some of them we deliver medication to them, and they are not happy to come in. They keep saying that they are not feeling well, so they won't come in. Yeah. So, we do do our checks on the phone, but it's not a face-to-face conversation, so that we don't do a recorded MUR.

HP15
R: It improves it in the way from…because we already know what happened to them while they were in the hospital. So, what treatment they had there and how it might have changed. So, it gives us an idea. So, we can ask them questions accordingly, yeah. So, they are lucky in a way…oh they feel like we already know about what's going on with them.
I: So, what responses have you had from patients then? Are they aware that now you know that information?
R: Yeah. Yeah.

I: And what have they said about that?
R: So, they are like, how do you know about it? They ask us those questions. Like we get an online referral on when you were admitted, and then when you were discharged. So, we already have got your medicine details when you get out of hospital. So, they are like, oh that's really good. That's quick. Because before…the experience they've had before is different from previously. If they were admitted before as well. So, they like…they like that change.

HP15
I: And has having the extra information helped with the MURs then, that you've come in to…?
R: Yeah, it does, yeah.
I: In what sort of ways?
R: Just because it tells us if there have been…so in a couple that I've seen, when the new medicine has been started, they'll tell us that the patient has been explained about all this, all the side effects and how to take them. And they also put on the note saying what the patient thought about it, or if they were confused, or they didn't understand it. So that helps us to decide how to speak to the patient about it. So, if they were confused, we can look for other information that might help them, from beforehand, if you need extra material. Otherwise, if they are here and we realise, oh they are not happy about it, we might have to look for it while they are here. And they might have to wait longer. But in this way, we can get the material that we will need to explain to them beforehand. And we also try to set a time with them, because we wouldn't like them to wait if they come here. So, we ask them, when's the best time for you to come in? So, we make sure everything is ready by the time they come.

HP16
And the other part I think people forget to recognise is that the GP deals with the patient, we deal with the family and the carers as well. And you can have family members coming in, carers coming in, all sorts of other things going, where's this, where's that, where's this, where's that? And you could get to a point on a Friday, for example, where this patient's been in hospital, we never knew about it, they need a blister pack now and what's happened? Well, we haven't had the information.

HP16
I: We're moving onto values of this actually now but how useful is that, that you have the same information that they have?
R: It's invaluable for us because we've never had that level of information before, we've never been able to make the same impact, I think, and that's the key thing, it's about impact to the patient and its value for that patient at the end of the day and for the system. The system needs to get value for its investment but actually medicines readmissions are such a key, I think six or eight per cent of admissions are due to medicines issues but actually why are the patients getting readmitted if there's an issue? There's a fault in the system there that's allowed them to have an issue and regularly multiple patients it's happening to where actually we could make an impact, it could just be simple AKA counselling. It could be very simple, a couple of words, it could be a couple of sentences, it could be just a point, but even if that can prevent an admission that's extremely powerful, and for the system even one admission saved is...it's massive. And not only for the system but actually the patient, that impacts their lifestyle, it impacts their care, it impacts their view on the health system, et cetera. So actually if we can improve patient outcome whilst delivering excellent clinical care for us that's very job satisfying and that shows us that we are making a huge difference to the patient.

HP16
R: So I think for us it would raise the profile in terms if it's done properly, then patients see the benefit that actually, do you know what, I didn't know you knew I was in hospital, for example, or actually that we're taking that step to have a different conversation. So the conversation isn't, are you out of hospital, do you need medicines, have there been any changes? Again, it's the same conversation that we're having with everybody, the conversation is around, we can see there's been changes do you need any advice on this, did the pharmacy speak to you about it, do you want to come in, we can try and do discharge MUR for you if there's been changes? All those things, this has been started, do you know what it's for, did they explain to you? And they may well have done but actually the patient's state of mind hasn't taken that information in. And that's very, very typical for us so we're able to make a much better impact.

HP16
R: And new medicines service wise actually that's where people fall out because they don't know, they've not had the information, everyone assumes somebody else has given them the information. When you look at inhaler technique and eight per cent of people use inhalers properly, shocking statistic, but actually we ask patients, did anybody ever show you how to use your inhalers? No. And we see it, has anybody ever shown you how to use it? Well, I use them like this. Well, did you know there's a better way to use it? And that's the conversation that we're having so it's more, it's clinical impact on care conversation rather than actually, do you want your delivery day changing, are you going to come in, who's going to come in for it and all these things? Because we already know that they're going to be housebound or they're going to have an issue with mobility or they're going to have something, something has been changed so it doesn't come as a shock to us when they're asking us about it.

HP16
R: I think we've not seen it in our area, our area's probably slightly unique in that, candidly, our GPs are quite archaic.
I: There's quite a few single-hander GPs here.
R: We've got a couple of single-handers but the big practices also don't...they're too worried about, well, we can't talk to one pharmacy and not the other and all these things, and we're getting into all this debate all the time, it's like, well, actually if I've come to you with a question...
I: About a patient.
R: Not even a patient but actually a service or something then we're doing it for the benefits of patients, that's our patient cohort, it doesn't need to be the whole of the...
I: Yes, it's the patient cohort.
R: It's the patient cohort that we serve and there are a significant number at your practice. So I think in other areas I've seen it work very, very well, for us probably not as much but it is getting better.

HP15
R: It's down to the NIPP I think.
I: Yeah, and it's down to that and what's happening with that.

R: And I've worked equivalent to a NIPP before and my first job was to go and say hello to all the pharmacies in the area because to me with the issues that we have in the market, stock availability, all those things, we're an invaluable resource for them. Is this available? No. Is this available? No. What's the alternative? Here you go.

I: And just being able to know what issues there are.

R: But it's also the medicine queries, they're meant to be there to support us with our medicine queries as well as the practices, so to resolve issues around medicines so if you have a complex patient who has issues then they need to be resolved and we want to have a point of contact for those.

HP17

I: Sorry, we were saying? Yes, talking to patients, yeah.

R: Talking to patients. So, you know…it might not be the patients specifically, it could be the carers. So, it could be the daughter, it could be the son and you will question…well the first thing is you'll question regarding the discharge is right. If they come out of hospital is that correct? When did they come out? So, then you're matching the dates because you might not always get the exact date when they come out of hospital, or hospital discharge could have been delayed. So, it might say a Monday but then they might have had no transport and they've not come out until Tuesday. So, then we need to prioritise, well did they give you seven days from hospital? Usually it'll state on the discharge sheet, Venalink given, seven days' supply. Or, no, no changes, no Venalink given. Then the hospital should really phone us and say, if there're no changes, right, well have they got a tray at home, have they kept the tray, has it been binned? So, then you need to liaise with the patient or the carer and say, well have they got supply, do they need a new supply? If there's…so then you need to establish exactly how many days they've got down to the day, down to what time of day because you could be under that much pressure trying to get a new script, can it wait until the next day? Then we've got a cut off for delivery. Or, can the family member collect it?

I: Yeah. So, there's quite a lot of conversation but are there more conversations as a consequence of having that information, or are there fewer con…is it…?

R: I'd probably say more. Well with the carers specifically?

I: Well, yeah, or the carers or with the patient as a consequence of having concerns.

R: I'd say because you've got more information you're going to have more conversations but in a good way, not in a negative way.

HP17

R: Yeah, I mean I've definitely done some NMSs based on new medicines that have been started. So, it does highlight that to us. Potentially I could do discharge MURs. I don't specifically, off the top of my head, think I've done any discharge MURs but I've definitely done NMSs, new recruits for MMSs based on…not necessarily based on discharge sheets specifically but it would have highlighted it to me. So, then it would have prompted me to put a sticker on and get a consent form and think about that.

HP3

R: I didn't really have any expectations, to be honest. I think because I didn't really fully understand... I didn't understand what was going to happen. All I knew was...because (name) said it will just make things a lot more efficient. I remember at one of the meetings that I went to some of the other pharmacists weren't really happy about the whole e-referral situation. I think for them... But then I think it was mainly the senior pharmacists that weren't happy about it. I'm not sure if it's because they're so used to the system that we had before, but I know one of them said it's because the pharmacies will now be aware of why the patient's in and what's happened and it's too much information. But I feel like the more information the better, to be honest.

I: Why do you think that?

R: I feel like it's just better for them to understand why this patient's been in. If they're left in the dark, it will just be... I don't know what the word is. I think them having more information will help them with their work as much as it helps with us knowing fully what's happening in the community for the patient. So it will help them understand, okay, this patient obviously hasn't been adherent with their medication and maybe we can do something better to help them be more compliant, I guess. Because, say, it's a case of their blood sugars are ridiculously high because they haven't been taking any of their medication, if the pharmacy's not aware of that they're just going to keep doing the same thing because they think nothing's wrong.

I: Yes, precisely.

R: Or they could, I guess, maybe have more consultations with the patient, make them understand this is why it's important to take your medication. I think it's just a much better handover and transfer of care for the pharmacy to be aware of what's going on in secondary care

HP7

R: There is a link so you just click it, you type in – I always type in, the postcode of the pharmacy, because if you type in the name it's not going to map it to the map, so it's better to just type the name and the postcode, find it on the map. And, then I'll send it. Sometimes if the patients don't bring in their blister packs, and I don't know what time slots things go in, I might have to ring the pharmacy, or I will ask the family to bring the blister pack in.

HP8

I: I think in some respects, until I was talking to you today, I have been saying to people, this is very linear. This is very, sort of, one way. It's very, sort of, like, hospital - community pharmacy communication. Where's the communication back from that. But actually, your bit is, sort of, it's a little hook at the end of it, isn't it, coming back to practice. Do you think there should be more of sort of a rounded communication going on?

R: Oh, definitely, yeah. I think it's part of integrated care that everyone should talk to each other about the patient, if possible, rather than just say, right, I've done my bit, that's it. You know, I'll wash my hands of the patient now, and they're on to the next thing. Because a lot of the patients that we are seeing have these chronic long-term conditions and they are probably going to be back in hospital and they're probably going to be going to their community pharmacy for a very long period of time. You know, it's not like healthy people who might have a prescription for a month and then, you know.

HP8

I: I suppose, with some of these patients it's going to be... There are people who will have their medicines delivered and they won't actually go into the...

R: Go into pharmacy, yeah.

I: And that could be... Is there therefore something, is that the role the NIPPS could pick up in terms of...?

R: Yeah. I mean, we do, do home visits for patients who are house bound and unable to come out to the GP. And certainly, providing advice on long-term chronic conditions, it's not just about medicines. So we ask for, using the COPD example, we'll refer patients to pulmonary rehab and smoking cessation and all those, sort of, lifestyle things that will help and try and reduce that medicines burden as much as possible.

HP9

R: Knowing more about their medicines. I mean, I don't know if the chemist…I think they do call the patient and do speak to them as well when they get discharged, just like to make sure that they've got enough supply. I don't know about informing them about their medications but they could potentially have a role in this because, like I say, if not all the discharge…when the discharges come to me I call the patient, go through their medications but if they don't come to me and go to a GP they obviously won't have that conversation.

I: So if you get a dis…when you get a discharge note you generally call the patient?

R: I generally call patients unless there's a reason that I can't get in touch with them or they…I'll just confuse matters if I call them if they've got dementia or something like that and then I'd speak to their carers but that might not always be possible as well. They might have carers coming four times…

I: So that call you're making is to check that they've got what they need?

R: That they've got what they need, but also I go through the list and make sure that they're aware what's changed and what's new, what's stopped and usually give them some advice about any new tablets, if they get started on a lot of medications, I give them advice about it as well, what to look out for. And if they've got any monitoring I make sure that they know about it as well and that they need to book in.

I: Yeah, 'cause if they've been put on a tablet that needs monitoring, yeah.

R: So that bit I do but obviously GPs don't have that conversation with a patient because they do the discharges and they've got obviously clinics and things at the same time.

I: Yeah, and you've got more time.

R: Yeah, I've got more time, so they will just do the…look at the discharge and process it and do send a task to the admin and say, book them in for bloods, or whatever or book them in for a review, but then nobody would have that conversation then with a patient, so you'd be relying on the hospital pharmacist that they might have told them about the changes. They're relying on the nurse as well I guess, telling them what's changed. But, I can guarantee you because I've come across a lot of patients and they've just not had that conversation at all or they might have just briefly mentioned it but when I speak to them they're like, no, nobody's told us anything about this, so I have to go through it.

P1_C1

I: So, when the blister pack, did that come from the local chemist?

R2: It comes from; it's a dispensing chemist, which is part of (name of community pharmacy).

I: Right, okay.

R2: And, then it gets sent to the local chemist, and then it's collected and then brought through.

I: Right. So, was there any conversation with the local chemist about…?

R2: No, because the main carers pick them up but even when I've picked them up there is no, it's just I go in and I say prescription for…and then they say, right, and that's it, there is no conversation.

I: No conversation. Have you ever had any conversation with, you know, a pharmacist or a chemist about your medicine.

R1: No, never

P1_C1

I: But presumably, when they have been prescribed, GPs or the hospital consultant has talked to you about why they are being prescribed?

R1: Yeah.

I: Have you had any information from them about…

R2: The GP goes through them, we do a review probably once a year, but again there is different types of medicine that do the same thing, they are for the same treatment.

I: Right, yes.

R2: But they have different ways of working.

I: Yeah.

R2: With things, so, especially with the gastro. You know, you have the omeprazole, which is, I can never say that properly. Then we've got the ranitidine which is basically, they are both gastro tablets.

I: Yes.

R2: One works with the acid, the other one negates it.

I: Right.

R2: But the one that works with the acid causes issues with salt levels. If it's not monitored. But we've only found that out recently.

P1_C1

I: Which is really interesting from our point of view. Because actually this is, what they are supposed to avoid isn't it. But, yeah, that's really interesting. So, how was that explained and who explained to you about those changes in the hospital?

R2: Well this was more to do with the consultants.

I: Right.

R2: And, they were looking at mum's overall health, and they were saying because they need to get the fluid down, but they still needed to use a diuretic to reduce the fluid. That was how it came about, that there should be regular checks with a certain type of tablet.

I: Yes.

R2: So that the salt levels are monitored. And, even though that mum goes and has blood tests done every year, that didn't show up.

I: Yeah.

R2: So, it must have just been a build-up over time.

P1_C1

R1: But the GP, Dr (name) she explains everything. She is really good.

R2: Well she does and she doesn't. She doesn't explain what the tablets actually do.

I: All do.

R2: And what interacts with them.

I: Yeah.
R2: She's good in the fact that she's aware of what mum's conditions are and why the tablets have been given.
I: So, basically you would like, more information would be better?
R2: Yeah. Even with the venapack, even if, obviously if it was going to be regularly changed, then that would defeat the object, but if you've got regular doses and you are on them for however many, even for 12 months, if one piece of information, like a piece of paper of the all the drugs that's on and what the interactions would be, what to avoid, what not to avoid, if you got that information, with the pack, just the once, so you've got them here. And then when it's changed, that information changes with it.

P2
I: No one at all? Did anyone at the hospital talk you through what your medicines were for?
R: No, it was just changed, and they just kept saying, oh we'll change this and change the dosage and see if it works, but if it isn't a heart problem, why are they treating me for a heart problem? You know with the medication.
I: Right, so you think they've given you medicines for your heart?
R: Yeah.
I: But they've also said to you, that there isn't a problem with your heart?
R: Yeah, you know, I mean, with all them papers, me daughter's read them, and they're sort of saying, well it's not your heart. But how can your heart rate go so high and then stop and that's it, and they say that it is your heart rate but then, now, with all them papers they're saying it isn't the heart but yet they're still treating me with the medication.

P2
R: Because I wanted to check, and there is a change, only in one, but there still is a change and I've not been told about it.
I: Right, right.
R: So, I know you said, well we'll drop the dosage of such thing and we'll higher the dosage, so its alright saying, oh well higher the dosage and then lower the dosage, but … why? And of course, they talk amongst themselves and they talk, oh well we'll do this, and we'll do that, alright I understand that it's a teaching hospital and they have to know, but I have to know also.

P2
R: Yeah, and then they just keep…when I go and I've got a problem, oh we'll just change it.
I: But no one explains to you why?
R: And nobody explains why they're changing it, so of course they just change it and they don't have the time to sit down and talk to you, this is the problem. You know, you go and oh it's if you go to your GP, they haven't got time to discuss everything, so of course, I'm on a lot of medication but I don't know what it's for.

P2
I: That's interesting because…I mean in what way would you like to be told about that? Would you…who do you…or firstly, how do you think it would help you to know what you're on? Do you think it would help you to know why you're on those medicines?
R: Well I'd like, yeah…I mean me, daughter's more taken control because I forget so much, and this is the problem and I do, you know, because she said to me write it down mum, but I find that at the hospital and the GP, they're just changing it, and of course they haven't got time to explain everything to you because you're only allowed so many minutes with your GP and at the hospital all they do, is just discuss it amongst themselves, and they don't discuss it with the individual.

P2
I: You said when you went to (name of hospital) recently, this last visit just, you were discharged well, 9 days ago, or a week ago, you came out of (name of hospital) last time? Was it last week you came out?
R: I went in, yeah.
I: Yeah, so you said that some of the medicines were changed then, do you know who made those changes, who decided on those changes?
R: The doctor at the hospital.
I: The doctor, yeah. And do you know…but you don't know why those changes were made?
R: No. Oh well that dosage isn't right.
I: Ah right, so they told you the dosage wasn't right.
R: So, we'll try a new dosage and see how you go. You know, although, but they don't actually speak to you, and face to face they don't speak to you, they speak to the medical team and, oh well, we'll change that to such a thing, but they don't actually speak to you face to face. And to me that's important, you know, but they don't and of course I think, oh well, they know what they're talking about. I think, well they should know what they're talking about, but do they? They're just changing it to see if it works, and that's all. They're not sure whether it will work.

P2
I: One of the things we've talked about a lot, and I'm going to wind up in a minute because I think we're coming up to half an hour or so, is about the fact that no one has talked to you about these medicines, which is really interesting. Would you like an explanation from people, who would you like to talk to? Who do you think would be the right person to talk to about them, your GP or the doctors at the hospital or who? Who do you think would be best placed? Or the chemist?
R: Maybe the chemist, like I understand the specialists at the hospital don't have the time, you see and that's what me daughter wants.
I: But now you're with the, I think from what you're saying, you're with the chemist, where they've got one chemist that deals with all the blister packs.
R: Maybe the chemist is the best to talk to.
I: But there's not…you haven't got your local…there's not one just around the corner is there, where you walk in and you go actually have a chat with them. Do you think having a chat with the chemist would be helpful, to go through what those medicines are?
R: Yeah, but you see me daughter, she would have liked to have sorted it before I came out of hospital, but it was just brushed under the carpet.
I: Yeah.
R: You know, and she personally asked, and the doctor said, yes, we'll sit down with your mum and that never happened.

I:	Never happened.
R:	You know, and it didn't happen.

P3
R:	It's not the first time. I mean, (name of pharmacy), went into a new system on their computers and I went in several times and asked for the blister pack, oh, it'll be another half an hour, another 20 minutes, and this system, every time my doctor sent the prescription and everything and (name of pharmacy), wanted to take it off, it crashed, it crashed their system, so that took a few days to sort out. I don't know who the fault was with, you know, but I did hear the girl say in the chemist, I'm not getting off this phone till you sort your end out, you know, so… But what's happened this time I don't know – well, as I say, they didn't get the discharge papers…
I:	Yeah, which is what they should be getting.
R:	Yeah, yeah, well (name of pharmacy) have still had nothing, because I promised to take a copy in of mine. So that's a bit of a fall-down in the, you know. Plus it's a bit of a let-down at (name of pharmacy) because even though they've got the new meds from the doctors, they didn't put them into the pack…

P3
I:	When you went into (name of hospital) recently, who talked to you about those changes of the medicines?
R:	It was one of the ward doctors, see, I went into A&E, went into the EAU, and from the EAU I went into the heart unit…
I:	Oh right, so you got moved about a fair…
R:	Yeah, yeah. So I got a bit conflicted information, there was one doctor said that – I don't know if he said artefacts on the ECG [voices overlap 19:53]. He said, we noticed something on the right side of your heart which is normally related to people who have lung problems. So I thought, oh well, perhaps we're getting to the breathing side of it. So he said, we're going to do a CT scan to see if there's any clots in your lungs. And then somebody came in and said, we've booked you in for an echocardiogram, so I said, oh right, I said, I have had one of them before a few years ago. Well, we want to see what's going on. So I said, well, I have had the MRI, so this doctor, oh, he said, I'll see if I can get the pictures off that, he said, it might be better than the echo. So the next thing, another consultant comes in the next day and he's got trainees with him and he's got like a big TV on a stand that they're moving around, there's another chap with him, and he's talking to the students about my condition, then he turns to me and he said, we're going to do this scan on your lungs, so he said to see if there's any clots. So I said, well, they did a blood test in the A&E for that to see if there's any blood clots and he said there wasn't any. Oh, don't worry about that, he said, we'll do it this way – it came back clear, all the X-rays I've had have come back clear, you know. And I mentioned the echo and he said, you don't need that, so I said, oh, fine, no problem, he said, you can't get better than the MRI, said, fair enough, I'm not bothered – I got a letter yesterday for my echo.

P3
I:	So in terms of anyone explaining the medicines to you, when you said they made the changes, did anyone sort of say to you why they're making the changes and stuff like that?
R:	They did to an extent, one doctor said, we're stopping all your Ranexa, so I said, fine, he said, and we're giving you this one, I think it was the Amlodipine, that one.
I:	Do you know what that's for, that?
R:	I believe it's got two purposes, I think it's angina protection and it also helps with blood pressure.
I:	Right, okay, yeah. And they told you that?
R:	Yeah, yeah. And he said, I'm reducing your statin by half, he said, because that can interfere with the Amlodipine I think if it's too high.
I:	Which is now why they're doing this half tablet thing.
R:	Yeah. So I know now what I'm going to be taking because I've got the hospital's which is why I kept that blister pack when it was finished, it's just a matter now of sorting it out. You get explanations now and again about certain meds. The hospital stopped two blood pressure tablets in May. They looked at that being one of the reasons for the headaches, because my blood pressure went right up. So what they did, one of them, the Candesartan, they reintroduced it at a quarter of what I'd had initially, I had 32 mg, so they brought it back in steps of 8 mg until we were up to 32. Whether it's that and the angina tablet working together, my blood pressure has been spot on at the hospital. They do try and explain sometimes, but I forget, my memory is not at its best, so…

P3
I:	So you've done a phone call with…
R:	It's a phone call, yeah.
I:	With the pharmacist there.
R:	With the pharmacist, and all he said, he's just picked odd ones out, how you doing with this and how's that, and then he stopped the antibiotic I was on, so three days later I had to ring him back and tell him that I'd got a water infection again. So the pharmacist said, well, you've been on that tablet six months, we have to stop it and see how you went on, I said, well now you know.

P3
R:	You know what I mean, so the hospital said put me on it permanently. But no, in (name of pharmacy) I've had a review from their pharmacist, yeah, they've done a review.
I:	Is that recent or…?
R:	It was earlier this year, yeah, she said…giving me my tablets and that, then she said, well, you can just come in this room and have a chat or…yeah, no problem, you know, like I said, I'm retired, the time is my own, so yeah, I've had review and they go through how you coping with this and how you coping with that, and if you ask, they'll tell you what they are, you know.
I:	And do you find that useful…
R:	Yeah.
I:	…and the one over the phone as well with the guy at the surgery?
R:	I prefer face-to-face, but you can't get an appointment with our pharmacist at the surgery, you can have a phone call but you can't come in and talk to them. And if you ask the doctors what's that tablet for then they'll tell you, you know, but they don't just sit down and tell you what, you know, they're going to put you on this now and that should help with so-and-so. But then as I say, the two blood pressure tablets I was on that they stopped, the consultant in the EAU that time said, you've medication here that's working against each other, he said, and you've also got some that's working too well.

P3
I: Apart from (name of pharmacist), did any of the pharmacists at the hospital come and talk to you at all at any stage apart from (name of pharmacist) about it?
R: Yeah, they usually come up on the day before you're being discharged, yeah, and they'll tell you what you, you know, we'll have your blister pack ready, any changes that the doctors have made will be included in there, and as I say, they've put a list of all the tablets on the blister pack, so you know, what they all are, but, you know. There's a lot of information given now from the pharmacy. And as I say, it's always whatever they say it's going to be, whatever is going to be in it is in it, you know, it's pretty.

P4_C4_C5
I: Yeah, so when the pharmacist came out to the house, how did that help, or did that help?
R2: No, he was to go to the doctor's to be assessed by the pharmacist. He couldn't walk, he couldn't stand up, his legs were bad, so I phoned the doctor and I said, can they not come to house? Everyone comes to the house for him, except the doctor.
I: Yes, except the doctor. But on this occasion, the pharmacist did come out, yeah?
R1: The pharmacist, yeah.
R2: The pharmacist did come and he's supposed to go for a medical check-up, 12 months, and he's in and out of hospital, where the check up he has to do, I don't know.

P4_C4_C5
R2: Yeah, why they'd changed them, yeah.
I: Why the things have been changed?
R2: Yeah. The reason for it, yeah.
I: And the reasons for that, good.
R2: Yeah, she said the reasons, and why they'd changed them.
I: And do you think, sort of there and generally, and I know the way this might go, do you think you get enough information about your medicines generally, from people?
R3: I think what can happen sometimes, when the doctor does his rounds, I think they might sit and explain everything to my dad, but my dad…
R2: But he doesn't take it in.
R3: …at this moment in time, especially when he's got a fever, or when he's on his medication and that, his memory is not the very best, and he does not relay that information very well.
R2: He doesn't want to know, [inaudible 00:20:39], he just doesn't want to know.
R3: No, he doesn't retain the information.
R2: Doesn't register.

P4_C4_C5
R1: They did do that once, (name of community pharmacists) did it once, and that was…
R2: Yeah, but (name of practice pharmacist), he wrote it out for me, and sent me a letter.
R1: Yeah.
R2: 'Cause I asked for it.
I: He's the pharmacist at the practice?
R2: Yeah.
R3: If you went and asked (name of community pharmacist), she'd sit down and tell you as well, wouldn't she?

P4_C4_C5
I: Yeah, the chemist was going to know that you'd been in hospital and so on. So, was it just (name of hospital pharmacist) that talked to you about that, or did anyone else talk to you about that?
R2: Yeah, no, just (name of hospital pharmacist) wasn't it?
R3: On that occasion, it was just (name of hospital pharmacist). We have had occasions where other doctors, in the past, have mentioned changing medication, and what they're changing for. It depends on what ward you were on, and what doctor's there at the time.
I: Right, yes.
R3: When you get discharged, if he ever goes to the discharge lounge, they will sit there and go through the discharge notes with you won't they?
R2: Yeah.
R3: And go through the medication there, but they do say…
R2: But they won't tell you what it's for though.
R3: It depends on, I've been sat there with my dad in the discharge lounge, and sometimes the nurses will tell you what they're for, other times, again it's dependant on the member of staff who's dealing with you at the time.
I: Yeah, quite, yeah.
R3: You get some that'll just, you're out the door, and you get others that will sit and go through everything. It's a lottery.

P4_C4_C5
I: And on each of those occasions, when you come out, and medicines have then been changed, so it has been, you say there's been lots, yeah?
R1: They might just change one tablet, they might change three or four.
R2: Or they'll take one out and put something else in.
I: Put something else in, yeah. So, there's been thing that have, so in terms of over the last six months, there's been a lot of chopping and changing [inaudible 00:34:50]?
R2: Yeah.
R1: Yeah. They've stood at the side of the bed, and you've had the, it could have been the registrar, but it was the chief of the team, he'd be there, and then there's, the registrar would be there for instance, and they'd have a chat between them, and they'd say, yeah, well we'll get rid of that. Okay then, (name of patient), you know, see you later. And go away.

I: Yeah.
R1: Now, I know they've changed two, maybe three tablets.

P4_C4_C5
I: Yeah.
R3: He's bad on his feet, he's also not in the best of health.
R2: He can't see proper.
R3: And it's like, what you're doing to him, what are you doing to his body? If you change it, change it, but keep up with the… 'cause there was one time, where he had to lose weight, and he was told to be seen in four weeks' time.
R1: Oh yeah, that's right.
R3: That was ridiculous. So he's trying to lose weight, he couldn't even get an appointment for four weeks' time, then they changed his doctor and he was under two consultants, and they're contradicting each other, and you just don't know…
R2: What's the best.
R1: One was about the weight wasn't he?
R2: Yeah.
R3: Yeah.
R1: Get rid of your weight, and the other one said…
R2: That's when they upped your water tablets.
R3: Who's right and who's wrong? Do you take that tablet, or do you take that tablet?
R2: Antibiotics - have a lot of fluid and drink a lot of water. Don't drink a lot of water with his legs.

Theme - 3c Infrastructures - existing, adapted and new

3c 1 Code - Changes in technology and technologies

HP1
I: Yes. So, thinking about that, and I mean, the way pharmacists are working in general practice now, I'm trying to see how, in what ways, knowing that discharge information, having that three way link, how would that have actually improved the work that you're doing in practice, or helped the work you're doing in practice?
R: I mean currently, it's difficult to generalise, because the practices don't always have that priority on certain work, so they might be doing something else, like asthma clinics, but if a pharmacist was working on Docman, where all…do you know what Docman is, so all the…? So, all the outpatient letters and discharged and queries, all come through Docman. So, if I was in charge of going through Docman for that practice, then we look at the discharge summaries anyway, but it's by date order, so as it comes, we look. Now, if we had access to PharmOutcomes, we could then prioritise those discharges straight away, and look at those and also the queries would be highlighted, so we would know to look at those queries, sort them out first. And I think it would then be easier, because then you'd go on Docman and say, oh that's been actioned, that's been actioned, and also you're prioritising what needs to be done, you're spotting it, sometimes we do miss things, so hopefully if there was something that needed looking at, that was highlighted on PharmOutcomes, and there was another point…I don't know if I've answered your question?

HP1
I: Yeah, that's really interesting actually, getting some understanding of how that triangle might have worked, is really helpful. Just going back a little bit. So, what you see is this notification by email, can you describe how it's used, what happens in you work with it?
R: Okay, so I hate the notification, 'cause the first time I had it, and I'm quite computer savvy, my mum was a computer…anyway, we used to do everything on computer from the day we were born, so I know how to use computers. But, when I looked at it, I thought, oh God, what the hell is this? So, what it is, is they send you an Excel, I'm trying to open it, but it's not really going to be of benefit, so I can describe it, so they send you an Excel spreadsheet, with…this has actually been, so I've actually changed it so you can see the words, but initially when I got it, there were just codes on the Excel spreadsheet. So, you have to then change the format to work like Word, so text, and then you'd see it. So it's quite simple but someone that doesn't really know, they'd kind of be looking at it for about ten minutes, thinking, what is this, I don't really know what this is? So, I think that's now been fixed, because I raised it with (name), and I think (name) now will change it, before she sends it out. And that was my initial experience. The other thing was, there's no NHS number, so what happens in general practice is, I receive this email via my NHS email, and then I then, there is a column that said, where I eventually found, there is a column that says what is the issue. And so, for example, the first one I got, the issue was medicines stopped in hospital, still on repeat. I don't actually know what the medicines were, that's it.
I: It's not told you which of them, or all of them?
R: Not told me which, yeah, but I just guessed that another reason, going back to having training, it would be nice to actually see how it looks on the hospital side, so we have an idea what that has come from. Was it actually someone free texting it, or was it just a tick box?
I: Yes, a drop tick box.
R: If it was a drop tick box, then it would make me think, okay, it might just be a general thing, it might not even be an issue. So, that just helps with our thoughts. So, I knew that there was an issue, but I didn't actually know, there was only a patient ID. Now, in EMIS, and Vision, you can't get the patient's records via their hospital ID, that's only for the hospital system. So, what I had to do, was log on to Sunrise, now you wouldn't appreciate it if you haven't done it, it is easier now, but some practices still have some block on the computer, so sometimes it's difficult to log onto sunrise from a practice, which is the hospital EPR. So, what I had to do, was log on to sunrise, type in the hospital ID, find the patient, go onto the patient information to find out what the NHS number is, so I've got the NHS number, go back to the practice system, find out who…and this is just to find out who the patient is.
I: Yeah, before you do anything else.
R: So, the first day I got this, I couldn't log onto EPR, which is sods law but they had updated the system at the practice, and I couldn't log on to EPR. So, it was very stressful for me, because I knew that something wasn't right, because we were issuing something on the repeat, but it had been stopped in hospital. I didn't know whether it had just been stopped, or is this like, has the patient been having it for about two weeks? And I didn't know any other information. I didn't know who the patient was. I didn't know what to do. Didn't know what pharmacy it was, because there's no information about who the pharmacy is, which pharmacy it is, so I had to guess and just called, yeah, there's no information on what pharmacy it's come from. So, I didn't like it. And, I did speak to (name) about it, and said, why isn't the NHS number in there, just so it's fast? And she said, that the way that it's sent via the PharmOutcomes, is they can't generate the NHS number, so it can only be this way for some

reason, I don't know. So, yeah, in fact, it had got a pharmacy number, (name of pharmacy), but there's hundreds of (name of pharmacy), pharmacies in (place), so I didn't know which, so I just called the one next door, and luckily it was them. So, that's how it looks like in practice.

HP1

I: And also, then, having to do further things, as you…I mean, do you think, what's the sort of, how's this going to move forward then, in terms of, is this going to stay the same like this, or is this going to be, do you think this'll be…?

R: I mean, I think that there's a lot of things that don't need to be in this report that we get sent. I mean, this is assuming that we're not going to be in the e-referral system, and it's going to be a two-way, if it was a three-way, it would be a different story, 'cause we could actually see the system, and yeah, but currently, as it stands, we're just getting these reports as and when we need to. And the reports are really, there's information that we don't really need, like DISWA B3, ah, that's probably Discharge Ward.

I: Discharge Ward, yeah.

R: I mean, yeah, that's useful, because if we wanted to call the ward, and we know where they got discharged, yeah. But there are things that, I think there's information that we don't really need, and the information that we do need, probably needs highlighting more. We also definitely need an NHS number, or an easier way to get to know who the patient is, because not everyone can access EPR unfortunately, which I think they should be able to, but even if they can access it, it takes time. And the way that we access EPR in general practice, we have to log on via and then we have to go on to our hospital system, and then we have to select Sunrise, and then we have to log onto Sunrise, so already there's three log ons, yeah, just to get onto the system, so it's not really that easy to do, yeah, and it takes time.

HP1

I: Yeah. So, a lot of pharmacists would be in the position anyway, it wouldn't be an unfamiliar system to…presumably. So, if you had that three, if you were seeing as the discharge or e-referral went to the community pharmacist, if it came to the practice pharmacist at the same time, how do you think that would be of benefit then?

R: I mean, it depends on what needs changing. If it was something to change on a repeat, we'd just do it there and then. I think also, the issue is that the community pharmacists, there's been two occasions where they've said something, and actually it didn't need reporting, because it would have been removed anyway. So, if it was a, they had a repeat, and they said, it's still on the repeat, it was still on the repeat because that's an old prescription, an old repeat. So, sometimes I think that the selection of boxes, they also need to understand what that would look like for us, so I think the importance of the community pharmacy selecting the right boxes, and the hospital pharmacy selecting the right boxes, because if they select the wrong box, it just means something different to us. So, for example, I'll try to make sense of what I've just said, if it was about, like if we take this example, so, medicines stopped in hospital, still on repeat. This is what the pharmacy had told us. And again, it's difficult, 'cause I haven't seen the system, I don't even know how they do it. But, do they know that that is actually the warning for us, the issue that's been highlighted for us to action, or are they just ticking this, assuming that it's just information, just saying that the hospital's stopped this, and it's on repeat, but it's fine, it's nothing we want anyone to do anything about it, we're just stating a fact. But I don't think that they realise that that, to us, is, okay, it's stopped in hospital, but it's still on repeat, there's a problem, why is it still on repeat? But then going back to the community pharmacy, when I called them, they said, oh it's fine, it's just that it had been stopped, but I don't think they realised that that was something that's been highlighted…

I: It's about the clarity of that information as well, isn't it, coming to you?

HP1

I: It's interesting actually, because I think, you know, a lot of these service…ah, well, we're going to, there's going to be fewer adverse events, there's going to be fewer readmissions to hospital, in the worst case scenario there probably is, someone's discharged on certain medicines, and then gets an adverse event on those new meds, and is back in hospital, which would be worst case scenario wouldn't it? But it's more around the working side of things, you think?

R: Yeah, and also, this system works, I think, for only some practices, because some practices haven't got to that stage yet, of being quite computer savvy and looking at things in a timely manner, and have they even got a person to look at this in their practice? So, I think also, we shouldn't generalise that this system is great across the neighbourhood, because my practices, for example, are good in that they're big, so they have lots of staff, they've got a staff to do recalls, they've got a staff to look at sending texts out to patients, staff looking at Docman, staff looking at discharges. Other practices don't have that, they don't have anyone, so are they going to be looking at this, and who's going to be actioning it, and have they actioned it properly?

HP10

I: Just before we move forward, your role, you're a community pharmacist, yeah?

R: Yeah, I am. I'm a community pharmacist and my role is... Overall, my label is I'm pharmacy manager as well, so I manage the staff as well as manage the pharmacy. If you want some more information about the pharmacy, of the roles, it's about 11... In the pharmacy, we judge how big the pharmacy is by the numbers of items we will dispense in a month, so we do around 11,000, which I think is pretty big. We've got five members of staff, three full-timers, two part-timers, and we do a wide range of services, a wide range of things. We've got about...which is related to our interview, our chat, I've got about 100 patients on blister packs, so this is quite a bit. So one full-timer is completely dedicated to do this task and others sometimes will help with the admin of it. It's a huge task. We've calculated it takes more than 40 hours of work to run it. There are a number of issues with it but, you know, that's pretty much the description of my pharmacy in relation to blister packs and to overall.

HP11

R: Just what we expected to happen, really. Except actually I think the very first one…the one of the first few – I can't remember if it was the very first one – it wasn't our patient. It was from MDS, which is our other unit.

I: Oh, yes. Yes.

R: So I think I had to send it back. And then I think I tried to phone and say, mm, what do I do about this, and…? But yeah, I think I spoke to (name), then actually, who was really helpful.

I: Yeah, because you've got a unit that deals with the MDSs, is that right?

R: Yes. Well, they h…

I: Some of them.

R: At the moment they've got a robot which I think is going, but yeah. That's all they do, basically.

I: Yeah, but you still do…deal with Venalinks and stuff here as well, do you?

R: Yeah, see, at one time we were sending out some of our Venalinks, not all of them. It's complicated because they can't put everything…their robot doesn't have all the drugs, so we were sending some of our simple ones to them, but it's a real faff because we had to get the scripts, process the scripts, encrypt it all, send it up there and then they would send it back and then we still had to check it and it was just…

I: You might as well just be doing them, yeah.

R: So because of IT issues, we got a new IT system and we couldn't do that while we were using the robot, so we used that as a…that's fine, we'll just do our own and I think it was a good decision. So yeah, so there was a bit of messing about where we were getting MDSs but that very rarely happens now.

HP11

R: Well, it's stuff we would have been doing anyway really. I suppose what's really good is you could go on and check…if you wanted to reprint it, you could, couldn't you? Whereas if we had a…it's there, isn't it?

I: It's there. It's in that system.

R: Rather than just a…

I: That's interesting, yeah.

R: …rather than just a piece of paper that we might have taken next door and forgotten to copy it, we could just go back on and look at that.

HP12

R: We have now got the pharmalarm as well.

I: Yes. Talk me through those actually, because I've heard about this.

R: Yeah, so it's like a little plug in alarm thing that basically changes colour when we get any messages or any notifications. So, you still get the email as well. So, sometimes it will change and start flashing blue when we haven't even noticed and we just see the email first. It's just like a little widget that changes colour and flashes.

I: When you have got a message?

R: Yes, so it lights up lighter initially if we've got any discharge referrals it will flash blue.

I: That's cool.

R: Other times we don't notice it until we go on the emails and notice that we've got an email. We are getting better at noticing it.

I: So you get an email to say there is a message, you now get a pharmalarm.

R: So we get the pharm alarm as well to say we've got a message so we can go on and see the messages. And, then obviously click on the message and action it according to what we need to do

HP13

I: And it's there within. And you've now got the PharmAlarms, haven't you?

R: Not everybody, no, not everybody. Only people that went to the recent meeting got one, I'd got one and that's even better, that's fantastic because we did open PharmOutcomes every day so we did know if people were in hospital but we know more instantly now.It's just plugged, it's at eye level, got it plugged in to an eye level usb so we immediately see, all the staff know if it's orange that means we've got something, get PharmOutcomes up. Well, actually you can go in…there's an app anyway where you can go directly into PharmOutcomes, once you see it's orange it takes you to your login page. It's all very handy.

I: Yes, and then just log in and see.

R: Yeah, 'cause we could just be doing that person's prescription, for all we know, and then the light comes on - excellent. Whereas before the PharmAlarm, it did rely on us opening PharmOutcomes, which, as I say, we did but only in the morning and maybe again in the evening. If they went in at four o'clock in the afternoon we wouldn't know maybe 'til the next day or something.

I: Yeah, precisely.

R: This PharmAlarm is good, it's very good, yeah.

HP13

R: Well, the first one wasn't very well attended, to be fair. I think there was only about me and two or three other people there. The second was a bit better attended but they're very good events, they were run well, I just thought it was rather disappointing that not many people were…

I: From community pharmacy.

R: Engaging with it, yeah, you know. But I didn't like the faxing that they did before, anyway. I didn't like all these phone calls and faxes. I'm very technological-oriented, so this appealed to me, that's why I went, you know, 'cause it appealed to me. And it's even better than I thought because I like this feedback thing that I've got to do, this thing about going through logically what I've done.

I: A checklist….

R: Yeah, the checklist, yeah and that's there as a formal record, anyway, of what you really did do.

HP13

I: And is there a cost saving on waste or stuff like that involved as well?

R: Well, there would be because in the past if the GP did the wrong prescription, you'd take it to them and then you might still have had to get, the patient wouldn't have had to be using that one. You'd have to go along a day later with the correct thing 'cause somebody realised somewhere, you know. Yeah, there would be a saving and also the fact that because of the quick notification, it saves us making a pack up. So that prescription can just be returned to the [inaudible 28:14] or something, it can be… It means you're saving, you're not even having to dispense that prescription. And if it's incorrect, you just destroy it, it's not a waste at all.

HP15

I: Yeah. I mean that's actually one of the things…we're moving through these questions, is one of the things that basically, how do you actually use the e-referral as part of your service. Could you perhaps talk me through what happens from the moment you get that notification that someone is the hospital? What do you actually then do?

R: Okay. So, once we receive it, we have to open it and accept the referral and complete it. So basically, it's just to say that, yeah, we have received it. And then we mark our PMR system to say that the patient is in hospital, so we don't dispense any medication while they are in the hospital or even delete their retrieval request. So, we don't send the request, yeah. And so, every time someone would go in their record, it will tell you the patient is in hospital. So, we don't do anything with them, yeah. So, until we receive another discharge letter or something, information from the online referral, then we'll accept that and complete whatever it says.

I: Have you got the little PharmAlarm with the PharmOutcomes?
R: Yes, we have. But we've just misplaced ours because we had a computer before here. So, when we had our new computer setup, we've misplaced it. So, they are sending a new one out to us. We just had a call today about it yet. Yeah.
I: Right. Brill.
R: Yeah, we were using it until three weeks before, but we misplaced when everything moved about.
I: Yeah. Alright. When you had…so you had a different computer in here. So, you've updated and the PharmAlarm has gone missing. But did you find the PharmAlarm useful then?
R: Yes, it was useful, yeah.

HP15
R: It helps us a lot the way we work here.
I: In what sort of ways?
R: Because before we used to have to be careful that we don't lose the discharge. Because it was all printed, we just have one copy, that's it, yeah. Unless yeah…even if you do a double copy, but if you're busy and we don't have…for instance, when our dispenser is finished and it is just me here, then if it's too busy, she might have put it somewhere else, and I don't know where she has put it. Or it might have been destroyed.[Interruption to the interview 23:48-23:59].
I: Yeah. Sorry. So yeah. So that's helped the way you are working there.
R: So, before when it was just one copy, it might have been put somewhere else, or it might have been destroyed by mistake, so we've lost the copy. But now, at least we've got it electronic, so we can reprint it if we need it.

HP16
R: So I think for me it's slightly different because I was involved in it from very, very early on, so we've been scoping this for a couple of years now but the issue was getting the information and the IT bit behind. Now being involving with some of seamless care work we know that (name of place) opted to do the test bed for (name of metropolitan area) , that meant the process had to be quite rigorous because they would need to make sure that it suits any trust's system potentially, it is interoperable with what other people would need from very, very basic paper records all the way up to what (name of place) have, which is an integrated system. So it's that kind of mix so that's where some of the delays came in so I've known about it for quite a while.

HP2
R: I think you've got peace of mind knowing once that discharge summary has been written and is sent, you know, the chemist is going to…it's going to receive documentation. Because if we…we had failed in the pharmacy previously by faxing everything, then I think that would make me feel that was my fault.
I: Right. Right. Yeah.
R: Do you know what I mean? So, with e-referral you know it's going to get there.
I: So, it's more…a confidence.
R: So, it's more of a confidence, yeah, beforehand if there was…if somebody even forgot to put a cover note on the top of the prescription to say, please fax, that may at some stage not have gone to the chemist.
I: Right.
R: It may never have got there.
I: So, the previous system was one which involved paper and…
R: Paperwork and human error. Yeah.
I: And now it's just…
R: And now it's electronic it goes…[…] Click a button and it's gone.

HP3
R: So I think it took all of us a bit of time to fully understand what we're doing now. I think it started on a Monday, and so we did have our team huddle and (name) did explain again, 'guys, it's going live, make sure you write it down as the electronic referral.' So before, when we'd send down the blister packs and we'd have the fax sheet in front of it, we all knew that it was a blister pack, but now, (name) said there's no need for the fax sheet anymore, just write the letter e on top of it, saying that it's Venalink, and so that should be fine by itself. And with that she said you don't even have to put what goes in which slot, because usually, when we do the TTOs, we'd write on the top that it's a Venalink.
I: TTOs are the...?
R: The discharge medication.
I: Medication, yeah.
R: Yeah. And so on that we would write, this is a Venalink blister pack, so when they're dispensing it they know that...
I: They know how to put it and make it up.
R: Make it up. But (name), when we first started it, she said, oh, that's not needed anymore, because if you put the letter e, they should just know that this is an e-referral, so they should know what should go in the blister pack. But I think there was confusion, because there were some that went through and they were just putting them in boxes, in separate boxes, instead of actually putting them in the Venalink. So, I think there were some discharge meds that went through that weren't blister packs, which I think it just confused the dispensary staff, but then I don't know if they'd had...

HP3
R: Were a bit confused by what to know, what to put inside of the blister pack, or even if it was a blister pack. And so I think we had a meeting, it was like the next huddle, we just said, we'll just continue writing that it is a blister pack just to make everyone aware that it is.
I: Yeah, so that they were aware. So could that have been done better then, that introduction?
R: Slightly. I don't think it was terrible, though, but I think it could have been done slightly better, but I think for what it was, it was fine. I think we were just a bit slow getting used to using...like finding pharmacies... I think that was another problem, actually, doing the community pharmacy search. I think some people struggled finding where the pharmacies were, just because it shows up with the actual google maps, and so you have to pick which pharmacy it is and search it. I think you can either search by pharmacy name or the actual location, but with pharmacy name, because there are so many (name of pharmacy), it's like how are you meant to find which (name of pharmacy)?

HP3
R: Yeah, so what I usually do is I assume that...because what we have for patients, like GP records here, is the SIR record. I'm not sure if you know the SCR record.
I: Yeah.
R: So patients who come in that have a SIR record, I would just assume that they have a (place) pharmacy, whereas the patients that were out of area, I would just assume that they're not in the area, so it wouldn't be an e-referral.

HP3
I: And then you do your stuff, but then it just drops down... Oh, right, that's... So it just takes you...
R: Yeah.
I: ...via that. But if you answer yes to that question, it then just takes you to the system.
R: Because before, what we had to do was put it in as an order, so it's similar to the orders where the doctors do the prescriptions, so we'd have to put it in as a community pharmacy referral and then... Which, I mean, it's not much longer but it's just easier when you're doing the medicines reconciliation, just click yes, and then it will take you straight to it.
I: It takes you straight to it. And then what happens?
R: So then it will come up with the pharmacy search, you put in the pharmacy, and then, at the bottom, it's like a consent, saying, I have told the patient, they've consented, or, I was unable to confirm with the patient if they're okay with it, and then you just confirm and then it sends, so it's just like an automatic...
I: So it then sends that the patient's been admitted and what's happened?
R: No, I don't think it sends that. I think it just sends that the patient's currently an inpatient, so I don't think there's any information of why they're in...
I: There's no information, just that they're in.

HP3
I: If you were to make any changes to it, if someone came along, (name) or (name) or someone, and said, right, we think it's not going very well and we're going to give it to you to change it. What changes would you make? What different things would you do with it, or would you?
R: I don't know. I think maybe the map, I'm not a big fan of the map, but it's not a hindrance of anything. I think it's helpful that when you open it up to find the pharmacy, it goes straight to their home location, because it's assumed that most people's pharmacies are in the area where they live. But I think I'd probably just put it in as just an address, like I'd just find the address and then have it like a drop-down list and pick the pharmacy that I need.

HP4
R: It's an electronic document which pharmacists and technicians use to basically – it's like a stepwise document you kind of go through, and it's all about accurately documenting the drug history for a patient, so when they come in it says, which sources have you used, and it will be GP prescription, patient, patients on medicines for example, so you'd tick it through, you'd work your way through, it's, does the patient have any medicines with them. And then you document the drug history, so you write whatever they're on at home, and then it says, is this – I can't remember the exact kind of terminology, but it says something like, can you complete an e-referral, do they have a compliance aid basically, and if you tick yes, it automatically brings up the box for the e-referral, so you can click on that and it exports you to…
I: And then just takes you…
R: …yeah, takes you straight through to the e-referral, which is good because it means you're less likely to forget to do it.
I: Yes, so you haven't got to think, oh, that patient's got a Venalink…
R: No, so…
I: …I need to do…
R: …you did last week, but since this new kind of document has been introduced…
I: So this is a change from when it originally started then.
R: It is, yeah.
I: Has that change occurred because of the e-referral or is it just something separate?
R: I don't think so, it's been in the pipeline for a long time, there were lots of new things that the Trust were asking pharmacy to do or things that…the old document we used was kind of donkey's years old, so this new document incorporates a lot of kind of things we do now that weren't necessarily there 20 years ago when it was introduced, so it's just been modernised really to kind of work better for us. But part of that…
I: But because of that…
R: But because of that, they've been able to incorporate it, which is really good I think.
I: So until this week, that was happening, was that previously what you were doing to…?
R: So previously you'd kind of rely on the fact that you had to remember to do it, and I did nearly forget a couple of times, because it's easy to do when you've got a complicated drug history, kind of in and out of different documents, you know, looking around and documenting things, and you'd put like…you still have to record it in the significant event button that we use, we still put blister pack there or Venalink and the details of the chemist. So then you'd have to remember to go into the orders and type in community pharmacy referral, it's like a separate step that you'd do at the end…
I: Whereas now, it's an automatic dropdown, soon as you've said yes…
R: Yeah.
I: …they're on a Venalink…
R: Yeah, it takes you through to it, yeah, which is better because it reminds…it's almost like it's more reliable because it's a prompt really.
I: Yes, so there's less…
R: Yeah, less likely that you're going to forget to do it at the end, yeah.
I: So that's the point at which you alert the admission, is that right?
R: Yeah, so that's when we would submit the document, yeah – the e-referral, sorry, yeah, the order.
I: Yes. So the community pharmacist now knows their patient's in hospital.
R: Yeah.
I: And they now know not to make a blister pack if they were going to.
R: I don't know how it works from their side, I'll be honest, like so (name) has told us a little bit about…
I: Yeah, I think they'll get that notification…

R: Yeah, I think they kind of either accept or reject and if they reject it she gets notified so she can feed back to us that we've got the wrong chemist or whatever, however it kind of works.
I: Yeah, and I think they get chased up if they reject without reason…
R: Okay, oh right, okay.
I: …I think.
R: Yeah, so I've not seen it from the other side, but kind of…
I: No, no, I mean, and I haven't either yet, so yeah. So what happens then, so that's on admission…
R: Yeah, so that order stays on their current inpatient chart throughout their admission, then my understanding is on discharge basically that order will automatically ping off…
I: Right. So when you then…do you have to physically do anything?
R: You don't have to do anything – yeah, my understanding is once you've put that referral on, that electronic order, when they are discharged, that will automatically notify the community pharmacy that they've gone home and they'll get a copy of the discharge summary.
I: So you only ever go into the system once, really.
R: To the order, yes, to the referral. So on discharge it's a slightly different process, there are certain things we have to do on discharge, like in the guidelines they kind of set up with this, we have to annotate the discharge summary with documentation to say who's clinically checked the discharge summary, the time, the fact that we've asked the patient's consent if they're able to, and details of what we've supplied. And as part of that we check that the discharge summary was accurate in terms of the stop-started amended medication. So we do annotate the discharge summary on discharge to make it clear to the community pharmacy what we've actually done. Because before I guess you would have ticked on the coversheet that we used to use, the paper sheet, we would have ticked on there, seven day supply, Venalink dispensed, 14 day supply, you know, it would have been in black and white on paper, so this is kind of another way of…
I: With the cover letter with the discharge summary handwritten just…
R: Yeah, basically, yeah, yeah.
I: Faxed.
R: Yeah.
I: So it's quite a change…

HP4
R: Yeah, it is, yeah.
I: …it's quite a different change in the way of working, isn't it?
R: It's certainly easier. I think maybe sometimes it's sometimes easier to forget to annotate the discharge summary with all the documentation you're meant to put on, but we should be checking that anyway.

HP4
I: But is the system seamless enough then for them to just be able to go in and go, oh yeah, I've seen that, can they see what you've done…?
R: Yeah, they can see, yeah, so the community pharmacy like referral is just an electronic order, so it appears on the drug chart orders which is what we…it's like the thing we look at basically primarily. So yeah, it's really obvious.
I: Yeah. Well that's good, isn't it, yeah.
R: Yeah. The only slightly annoying thing with it is because it's relatively new, when people keep coming back in, you have to try and find out the postcode for the community pharmacy, just to make it easier to search, that's the only thing at the minute, but once people bounce back in and out, then we'll have it on the system, it's just not everybody in the past has recorded it.

HP5
So, you didn't know, so quite often when you think it's gone it's not gone. So, the information that the community pharmacy should be receiving they never receive, because of the type of system that we are actually using. Fax is an archaic system, it was good in its day, but the information highway, there's a lot more patients now using blister packs and so on. So, you know, if you were to fax information, the fax needed to be free, whereas they tend to be a shared line, I don't know because of costs whatever. So, the exchange of information could be halted by just the type of equipment we were using, and the circumstances under which we use them.
I: Yes, I know.
R: And faxes broke, faxes didn't always work and faxes weren't on every ward. That's the other thing as well, so if you had a fax on the EAU but if you went to another ward like on the L block et cetera, maybe one out of six wards had a fax machine. So, you know, it all impacted on an individual time wise.

HP5
I: Yes, right. And in terms of, yes, so you know, there was…it was introduced…do you think it, sort of….and you talked about the training and help you had here…that start-off, what went well in that sort of first week or so, 17 February I think?
R: Yes, I did send…what, what happened? I think I sent an e-referral twice. The worse that has happened that I sent a new referral twice because, that's the worst thing that happened. As to the use of the…it's quite easy to be honest. So, the other thing was searching for the chemist, it's knowing which chemist and picking and…Well, the more you do it the more you can pick up which chemists and select the right chemist by Google Maps. That one was a bit of a challenge because you had a small map, and being not from the local area it's knowing, oh, where do I look to find this chemist now and so on, but it's become easier as you use it.

HP5
I: So, but it's moving from here to community pharmacy, is there any transfer of information back from the community pharmacy to yourselves, or to here?
R: No, no.
I: Do you think there should be?
R: It would be helpful. I suppose the community pharmacist will deal with us on a need to know basis. I mean at the end of the day if you want information from the community pharmacy, we will have to initiate it, you know. So, it would be great if the community pharmacy could let us know, have a complete history of what a patient has had from them, but it's never necessarily complete because patients are not necessarily loyal to community pharmacy these days either. So, the best source of history of medication will be via the GP surgery, where we already have

access to the SCR anyway as to what has been dispensed, and we can just confirm that information with the community pharmacy. Having said that telephone systems are prone to being busy, a lot of people are using telephones to contact pharmacy et cetera. Whereas, if you can get an electronic confirmation of information safely that you need, then that could save time.

I: Do you know if it's been received and when you, when you send that through do you know that community pharmacist has gone oh yes, that's my patient.

R: Yes, because I phone them.

I: Oh right.

R: What I did, I'm not one for using a system and then assuming that it's worked perfectly.

HP5

R: Yes, I know, I know. I had to, for my own peace of mind the first, in fact the first at least a dozen e-referrals that are sent I actually phoned to make sure that they received them and what they've received as well in a pharmacy outcome. And there's another, there's a community pharmacy that I deal with that's actually next door to the facility where I work and I've actually walked, gone into there and I've said have you received. Oh yes, yes, yes, we have, we have. So, I, sort of, like, I've made sure. I've needed to be satisfied that it's working as I said it would. And I've had 100 per cent hit, hit with regards to the telephone call follow up of whether or not…So, I'm quite happy that it works as it said it would.

HP5

I: Yes, so who, are there, do you think…I mean one of the things when this started this service there were some thoughts there would be a three-way communication between community pharmacy practice-based pharmacists, GP in GP land and the hospital. Do you think that's happening or do you think that would be a good thing if it did happen, or what?

R: I can see the idea working. The problem is…well no, there aren't problems there are challenges. The challenge is this, if you are going to have three sets of disciplines working there has to be a starting point and it's pretty obvious a starting point would have to be ourselves when the patient comes in. Now, at what point are we going to involve say like the GP surgery. We already or the GP pharmacists, because we already have the information we need via the SCR right, which all of us can access if you've got the card.

I: Yes.

R: So, and we've also got something called SIRS which is the (name of place) Integrated Records System. There's not an awful lot that a pharmacist linked to a surgery can tell us unless they've done optimisation of medications and so on. Well, that doesn't necessarily involve e-referral, need to involve e-referral that could be documented as part of our SCR access as part of access in the medicines records so we can see what they've done. And contact shouldn't only be if we are uncertain as to why certain decisions have been made. For instance, because I had an interesting patient the other day who was on a clopidogrel on a PPI which is not normally used with that particular drug, but normally we would change it because one interferes with the efficacy of the other.

HP7

R: So, when I speak to a patient, when they are admitted. I find out if they have got a blister pack, I'll find out what pharmacy it is and if it's a (place) Pharmacy, I will go on the electronic system; there is a little link to get to it.

I: Right.

R: There is a link so you just click it, you type in – I always type in, the postcode of the pharmacy, because if you type in the name it's not going to map it to the map, so it's better to just type the name and the postcode, find it on the map. And, then I'll send it. Sometimes if the patients don't bring in their blister packs, and I don't know what time slots things go in, I might have to ring the pharmacy, or I will ask the family to bring the blister pack in.

I: Right. That's interesting because you've got, you are telling the pharmacist at that point of admission to stop making up the blister pack, and effectively that's the point of that notification.

R: Yeah, don't send it out for delivery, yeah.

HP8

R: Definitely, yeah. It doesn't take me a lot of time out of my day. It's fairly straightforward what I have to do with it and then when the NIPPS team chase it up they get quite quick answers about what's happened.

I: I think on that note, unless you've got anything...?

R: Just that in setting up the generic NIPPS email, that has then opened up more communication between the hospital and the NIPPS team, so (name) circulated the NIPPS email and said that you can send us non-urgent patient queries. So, again, I triage those and it's literally just this is the generic NIPPS email send something through. But we have had urgent patient queries sent through on them. So we need to work on our messaging.

I: Yeah, because if you're get anything urgent, because you're not looking at it regularly enough for...

R: I am looking at it regularly but we don't have the team in place that are in the patient surgeries. As I've said every...

I: Every day, no…yes, precisely.

R: ...like there's not a nominated pharmacy. It might be a week before a NIPPS team member works in a surgery due to...

HP9

R: Yeah. I think it would have been helpful, yeah, 'cause when I got the referrals and I looked at this big spreadsheet, I was like, I don't really know what's all these fields, there's loads of them and I was like, does that mean it's been done? 'Cause it said…I think on the status bit, said something like, that's been completed, but then (name) was like, no, I think that means that the chemists completed their side of it. I was like, oh, that's really confusing, 'cause I was like…and that makes it look like that has been done and sorted but…

I: Yeah, 'cause you're saying it was a spreadsheet…

R: Yeah.

I: …whereas…I mean, they're seeing it through PharmaOutcomes which is completely different.

R: Completely different, yeah.

I: But you're seeing it…

R: …as a spreadsheet, and it's got a lot of…

I: …numbers.

R: …fields and…yeah, numbers and the only thing that made sense was that status, but again, I'd interpreted that wrong because I thought that meant, yeah, I had dealt with it, me, as in…'cause I had dealt with it at that point, I was like, oh, but it says completed there…she was like, no, that means the chemist completed that part, I was like, right. Okay.
I: Yeah. So there is some sort of…would it be better if that was a simpler thing though?
R: Yeah, I think so, the spreadsheet definitely would be a bit more helpful. I don't know if that's because it's how it's pulled from the PharmaOutcomes, it…does it just pull it out [inaudible 13:33]?
I: Yeah, I don't know. I don't know how that …yes.
R: I think it pulls it off directly because some of the fields are just like…
I: That's being sent to (name) and then (name) is sending that onto whoever…
R: Yeah, to everyone else and she's got to tell us which field it is 'cause there's loads and loads and loads of patients on there.
I: Or does she send it to the lead for this? Are you the lead for this, no?
R: No, so she's cc'd the lead and me in the email and she sent it over to the people who are flagging it as they need highlighting, so that's what..
I: Yes, I think it's sent to the…
R: …lead, yeah, so it's (name) she sent it to and myself.

HP9
I: …on the discharge summary. And they might not match up. The discharge summary list should have been sent to the community pharmacist. What I'm trying to think of there is then if these things are happening, in what ways then is the referral going to help that?
R: Help it. Well, because I think the issue that…from working in (name of place) and now, the issue is with the discharges being sent to the chemist, it doesn't always happen because it is done by fax and it relies on the pharmacist actually calling the chemist, which they don't always, but their number could be busy for hours and hours. It doesn't sometimes…either the discharge doesn't arrive via the fax because there's a problem with the fax machine.
I: So you get discharges here by fax?
R: No, electronically…
I: Electronically.
R: …but it used to…
I: …it used to be by fax, yeah.
R: …it used to be…to the chemist it…
I: …by fax.
R: …used to be by fax until this new system came, and that used to be a nightmare. I know from working in the hospital and here, it just…it's a nightmare for both ends because it doesn't…fax is not really reliable and doesn't always work and the number's busy and sometimes they don't receive the full fax. So that causes a lot of issues and they always used to complain to the hospital that they'd not received it but then it's not always the hospital because they might have done their part but it's just not got through, so it's just completely not a reliable way of sending something that contains confidential information as well on the patient.

HP9
R: There's not, like, a set system at all.
I: There's no system?
R: No. Each practice is very different. And how much they send you and what they send you is very different as well. Some of them like to send a lot of letters, some of them send you discharges, or some a mix of both of them, so that's the only thing you do at some of them, just discharges. So it's very, very different. There's no set way. That's why every practice you go to you've got to start again from zero.
I: This is the evolving role of clinical pharmacy in general practice, isn't it?
R: Yeah. You've just got to adapt to what they do or learn their ways of doing it because yeah, it's different, you can't apply what you do here over there. Completely different. But you still – as a pharmacist – we still do the same process in each practice, we tend to try and call the patient and if we can't get in touch with them then we can't, but we try most of the time to just call them and speak to them about it. If they…sometimes I've had them where they've…I know that I'm going to confuse things so that's when I stop, like, if they're a blister pack and literally got no idea because someone helps them, so I usually speak to them.

P4_C4_C5
R3: That is when Alexa comes in really good isn't it?
R2: Yeah.
I: Oh really? Right.
R2: Yeah.
R3: Oh yeah.
I: Tell me about that.
R1: Alexa, aye.
R2: Echo.
R3: Echo.
I: Echo?
R1: She'll talk to you, so watch out.
R3: Have you got it switched on?
R2: You've turned it off (name of patient)
R3: Yeah, we've had Echo, yeah.
R1: It'll give you the time of day and remind you what you're doing.
R3: It my mam has to nip out, like for five, ten minutes, well I say five, ten minutes, she might nip over and see my sister, or whatever, and if it falls in line with him needing his tablets, she'll put them there for him, and, I'll be back whatever, and she'll get Echo to shout, (name of patient), take your tablets.
I: Oh, that's amazing.
R2: I give it a time.
R3: Yeah, give it a time, at one o'clock remind (name of patient) to take his tablets.

R1:	Have you switched it on now?
R2:	I'll have to do it in a minute.

3c 2 Code - Information Technology - benefits of, availability, access, use.

HP1
I:	And also, then, having to do further things, as you…I mean, do you think, what's the sort of, how's this going to move forward then, in terms of, is this going to stay the same like this, or is this going to be, do you think this'll be…?
R:	I mean, I think that there's a lot of things that don't need to be in this report that we get sent. I mean, this is assuming that we're not going to be in the e-referral system, and it's going to be a two-way, if it was a three-way, it would be a different story, 'cause we could actually see the system, and yeah, but currently, as it stands, we're just getting these reports as and when we need to. And the reports are really, there's information that we don't really need, like DISWA B3, ah, that's probably Discharge Ward.
I:	Discharge Ward, yeah.
R:	I mean, yeah, that's useful, because if we wanted to call the ward, and we know where they got discharged, yeah. But there are things that, I think there's information that we don't really need, and the information that we do need, probably needs highlighting more. We also definitely need an NHS number, or an easier way to get to know who the patient is, because not everyone can access EPR unfortunately, which I think they should be able to, but even if they can access it, it takes time. And the way that we access EPR in general practice, we have to log on via …and then we have to go on to our hospital system, and then we have to select Sunrise, and then we have to log onto Sunrise, so already there's three log ons, yeah, just to get onto the system, so it's not really that easy to do, yeah, and it takes time.

HP1
Yeah. So, a lot of pharmacists would be in the position anyway, it wouldn't be an unfamiliar system to…presumably. So, if you had that three, if you were seeing as the discharge or e-referral went to the community pharmacist, if it came to the practice pharmacist at the same time, how do you think that would be of benefit then?
R:	I mean, it depends on what needs changing. If it was something to change on a repeat, we'd just do it there and then. I think also, the issue is that the community pharmacists, there's been two occasions where they've said something, and actually it didn't need reporting, because it would have been removed anyway. So, if it was a, they had a repeat, and they said, it's still on the repeat, it was still on the repeat because that's an old prescription, an old repeat. So, sometimes I think that the selection of boxes, they also need to understand what that would look like for us, so I think the importance of the community pharmacy selecting the right boxes, and the hospital pharmacy selecting the right boxes, because if they select the wrong box, it just means something different to us. So, for example, I'll try to make sense of what I've just said, if it was about, like if we take this example, so, medicines stopped in hospital, still on repeat. This is what the pharmacy had told us. And again, it's difficult, 'cause I haven't seen the system, I don't even know how they do it. But, do they know that that is actually the warning for us, the issue that's been highlighted for us to action, or are they just ticking this, assuming that it's just information, just saying that the hospital's stopped this, and it's on repeat, but it's fine, it's nothing we want anyone to do anything about it, we're just stating a fact. But I don't think that they realise that that, to us, is, okay, it's stopped in hospital, but it's still on repeat, there's a problem, why is it still on repeat? But then going back to the community pharmacy, when I called them, they said, oh it's fine, it's just that it had been stopped, but I don't think they realised that that was something that's been highlighted…

HP11
R:	Well, it's stuff we would have been doing anyway really. I suppose what's really good is you could go on and check…if you wanted to reprint it, you could, couldn't you? Whereas if we had a…it's there, isn't it?
I:	It's there. It's in that system.
R:	Rather than just a…
I:	That's interesting, yeah.
R:	…rather than just a piece of paper that we might have taken next door and forgotten to copy it, we could just go back on and look at that.

HP11
R:	Well, definitely because it needs to go to whoever is arranging the discharge prescriptions, doesn't it? There's no point in sending it to somebody who's not. So I imagined they were just sending it, he would then get that information, but he's not…
I:	No.
R:	…he's not doing…
I:	It's not.
R:	…so…but…
I:	…he's the practice-based pharmacist, but yes…
R:	Yeah, so what's the point of that, because he's then relying on us to go in with a bit of paper.
I:	Yeah. That was a sort of …
R:	So that is probably where it's falling down, if [five 28:02] didn't really know why he wasn't seeing that, whether they just hadn't allowed him access to the right email box or whether it wasn't being directed properly within the practice. But that's obviously…

HP12
R:	We have now got the pharmalarm as well.
I:	Yes. Talk me through those actually, because I've heard about this.
R:	Yeah, so it's like a little plug in alarm thing that basically changes colour when we get any messages or any notifications. So, you still get the email as well. So, sometimes it will change and start flashing blue when we haven't even noticed and we just see the email first. It's just like a little widget that changes colour and flashes.
I:	When you have got a message?
R:	Yes, so it lights up lighter initially if we've got any discharge referrals it will flash blue.
I:	That's cool.
R:	Other times we don't notice it until we go on the emails and notice that we've got an email. We are getting better at noticing it.
I:	So you get an email to say there is a message, you now get a pharmalarm.

R: So we get the pharm alarm as well to say we've got a message so we can go on and see the messages. And, then obviously click on the message and action it according to what we need to do.

HP2

R: It's brilliant. Love it. Yeah. It's really, really good. Because it has freed so much time for everybody and it's quick, easy to do. And we do it…it's actually in our medicines reconciliation, like, form now on the computer. It was separate before but now you start your medicines, reconciliation it is there, right in the middle, just click on it and yeah, it's gone.

HP3

I: And especially, as you say, if you don't particularly know. And I suppose the final thing to finish up now is, do you think this can/will/will not improve care?
R: I think it will improve care.
I: In what ways?
R: I think just moving more towards... I feel like just overall we're moving more towards more technology. I love the fact that this is an electronic prescribing hospital. I think the paper charts are old, I think faxing is old. I prefer... I think it's just more efficient and more convenient to have things like e-referral, have things like emailing and just sending things electronically. So I think it's just progressive, pretty much.

HP4

R: Yeah, so this week we've just recently had a new meds reconciliation document version, I don't know if you've heard of that, but that now links to the e-referral, which is really good, because…
I: Right, so this is an electronic document, yes.
R: It's an electronic document which pharmacists and technicians use to basically – it's like a stepwise document you kind of go through, and it's all about accurately documenting the drug history for a patient, so when they come in it says, which sources have you used, and it will be GP prescription, patient, patients on medicines for example, so you'd tick it through, you'd work your way through, it's, does the patient have any medicines with them. And then you document the drug history, so you write whatever they're on at home, and then it says, is this – I can't remember the exact kind of terminology, but it says something like, can you complete an e-referral, do they have a compliance aid basically, and if you tick yes, it automatically brings up the box for the e-referral, so you can click on that and it exports you to…
I: And then just takes you…
R: …yeah, takes you straight through to the e-referral, which is good because it means you're less likely to forget to do it.
I: Yes, so you haven't got to think, oh, that patient's got a Venalink…
R: No, so…
I: …I need to do…
R: …you did last week, but since this new kind of document has been introduced…
I: So this is a change from when it originally started then.
R: It is, yeah.
I: Has that change occurred because of the e-referral or is it just something separate?
R: I don't think so, it's been in the pipeline for a long time, there were lots of new things that the Trust were asking pharmacy to do or things that…the old document we used was kind of donkey's years old, so this new document incorporates a lot of kind of things we do now that weren't necessarily there 20 years ago when it was introduced, so it's just been modernised really to kind of work better for us. But part of that…
I: But because of that…
R: But because of that, they've been able to incorporate it, which is really good I think.2MJ26/02/2020 16:09
I: But is the system seamless enough then for them to just be able to go in and go, oh yeah, I've seen that, can they see what you've done…?
R: Yeah, they can see, yeah, so the community pharmacy like referral is just an electronic order, so it appears on the drug chart orders which is what we…it's like the thing we look at basically primarily. So yeah, it's really obvious.
I: Yeah. Well that's good, isn't it, yeah.
R: Yeah. The only slightly annoying thing with it is because it's relatively new, when people keep coming back in, you have to try and find out the postcode for the community pharmacy, just to make it easier to search, that's the only thing at the minute, but once people bounce back in and out, then we'll have it on the system, it's just not everybody in the past has recorded it.

HP5

I: Yes, so who, are there, do you think…I mean one of the things when this started this service there were some thoughts there would be a three-way communication between community pharmacy practice-based pharmacists, GP in GP land and the hospital. Do you think that's happening or do you think that would be a good thing if it did happen, or what?
R: I can see the idea working. The problem is…well no, there aren't problems there are challenges. The challenge is this, if you are going to have three sets of disciplines working there has to be a starting point and it's pretty obvious a starting point would have to be ourselves when the patient comes in. Now, at what point are we going to involve say like the GP surgery. We already or the GP pharmacists, because we already have the information we need via the SCR right, which all of us can access if you've got the card.
I: Yes.
R: So, and we've also got something called SIRS which is the (name of place) Integrated Records System. There's not an awful lot that a pharmacist linked to a surgery can tell us unless they've done optimisation of medications and so on. Well, that doesn't necessarily involve e-referral, need to involve e-referral that could be documented as part of our SCR access as part of access in the medicines records so we can see what they've done. And contact shouldn't only be if we are uncertain as to why certain decisions have been made. For instance, because I had an interesting patient the other day who was on a clopidogrel on a PPI which is not normally used with that particular drug, but normally we would change it because one interferes with the efficacy of the other.

HP8

I: Right, okay. So then that was devised that that was what was going to happen. We can go on as to how it's happened, whether it's worked, as we move on. So when it went live was there any initial, sort of, like, response from your end what was happening? Did you see that, oh, gosh, suddenly it's all happened?

R: Not really. It was difficult. So initially I don't think the email address had actually been set up in time because we had to contact (name of hospital) IT to get them to set that up, making no comment on (name of hospital) IT. So I was set up instead with the log in for PharmOutcomes, which is a portal, I'm not too sure, I think it's mainly used by community pharmacy. I was set up with a log on so that I could go and manually download the spreadsheets. So that's what I was doing for the first week or so, I believe, of the service, which was difficult because there was quite a lot of different reports I could have downloaded. So I was, sort of, downloading them, sending them to (name) and going is this the right one and she was, like, oh, I don't think it is, no, it's here. So there was a lot of screen shots back and forth in emails before I realised what the right one was, so that was in the initial couple of weeks. And then once I'd gotten the right report, it was then reading that report because when you open it, you just got a massive Excel spreadsheet, loads of columns, loads of rows, mostly numerical data. So, like, MRN numbers, ages, a few, I guess, multiple choice, like, male or female, and then there's some free text boxes as well. So locating where it was that I had to look to see whether a referral was made back to the GP surgery was the next big challenge. Eventually we found the column and, yeah, it would either say yes or no referral back to GP. And then there was some more free text boxes, which would say a little bit more about the problem that you can then read.

HP8

I: So (name) or whoever comes to you and says, (name), we've decided that e-referrals service needs changing and we think you're the person to change it, so what would you do? You're in charge now, what are you going to do?

R: So I know that one of my pharmacist said to me that it was very difficult to identify the patients from the information given. I don't know what information they need but I would find out and see if we could add that information. Maybe it's their NHS number, which I don't think is included in the spreadsheet, so it's hard for them to identify patients. So that would be the first thing I'd do to improve it. See if I could fix the lag that (name) was talking about as well because then maybe we could action things a little bit quicker and it wouldn't automatically go...Or the community pharmacy wouldn't send the communication to the GP in the first instance, so the GP wouldn't then book appointments in with patients who could maybe see the pharmacist for a better outcome.

I: So you getting the information earlier basically.

R: Yeah. And then maybe in an ideal world, we could get a spreadsheet with less of the information we don't need, just the information we do need.

I: So streamlining things really?

3c 3.Code - MDS - The processes and technology involved

HP10.

I: Just before we move forward, your role, you're a community pharmacist, yeah?

R: Yeah, I am. I'm a community pharmacist and my role is... Overall, my label is I'm pharmacy manager as well, so I manage the staff as well as manage the pharmacy. If you want some more information about the pharmacy, of the roles, it's about 11... In the pharmacy, we judge how big the pharmacy is by the numbers of items we will dispense in a month, so we do around 11,000, which I think is pretty big. We've got five members of staff, three full-timers, two part-timers, and we do a wide range of services, a wide range of things. We've got about...which is related to our interview, our chat, I've got about 100 patients on blister packs, so this is quite a bit. So one full-timer is completely dedicated to do this task and others sometimes will help with the admin of it. It's a huge task. We've calculated it takes more than 40 hours of work to run it. There are a number of issues with it but, you know, that's pretty much the description of my pharmacy in relation to blister packs and to overall.

HP10

I: You said earlier you've got about 100 patients on blister packs, so how many referrals are you getting? And they're obviously not all going into hospital all the time.

R: It's hard to give you the exact number. I would say, overall, we've got, now, since it was introduced, probably around...between 25 and 30.

I: That's quite a lot.

R: Quite a bit, yeah, but sometimes we will have, say, four or five happening at the same time. At the moment, I've got, now, two patients who are in, no discharge is coming out. So always some activity on it, but there's no, like, regular...

I: I suppose by the nature of the person, somebody who's on a blister pack, they are vulnerable and they're likely to... There's a possibility of them being hospitalised.

R: Unfortunately, they're usually on numerous medications, complicated patients with overlapping conditions. It's usually patients who, unfortunately, they suffer a lot of problems and have been admitted, so it's not like the simplest patients.

HP11

R: Just what we expected to happen, really. Except actually I think the very first one…the one of the first few – I can't remember if it was the very first one – it wasn't our patient. It was from MDS, which is our other unit.

I: Oh, yes. Yes.

R: So I think I had to send it back. And then I think I tried to phone and say, mm, what do I do about this, and…? But yeah, I think I spoke to (name), then actually, who was really helpful.

I: Yeah, because you've got a unit that deals with the MDSs, is that right?

R: Yes. Well, they h…

I: Some of them.

R: At the moment they've got a robot which I think is going, but yeah. That's all they do, basically.

I: Yeah, but you still do…deal with Venalinks and stuff here as well, do you?

R: Yeah, see, at one time we were sending out some of our Venalinks, not all of them. It's complicated because they can't put everything…their robot doesn't have all the drugs, so we were sending some of our simple ones to them, but it's a real faff because we had to get the scripts, process the scripts, encrypt it all, send it up there and then they would send it back and then we still had to check it and it was just…

I: You might as well just be doing them, yeah.

R: So because of IT issues, we got a new IT system and we couldn't do that while we were using the robot, so we used that as a…that's fine, we'll just do our own and I think it was a good decision. So yeah, so there was a bit of messing about where we were getting MDSs but that very rarely happens now.

HP2

I: Yeah. Stuff like that. Yeah. Yeah. Precisely. And in terms of the way you, you know, using it as part of your work, how often are you sending out those referrals or how often do you go into the system?

R: The e-referral system. Every time a patient who has a blister pack, so, it depends how many we've got on the ward at any one time. So, maybe…I think I saw two patients with blister packs yesterday, but there are other people doing medicines reconciliations, so, then you do…they are probably using it as well. So, I couldn't say day-to-day. It's constant, if, you know…

I: If you've got those patients.

R: If you've got people coming in with a blister pack or a Venalink, then, yeah.

HP4

R: So I think inevitably the whole, you know, people who have blister packs and Venalinks need them for a reason, so generally they're elderly patients, patients who might live at home who are getting muddled up with their medicines or they might forget to take them, people with learning disabilities or disabilities in general, people sometimes just if they're on lots of medicines, they're younger but they can't quite get their head around what's meant to be taken at what time, you do see some younger patients come in with blister packs as well. I guess it's that demographic of patients who are higher risk generally, aren't they, and they are going to need that extra support with their medication, generally they're on lots more medication because most of them are elderly patients who are polypharmacy like I said who need a lot of careful titrating with their medication, medication reviews, all that kind of stuff. And it's easy to make mistakes, when they're on 20-30 medicines or when they come into hospital, it's easy to prescribe the wrong, you know, you're inevitably going to get some prescribing errors on admission. So it really makes it important I think for those kind of patients if you get the medicines right on discharge, because otherwise they're just going to end up taking the wrong thing, aren't they, they'll just take whatever is in the blister pack.

I: Yes, yeah, we said, because you're totally reliant upon it.

R: You are. And carers as well, because a lot of the patients who have carers in (place), the carers won't administer medication if it's not in a blister pack. Which is…so, yeah, I mean, it's difficult, isn't it, because if you didn't have a patient with a blister pack, you'd just say, these are the changes, don't take this one anymore, we're going to increase this, we're going to give you an extra dose of this – it just makes it so much more tricky when you've got cognitive impairment, dementia, elderly patients on lots of stuff, it just…it's just waiting for mistakes to happen, isn't it, really, I think.

HP5

I: And that's just a chat, as you, it's a bit like your telephone, looking to [inaudible 0.28.51], it's checking it's all working through isn't it. It's a sort of interesting, sort of, evaluating of it that it's all sort of happening. One of the things that we, you know…currently it's used purely and simply for the blister packs. Do you think it should perhaps be used, sort of, more widely for, you know, discharges more generally, what do you think about that?

R: It's safe to use that way, it is definitely safe to use that way. I suppose the supply of original packs, let's say patients are not using blister packs, these patients tend to be not so loyal to their chemist. But I must admit I have used it on occasion. That…one thing I forgot to mention within the facility is that sometimes we start a package of care. Package of care implies that we have carers come in and they will only get medications from blister packs, and therefore we need to initiate the supply of the blister pack.

I: Right, you start them off, yes.

R: So, we need to find out from the patient which chemist they normally use, and then set up an e-referral as well. So, sometimes we used to initiate rather than continue as well, I've used it for that basis, providing the chemist agrees to continue a supply of blister packs once the patient is discharged as well. So, we've used it for that, so yes it will benefit us with patients who are actually using original packs as well, because of the number of instances I've had where we've had to initiate the supply of blister packs because of the type of care a patient needed post discharge.

P2

I: Have you talked to the chemist at all about it, or has anyone talked to the chemist?

R: No, because you see, I used to get it from…because my doctors is at (name of surgery), and I used to get me medication from there, but when they put it in the blister pack they moved me to (name of pharmacy). I don't even know where (name of pharmacy) is, I haven't got a clue where it is, so they're just sending these now from there and I don't know…

I: (name of pharmacy)?

R: I've no idea where it is. You know, I haven't got a clue. So, it just comes from (name of pharmacy), and that's all I know.

I: Right, okay.

R: You know, I don't know anything else. You know, I mean, before it came from (name of surgery) and I knew and if it was right, I could go in and get the prescription.

I: Right, so you don't even know where it's…

R: No, where now they just give me this because of the way that I am forgetful. And I just said, well you know, I can't remember what I'm taking so they said we'll give you the blister pack.

P4_C4_C5

R2: Well, I'd be opening boxes all the time.

I: Yeah, because it's all organised, it's…four time a day isn't it?

R2: Yeah, what's not in there, his is for his moods.

I: Right.

R2: I've got that in a separate, and…

I: Why's that separate, is it recent or?

R2: It's 'cause they changed it.

I: They've changed it.

R2: And they didn't put it in the first week.

I: Right.

R1: It's not got in the system yet.

I: Yeah.

R2: So, it's in the system now, and she's sent it, before we got it off the daughter, when we went.

I: Right, yeah.
R2: Now I've got two lots.
I: Oh right.
R3: Yeah, for my mam to stand there every morning, sorting them out, or every week, it would be an absolute nightmare.

Theme 4 a Changes in meds safety work

4a 1 Code - Admin time down - Clinical time up

HP10
I: That's something, isn't it? Because one of the things I think that was said to me way back, sort of last year sometime, around community pharmacy, and I know that there were people really pushing this agenda forward and saying, well, actually, this is going to give value to community pharmacy. Do you think it has that potential to do that?
R: Most definitely. Honestly, most definitely. We, as a pharmacist, believe me, we do, apart from making accuracy checks, we clinically check things. And if you're clinical checking, it's not only about interactions and side-effects of meds, it's about the broader picture, so you're looking at a number of things. As a clinical check, you're making sure that an elderly lady, can she open it, can she apply that drop, has she had any falls, what's her history? When I do a Medicine Use Review, I will ask them, let them speak for a minute. I say, how are you doing, how are things, how are you managing your meds, how is your life to do with your meds? I listen to them, so that gives you an understanding and picture and I always make myself notes, so, oh, this is this patient. If you try... I always call it I'm trying to do a proper job today. So if you do a proper job, that's how it should be, so as much information as you get, you're able to apply the information later in order to improve the overall safety of patients, and that's it.

HP10
R: Yes, you're right. You're right, yes, it does. I'm not sure whether you are trying to refer to the fact that I've just said about communication with the patient.
I: Yeah.
R: That's what you meant. Yeah, that's right. True, I didn't think about that initially, it just came out in the conversation. Yeah, you're right. It's kind of like you're looking at it, when you're dealing with that on PharmaOutcomes it says, what have you done? Commenced a blister pack, phone them post discharge, MUR, NMS, this, that and the other. So you're thinking, oh, maybe I should do that, you know, like even that or just... It's just basic communication and those people often, you know, they don't... You're not trying to patronise them in any way, but where I work, there are a lot of working class people, so they're just people that don't necessarily understand things very well but they appreciate, like, you know, like you look after them, you tell them, you... To be honest with you, if you ring them, give them a ring and reassure them, you save yourself phone calls later, oh, where's this, where's that? It's just you do it and you know everybody is happy, it's much better. And again, you know, as I said to you, maybe it's me, but I always enjoy doing clinical stuff, I always like to kind of have that broader picture overall. Communication makes them safe and makes the drugs they use safer, in my view.

HP13
R: I just feel I'm giving those patients a much better service because of this than I used to. I just feel that and I'm sure they feel that too, you know.
I: Because of that conversation?
R: Yeah, I really did used to think it was always the doctors' responsibility whereas now I had to be dragged into it and I prefer that. I feel as if it's a good…you're giving them a good service and making sure there's no errors, yeah.
I: And do you feel the patients value that?
R: Oh yeah they do, definitely, 'cause that's another thing, when I call at their house, I'll take the old packs away 'cause that's another issue if the medicines have changed, you don't want them picking up two weeks' ago, you don't know how long they've been in hospital, they might've been in hospital two months, they might have loads of packs there so I'll clear them out when I take the new pack. And I will write on the new pack so I know to tell them that this is the new, up-to-date pack and I get rid of all the others and take them away.
I: Right, yeah, and so the patient, yeah.
R: I didn't used to do that, I don't think I did used to do that, I just used to deliver it and say, that's your new medicines, you see. It's just a safer service, really, it's made it safer.

HP16
I know that's quite a long question, it's quite a detailed question, but if we're thinking of value, - patients, community pharmacies, hospital, what is that value, what is that impact that's [voices overlap 19:57].
R: I think there's multiple impact really, for the patient it's seamless care which is the aim of the project, it's about seamless transfer of care between the two sectors. For a patient that's what they need, they want to see the medicines on time, they want to see that actually they've not had to chase things up, they're not worried, they're not anxious about getting their medicines. Those sorts of things, actually will they come on time, did you know I was in hospital, did you know I've come out, did you know this has changed? All these things are avoided. But actually from a GP perspective they know that we're getting the same information now so actually we're not phoning up, our phone calls are not, have you got a copy of the discharge, has there been any changes? Those questions which are more, I would say, logistical, we're moving to clinical conversations, we're actually doing the technical conversation rather than just all the admin stuff that actually this sorts out because we've got the information there. It's what do you do with the information rather than actually have we got the information in the first place?

HP16
R: It makes things easier for us, to be honest with you, now I'm a very big data person so I love having information, I'd love to be able to see the GP record, I'd love to be able to make our contacts count even more because we have more information to deal with at our disposal. So that would be great for us because that impacts on clinical care but actually trying to get hold of a fax, trying to phone a GP about, have you got a discharge, can you send it over, what's changed, what hasn't, that whole workflow for us is reduced because I've got a copy of the discharge there to utilise. So that means again our conversations are more technical, they're more about the clinical, the issues rather than actually trying to do, in essence, admin work, trying to just get the information so that we can try and do something with it.

HP2

I: Yeah. Oh, that's actually brought us perfectly seamlessly on to our next question, which is about how it was introduced into your workplace. So, what, sort of, things went well, when that went live, 17 February wasn't it? I think.

R: I think it was, yes. Well, it was just so easy to follow. Just the whole process was easy to follow. You know, you clicked on one thing and the next screen came up. So, it felt too easy. Yeah, it felt too easy to be right, but because we did that, we knew the people in the dispensary every evening who were faxing, faxing, faxing, had all that spare time to do something else. So, it took time off people down in the dispensary…a busy time of night, when they are faxing all of these prescriptions. So, they could carry on with something else, help out other staff, so…

I: Yeah. So, it freed up people.

R: It freed up people. Yeah.

I: Who had been, sort of, like, clogged down with this.

R: Yeah. Who were standing at the fax machine, just faxing for however long.

HP3

R: It allows me time to actually review the ward and look at other patients who are probably more sick, other patients that have been in longer. It just allows me more time to do other ward work or just work on other...

HP4

I: Is it therefore, you know, it's adapted the way you work then, has it then improved things…

R: Yeah.

I: …save time or…?

R: It does save time, definitely, I would say it probably doesn't save – well, it saves a little bit of time on admission because otherwise you would have had to ring the community pharmacy, so it's quite nice to not have to ring them in certain situations. You know, if you've got the blister pack in front of you and you've got all the information you need, you can just stick the e-referral on. Sometimes you'd be ringing all morning trying to get through to the chemist and it would waste a lot of time actually having to keep going back and ringing the same number and nobody is answering. So that definitely saves time. And then on discharge as well obviously with the whole, you know, it's just much easier on discharge.

I: One of the things when health professionals say to me that that saves time or this only takes so much time and now we've saved some time, one of the things I often say is then, so what do you do with that time?

R: Yeah. Well, we see more patients, which is good, or you have more time to do clinical things rather than just kind of, you know, desk…

I: So it takes away some of this administrative work…

R: Yeah, exactly.

I: …and brings you back into those patients.

R: Yeah, that's right, which helps, you know, so we can then have more time to correct mistakes and prescribe on the ward and things like that. So yeah, I would say definitely saves time.

HP5

R: Oh, the e-referral itself has saved time. I mean, there is no joy going to a fax machine and having to wait for all those pages to go through and then at the end of it, it puts…it's not gone. And then you've got to send it again, you've got something else you want to get on with.

I: Yes, got to send it again.

R: And e-referral, in fact at the point of discharge, that's where the big difference is. I don't have to worry about sending any paperwork to the chemist once the e-referral is initially set.

I: Because once it's initiated in the system it's happened. So, that bulk of work that would have happened at discharge isn't now happening. So, I assume, a huge sum of work. And as I often say to health professionals when they say, right, it saved loads of time, what do then do with that time, that time you've gained, you know.

R: I work at different facilities. That time I have gained helps me to…I've got another project that I'm on, so I'm able to focus also on that project a little bit more. I'm not under pressure, I don't feel as much under pressure, let's put it like that. I feel less of the pressure. I mean the only other thing I do differently is once the patients are there, I make sure I go through the list of patients who have got blister packs and make sure they've got e-referrals. So that, but that doesn't take long, you know. It's a small price to pay for something that saves a lot of time.

4a 2 Code - Discharge process, structures, systems and new ways of working. Benefits of, or problems, with the new system

C2_C3

I: That's actually the next question, what information was given to you, and that's good. So have there been any problems since last Friday in terms of the medicines?

R1: No, everything's been pretty good at it. They tend to deliver it the day before you use your last tablet in the packet. So they're pretty…

R2: And if you're not in, he comes in.

R1: He has a key, he has that arrangement. They've got the key number, so we don't miss any. He just comes in shouting I've got your tablets and leave them. And if on the odd occasion I've got my mum out and he can't bring them in or he doesn't…some bring them in, some don't, they'll always leave it with the next door neighbour. So they're quite good really, I've never had a problem where they've not left them, they've been quite good.

I: So you're getting them on time.

R1: Yeah.

I: Are they all there? Is everything alright with the blister pack? You've not had problems with the wrong ones?

R1: No, that's never happened.

C2_C3

I: Did they make the change…because she came out…would have come out, what seven days…

R1: On the Friday, she came out with the hospital ones.

I: For seven days.

R1: For seven days, and then they delivered those last…would have been Friday.

I: And the changes that were made in hospital have now been made into that.

R1: Yeah, continuing them.

I:	So it's all sort of worked.
R1:	Yeah.
R2:	Quite organised.
R1:	Yeah, it's pretty organised actually, it really is.

HP1
I:	Actually, lets, because, we've jumped a bit around, but we can come back to other stuff anyway. In terms of then how it was introduced into what you're doing, to your work then, what happened firstly? Just talk me through how you started getting those referrals, or those notifications?
R:	Well, I knew it was coming, because I get the emails from (name), and we were just told, in our team, in our Leads Meeting, what the procedure was going to be, that (name) was going to forward it on to us. We hadn't seen what it would look like or anything, so I just received an email, and just the attachment, and that was the first time, that I had it, a few months ago, yeah.
I:	Right.
R:	So, we hadn't been trained or anything like that, we didn't know what it was going to look like. We just knew that there's going to be an email, a generic email that (name) was going to look over. We have agreed that if she's on annual leave or off sick, then she would then give that role to somebody else, one of us, which has happened since, and yeah, so that's just what we were told and that's just what…

HP1
R:	Yeah, I mean I try not to leave it a week, but it depends on them, like (name of practice) are the largest in (name), so each of them are over 10,000, so there's many discharges a day, so yeah, so generally maximum a week, yeah, and they're getting it after two hours. So it's kind of, they're calling me, well I've not got to that yet, just one…so it's a bit of a waste of time.
I:	So, because it's automatic, because that's the point of it being electronic, it's that quick?
R:	Yeah, which is good, it's definitely good, but it would probably be better if we had it, because then we could then action it all, because the community pharmacy can't really do much, apart from tell us about the change, and then we make the change. Yes, they can amend the dosette, but without an actual prescription, it's risk for them, so some pharmacists, I know from working in community might not want to change anything from that, they want an actual prescription to see, even though…
I:	So, even though [inaudible 00:14:19] doesn't need, or it needs x instead of y.
R:	Yeah, I don't think many, especially the inexperienced pharmacists, the newly qualified pharmacists, I don't think they'd be comfortable to send out a Venalink, just from a discharge summary, 'cause they're still quite young and inexperienced, they would like to see a valid prescription, to say, you know, what the patient is having, and then they will send it out.

HP10.
I:	One of the next bits is about who uses is, but you've talked a bit about that, but sort of talk me through, actually, how it works then when you get a referral through.
R:	Okay. Right, I'll tell you from my own kind of practice. So obviously when we get any information... First of all, the system is quite good because it sends us an email as well, and I'm not sure whether that was introduced right from the beginning, but we usually get a notification. So my staff will always scan all the emails in the main mailbox and they will send me anything relevant to my attention, to my personal NHS email, and I will always check, oh, there's something from PharmOutcomes, a notification, that's one prompt. And again, as I said to you, myself and recently I've asked one of the members of staff who normally looks after blister packs to, as well, check it twice a day. We check it in the morning and check in the afternoon. So if we get information that one of the patients is going, we check it, oh, this is our patient, yes it is, okay, cool. We're making a note, we're obviously finding out what's...you know, where are we up to, whether we have got a blister pack on the shelf, whether it's out for delivery or just there, whether it's maybe with the delivery driver to be delivered on a given day, so then we're stopping it from going out. Just checking whereabouts where we are with this patient and putting it on hold. So if there's any information, usually written information, the patient in the hospital bank, right in the middle of the file, each patient in my pharmacy will have a file with all the information sent, all the discharges will be kept in that file. So we just put big letters, 'in hospital now', ping, and that's it. And I always just look at it and on the PharmOutcomes you just accept it, I think, and that's it, done. And then obviously once we're getting any information that the patient is out, so that immediately gets picked up. We're looking at the discharge. We normally, now, we've got a system with next door that we will print it off, write down, for attention of (name) or (name), who are both pharmacists at the medical centre, and that will be faxed across to them. They keep saying to us that we get this quicker than them, that information comes through us faster than it comes through on their screens, but they at least know they will put it on their, whatever, list, but they know this patient. And we always try to write down or highlight in a discharge, she will be running out of her meds on a given day, let's say on Friday, because, you see, we've got a number of patients that we deliver to.

HP10.
So we try to make sure that the next door pharmacist, doctors, whoever is managing the discharge, will know exactly when do we need that set of prescriptions, see if there are any changes for. And then we're looking out for scripts. Recently, it has become better and better. We will sometimes obviously chase things up or we will receive things: oh, there is an error here, there is an error there, we will question things, but sometimes realising they don't always necessarily always agree, the GPs, or with what's on that discharge, they will make their own changes, they will make their own little implementations, you know, whatever they do. We, ultimately, do what we're told, but at the same time, we watch their hands, we're making sure that this is going to be right. So we will be in the habit of still double-checking. We receive something that is in contrary with what the district was saying and we'll say, guys, is that definitely right, that furosemide, is it definitely...? It says there 40 and you just dropped it to 20. Oh yeah, because she's doing a blood test in a week's time so we'll then... So we always have that verbal communication, and once everything is cross-checked, then the preparation will start. We'll just prepare it. I always do an in initial check against the discharge. So, to be honest with you, my, (name) who does the blister packs, she's really good, she's so good that she always... I check but it's hardly ever that I pick up any errors or discrepancies, but I do it anyway, I'll always check the information on the script against the discharge initially, and then once I'm happy with that, then I will do whatever checks need doing, and then it's going out. So, yeah, I think that's the best way I can describe it really, giving you that kind of journey of that.

HP11
I:	Yeah, that's fine, yeah, absolutely. No, that's perfect. So thinking about the pharmacy referral service, why do you think it was introduced? What do you think was the point of it, if you like?

R: Well, I think it's always been particularly good in (name of place) anyway because they've always let community pharmacists know directly, which a lot of places don't do. So (name of hospital) has always been a bit ahead of its time with that. And then they've always communicated directly with the GPs, where I know, like my in-laws live in the Midlands and they get a scrappy piece of paper when they come out of hospital, to take themselves to the GP. Presumably they then get a letter at some point but there isn't the direct contact that they have in (name of place). But obviously, before they were just faxing it, it's a bit hit and miss and sometimes they'd fax our phone number rather than our fax number and it's not secure, is it?

HP11
I: But now, with the notification of admission at least…
R: You know they're in hospital, you can…we have a system where we…and it's not a very sophisticated system but we cross them out the book and we put the pack on one side and we write in the hospital pile, so we're not sending that until we then get the discharge.

HP11
R: Just what we expected to happen, really. Except actually I think the very first one…the one of the first few – I can't remember if it was the very first one – it wasn't our patient. It was from MDS, which is our other unit.
I: Oh, yes. Yes.
R: So I think I had to send it back. And then I think I tried to phone and say, mm, what do I do about this, and…? But yeah, I think I spoke to (name), then actually, who was really helpful.
I: Yeah, because you've got a unit that deals with the MDSs, is that right?
R: Yes. Well, they h…
I: Some of them.
R: At the moment they've got a robot which I think is going, but yeah. That's all they do, basically.
I: Yeah, but you still do…deal with Venalinks and stuff here as well, do you?
R: Yeah, see, at one time we were sending out some of our Venalinks, not all of them. It's complicated because they can't put everything…their robot doesn't have all the drugs, so we were sending some of our simple ones to them, but it's a real faff because we had to get the scripts, process the scripts, encrypt it all, send it up there and then they would send it back and then we still had to check it and it was just…
I: You might as well just be doing them, yeah.
R: So because of IT issues, we got a new IT system and we couldn't do that while we were using the robot, so we used that as a…that's fine, we'll just do our own and I think it was a good decision. So yeah, so there was a bit of messing about where we were getting MDSs but that very rarely happens now.

HP11
I: So what sort of…who uses it here then? Who's involved in doing things here, I think, 'cause you mentioned somebody else who…
R: So (name), and I and possibly the pre-reg, we've just got a new pre-reg…we tend to be checking the emails and then we've got the PharmAlarm thing now. So we'll tend to go onto PharmOutcomes and print the information off and then pass that onto the lady who's physically doing them and she tends to liaise with the surgery about the scripts. She'll go and say to (name),, this patient's been discharged, here's the discharge, when can I have the scripts? And we need them for this…the [inaudible 11:12] needs to be sent out.
I: Are most of your patients at that surgery next door?
R: Yes.
I: That makes life…must be very…makes it…
R: Especially our Venalink patients. Yeah, we do have a lot of patients who are from elsewhere, but most of our Venalink patients are…yeah, we've got…
I: And it being such a big practice.
R: Yeah, we've got the odd one like…it tends to be like parents of staff who are a different one…who are at different practices that we've taken on. But yeah, most of our…yeah, I would say probably 98 per cent of the Venalink patients are next door. 'Cause it's like…you're right, it just makes it so much easier.

HP11
I: Yeah. So can you talk through what happens when you get that first…from the moment you get the admission notification?
R: So when we get the admission notification we'll just say, oh, this patient's in hospital, we're not sending any more deliveries, we're not expecting them to be coming and collecting them, put them to one side and then we'll just leave it at that, then 'til we hear something. And then…do you want me to move onto the discharge?
I: Yeah.
R: Yeah. So then when we get the discharge, we'll look at when they need it for. So we have a control sheet with everything written on and signed off and then any discharges from the hospital we keep in a folder for that patient. So then we would then look at what medication they were on before, what medication they're on now, any changes that have been made and then go and speak to the practice pharmacist, and then he would then generate the scripts and then we would then make those changes to our sheet. And it would be clinically checked by me or (name). And then we'd make the pack up and either deliver it or they would collect it.

HP12
I: Can we talk about when it…so you have had that bit of training, so can you describe when it was introduced here, when the first ones started coming through, what happened?
R: I got quite excited when we got the first email, it was like, oh, we've got one. So, to be fair, as I know the first one we got we all huddled round the computer together and it was like let's work out how to do this, how to see it, let's everybody look at it.
I: So, it gives an email to say you've got…?
R: So, we get an email information to say there is a pharm outcome referral from the hospital. We get that whether it's an inpatient or a discharge.
I: Yeah.
R: We have now got the pharmalarm as well.
I: Yes. Talk me through those actually, because I've heard about this.

R: Yeah, so it's like a little plug in alarm thing that basically changes colour when we get any messages or any notifications. So, you still get the email as well. So, sometimes it will change and start flashing blue when we haven't even noticed and we just see the email first. It's just like a little widget that changes colour and flashes.

HP12

I: When you have got a message?

R: Yes, so it lights up lighter initially if we've got any discharge referrals it will flash blue.

I: That's cool.

R: Other times we don't notice it until we go on the emails and notice that we've got an email. We are getting better at noticing it.

I: So you get an email to say there is a message, you now get a pharmalarm.

R: So we get the pharm alarm as well to say we've got a message so we can go on and see the messages. And, then obviously click on the message and action it according to what we need to do.

HP12

I: So, when you first got that first one, you are all looking at it, then what happened in terms of that first notification – one of your patients is in hospital presumably?

R: I can't remember whether the first one was one of those or a discharge, I can't remember. But we went through together so that everybody saw the process of how to accept it. I think it was an inpatient one and then about two days later they actually came out again so we could all see the process through quite quickly. Because obviously you get people going in and they don't come out for weeks.

I: Yes quite.

R: So, that one was quite a quick one so we could all see and we could show the staff and the pre-registration pharmacist how to view the discharge letter, how to get that information, and what to do with it once we'd got it.

I: And once you'd seen that was that, you know, was it pretty straightforward?

R: It was fairly self-explanatory, I don't think, if we hadn't have gone to the training meeting, I don't think it would have been quite as straightforward to follow, knowing where to look for the information and what have you. I think the inpatient ones are very straightforward. It basically says, this patient has been admitted, accept or whatever.

I: Right. So, you would accept, would you then check that it is one of your patients?

R: We would check it was our patient, if it wasn't then we'd return it with an explanation – not our patient. If it's one of our patients, we will then make a note on our files that that patient is now in hospital so nothing else gets done or sent for them until they know and we can complete it and done.

HP12

I: What are the implications for the service for staff, I suppose that brings me on actually, to how it's used here by staff?

R: Yeah. So, I mean for us it's definitely made the process easier, because you know you've got the correct information and the most up to date information, you've got it quickly. So, if we get a discharge through we would then compare the discharge medication to what we've got. Since we've been to the second training event, we're more aware of looking at where they've actually been discharged to, because sometimes we'll get one but actually, they've gone into a nursing home.

HP12

R: Yeah, definitely. And I think, so the more we are getting and the more used to it, and we love the inpatient notification, that's fantastic.

I: Why is that so useful?

R: Because we prepare them all in advance and they are all ready to go out for delivery and normally if you don't find out for however many days, that pack could have gone out for delivery, the delivery drivers might have delivered it, left it with a neighbour, husband, wife and then there is a danger that that is still sat at that patient's house when they come home, with old medication in. Whereas as soon as we get the inpatient notification now, it's like, right remove all of that patient; put them away in a separate area. We leave it all up there until we know that they are back out. So, that's a definite plus for us.

HP12

I: If there hadn't been changes on the discharge, that would be okay to go?

R: Yes, unless it had been sat for months and months and it had degraded. But yeah, if it was only two or three days, you know or a week or so, and we've had packs made up that were all still the same, then we would just carry on supplying as normal. Although it's very rare that there's no changes whatsoever.

I: Yeah. Where's the benefits there then?

R: Just speed and accuracy of information and patient safety that there is not these things going out that they shouldn't be having.

HP12

I: Yes, but you are still empowered with that information to have that…

R: Yeah, because we got the initial information, we spoke to them, sent them the information so he's then rung and said, I've dealt with it all but I've not done this because of…you know.

I: Yeah, precisely. Is there any communication, apart from the little notification you can put in there as to why you are accepting or rejecting, is there any communication back and forward to the hospital?

R: Not an awful lot. We do find if we are ringing up now it's much easier to say, I've had a discharge summary from such a person, and this is…we did have some referrals the other day, so they were discharge referrals, no they weren't they were inpatient referrals, but they were patients we didn't do a blister pack for. So, we sent it back saying, we rejected it, we don't do a blister pack for this patient, and then somebody from the hospital rang us and said, they had been assessed, we feel they need a blister pack that's why we've referred them to you.

I: Okay.

R: And then when I did look at it more closely it did sort of say, referred for new compliance aid. So, it's all still a learning process. We kind of, just looked at the name and went, no we don't do her, reject, you've picked the wrong pharmacy kind of thing.

I: Yeah.

R: So, it's all still learning and the fact that we'd had that phone call, now makes me think, right when we get one that I don't know, I'll check that it doesn't say.

HP13

R: Well, that was before we got the PharmAlarm, wasn't it, yeah. Well, we just used to have to log on to PharmOutcomes every day but as it happened we do that a lot anyway so that wasn't a problem. But we didn't see the discharges as quick as now. Anyway, we'd log on and then look in our services section to see if somebody had gone into hospital or not and then we'd do the same thing, put a note on and then when we get the discharge, well, I print that off and then I go through it, reconcile the medication with what prescription we've actually got and I phone the GP if it's wrong which invariably it is.

I: Right, if that's a script that's already come from the GP.

R: Yeah, it might be theirs, if there's changes, more than likely...it's only if they've gone in and there's…they're out of hospital, everything's the same then you can use that prescription. But more often than not you will have to then…this discharge letter, as I say, print this off.

HP13

R: Yeah, go through it. I like the PharmOutcomes thing because this actually makes you go through it in a systematic way. See, because when you've got the discharge now, you have to actually say what you've done and tick boxes, you see, which is marvellous because before, I did it but I wasn't acknowledging to myself or anyone else that I did it.You know and it's saying, the first question is have you reconciled all the medicines and that's the first thing you actually do which we always did but now it's making you do it, it's making you declare that you did it and it's asking you have you got a repeat prescription, which is good. You have to look and see have you and then they ask you is it correct. And then they ask you have you had to refer anything back to the GP which again, if I do, which I often do, then I have to obviously press a yes.

I: Is that all within PharmOutcomes?

R: Yeah, it's all in one page, it's excellent and there's notes to say…you can put what exactly you had to refer to the GP and why you had to and what the GPs done about it. This is obviously marvellous.

HP13

I: And it's there within. And you've now got the PharmAlarms, haven't you?

R: Not everybody, no, not everybody. Only people that went to the recent meeting got one, I'd got one and that's even better, that's fantastic because we did open PharmOutcomes every day so we did know if people were in hospital but we know more instantly now.It's just plugged, it's at eye level, got it plugged in to an eye level usb so we immediately see, all the staff know if it's orange that means we've got something, get PharmOutcomes up. Well, actually you can go in…there's an app anyway where you can go directly into PharmOutcomes, once you see it's orange it takes you to your login page. It's all very handy.

I: Yes, and then just log in and see.

R: Yeah, 'cause we could just be doing that person's prescription, for all we know, and then the light comes on - excellent. Whereas before the PharmAlarm, it did rely on us opening PharmOutcomes, which, as I say, we did but only in the morning and maybe again in the evening. If they went in at four o'clock in the afternoon we wouldn't know maybe 'til the next day or something.

I: Yeah, precisely.

R: This PharmAlarm is good, it's very good, yeah.

HP13

I: When it came here, who here uses…is working with that e-referral, obviously yourself, is anybody else involved?

R: Oh, yeah, I'll get the staff to…it depends what I'm doing, if I'm busy checking things off, I'll print off the discharge and I'll get one of the staff to reconcile it and if she's happy that it's the same then I'll double check it myself but sometimes just reconciling it with what they've already had can be a little tricky job but they can all do it.

I: And do you have someone who does the blister packs here?

R: We all do them, yeah.

I: You don't have some dedicated work….

R: No, we take turns 'cause it's a bit of a tedious job so we know some girls do it some days and others do it other days but they can all do this reconciliation thing, yeah. And if I have a locum in, he can do it as well 'cause I stick to the same locum mainly. He's fine with printing off the discharge…

I: So it's just yourself as the pharmacist here but you have locum pharmacists coming in?

R: Yeah.

I: And you've got, what, technicians?

R: Yeah, I've got technicians and a couple of dispensers, yeah.

HP13

R: Well, it's not hugely busy but it's sort of, medium-ish ones, 'cause we've only got the one, it's not hugely busy.

I: Yeah, who do you think are the important people to the pharmacy referral service, who do you think are the most…the crucial people to make it work, as it were?

R: Probably in some ways it would be the dispensing staff, you know, because as the pharmacist you may see that a notification has come through but you maybe haven't got the time just then to open it up and really view it; it's possible that you get interrupted, you're checking something, whatever. I don't know, possibly but then I have to oversee it anyway as a pharmacist so that I'm still involved. It's a team thing, really, isn't it.

I: Yeah, that's interesting.

R: But in a way I probably rely on my staff to alert me that something has happened in the first instance and then we open it up and have a look at it.

HP13

I: In that way the service and other things here, how has it impacted your work, how has it changed your work, that is one of the ways it has changed your work. Has that caused some extra time involved there as well?

R: A bit but not really 'cause, you know, a bit not not hugely, not huge amounts, no, I wouldn't say it does really 'cause I mean, I've still got to deliver to the patient their new pack anyway, I always would've done that anyway. I'm maybe saying a bit more when I get there but, you know, you're only talking maybe five minutes.

I: Yeah, just to say are you happy with or do you know what you've changed, are you happy with this new medicine, yeah.

R: Yeah, and explain if they ask and maybe a quick phone call early in the week to tell them that we'll be coming, you know, a two-minute job or I'd get the staff to that, two or three minutes here and there, it's not huge. And the PharmOutcomes thing, it's just tick boxes mainly unless you've had to write a little sentence about what you said to a doctor and that doesn't crop up too…I mean, it does crop up but I make it brief, I don't go on and on and on. I make as little typing as possible in it, just clear and precise, yeah. No, not really, I don't know, maybe ten or fifteen minutes' work.

HP13
I: I was going to say is it the system itself, you said that check…be able to having those checks through it or is it the fact that you've got that information itself that drives that?
R: It's both those things; it's having more information and also the PharmOutcomes, making you, as I say, go through it in a logical way and making you realise what should actually and what could be done.
I: It's the steps of what needs to be done, yeah.
R: I think it's those two things really, yeah.

HP15
I: So, what's…yes, what's the way things work now then with the new system?
R: Yes. So, with the new system, when we receive it…when we first receive the notification that the patient is in the hospital, afterwards we know so we just follow our processes and mark that. But when we get the discharge letter on their discharge, we check with the patient if they're home that day. Because sometimes they send the discharge but they are still waiting for their medicines and they are not back home.
I: Right. Yes. Yeah.
R: But at least we can start our process and check with the GP surgery the next day. Well usually the day, because we are not always sure when they receive the discharge letter either. So, we leave a day and then we check with the surgery if they've received the discharge letter, if there are any changes. And then we request the medication accordingly. According to the supply that they have had. Yeah. But we just then check with the current medication if there are any changes when we receive it. And then after two weeks, if there are any more changes, again we complete the online request and say, yeah, everything is fine and we have received the request. We have checked with the patient, their MUR or whatever needs doing.

HP15
I: Yeah. I mean that's actually one of the things…we're moving through these questions, is one of the things that basically, how do you actually use the e-referral as part of your service. Could you perhaps talk me through what happens from the moment you get that notification that someone is the hospital? What do you actually then do?
R: Okay. So, once we receive it, we have to open it and accept the referral and complete it. So basically, it's just to say that, yeah, we have received it. And then we mark our PMR system to say that the patient is in hospital, so we don't dispense any medication while they are in the hospital or even delete their retrieval request. So, we don't send the request, yeah. And so, every time someone would go in their record, it will tell you the patient is in hospital. So, we don't do anything with them, yeah. So, until we receive another discharge letter or something, information from the online referral, then we'll accept that and complete whatever it says.
I: Have you got the little PharmAlarm with the PharmOutcomes?
R: Yes, we have. But we've just misplaced ours because we had a computer before here. So, when we had our new computer setup, we've misplaced it. So, they are sending a new one out to us. We just had a call today about it yet. Yeah.
I: Right. Brill.
R: Yeah, we were using it until three weeks before, but we misplaced when everything moved about.

HP15
I: Yes. Right. So yes, you've got a notification, then you log into PharmOutcomes and see. So, you don't do anything…once the patient's…you know that patient is in the hospital, that patient's medicines are set aside, you don't make up blister packs or anything like. Then what happens when you get the notification of discharge from them, for them?
R: So, we print out the discharge letter and go through all the details. It's very useful in this way. So, we know what the patient was admitted for as well and what the results are. Before this, we couldn't see any of this, and when we check with the patients sometimes, they are confused. They don't even know all the medical terms. So, they wouldn't know the exact problem.

HP15
I: Yeah. And then when the prescription comes through, you make up the new…the new blister pack and stuff.
R: Yeah. Yes. And then send it off or they come and collect, depending on the patient, what it's like, yeah.
I: Yeah. Yeah. So, what percentage are delivered out of…do you get patients coming in collecting the blister packs, as well as being delivered out?
R: Yeah. Yeah. So, I would say it's about 40 to 60. So, 40 per cent deliveries and 60 collections.
I: And was that collections from the patients or carers or families?
R: Carers or family, yeah. Usually when they have been discharged, patients themselves don't come as much. Because they are not feeling well. So, it's carers or friends or relatives or someone will come here. But most likely, if they are around, we will deliver for them if they like, the first few times, yeah.

HP16
R: I think it was quite rigorous and we got to a provider which was interoperable, which was the key bit so that the referrals were quite seamless, they became part of everyday workflow, or they could become part of everyday workflow. So what we wanted was something like that if the system in the hospital could handle it. But also if the hospital...

HP16
The key bit for us, I think, was that actually we wanted a system which doesn't have too much of any impact on workflow, it's not onerous, because when it becomes onerous it doesn't get used. But the system has the capability of being used at a very basic level where, say, a hospital doesn't have...you know we've got varying levels of record across (name of place) completely, to people have separate PAS and EPR system, to people who have half paper, half electronic records even, but the system can work on all of those.

HP16

R: I think not as much as we probably would have hoped but it's helped both sectors understand some of the pressures being faced. But I think, on the converse, from hospitals and community because we used to hear a lot of, well, I don't understand why this can't happen or I don't understand why this is a problem when it's not a problem in the hospital. NHS purchasing and all those sorts of things but there's an understanding of the differences that the two sectors have. And the pharmacist in the hospital can go and green pen things very, very easily whereas we can't. All those sorts of things actually they would very much go and do something and change a prescription and if it was a brand they could make it generic and change a capsule to a tablet, no problem. Whereas we have very, very restricted and can't even change formulation. Until obviously now with the SPS stuff coming through but even that's in itself in its own little...with caveats.

HP16

R: So when we first get notification we're literally quite quick, we're quite responsive to pharm outcomes because we are on it all day and it is part of our normal workflow anyway. So if somebody sees something they will either flag it up, the emails will be checked anyway. And that was commented on by the hospital that we were actually the most responsive to accept notifications, so that was fed back to us as well that actually you are very quick at accepting things on there. But for us it about as soon as we get the notification it's just a team chat really, this person's in hospital can we stop, so we'll put a note on their MDS and we'll just have a quick conversation around is there anything that we need to consider about that patient? So, for example, controlled drugs or prescriptions expiring, returning prescriptions, buy in, et cetera, et cetera, we would do all of those processes.

HP16

I: So what happens then when you get that discharge?

R: So when we get the discharge we accept the discharge, we'd compare the discharge to the original prescription that we had or the information that we have, see if there's any differences, see if there was a mention on the started/stopped section. We'd have a quick glance over the discharge as well to see if there's any relevant information for us and see if the patient's able to come in, so we'd try and get them in for discharge MUR, for example, or see if there's something else that we can do to support them, make sure that there's nothing changed in terms of them. If they were collecting, for example, but they'd had a hip operation then they're going to be housebound for six weeks, actually do we think about delivery, do we think about another option, a carer coming in to collect, a family member coming, all those things? So we have a quick conversation around that as well and then if there's any discrepancies we try and let the GP practice know this patient's out of hospital, can they prioritise discharge and they need prescriptions for that so that we can get our process ready, especially if there's significant changes.

HP17

And before this…this was…this chemist, I've been here since 8 May and it's the first time that I've seen this electronic discharge sheet through PharmOutcomes. So, before, if I go back to when I used to work in the area of (name of place) way, dealing with the surgeries in (name of place), or looking, when I was at my other branch for a few years…because I was in (name of place) for ten years and then in (name of place) for two and a bit years and dealing with surgeries in (name of place) and round there, all those surgeries, and you might get no discharge sheet. You might get a discharge sheet. You might get a call from the hospital saying they're coming out but then a discharge sheet doesn't come. You might get no information at all and the patient just turns up and they've run out. And we're constantly trying to educate the patients, if you go in hospital tell us straightaway, or as soon as you come out of hospital don't leave it until you've run out, tell us the day you come out that you've come out, bring your discharge sheet in to show us, bring the tray in to show us if you've not got a discharge sheet. And then on the other side of the coin we've got to liaise with the surgery and say, can we have a copy of the discharge sheet? Okay. In previous cases they might have faxed it. Some cases you might have to physically pick up a copy from the surgery. But the latest is if you don't get it through PharmOutcomes will be, have you got a secure email address? So, with the advent of NHS Mail they'll now email us the discharge sheet, usually, but you might get some surgeries that are difficult, like the one that I dealt with in the area of (name of place). That was when the practice manager was very difficult, refused to release any information, and that was what really annoyed me at the time where she said, well the doctor's done the prescription, it will be right. How do I know?

HP17

I: So, it's not much, much later. Were you given any sort of…when you got here was there any training, was there any…were you told anything about it, or…?

R: All I was told was that we get electronic discharge sheets and it comes on PharmOutcomes, and then my supervisor will print it off, but I've now made sure that the main dispenser that does the trays that she knows how to print it off and look at it. I mean we monitor emails multiple times a day. We're on PharmOutcomes multiple times a day. So, we're constantly looking and as soon as the…it comes through, you know, we can see. If you're bored you can even monitor it on home at the weekend if you want to and log in and say, oh, so and so's been in hospital, and then you're prepared for Monday then, you know?

HP17

R: So, an email comes through on our email system and that flags as, there's a message on PharmOutcomes. So, then we'll log into PharmOutcomes and it'll say, we've got an inpatient. So, one of our patients is…that's on a tray is now in hospital. So, then we need to identify, has a…is a tray…first thing, when's she next due a tray? Is a tray going out today? Yes/no. So, here's your little flow chart. So, is it going out today? Yes or no. When is it next going out? Right, grab this patient. Each patient's got an individual special box, a file box. Grab that. Put them on the hospital shelf. Put the printout from PharmOutcomes in that box. Put everything on hold. Once you're on the hospital shelf you just…that's like the on hold shelf. And that's it really. We've just got to identify and put them on hold until we hear about a discharge. So, everything just stops and they go on the special shelf.

I: And then at discharge?

R: At discharge then email comes through on our computer to say, a message is on PharmOutcomes. We then log into PharmOutcomes. PharmOut…prints…accept the information on PharmOutcomes. print out the attached discharge sheet. and then pull the box back off the shelf from PharmOutcomes. Right, there's (name). This is our (name). She's the tray expert.

HP17

R: Not that I know of, not that…there's no specific area on the discharge sheet that would suggest that.

I: Would suggest that.

R:	I mean there is…when I accept the discharge there's a bit at the bottom that says, have I actioned it, have I just actioned it and accepted the information, or, have I have actioned it and I've done an MUR, or, and I've done an NMS? There's a bit [for flicking 00:07:53] but at that point I don't know yet.

HP17
I:	Currently they just send out the e-referrals for people who are on blister packs, or Venalinks, or whatever. Do you think it would be of benefit if it were other patients as well, and, if so, which groups of patients do you think would benefit?
R:	So, yes, it would be a benefit. We've just got to be careful. You don't want to flood the system. You don't want to log into PharmOutcomes and have 20 referrals and then you've got to do that. That's when you'd have the time wasting. If I turned on PharmOutcomes and logged in and I wouldn't know which was a tray and I've got another…I've got two trays in there and I've got 30 other patients that have been discharged but they're just normal patients and we don't manage their medication. So, we do have the repeat system where we order people's medication and they verbally, or physically written, confirm what medication they want in four weeks' time, or eight weeks' time. It would notify us, so we know we could cancel that and put a note on the computer, they're in hospital, but that's when you get to the limits as well, you're creating more workload. So, we're not…I think just doing trays is great. I think you'd need to think very carefully whether you'd want to do more than trays because that would then create more workload. I mean there might be…if there was another way to separate the referrals, if they're just normal community patients that are on medicines, and not mix that up with the trays and you could log into some sort of system and know that they've been in hospital. I mean you've got the summary care record but then you need to have permission from the patient to access the summary care record. So, I mean you've got to let…where they're talking about letting pharmacists look at GP records and sharing records with pharmacy. So, it's something for the future where everyone's got access to everyone's records, well community pharmacy can access GP records, and it's all streamlined and you're all on the one system, that will be great, but you have got summary care records but then you need permission to look at that, unless it's an emergency.

HP2
R:	Yeah. Anybody who does medicines reconciliations for the patients.
I:	Right. So, what…you do the first bit at the admission. What happens then on the discharge then?
R:	Right. So, when a patient comes in, we will go through a medicines reconciliation. So, we will go through all their meds, what they have and we'll do the e-referral. On discharge, if a patient is going home, the nursing staff or the doctor will let us know. If they need anything…because a lot of the time they will bring medicines in with them, so, we don't have to re-prescribe and we don't have to send them home. But it's if they need antibiotics or pain relief for anything new. And then once that discharge summary is done, then that should go automatically to the chemist via e-referrals, by PharmOutcomes. Yeah.
I:	So, in terms of, you know, what you then do, you do the meds rec in the beginning…
R:	Yeah. We do the meds rec…
I:	But then you do, any changes.
R:	Yes. So, if there are any changes throughout their hospital stay, when the doctor writes the prescription or electronically writes a prescription, that will then be clinically checked by a pharmacist. So, they'll check that there's nothing wrong with it. If there is anything wrong, they will get them to change it straightaway. They should then do the changes electronically and then that can go to the chemist. And one is given to the patient, the chemist and the GP, I think. Yeah, all three. So, they all have a discharge summary.
I:	Yeah. I think most of the information goes to the community pharmacist. Do you think it should be going to the GP there as well?
R:	Definitely. Yeah.
I:	Yeah. That's really interesting. Yeah.
R:	Definitely. Because on the occasion…some occasions I'll ring the community pharmacy and they've not had…they have changed something, but it's not on the patient's SIR record. So, there's a discrepancy…why does he not know that but they know the changes. So, I think the GP definitely should.

HP2
I:	Has there been any problems with…you know, for instance knowing a patient's nominated pharmacy, things like that? Have there been any glitches in the system at all?
R:	I think…I don't know if some people have come across maybe it being sent to the wrong pharmacy with the postcodes and some pharmacies being practically next door to each other.
I:	Yes. Yeah.
R:	So, they'll have the same postcode but…so, maybe that may have been a glitch but no, no others, no. I've not come across any.
I:	And patients have a nominated…most patients have a nominated pharmacy.
R:	Yes. Most patients, yeah, have a nominated pharmacy.
I:	Is that because of the nature of those patients? Because they're on Venalinks?
R:	Yes, when they're on Venalinks they have a nominated pharmacy to make up their Venalinks. So, they've got, like, their…not a contract but a little [con…so 15:47] they might deliver it to them, they get to know the patients, they've been going in there years. They have a nominated day they know, you know, if that patient goes out shopping on a Friday morning, they'll deliver it in the afternoon. So, yeah.

HP2
I:	You talked earlier about the potential for, you know, discrepancies between the GP list and the pharmacist's list and the patient…what the patient thinks they are on, you know, which, you know, can often happen. Do you think this can help?
R:	I'd hope so. The thing is with the patients, they'll come in with a particular blister pack and we'll say, you can't use that one, it's been changed. We will provide them with a new one, which we do for a week, but then they may go home and they have a blister pack lying there from a couple of weeks ago…
I:	Yes, they got a box.
R:	…and carry on using that. So, there's got to be…somehow, we've got to get through to that patient, where if it is not in hospital, it has got to be in primary care, where they are up to with the medications. And I think if we are all on the ball, the chemists, the doctors and the pharmacists here…the hospital pharmacy here, we can bet that patient can benefit, but they…I don't know.
I:	Yeah. Because, yeah, what you are saying is that that if you change the Venalink and they end up…they go home and they've got a different one or there's…
R:	They've got a different one at home, they are going to carry on taking the other one until that one is finished.

I: Or the GP isn't aware that things have been changed.
R: The GP isn't aware that things are changed, so they will do in three weeks' time, you know, when the letter comes.
I: Yeah, when the letter comes through.
R: But like with the e-referral, the chemist should know straight away. Well, they'll get to know straight away and should prepare that next time.

HP2
R: It's brilliant. Love it. Yeah. It's really, really good. Because it has freed so much time for everybody and it's quick, easy to do. And we do it…it's actually in our medicines reconciliation, like, form now on the computer. It was separate before but now you start your medicines, reconciliation it is there, right in the middle, just click on it and yeah, it's gone.

HP3
I: In some respects, you've covered some of this in some of the things you've already said, but the next bit was really just to talk about how you use it as part of your work. So perhaps if you just describe to me what you do, in terms of a patient's admitted, from the moment a patient is admitted, what will you do, in terms of the e-referral then?
R: Oh, with the e-referral?
I: Yeah, so from the moment a patient is admitted, what do you then do?
R: So we'll do a drug history, get the patient's medication. I'll ask them, is it okay if I contact your pharmacy and let them know that you're here and can they have information about your discharge summary? Ten times out of ten, they always say, yeah, that's fine. And then I will type up the drug history. And we've actually changed our medicines reconciliation document now, so it includes the community pharmacy referral, so there's a question saying, compliance aid, yes or no? If it's yes, then it will come up with the e-referral link, and so we just click on that, search the pharmacy and put it in.

HP3
I: And then you do your stuff, but then it just drops down... Oh, right, that's... So it just takes you...
R: Yeah.
I: ...via that. But if you answer yes to that question, it then just takes you to the system.
R: Because before, what we had to do was put it in as an order, so it's similar to the orders where the doctors do the prescriptions, so we'd have to put it in as a community pharmacy referral and then... Which, I mean, it's not much longer but it's just easier when you're doing the medicines reconciliation, just click yes, and then it will take you straight to it.
I: It takes you straight to it. And then what happens?
R: So then it will come up with the pharmacy search, you put in the pharmacy, and then, at the bottom, it's like a consent, saying, I have told the patient, they've consented, or, I was unable to confirm with the patient if they're okay with it, and then you just confirm and then it sends, so it's just like an automatic...
I: So it then sends that the patient's been admitted and what's happened?
R: No, I don't think it sends that. I think it just sends that the patient's currently an inpatient, so I don't think there's any information of why they're in...
I: There's no information, just that they're in.

HP3
R: Yeah, I'd say. And I'd say it's even been helpful in other cases where the patient doesn't even have a compliance aid, because there was a patient I had a couple of weeks ago who was a methadone user. We tried to get in touch with the pharmacy, but I guess their line was just busy or it was just going straight to voicemail. And that is an important case, to make them aware that this patient is in, so that no one else collects their prescription in the pharmacy.
I: Yeah, precisely.
R: And so I sent through a referral to the pharmacy after consenting with the patient, just to make them aware that they're currently in, even though it wasn't a compliance aid, it was just so that they were aware that the patient was currently in.

HP3
I: Yeah, precisely, so they know it's not going to be collected, and so on, which is really important. Beyond then just those sorts of things, are there any other reasons why it would be good to have it used more widely, or are there any challenges in using it more widely, do you think?
R: I'm sure there are challenges using it more widely. I guess one of the challenges would be, because we're inputting into the system, this is the pharmacy, and then we send through the referral, if we send it to... Like I said before, there are so many (name of pharmacy). Say I send it to the (name of pharmacy) on the other side of town and they've got all of this patient's information. I'm not sure, I think on their side they have to accept or reject, they can accept or reject.
I: Yeah, I think there is something, but I think if they reject, I think they then get phoned up by somebody, or something.
R: Oh, okay.
I: Something happens; I'm not sure.
R: So I'm not sure how it works on their side but I guess it is a case of patient confidentiality, because I've sent it now to the wrong pharmacy, and is that an issue with that? Say it was as pharmacy that the patient used to be at but they no longer want to go to, and they don't want them knowing any of their business.

HP3
I: In terms of the service, then, have there been sort of technical issues? Has it had any impact upon working relationships, things like that? What are the problems that this has created, or are there any problems?
R: I don't think there have been any problems. I guess, at the beginning, there were some pharmacies that I didn't realise were in the area for referrals so I just put them through as fax. And then (name) told me, oh, that was actually an e-referral and I've sent it through. No, I don't think there's... Because I think at the beginning she had a list, she sent us all a list of all the pharmacies.
I: All the pharmacies, yeah.

R: But then I was thinking I'm just going to assume the (City) ones are (City) patients, so that they've got (City) pharmacies, because again that would be me spending time going through the Excel spreadsheet to find if they are a (City) pharmacy. But I think I've gotten so used to it now that I know what I'm doing with them.

HP4
I: So that communication, how can that help with that safety or how is that going to help…?
R: Because inevitably I see it all the time when patients are discharged from hospital, we see so many problems with mistakes, errors, prescribing errors, when patients go from one setting to another, when they go from primary to secondary, secondary to primary, you're constantly facing that battle of mistakes in that transition, and the aim really is to make sure it's communicated reliably. So if the chemist are getting an electronic discharge of like a snapshot of exactly what they were discharged with, that takes out that kind of element of, you know, they've got the information there so they can then hopefully act on that quickly, much more quickly than relying on a fax two days, three days later where there used to be quite a significant delay, things can get changed quicker, so if it's blister packs they can get their new blister pack up and running, they can nag the GP to get the new prescriptions through. So hopefully the next time the blister pack comes out, it will have the correct medication in, not medication from like preadmission.

HP4
R: Yeah, so this week we've just recently had a new meds reconciliation document version, I don't know if you've heard of that, but that now links to the e-referral, which is really good, because…
I: Right, so this is an electronic document, yes.
R: It's an electronic document which pharmacists and technicians use to basically – it's like a stepwise document you kind of go through, and it's all about accurately documenting the drug history for a patient, so when they come in it says, which sources have you used, and it will be GP prescription, patient, patients on medicines for example, so you'd tick it through, you'd work your way through, it's, does the patient have any medicines with them. And then you document the drug history, so you write whatever they're on at home, and then it says, is this – I can't remember the exact kind of terminology, but it says something like, can you complete an e-referral, do they have a compliance aid basically, and if you tick yes, it automatically brings up the box for the e-referral, so you can click on that and it exports you to…
I: And then just takes you…
R: …yeah, takes you straight through to the e-referral, which is good because it means you're less likely to forget to do it.
I: Yes, so you haven't got to think, oh, that patient's got a Venalink…
R: No, so…
I: …I need to do…
R: …you did last week, but since this new kind of document has been introduced…

HP4
R: But so anything that can help, you know, is a good thing.
I: Is improve…
R: It's a complex…it's really, you know, discharge, admissions and discharge is really complex business, isn't it, in terms of… And some of the factors, you've got carers, patients, relatives to throw into the mix on top of all the other stuff, it's just…
I: And finding that communication…
R: The loop, yeah.

HP4
R: Are continued, yeah, that's right. The only…I guess the only downfall slightly is that our system doesn't necessarily show that it's had a pharmacist clinical check. So if a patient's discharged but the nurses or the doctors don't tell pharmacy about the discharge summary, that discharge will automatically still ping off to the community pharmacy, but it might not have had a clinical check, so there might still be errors on that. That's one of the big risks I think in our kind of system…
I: Because unless they make any changes, they don't need to involve you, is that what…?
R: So we try to encourage doctors and nurses to inform us of all discharges basically so we can cast our eye over it, because a lot of the time you do see doctors writing, no changes to medicines, or… We did a recent audit on EAU where we found we changed nine out of ten prescriptions in terms of documentation and prescribing. So if you imagine that and if you imagine that say out of hours overnight if pharmacy aren't open or if a nurse just says, oh, there's no changes, I'm not going to bother telling pharmacy, we'll just discharge the patient, and then that automatically goes off and those errors are continued.
I: Errors can carry on, yeah.
R: That is still a risk, but it's difficult to get around that, so other than encouraging staff to inform pharmacy of all discharges, problem is pharmacy aren't there 24/7, at the moment we just have 9:00-5:00 really, 9:00-7:00 on EAU, so that is still a risk. So the problem with this e-referral I guess is that you can't guarantee it's necessarily going to be correct. If a pharmacist has been involved, you'd hope it was correct, but you don't necessarily… So that's why we annotate I guess if we've seen it or not, if we've clinically checked it, it puts our electronic stamp on it to say, we have clinically checked this. If it doesn't have that and there's errors, I guess the community pharmacy could then ring and say, is this right, has anyone actually seen it from pharmacy, is it…
I: Because you do the order at admission, if you then don't get involved in the discharge, it goes without…
R: Yeah, that's it, it goes. It goes, yeah.
I: …you having anything…
R: That's right, yeah.
I: And it's about keeping people…
R: It really is, yeah, so there is still an element of communication and unreliability to some extent. It's just better than it was. Yeah.

HP4
R: That's the main niggle I have with the whole system, but it's like an EPR system generally, it's not the e-referral system, it's just the whole discharge prescription itself, the discharge process in itself, it's still an active prescription even if a pharmacist hasn't seen it.

HP5
I: Right, okay. And what do you know about the pharmacy e-referral service that's been introduced here, what do you think it's there for?

R: Well, I'll tell you what, I was very excited when I first heard of e-referrals. Only because within the unit where I work, the units, if a patient is discharged or if they arrive, one, is I don't know if they've been contacted by the hospital prior to transfer to my facility, that's the first thing. That information is not necessarily in the medical history, the medicines history or medicines reconciliation, that's the first one. And the second thing is, is because I work between units my contact with the community pharmacy that if they had a blister pack, for example, would be via fax, via fax. But quite often they would get discharged and I wouldn't be at that facility and I missed the opportunity to actually inform the community pharmacy of what the patient has actually been discharged with. So, the beauty of the e-referral, I'm not being funny, but the beauty of it, is that firstly we get to inform the community pharmacy that the patient is admitted into hospital, they get to know that electronically. And the second thing is whether or not I'm at the facility, once an e-referral has been setup they get a discharge summary once the patient goes. So, it takes the pressure off of me, trying to remember whether or not we've informed community pharmacists, et cetera. I've got a visualisation, I can see now whether or not a patient has had an e-referral, and I know that's going to go once they get discharged, so that takes the pressure off of me, I love it, I absolutely love it.

HP5
I: Yes, perhaps moving on, could you describe because I think we are getting towards that that this next question, how you use the service as part of your work, what do you do?
R: Right, when I'm doing a medicines reconciliation the majority of the patients who actually come in to the facility where I work actually use blister packs. So, when I'm doing the medicines reconciliation it's one of the questions that now come up with this new version of medicines reconciliation anyway, whether or not a community referral has been sent. But when I look at all this, I look at the bottom to see if one has been sent because there will be times where perhaps a patient will come in and they've not been on the ward long enough to get a medicines reconciliation, then come on to our facility. So, I do check to see if…I ask the patients if they use a compliance aid and I also asked them where they get their compliance aid from. And then show that the community pharmacy's referral is either in situ or [inaudible 0.12.46], or I actually do, put the e-referral on myself. It's not that many that come through without an e-referral to be honest.
I: So, some of the, for some of your patients because they've come, they are in that sort of stepping stone out of hospital, some of them would have already had the e-referral happen in hospital.
R: Yes, the majority would have.
I: Right, but you check that that's the case.
R: Yes, but I check to make sure that that's the case.

HP5
I: So, but it's moving from here to community pharmacy, is there any transfer of information back from the community pharmacy to yourselves, or to here?
R: No, no.
I: Do you think there should be?
R: It would be helpful. I suppose the community pharmacist will deal with us on a need to know basis. I mean at the end of the day if you want information from the community pharmacy, we will have to initiate it, you know. So, it would be great if the community pharmacy could let us know, have a complete history of what a patient has had from them, but it's never necessarily complete because patients are not necessarily loyal to community pharmacy these days either. So, the best source of history of medication will be via the GP surgery, where we already have access to the SCR anyway as to what has been dispensed, and we can just confirm that information with the community pharmacy. Having said that telephone systems are prone to being busy, a lot of people are using telephones to contact pharmacy et cetera. Whereas, if you can get an electronic confirmation of information safely that you need, then that could save time.
I: Do you know if it's been received and when you, when you send that through do you know that community pharmacist has gone oh yes, that's my patient.
R: Yes, because I phone them.
I: Oh right.
R: What I did, I'm not one for using a system and then assuming that it's worked perfectly.

HP5
I: And that's just a chat, as you, it's a bit like your telephone, looking to [inaudible 0.28.51], it's checking it's all working through isn't it. It's a sort of interesting, sort of, evaluating of it that it's all sort of happening. One of the things that we, you know…currently it's used purely and simply for the blister packs. Do you think it should perhaps be used, sort of, more widely for, you know, discharges more generally, what do you think about that?
R: It's safe to use that way, it is definitely safe to use that way. I suppose the supply of original packs, let's say patients are not using blister packs, these patients tend to be not so loyal to their chemist. But I must admit I have used it on occasion. That…one thing I forgot to mention within the facility is that sometimes we start a package of care. Package of care implies that we have carers come in and they will only get medications from blister packs, and therefore we need to initiate the supply of the blister pack.
I: Right, you start them off, yes.
R: So, we need to find out from the patient which chemist they normally use, and then set up an e-referral as well. So, sometimes we used to initiate rather than continue as well, I've used it for that basis, providing the chemist agrees to continue a supply of blister packs once the patient is discharged as well. So, we've used it for that, so yes it will benefit us with patients who are actually using original packs as well, because of the number of instances I've had where we've had to initiate the supply of blister packs because of the type of care a patient needed post discharge.

HP6
I: Right, that's really interesting, so basically if when the e-referral goes, the pharmacist rejects it, what do you then do?
R1: What I do, every day, as often as I can, morning time, before I start doing other things, I have a look on the PharmOutcomes, I log-in, see if there's an e-referrals, at nine or quarter past nine, at that time, probably there is none but later on, they start appearing on the system,on PharmOutcomes. So, when I see one of these rejections…[…] So they prompt out, there's a list for all the e-referrals that are pending on PharmOutcomes but the ones that are rejected, they go on the top of the list. So it could be, one, two, three, I've seen three the other day. So I go on each one individually, so I press the one that I need to see what's the reason behind the rejection and try to deal with it appropriately, accordingly, find out the reason. There's like a record as well, so I also try to sort out the problem, which is the most important thing, we need to sort it out, we need to get it sent to the right place.

I:	Right, because sometimes it will be that it's gone to the wrong pharmacy?
R1:	Exactly, well yes most of the time, I think, so far, it was directed to a wrong pharmacy. There are many reasons, if you want me to mention them now. […] So let's start in order, so once I open that rejected Venalink prescription, I find out the reason, I write it on a log and that log goes to (name), the pharmacist whose in charge, to follow up and change something on the EPR, which I'm not allowed to, I'm not authorised to. Once I write it on that log, I try to follow the steps, if the patient is not with that pharmacy, he doesn't deal with the pharmacy, he or she, then I try to find out what the right pharmacy is. So sometimes, what happens, they pick up, on the system, the wrong pharmacy, so because the map, it's not very, very clear and especially under work pressure, people tend to, when they see the area and see that sign of a pharmacy, they click on it, while there is one very close to it, carrying the same name but in a different lot and then mistakes happen.So I try to just make sure, go on EPR, check the right address, check the right telephone number, try to speak to them if there's any need for that and this is one of the easiest things to do, actually. So I just click on the right pharmacy and say that it's done. Well if it's the wrong pharmacy on the EPR, then I have to report that to (name) or the person who is dealing with the EPR thing, who have authorisation to go and change things over EPR. As I mentioned, I don't have that authorisation.

HP6
This is another story, when a patient passed away, for example, we just…they reject it from the pharmacy for that reason. When a patient is readmitted to the hospital, so we send the patient out but he stays less than a week. When he stays for less than a week, a patient is discharged normally with a Venalink enough for a week or two.
I:	Yes, a week.
R1:	Or two sometimes, I think two when the patient is going into a care home, one if the patient is going to his own home. So within a week, if the patient is readmitted to our hospital or another hospital, then there's no longer a need for a Venalink for a medication. We will start the process from scratch. I mean the patient needs to readmit it and then start a new e-referral and then we send a new thing to the right place. So in this scenario, I can just cancel that and we don't need to do anything.

HP7
R:	So, when I speak to a patient, when they are admitted. I find out if they have got a blister pack, I'll find out what pharmacy it is and if it's a (place) Pharmacy, I will go on the electronic system; there is a little link to get to it.
I:	Right.
R:	There is a link so you just click it, you type in – I always type in, the postcode of the pharmacy, because if you type in the name it's not going to map it to the map, so it's better to just type the name and the postcode, find it on the map. And, then I'll send it. Sometimes if the patients don't bring in their blister packs, and I don't know what time slots things go in, I might have to ring the pharmacy, or I will ask the family to bring the blister pack in.
I:	Right. That's interesting because you've got, you are telling the pharmacist at that point of admission to stop making up the blister pack, and effectively that's the point of that notification.
R:	Yeah, don't send it out for delivery, yeah.

HP8
I:	Yeah, precisely. We've gone slightly round those two bits there, but we've gone... The service has been introduced. You started to see these big excel sheet things. So how is that then working now, as it were? What happens on a daily, weekly basis now?
R:	So I get an email every day to the NIPPS generic email address. It's sent at about 4am I think, so it's there when I log on in the morning, and then I check that. And it's not very labour or time intensive now I know where to look, see if there are any e-referrals, there's usually not. So I think since between February and May we'd only had three. In the last few weeks there's been a bit of a flurry of activity and we've had maybe five more.
I:	Why are you getting fewer than the community pharmacists?
R:	So every patient that is discharged on a Venalink, I believe, their community pharmacy, if they have one, if it's in (name of place), will be notified. But then we only get one if there's a query that the community pharmacist decides, needs to be addressed in primary care.

HP8
R:	Not so much. So, as I said, I log on first thing in the morning. I've got my discharge there. I have a quick glance over it. If there's a yes, then I will extract what information I can. Although I do attach the spreadsheet and send it to the lead. I make it as easy as possible for the team because they tend to glance at emails rather than read them. So you can't send an email with two pieces of information. So you can't say I need you to sign up to one of these training sessions. And also, we've got a meeting next week about something else because they won't read the second bit, so you've got to send one email per piece of information. So I will extract the information and say we've got this e-referral here, it is essentially. But then they've got the spreadsheet as a backup if they think I can't understand that, I need more information, they can go on. So, yeah, it takes a few moments to, sort of, extract that information and put it in the email. And then I email, as I said, the lead in that neighbourhood.They do keep timetables of where their pharmacists are. So each of our pharmacists covers a minimum of two practices in the area in sessions, and they chop and change a lot. Most of our pharmacists work part-time. So there's no centralised timetable of where everybody is day to day because it changes so much it would just be too much of a grand undertaking. But I'll have a look at the timetable that each of the leads keeps and, sort of, make a decision as to whether I think it's up to date or not. So if it says, for example, oh, (name) will be in (name of practice) next and I think, oh, yeah, I've definitely seen her recently on a Monday in (name of practice), then I'll copy in her as well and say in the email I think it's (name) but can you just check. And then, as I said, sometimes because of people working part-time and leave, that practice might not have cover for a few days or a week and in that case, I'll also say can you ring, can you check up.
I:	So you know whether it's happened or not as well or...?
R:	No, no, I don't know whether it's happened. But some of the free text information it's, like, they will say a patient has an appointment to see the GP, so I'll include that information. I'll say, look, I think this has been actioned but can you check that the patient has either got the appointment in their diary or it's already happened. And every time they've come back and they've said yes, no, they're coming in tomorrow, or, yes, it's already happened.

HP8
I:	So (name) or whoever comes to you and says, (name), we've decided that e-referrals service needs changing and we think you're the person to change it, so what would you do? You're in charge now, what are you going to do?

R: So I know that one of my pharmacist said to me that it was very difficult to identify the patients from the information given. I don't know what information they need but I would find out and see if we could add that information. Maybe it's their NHS number, which I don't think is included in the spreadsheet, so it's hard for them to identify patients. So that would be the first thing I'd do to improve it. See if I could fix the lag that (name) was talking about as well because then maybe we could action things a little bit quicker and it wouldn't automatically go...Or the community pharmacy wouldn't send the communication to the GP in the first instance, so the GP wouldn't then book appointments in with patients who could maybe see the pharmacist for a better outcome.

HP9
I: That's recording nicely. So just to start off then, I want to start about the e-referral service more generally. What do you think it's there for?
R: So it's if you've got patients getting discharged from the hospital, then they inform the chemist, like if they're on a blister pack, they'll tell the chemist that the patient's been discharged so that the chemist can prepare the blister pack or can at least get in touch with the practice or know at least about the discharge 'cause where we've had problems in the past communicating between…from the hospital to the chemist. Yeah, sorry.
I: Is that what it's trying to achieve, that sort of communication?
R: Between – yeah – between the chemist and the hospital and possibly between the chemist and the GP practice as well, having that communication between them.

HP9
I: So the e-referral work, how does that fit in with the broader work of being a practice pharmacist within the NIPPS service then?
R: Well, it fits in in terms of the chemist…what I've come across so far I think, it's the chemist referring people to us if they have any issues like, they'll say, can you look at this? This is what I've come across so far if they have a problem with the…yeah, if there's a discharge that they've got a problem with like their medications don't match up with the ones on the repeat then they'll flag it up to us and they'll get us to review.
I: Are you seeing many of them?
R: So far I've only been highlighted one that (name) has sent over to me, but the pharmacy had already rang the GP practice the day before. I think because of that system, they'd rang the practice the day before and let me know that they'd got a problem with this discharge, so I had a look at it. So when (name) sent me over this, I was like, oh, okay, yeah, I've had a look at it already, so they must have come across it because of that system and then flagged it up the next day and then…but I'd already sorted it 'cause [inaudible 03:47]…

HP9
R: Yeah, so because (name) I think gets a lot of requests so it's basically she'll send them over but by the time she's sent them over it's probably…it's been done or by the time it flags up maybe on the system, it's already been dealt with. But this is so far what I've come across, that it, yeah, they've been flagging it I would say more often, 'cause usually I would do the discharge and then I would call the chemist but I think there's been quite a few instances where they've called me about a discharge and they've said, well, we've got this discharge from the hospital and the medications don't match, so can you have a look at it? Which is really good that they're being a bit proactive about it because…

HP9
I: Yeah. I think…that's really interesting actually. You're saying there about that three-way communication, 'cause I think that was… Do you think that that would be…if you were seeing electronically, immediately, like they're referring straight away, these patients who are on these blister packs, if you were seeing that straight away, what would the benefits be of that?
R: I guess it's having somebody…as in to get the discharges straight away?
I: Yeah, to get it in a w…the [inaudible 10:29], they get it in an hour…
R: The way they get it.
I: …or two or whatever, pretty instant.
R: I guess it depends who will…'cause I'm…most people on my team are not here full-time so I guess it's who gets this. I think that would be my only concern, would it be the admin staff that would get it and flag it up to us? But I think that would be really useful because then sometimes when you…like I say, what you were saying before? It takes a few days to come across and sometimes you do end up with patients on a blister pack maybe that…if the chemist is not as pro-active about it, that have got to the last day and the chemist has got to sort out the Venalink a day before, which is not…as you can probably anticipate, they don't particularly like that, but yeah, I think that would be useful because then you'd get the discharge straight away and then you'd be able to action it straight away and change the medications and send it over.

HP9
I: Yeah, yeah indeed. Who do you think are the important people in that?
R: The right answer I guess is everybody.
I: That's what everyone is saying.
R: 'Cause everybody needs to do their bit, so the hospital needs to like obviously do the referral and then the chemist needs to act on that referral. And then when they've forward the issues I guess, if it's to me or to the practice staff, then there'll be, there, everyone's got I guess their important and role. Do I have to specify who's the most important?
I: Yeah. Well if you think there's someone most important?
R: I mean, they're all really important. I guess to start that chain you'd need the people at the pharmacist, at the hospital or the technician if they can do it as well, to start the referral process because otherwise the rest of it will not…
I: …not happen.
R: …well, just not happen, yeah. So I guess yeah, I would say the person at the hospital, so it's the pharmacist or the pharmacy tech that does the referral to the chemist.

HP9
R: Yeah, mistakes. Communication is the biggest one. I think they usually don't either tell you on the discharge summary that something's changed or stopped or started. And then you compare the medication list, then it just…obviously, there's one that's on your repeat that's not on the discharge or there's something new on the discharge but they've not told you that they've started it or anything, so you have to…I dig through it because I've got time to actually look through it. That's part of my job but the GPs – some of them – don't have the time or they just look at this part that says medication changes and they go, oh, no changes. And they might just leave it and just mark it as complete. But then actually it's not

matching. But I dig through it. I just have to go back to…because I can access the (name of place) system, you know the EPR system, so I can check to see what's happened there. So that's what I usually do, and then I'd have to submit an incident report because…which I've had to do a few recently. So this is the main thing that I see.

HP9
R: Yeah, so because obviously if it's sending the discharge summary to them and I guess with the blister packs, they will then have a look, the chemists that have been doing that where they have a look at their sheets for their compliance aid blister pack and they'll just compare it and that's when they flag up to see that, oh, actually, they've got Furosemide for example on this. Oh, but this is not already in the blister pack, so that must be a new thing. That's thinking of the fact that the discharge doesn't document anything accurate in terms of what changes have been done. So they will check the list of medication against their list and see if there's anything, any changes. At least that's the hope with that, and I have had obviously instances where they have flagged things up and I was like, oh, yeah, you're right, I'm just trying to sort it now and…which has been quite good.
I: And the other thing is that the patient's going to come out with a blister pack anyway 'cause they're going to get seven days' supply. And that might be different to the…
R: You hope it's not.
I: Hopefully it's not going to be different, yeah.
R: Yeah. You hope it's not, but yeah, that in terms of…I guess in terms of errors as well. You've got somebody else checking it as well at that point. 'Cause I don't always get the discharges, like I said. So if there's a chemist there as well who's getting and checking the discharge, then that's another step as well in making sure the patient gets it at the right time 'cause they'll all go, oh, well, we usually send it on…they've been discharged for nearly a week now, can you sort this out? So they'll flag it up even before, sometimes before we've even seen it, so they'll go, patient's going to run out probably, can you sort it out? Yeah.

HP9
R: Yeah. Maybe people are on a big list of medic…on lots of medications it might be useful I suppose and you could tell them to enforce…I ring patients but again, because like I say, not all discharges come to all pharmacists, it might be that you could…the hospital pharmacist can say, could you reinforce some advice on this medication that they've been discharged with? 'Cause I do come across patients who maybe have been discharged over the weekend and they might not have a lot of information about the new medications that they've been started on. So when I ring them, they find that quite useful, so they could potentially do that with the community pharmacy, say, can you counsel them.
I: Yeah, and that was one of the other things that people were talking about last year with the service, was that this would…the potential for this to lead to patients better-informed about the medicines. Do you think that's likely?
R: Yeah. I think so. I think that's the potential with it, yeah, 'cause again, when you say, rolling it out to all patients, because there's people not on a Venalink but are on ten medications and something would have changed in hospital but then if nobody tells them about it then they don't know. They might have a box of Ramipril at home and it's been stopped at the hospital and if someone briefly mentioned that to them or not mentioned it at all then they'll go home and start taking that again. And if the chemist is not informed of that then they might have a box on the shelf waiting for them that repeat prescription or they'll just give them that and then this will just carry on. It's like a – what's it called – the Swiss cheese and when it's like an error, just yeah….
I: Just going through the hole, yeah.
R: …gets passed, yeah…

HP9
I: Yeah, 'cause if they've been put on a tablet that needs monitoring, yeah.
R: So that bit I do but obviously GPs don't have that conversation with a patient because they do the discharges and they've got obviously clinics and things at the same time.
I: Yeah, and you've got more time.
R: Yeah, I've got more time, so they will just do the…look at the discharge and process it and do send a task to the admin and say, book them in for bloods, or whatever or book them in for a review, but then nobody would have that conversation then with a patient, so you'd be relying on the hospital pharmacist that they might have told them about the changes. They're relying on the nurse as well I guess, telling them what's changed. But, I can guarantee you because I've come across a lot of patients and they've just not had that conversation at all or they might have just briefly mentioned it but when I speak to them they're like, no, nobody's told us anything about this, so I have to go through it.

P1_C1
R2: Which absolutely in the ideal world is what it should be. It should just be a smooth transition and nothing else. What we did find when we came out, when the hospital's blister pack came, there was medication…it was the same medication but over four times in the day.
I: Right.
R2: And, obviously, now that it's come back to MDS it's three times a day.
I: Right, okay.
R2: Which, when you are trying to do like for like into seeing what medications are being taken at what time.
I: Yes.
R2: Now, I understand why they did it in the hospital, because that's how they give meds through the day.
I: Right, so that the hospital pack was four times.
R2: No. This is the hospital pack, which is why I kept it as well.
I: Which is slightly different. Yeah.
R2: So, as you can see.
I: Right, four times a day. Whereas that one, there is nothing on the teatime one.
R2: Yes. Nothing on the teatime one. So, trying like for like.
I: So, is it the same.
R2: Which I did speak to the district nurse that came round to say, right, are these the same because again, the appearance is different.

P2
I: Have you talked to the chemist at all about it, or has anyone talked to the chemist?

R:	No, because you see, I used to get it from…because my doctors is at (name of surgery), and I used to get me medication from there, but when they put it in the blister pack they moved me to (name of pharmacy). I don't even know where (name of pharmacy) is, I haven't got a clue where it is, so they're just sending these now from there and I don't know…
I:	(name of pharmacy)?
R:	I've no idea where it is. You know, I haven't got a clue. So, it just comes from (name of pharmacy), and that's all I know.

P2
So, when you left the hospital, did a pharmacist, apart from (name), come around and talk to you about your medicines at all?
R:	No, nobody came up and said, oh there's, you know, your medication. I mean, like when I left the first time the week before, you had to wait so long for the medication and they know in the morning, I understand they've got thousands to do, but you know, I'm waiting, I come home and then have to rely on somebody to go back to the hospital to pick it up.
I:	Oh, right.
R:	After six o'clock.
I:	Right, so you came out without your seven day supply.
R:	Yeah, there's no medication.
I:	And then someone went back to get it.
R:	Yeah, me granddaughter's boyfriend went.
I:	Right.
R:	You know, but he couldn't get there until the following morning and they knew, you know, because I said well, I can't come back because I wasn't well enough and there was nobody else, and (name) said well, I can't go until tomorrow which was the Saturday, so the Friday, I missed two lots of medication because I didn't have it. You know, because it wasn't ready, and I think, that it should be ready for when you come home.
I:	Right, so that's interesting, so there was sort of a hold up then wasn't there, with that? Yeah.
R:	Yeah, you know, and I think you wait three or four hours, and I understand that you have to wait that long, but I don't think they should send you home without your medication because they've sent me home, and I've had to wait until the following day to get me medication, because I had nobody to…

P2
R:	I don't know. I mean to me, you know, as far as I'm concerned when you're coming out of hospital your medication should be ready, you know, because you wait three or four hours, which is fair enough, you know, I understand you've got to wait three or four hours but, it's not always convenient, they say oh we need the bed now, you're going home. So, they send you home with no medication.
I:	No medicines, yeah.
R:	You know, and it's getting it again, I mean I missed two lots of medication, well three actually because the Saturday morning when (name) picked it up he went to the gym and I still didn't get the medication, you know, he said oh nan I've got to go to the gym, and the gym to him was more important than my medication. And I said to him, but (name), I said, I've already missed three doses and you can't just take them all, you have to keep…to you know, and them tablets had to be destroyed, you know, because I can't take them and so I don't get mixed up they're destroyed. Well to me, that is a lot of money.
I:	Well it is, yes, quite.
R:	And it is a lot of money to throw away. I mean, that's all think, that is a lot, and I'm talking about one individual, me, and I'm one person, so how many other people are getting the same treatment as what I'm getting? You know, because I do say, right they've got to go, they've to go and there's two lots and then I can take them because, you see, mine starts on a Monday but I don't know what time he's going to be here, so I start them on a Tuesday. And this is what they cannot understand what I'm saying but I said, you're delivering this on a Monday and I go from Monday to Sunday, but if he doesn't come until Monday afternoon, I've missed two doses, so I start Tuesday, he brings this on a Monday and I take Monday's on the Tuesday because he's not…yesterday he didn't turn up until 3 o'clock, half three, so that's two doses missed and they have to be destroyed, so I start Mondays on a Tuesday.
I:	Yes, I'm with you.
R:	Are you with me? You know, I mean I know what I'm…

P3
I:	That's really, really interesting. What do you think to all that what sort of was that…what do you feel about those mistakes and so on?
R:	It's not the first time. I mean, (name of pharmacy), went into a new system on their computers and I went in several times and asked for the blister pack, oh, it'll be another half an hour, another 20 minutes, and this system, every time my doctor sent the prescription and everything and (name of pharmacy), wanted to take it off, it crashed, it crashed their system, so that took a few days to sort out. I don't know who the fault was with, you know, but I did hear the girl say in the chemist, I'm not getting off this phone till you sort your end out, you know, so… But what's happened this time I don't know – well, as I say, they didn't get the discharge papers…
I:	Yeah, which is what they should be getting.
R:	Yeah, yeah, well (name of pharmacy) have still had nothing, because I promised to take a copy in of mine. So that's a bit of a fall-down in the, you know. Plus it's a bit of a let-down at (name of pharmacy) because even though they've got the new meds from the doctors, they didn't put them into the pack…

P4_C4_C5
I:	Right, so there's been quite a few changes of both different tablets being changed, different dosages of different tablets and so on?
R3:	Every time he goes in, they do…they change something.
R1:	They make a cocktail up.
I:	Yeah.
R3:	Did they take you off your warfarin?
R2:	Yeah, but they put something else that's better in the pack. So he don't have to be tested every week.
I:	Right.
R2:	But's supposed to have bloods took, 'cause the potassium is it [inaudible 00:17:18]?

4a 3 Code - Medication safety - medicine related admissions, errors and delays in dispensing and supply of medicines - cost and wastage.

C2_C3

I: Going back before that time, in the past generally if you like, has there ever been any problems with the medicines?

R1: The only problem we've had is the chemist that previously used to do them, next to the doctor's, for some unknown reason they changed over to the one on the road and we've had a few issues with them not delivering them on time. One time they ran out the injection, you had to go running up to get them. That was a bit with that, but before that everything was fine. With the other chemist it was spot on.

R2: There were that time when she was in hospital, the one time when they changed the tablets and she came home and you had to phone the doctor to get her to put her back on the tablets.

R1: Oh, that's right, yeah.

R2: I think that might have been the second time.

R1: They took her off the tablets, sleeping tablets.

R2: She [voices overlap 05:39] these tablets but didn't let us know.

C2_C3

I: Do you think any further conversations with them might be of benefit or would help you at all?

R1: Yeah, I suppose like you say, the other is a bit more…I think they're more conscientious, but like I say we've not been under these long. I think it's the same sort of chemist.

R2: It's because they've got that many prescriptions.

R1: They've got that many they forward my mum's on to there, you see, so they've kind of passed a lot of them on to this chemist, but we have had a few teething problems with these. They're not as efficient, I don't think, as the other one.

I: In what sort of way?

R1: Well, like phoning up when I've ordered prescriptions that should have come on a certain day and they haven't. Not the blister pack, they tend to be pretty on with that, but like the other things. Like I phoned up about the sleeping tablets, before she went in hospital, I phoned up about the sleeping tablets and they say we'll deliver it, and I phoned again, I said you've not delivered with it. And there was a bit of an issue with the vials one time, and they didn't come. And the nurse came and there was none left. She said I'll come back later, well, I'll shoot up and get them. So there was a bit of an issue there with that. They should have delivered them a day or two before and they didn't come, so there was that problem. But like I say, this is just a new one that they've been sent.

HP12

I: Yeah. Has the service changed the way…I mean really what I'm looking for here is if you can tell me how the service fits in with what you are doing at the moment or what you were doing in your existing work. So, has it changed the way you work or impacted the way you work?

R: It's impacted on the way we work in the fact that we get the information much quicker and everything is done in less of a rush.

I: Yeah.

R: Which I think is quite a safety issue. So, we've got a lot more time to sort everything out. We've got time to sort the prescriptions with the surgery; they've got time to look at it properly, to work out everything. So, that's worked much better. And we're not bothering the surgery saying; please can you print me out a discharge summary.

HP12

I: If there hadn't been changes on the discharge, that would be okay to go?

R: Yes, unless it had been sat for months and months and it had degraded. But yeah, if it was only two or three days, you know or a week or so, and we've had packs made up that were all still the same, then we would just carry on supplying as normal. Although it's very rare that there's no changes whatsoever.

I: Yeah. Where's the benefits there then?

R: Just speed and accuracy of information and patient safety that there is not these things going out that they shouldn't be having.

I: Yeah.

R: And, especially because some patients get delivered a months' worth at a time, so if they have gone into hospital on the Monday, we delivered on the Tuesday and we didn't know, so that would still go, they've then got a months' worth of medication at home.

HP13

I: And you're getting that discharge a couple of hours after the patient's left the hospital.

R: At that point the patient's got a week's worth of tablets, right, therefore you do it quickly and reconcile it when they need a prescription, you've at least got a week.

I: To get it sorted, to get a new prescription done?

R: To get it, you know, and we're getting…that's, you see, very helpful because with that phoning business that we did before, it might not have been as prompt when they actually managed to get through to and then you were waiting for a fax. And it could be two days almost had gone through for all you know and by then the person's beginning to run out of medicines, you see. This is much better for the patient, much better, definitely.

I: Because that patient's not then anxiously wondering where their medicines are coming soon.

R: Well, what patients tend to do, to be fair, anyway, they wait 'til day six or maybe even the morning of day seven and then they phone you up and say, I've got no medicines left, you know, I've been in hospital. And that's no good 'cause this still happens with the non-MDS ones, you see. It still happens because we don't know they'd gone in hospital and it's amazing how many people do that. I think they just think everything's wonderful and it's all going to work so they wait 'til they're nearly running out and then they tell you which is not good.

I: Yes, 'cause that's actually not giving you that block of time to do things.

R: No, not at all, whereas with these MDS it's good and it's a shame they can't extend it, hopefully one day they will.

HP13

I: Yeah, I mean that's one of the questions we've got here is actually looking at that, whether it should be more broad.

R: It really should. I even…I told them at one of the meetings I had nearly a very serious error because of that, kind of, thing on a lady who wasn't an MDS because she had been in hospital, we didn't know, then she got out and we had packets there for her. They were only about a week or ten days' old, things that we were ready to deliver to her but because we didn't have a discharge, we didn't know what she was on and some blood thinning tablets got delivered to her by my staff who didn't realise; some of them didn't know she'd been in hospital, at that point I actually had done. But they'd gone before I realised so they were delivered to the lady's house and she wasn't on them anymore and that's quite a

serious…whereas if I'd have had a discharge for that lady it would've been much, you know, it would've all been organised in a much safer fashion. Nothing happened 'cause the lady was on the ball and she knew she wasn't on them anymore, but I didn't.
I: Yeah, and if she hadn't have been.
R: Yeah, exactly, she could've been taking things that were quite dangerous, but what can you do, if they can't roll it out…but it would be useful.

HP13
R: I just feel I'm giving those patients a much better service because of this than I used to. I just feel that and I'm sure they feel that too, you know.
I: Because of that conversation?
R: Yeah, I really did used to think it was always the doctors' responsibility whereas now I had to be dragged into it and I prefer that. I feel as if it's a good…you're giving them a good service and making sure there's no errors, yeah.
I: And do you feel the patients value that?
R: Oh yeah they do, definitely, 'cause that's another thing, when I call at their house, I'll take the old packs away 'cause that's another issue if the medicines have changed, you don't want them picking up two weeks' ago, you don't know how long they've been in hospital, they might've been in hospital two months, they might have loads of packs there so I'll clear them out when I take the new pack. And I will write on the new pack so I know to tell them that this is the new, up-to-date pack and I get rid of all the others and take them away.
I: Right, yeah, and so the patient, yeah.
R: I didn't used to do that, I don't think I did used to do that, I just used to deliver it and say, that's your new medicines, you see. It's just a safer service, really, it's made it safer.

HP15
I: Right. Okay. Have you then…interestedly, have you had any sort of referrals where it's asked you to do an MUR or NMS or something like that?
R: So, for all of them, when we complete the request, first of all we have to accept when it comes to say that we have accepted the request. And there are a couple of questions at the end, that we have to fill in what we have done with the discharge. So, it doesn't really ask us to do an MUR but in every pharmacy…so once the patient has come out from the hospital, most likely we have to do an MUR on them, to make sure they understand the new medicines. Yeah. Yeah.

HP15
I: The new medicines. Yeah. Yeah.
R: Yeah. So, it's called a discharge MUR. So, we do do that for most patients, but some of them we deliver medication to them, and they are not happy to come in. They keep saying that they are not feeling well, so they won't come in. Yeah. So, we do do our checks on the phone, but it's not a face-to-face conversation, so that we don't do a recorded MUR.

HP15
R: So, they do the prescriptions according to the discharge. Until or when they need the medication from us. So, some of them receive for seven days' supply.
I: Yeah, they get seven days' supply, yeah.
R: But some, they don't get any.
I: Oh right.
R: Yeah sometimes…it's rare, but it's happened and people say, oh we've not been discharged with any medicine and we need one urgently for today. Yeah, but that's unusual. Unless that's when there are no changes to their medicine. So, it's not a big problem but yeah, they can do a prescription then, but it's not straight away. So, we have to wait for a day after still.

HP16
I: We're moving onto values of this actually now but how useful is that, that you have the same information that they have?
R: It's invaluable for us because we've never had that level of information before, we've never been able to make the same impact, I think, and that's the key thing, it's about impact to the patient and its value for that patient at the end of the day and for the system. The system needs to get value for its investment but actually medicines readmissions are such a key, I think six or eight per cent of admissions are due to medicines issues but actually why are the patients getting readmitted if there's an issue? There's a fault in the system there that's allowed them to have an issue and regularly multiple patients it's happening to where actually we could make an impact, it could just be simple AKA counselling. It could be very simple, a couple of words, it could be a couple of sentences, it could be just a point, but even if that can prevent an admission that's extremely powerful, and for the system even one admission saved is...it's massive. And not only for the system but actually the patient, that impacts their lifestyle, it impacts their care, it impacts their view on the health system, et cetera. So actually if we can improve patient outcome whilst delivering excellent clinical care for us that's very job satisfying and that shows us that we are making a huge difference to the patient.

HP16
R: To be honest with you, I think we would welcome it because what we wouldn't want to see is actually somebody's gone in and they've got codeine and paracetamol because they've got a cut on their leg. Something very routine like that, we don't need to see...
I: But they're not likely to have a nominated pharmacy, well, they might, they may do, yes.
R: They may do, you see, that's it, they may do, for example, if you have a nominated pharmacy, you fell, you graze yourself, you want to get it dressed and cleaned and they gave you some paracetamol is that relevant to me?
I: No, it's not.
R: Probably not.
I: So it's about relevance?
R: It is, it's about relevance so actually if there's anything that could impact, so if it's somebody who's on four or more medicines but they've gone in with something, they could be in with, I don't know, a UTI, for example, but actually the link could be, oh, I've seen them three times with thrush or they've come in twice for thrush treatment recently. That's the beauty of it, that's the continuing care impact because they choose to come to us to get their OTC medicines as well as their regular medicines.

HP17

We've got the same data that they've got. We're protecting the GP in a way because we now have the full information rather than bits of it. So, we can get…because we're getting this full picture you're not missing stuff. So, you can look at the full picture of the patient, look…think in your head as a pharmacist, does it look right, is it okay, is there any risk here, are there any clinical issues to discuss, do I need to query this with the doctor? Whereas before we might get no information at all. So, overall there's less risk to the patient, less risk to the doctor of prescribing something wrong, or, say…no, say he does prescribe something wrong, or miss something, which is exactly what happened a few days ago. So, this GP decided to…a patient comes out of hospital, GP prescribes Furosemide Bumetanide. He'd signed off the script. The tray's ready to go. The tray's made up with it all in. Then it gets to the pharmacist after the dispenser's made it up. I said, well wait a minute, this doesn't look right, why is the GP prescribing Furosemide and Bumetanide? Okay. So, before I do anything once they've come out of hospital what do I do? I look at the discharge sheet versus the prescription. Does it match? Does the prescription and the discharge sheet and the backing sheet of the tray match? Are there any differences? Let's read the discharge sheet in detail. On the discharge sheet it clearly says, Furosemide stopped, new diuretic started, new water tablet started, Bumetanide. So, why is the GP prescribing Furosemide as well? Don't know. What's going on? Ring the GP, query it. No apology from the GP. Please put that as not…shall I put that as not dispensed? Yes. GP doesn't say sorry. They never apologise either, but there's a major incident. And that could have put the patient back in hospital.

HP2

I: So, in terms of timeliness then, if the previous service wasn't very timely… they didn't get that information very quickly. Why is having the information quicker, better?

R: For us or for the chemist?

I: For both.

R: Well, for us you know that…well hopefully, that the chemist is getting it there and then. They are receiving it quickly, therefore they are saving money, they are saving time, and they basically know that patient is in the hospital. So, they are waiting for them…for us to let them know that they are back out with any changes or…

I: So, presumably when the patient is in the hospital, they can just…

R: Yeah. They save the time. Yeah.

I: Yeah. They don't have to make up…yeah.

R: No.

I: If they are about to make up, the best fact that they found out that they are in. Yeah. Precisely.

R: Yeah. Because if there are changes then that blister pack is just thrown away wasted.

HP4

R: We see so many medicines-related admissions, you know, because of errors that occur in transition because things weren't accurately documented on discharge summaries, that if we'd stopped something it wasn't documented, so then it might not have been on the prescriptions but it wasn't written in the text, so then it's just continued in community. It's that kind of lack of communication that really causes harm.

I: Yeah. And you see medication-related…

R: Yeah, we see loads, so we have another order set for medicines-related admissions, and I would say we tend to do at least two, three a day. It might not be all due to prescribing errors, some of it might just be side-effects of medication, inevitably, but yeah, we do see I would say on a daily basis we see problems where people have recently been discharged and there's been some kind of error happened in transition between when they've gone home, what they should have been going home with and what they should have continued and then what's actually been continued by the GP. And that could be lack of communication with the patient or lack of documentation on discharge, or just mix-ups generally.

HP7

I: Yeah. And, so then, you've changed it all. Yeah. Going back to your job and your work, I want to look at how it fits in with what you do, so how it fitted into your existing work. Has it, if you can just tell me has it changed the way you work at all?

R: Yeah. I think it has made me think a lot more on discharges and be more precise and make sure everything is more accurate.

I: Yeah.

R: And, a bit more information given to the community pharmacies.

HP8

R: So, it's to improve medicine safety I think. So, we know in primary care, our pharmacists know, they tell me all the time, that when patients are discharged, their list of medications is not always accurate. And we actually spend quite a lot of time doing medicines reconciliations when a patient comes out of hospital. So, it's, as I understand it, an extra step so that the hospital and the community pharmacy have a clear channel to communicate about those medicines. And, oh, I know it's also for patients, it's only for patients who are discharged with the medicines compliance device, like a Venalink or something like that, so I know that it's specific. But, yeah, that's what I think it's for.

HP8

I: So, it's not, like, it's with people with acute…. What are the issues around those patients then and how can this service help that, if you like?

R: I think medicines optimisation is definitely the biggest issue when people are, when you've got people who have polypharmacy. A lot of people, obviously they are not started on all the same medicines by the same people at the same time. So historically they've maybe got some things that have stayed on there for a long period of time that need to be reviewed. New things are being added that might interfere with old things, and people aren't sure, you know, what they're already taking. So that's one of the biggest challenges, I think.

HP9

I: Yeah, and that was one of the other things that people were talking about last year with the service, was that this would…the potential for this to lead to patients better-informed about the medicines. Do you think that's likely?

R: Yeah. I think so. I think that's the potential with it, yeah, 'cause again, when you say, rolling it out to all patients, because there's people not on a Venalink but are on ten medications and something would have changed in hospital but then if nobody tells them about it then they don't know. They might have a box of Ramipril at home and it's been stopped at the hospital and if someone briefly mentioned that to them or not mentioned it at all then they'll go home and start taking that again. And if the chemist is not informed of that then they might have a box on the

shelf waiting for them that repeat prescription or they'll just give them that and then this will just carry on. It's like a – what's it called – the Swiss cheese and when it's like an error, just yeah….
I: Just going through the hole, yeah.
R: …gets passed, yeah…
I: Precisely.
R: …basically. Yeah, and this is a potential…if something stopped they could tell them, this has stopped, they can maybe take it out of the bag and not give them that obviously medication…

P1_C1
I: Right. So, that actually brings me onto the next bit which is like before you went into (name of hospital) recently, were there any problems with medicines and having your medicines, for instance, getting them on time that sort of thing, have you had any problems with medicines before you went into the hospital?
R2: A couple of times where they went missing.
R1: Yes.
R2: At the local pharmacist.
I: Right.
R2: And, they said that the carer had been to collect them and they hadn't and basically, what they'd done is they'd misplaced them in the pharmacist.
I: Right, okay.
R2: Didn't apologise, just went, oh yes we've found them and that was it.
I: And, was there a bit of a delay then in getting them.
R2: Well, fortunately I was on top of it, so I rang MDS the dispensers and said, we need an emergency pack because it was due to start the next day.
I: Yeah. Well, yeah, quite.
R2: And consequently, I said, my concern is the fact that these meds have gone missing.
I: Yeah.
R2: Because we don't know how people would use them or interact with them or interact with them if they were just left somewhere or thrown out, and then of course you've got mum's details.
I: Yes, precisely.
R2: But when they did find them, there was no apology whatsoever.

P1_C1
R2: Now that it's gone electronic, 'cause when it was, being faxed what did used to happen was the pharmacist used to go and revert back to an old prescription […] Which is quite dangerous. […] If things have been changed.
I: Yes, of course.
R2: So, we did do an investigation of that a couple of years ago […] Because it was massive, the fact that they'd reverted to an old…
I: Rather than…
R2: Speaking to the GP […] And seeing what the current prescription was.
I: Yeah, precisely.
R2: Because with bipolar, with mood swings, you have to get the right level. 'Cause it takes a while to kick in.
I: Yeah, to kick in.
R2: About six weeks or so, for it to have any effect. But if they are giving the wrong dosage, it's not going to, you know, mum in what she needs to do. And, have a well and healthy life […] So, we did investigate that. I said I'm not here for blame, if it's a training issue it needs to be worked on, if people need training to make sure that this doesn't happen again.

P1_C1
R2: Which absolutely in the ideal world is what it should be. It should just be a smooth transition and nothing else. What we did find when we came out, when the hospital's blister pack came, there was medication…it was the same medication but over four times in the day […] And, obviously, now that it's come back to MDS it's three times a day […] Which, when you are trying to do like for like into seeing what medications are being taken at what time […] Now, I understand why they did it in the hospital, because that's how they give meds through the day.
I: Right, so that the hospital pack was four times.
R2: No. This is the hospital pack, which is why I kept it as well.
I: Which is slightly different. Yeah.
R2: So, as you can see.
I: Right, four times a day. Whereas that one, there is nothing on the teatime one.
R2: Yes. Nothing on the teatime one. So, trying like for like.
I: So, is it the same.
R2: Which I did speak to the district nurse that came round to say, right, are these the same because again, the appearance is different.

P1_C1
I: Do you think, can I ask you to think what other problems you had with the medicines, and you talked about that one medicine that had gone wrong? Have you had problems in the past with these blister packs being made up incorrectly or being made up or not arriving on time?
R1: Not really.
R2: Only the one time that I mentioned where we had the review and we had to do a proper investigation as to what had happened there.
I: Yeah. But any other problems with them at all.
R2: Not that we can think of. Sometimes certain meds are missed because it doesn't seem to be part of, like your calcium tablets, they were hit and miss.
I: Right.
R2: So, mum would get a blister pack, she'd get the olanzapine but then she has calcium tablets which are an add on.
I: Yeah.
R2: But you've been taking them for over 12 months now so.

R1: But they've stopped them.
R2: Yeah, but they've stopped them now. But what was happened was, she'd get them and then she wouldn't get them, then she'd get them and then she wouldn't get them.
I: Yeah.
R2: And, so she'd go a couple of weeks without them.
I: Yeah.
R2: I mean they are not life threatening but it's the fact that it still happens.
I: Yeah.
R2: So, whether it's…I mean, whether it's a significant tablet or not, it's the fact that the consistency wasn't there.

P2
I: You get those from the pharmacist, don't you? So, how do they arrive, are they delivered?
R: They're delivered […] You see now they used to send four weekly, but because the hospital are changing the strength, they just send one week at a time, which I can understand because when they send, like last time, they sent four weeks and three weeks was no good.
I: So that's the week you have now from the chemist.
R: Yeah, on Monday, yeah. So of course when they send that, there are three weeks that's no good, so all that medication is being destroyed and I sort of think, well surely by now they must know what I need, and now they've turned round, because I've been in a few times, you know, last time wasn't the first time, I've been in a few times, I've gone to the clinic and they've not let me come home because me heart rate is so fast, and they send me upstairs, you know, to the ward and I wait, and then it's back. But now they're saying it's not the heart, but it must be the heart, because my heart rate just goes berserk and then it stops, so now they're giving me all this, I'm taking it, I don't know what it's for.

P2
I: So, when you left the hospital, did a pharmacist […] come around and talk to you about your medicines at all?
R: No, nobody came up and said, oh there's, you know, your medication. I mean, like when I left the first time the week before, you had to wait so long for the medication and they know in the morning, I understand they've got thousands to do, but you know, I'm waiting, I come home and then have to rely on somebody to go back to the hospital to pick it up.
I: Oh, right.
R: After six o'clock.
I: Right, so you came out without your seven day supply.
R: Yeah, there's no medication.
I: And then someone went back to get it.
R: Yeah, me granddaughter's boyfriend went.
I: Right.
R: You know, but he couldn't get there until the following morning and they knew, you know, because I said well, I can't come back because I wasn't well enough and there was nobody else, and (name) said well, I can't go until tomorrow which was the Saturday, so the Friday, I missed two lots of medication because I didn't have it. You know, because it wasn't ready, and I think, that it should be ready for when you come home.
I: Right, so that's interesting, so there was sort of a hold up then wasn't there, with that? Yeah.
R: Yeah, you know, and I think you wait three or four hours, and I understand that you have to wait that long, but I don't think they should send you home without your medication because they've sent me home, and I've had to wait until the following day to get me medication, because I had nobody to…

P2
R: I don't know. I mean to me, you know, as far as I'm concerned when you're coming out of hospital your medication should be ready, you know, because you wait three or four hours, which is fair enough, you know, I understand you've got to wait three or four hours but, it's not always convenient, they say oh we need the bed now, you're going home. So, they send you home with no medication.
I: No medicines, yeah.
R: You know, and it's getting it again, I mean I missed two lots of medication, well three actually because the Saturday morning when (name) picked it up he went to the gym and I still didn't get the medication, you know, he said oh nan I've got to go to the gym, and the gym to him was more important than my medication. And I said to him, but (name), I said, I've already missed three doses and you can't just take them all, you have to keep…to you know, and them tablets had to be destroyed, you know, because I can't take them and so I don't get mixed up they're destroyed. Well to me, that is a lot of money.
I: Well it is, yes, quite.
R: And it is a lot of money to throw away. I mean, that's all think, that is a lot, and I'm talking about one individual, me, and I'm one person, so how many other people are getting the same treatment as what I'm getting? You know, because I do say, right they've got to go, they've to go and there's two lots and then I can take them because, you see, mine starts on a Monday but I don't know what time he's going to be here, so I start them on a Tuesday. And this is what they cannot understand what I'm saying but I said, you're delivering this on a Monday and I go from Monday to Sunday, but if he doesn't come until Monday afternoon, I've missed two doses, so I start Tuesday, he brings this on a Monday and I take Monday's on the Tuesday because he's not…yesterday he didn't turn up until 3 o'clock, half three, so that's two doses missed and they have to be destroyed, so I start Mondays on a Tuesday.
I: Yes, I'm with you.
R: Are you with me? You know, I mean I know what I'm…

P3
R: Yeah. Now, with those, I had to take…that pink one, I have to take that out.
I: Oh right.
R: Right, and I have to put these two in, and that statin, I have to break it in half.
I: So currently this has been made up by…
R: My local chemist.
I: Your chemist.
R: Yeah.
I: And this is…so currently some of these bits and pieces are wrong.

R: It's pretty much as it was before I went in hospital.
I: Oh, so they haven't made the changes yet?
R: When I left hospital, the day after I went in to the pharmacy and asked them if they got all the information and they said no, they said, we've had a phone call from the hospital pharmacy and they have told us that there's some changes, so I said, so you've not had the discharge papers, so they said no, I said, have you had the new list of medication, no, have you heard from the surgery, no. So I said, right, I'll ring them, she said, well I'll ring them and have a word. So when I rang the surgery said, we've already sorted it out with (name of pharmacy), it's all done and everything, so I thought, right, that's fine, showed (name of pharmacy) the blister pack that I'd got from the hospital and they said that we haven't had time to get you one ready, your next one, she said, so the one that we've got ready has got all your old meds on and there's no changes, so we'll have to give you that and the boxes of the additional tablets and you'll have to take the Ranexa out and break the statin in half because they'd reduced…
I: They'd reduced the dose, yeah.
R: …dose. So that's the point I'm at at the moment, and my pharmacy said…I said, well I always used to pick it up on a Thursday because my first dose was Friday morning on the new one, so now I have to pick it up on Monday because they've rehashed everything, so I have to pick my next one up next Monday and that should be up-to-date…

P3
I: That's really, really interesting. What do you think to all that what sort of was that…what do you feel about those mistakes and so on?
R: It's not the first time. I mean, (name of pharmacy), went into a new system on their computers and I went in several times and asked for the blister pack, oh, it'll be another half an hour, another 20 minutes, and this system, every time my doctor sent the prescription and everything and (name of pharmacy), wanted to take it off, it crashed, it crashed their system, so that took a few days to sort out. I don't know who the fault was with, you know, but I did hear the girl say in the chemist, I'm not getting off this phone till you sort your end out, you know, so… But what's happened this time I don't know – well, as I say, they didn't get the discharge papers…
I: Yeah, which is what they should be getting.
R: Yeah, yeah, well (name of pharmacy) have still had nothing, because I promised to take a copy in of mine. So that's a bit of a fall-down in the, you know. Plus it's a bit of a let-down at (name of pharmacy) because even though they've got the new meds from the doctors, they didn't put them into the pack…

P3
I: You've just got boxes and packs.
R: Yeah, so what I've got to do now as I say is I've got to make the adjustments in the pack…
I: Which sort of defeats the object of the pack?
R: Exactly, yeah, yeah, yeah. So as I say, I have these two additional ones that I have to put in. The other problem is with that, one of these is a water tablet that…so that is a stronger one than the water tablet I've got, but I don't know which one it is, because I'd have to take that one out and replace it with this one. So all I can assume is – they've only given me seven days of these, so all I can assume is just take the one that's in, you know, because I could take the wrong one out.

P3
R: But as I say, it's just with the case…I mean, when we went in last week for the blister pack she said, oh, so-and-so has just redone it, it's not been checked yet, said, okay, we'll go and have a coffee, you know, no problem. When we went back, they still hadn't checked it, so we hung around and they checked it and came back and that's when she explained then that she'd put these…
I: Extra boxes.
R: But she didn't tell me I had to take the Ranexa out, which they've stopped, you know, so…
I: How did you know you'd got to take that out, who had told you that?
R: They told me at the hospital the Ranexa was stopped, yeah.
I: Right, and they hadn't taken it out the blister pack.
R: No, because they say they've not had the information.
I: Right, so they're working on the old prescription still.
R: Yeah. But when I rang the surgery, they said they'd sorted it all out and they'll send all the new stuff to (name of pharmacy).
I: So when you collected…do you collect it weekly?
R: Yeah.
I: Yeah, so when you collect it this Thursday…
R: Well, it'll be Monday next week.

P4_C4_C5
I: Yeah. Thinking about when you were, before you went into hospital, and sort of up until that point, as it were, has there been any problems with medicines, have you had any sort of like problems, like getting the medicines on time, getting the medicines from the pharmacist?
R2: Yeah, more so when he's been in hospital and they change it.
I: Right, yes.
R2: They change the medication.
I: So, because you've been in and out of hospital then, so is it around those times when you've come out of hospital that there's been problems?
R2: Yeah.
R1: Yes.
I: That's when you've had most of the problems?
R2: Yeah.
I: Right.
R2: 'Cause the doctor says he hasn't got them (the medicines).

P4_C4_C5
I: So, did they get sorted, those problems with those medicines?

R2: Well, I phoned and she said, what was it, I'm sorry there's nothing come through. I said, well I'll tell you what, I said, it better be through for Thursday, I said, or my husband's got no tablets. […] So she said, well just a minute. Oh yeah, they've found it […] They've never passed it through. And I know he takes his time for signing them off.

R3: There has been one occasion, where I have been chasing a prescription from morning, that should have gone through to him, and I've waited and waited, to the point where I've gone down to the…I've phoned all morning, oh, he's in clinic now, he'll do it when he's finished clinic. […] And I've gone into the surgery, and asked for him. Oh, he's on his lunch. And I've waited in the surgery, and he's walked through and I've gone, is that the doctor? Doctor, I forget his name now, but at the time I went, Dr (name) And he turned round, and he's going, and he's walking, and I went, Dr (name). And he's turned round, and I said, it's my…(name of patient) daughter, I said, have you got his prescription please, I said, only he's desperate for it? And he's looked at the girls and he's gone, oh has it come through yet? I said, yes, it come through at such a time this morning, and I said, he's urgent for it, the girls passed it through to you at such a time today. And he's just walked off, gone in his room and then sent it, I'm sat there, and he's sent it through to the pharmacy, and then the girls have said, oh, he's sent it through to the pharmacy…

R2: Won't come out.

R3: ..cause I was like that, I thought five more minutes, and I'm going to knock on his door, because I think they just…you was brought up with respect, my Mam and Dad always brought me up with respect, and now I have a few friends who are GPs and pharmacists and stuff like that, and they're ordinary people, but some of them get this chip on their shoulder, and you think, no, you treat people with respect. And to me, that GP, he's just taken on too much and can't cope, but he won't pay for the added locums and the extra staff to take on the workload he's got, and he's there for a service not to make money. If he's in that, he's in the wrong job. [..] He really is. The run ins we've had with him, I'm sorry, that is why…

P4_C4_C5

I: Right, so there's been quite a few changes of both different tablets being changed, different dosages of different tablets and so on?

R3: Every time he goes in, they do…they change something.

R1: They make a cocktail up.

I: Yeah.

R3: Did they take you off your warfarin?

R2: Yeah, but they put something else that's better in the pack. So he don't have to be tested every week.

I: Right.

R2: But's supposed to have bloods took, 'cause the potassium is it [inaudible 00:17:18]?

P4_C4_C5

I: And on each of those occasions, when you come out, and medicines have then been changed, so it has been, you say there's been lots, yeah?

R1: They might just change one tablet, they might change three or four.

R2: Or they'll take one out and put something else in.

I: Put something else in, yeah. So, there's been thing that have, so in terms of over the last six months, there's been a lot of chopping and changing [inaudible 00:34:50]?

R2: Yeah.

R1: Yeah. They've stood at the side of the bed, and you've had the, it could have been the registrar, but it was the chief of the team, he'd be there, and then there's, the registrar would be there for instance, and they'd have a chat between them, and they'd say, yeah, well we'll get rid of that. Okay then, (name of patient), you know, see you later. And go away.

I: Yeah.

R1: Now, I know they've changed two, maybe three tablets.

P4_C4_C5

I: But it seems it's not just about knowing about that, it's also, you were saying earlier, it's about having them on time?

R3: Aha.

R2: Yeah, that is basically the biggest worry.

I: Why is it such a big worry?

R2: Well, I'm phoning…

R3: They've changed it for a reason, he has blister packs, he's either got his old blister pack, but they've put him a week on the wrong medication, if you can imagine, when you're changing your medication, you go light-headed, you go strange.

P4_C4_C5

R3: He's bad on his feet, he's also not in the best of health.

R2: He can't see proper.

R3: And it's like, what you're doing to him, what are you doing to his body? If you change it, change it, but keep up with the… 'cause there was one time, where he had to lose weight, and he was told to be seen in four weeks' time.

R1: Oh yeah, that's right.

R3: That was ridiculous. So he's trying to lose weight, he couldn't even get an appointment for four weeks' time, then they changed his doctor and he was under two consultants, and they're contradicting each other, and you just don't know…

R2: What's the best.

R1: One was about the weight wasn't he? […] Get rid of your weight, and the other one said…

R2: That's when they upped your water tablets.

R3: Who's right and who's wrong? Do you take that tablet, or do you take that tablet?

R2: Antibiotics - have a lot of fluid and drink a lot of water. Don't drink a lot of water with his legs.

4a 4. 5Timesaving, speed, efficiency - streamlining of communication

HP1

I: No, no, I think that does, because it's sort of like, you know, basically the more information you're getting therefore, and the faster you're getting it, and if it's directly saying, you know, look at this?

R: Yeah, and also, exactly the faster you get the information, because what my experience was, we kind of just went around in circles, when we didn't really need to. If we were involved from the beginning, we would have just got that information, we didn't have to waste our time finding out where the information's come from, what we need…so it would have just been better, if we just had the information as well, and we just tackled it there and then.

HP1
I: Do you think, sort of moving on a little bit, do you think this is something which has potential value or benefit going forward, the service as a whole?
R: Yeah, I think so definitely. As a three way communication, and highlighting important changes that really will affect the patient, that will harm the patient if it hadn't been changed, or communicated, I think definitely, and I'm positive to change, I'm not usually negative about something, I think this is great, and it's definitely something that's needed, I think in time it will be more slick and I think, once we've used it, and everyone knows how to deal with it, then definitely it's very positive, yeah, definitely a move forward.

HP1
I: What do you think the impact upon, well a couple of things, the impact upon working relationships across those transitions, between yourselves and community pharmacists, yourselves and hospital pharmacists. What sort of changes do you think the service is going to make to those sorts of working relationships?
R: I think it will help with knowing who's who. 'Cause there's so many of us now, and all over the place, we're like ants. So, I think it will help us know who is in what team, and what their role is. And it will also, it's more streamlining, sending information across, so again, assuming it's three way communication, we could then just send a quick notification via pharm outcomes, to the community pharmacy, and they will then send one back, instead of being on the phone for endless hours waiting for someone to pick up, or…I think definitely, and also it's a formal way of communicating. Sometimes, there have been occasions where it's been, oh you didn't tell me that, oh I did tell you, why didn't you…you know? So, having it written down, formally, is also safer and a better way of communicating I think. And also, the hospital, definitely, it's useful knowing what ward they're in, and kind of…
I: Why does that help?
R: Because, sometimes, if there was a query, so on the discharge, they might not have put something, and we just wanted to know, it's easier to go straight to the ward, and say, do you remember this patient that you've just discharged? Yeah, so that was useful. Yeah.

HP11
I: That's great. That's brilliant. So in terms of work, has it made any differences to how you work? Has it changed things, hindered things, made it more complicated? Made it easier or made it better, or what?
R: I think it's changed massively. I think it's probably helped because we have all the information. But the actual process isn't much different because we were still getting the discharges from the hospital.
I: Yes, but just by fax.
R: Just by fax.
I: Yeah. So the processes…
R: We're still getting the same information now, just more reliably and immediately I think as well because I know (name), was saying as soon as that's generated at the hospital, that gets sent to us.
I: Yeah, I think it's two hours.
R: Yeah. Whereas before, you know, it might have been a day or so, mightn't it?
I: Yeah.
R: Whereas…which is obviously helpful for us because we've only got seven days and obviously with weekends and everything, the practice is only open five days, we've only got … if they send them out seven days…
I: Seven days' supply, yeah.
R: …we've only got five days to sort it and get it out to the…
I: Precisely.
R: …to the patient. So I think it's made it more efficient because we've probably got more time then.

HP11
R: Yeah, I don't think I…yeah, I think it'd probably just progress like…but I think (name)'s planning to do that anyway. I think that's…yeah. Yeah, I think for what we can do now with the services we have, yeah, I think we're getting all the information we need.
I: Yeah. So do you think it's…I mean, broadly, do you think it's a positive thing?
R: Oh, definitely. Yeah, in every way, yeah. I can't see that it could possibly be a negative thing, can it?
I: What are the main things that make it a positive thing then, I wonder?
R: I think there's the security, the extra information, the speed. Just communication really, it's just like, knowing more is always a positive thing, I think.

HP12
I: Yeah. Has the service changed the way…I mean really what I'm looking for here is if you can tell me how the service fits in with what you are doing at the moment or what you were doing in your existing work. So, has it changed the way you work or impacted the way you work?
R: It's impacted on the way we work in the fact that we get the information much quicker and everything is done in less of a rush.
I: Yeah.
R: Which I think is quite a safety issue. So, we've got a lot more time to sort everything out. We've got time to sort the prescriptions with the surgery; they've got time to look at it properly, to work out everything. So, that's worked much better. And we're not bothering the surgery saying; please can you print me out a discharge summary.
HP12
R: No probably not, but I don't think there's any disadvantages to our work. If that makes sense.
I: Yes.
R: We can plan it better we are not as rushed. But I don't think there is any other particular advantages other than we are not having to send maybe drivers out to pick up things that shouldn't have gone because we didn't know they were in hospital, or we've sent packs and it's not

got everything in, so they've gone and picked them up, and do new ones, that kind of thing. But no, it works; from my point of view, it works really well.

HP12

I: Another question is about working relationships. One of the things I wanted to expand that to is, has this changed the relationships you have with the – I mean by large I presume most of your patients are from next door.

R: The vast majority are from next door, yeah.

I: So, has it changed the relationship with the general practice?

R: Yeah. I mean the relationship has changed a lot anyway because of this change of practice pharmacist. We have always had a very very good relationship with the practice next door, which deteriorated when we had the previous practice pharmacist. There became quite a lot of issues.

I: Right.

R: And now we've got the new one the relationship is great again now. And I think from their point of view it's probably benefitted the relationship because we're not hassling them, we need these scripts by today, we need them now, you know, the patient's no medication.

I: Because you've got more time?

R: Because we've got time to sort it out.

I: Because if you are seeing the discharge summary within a couple of hours, you have got seven days before that patient has run out.

HP13

I: And you're getting that discharge a couple of hours after the patient's left the hospital.

R: At that point the patient's got a week's worth of tablets, right, therefore you do it quickly and reconcile it when they need a prescription, you've at least got a week.

I: To get it sorted, to get a new prescription done?

R: To get it, you know, and we're getting…that's, you see, very helpful because with that phoning business that we did before, it might not have been as prompt when they actually managed to get through to and then you were waiting for a fax. And it could be two days almost had gone through for all you know and by then the person's beginning to run out of medicines, you see. This is much better for the patient, much better, definitely.

I: Because that patient's not then anxiously wondering where their medicines are coming soon.

R: Well, what patients tend to do, to be fair, anyway, they wait 'til day six or maybe even the morning of day seven and then they phone you up and say, I've got no medicines left, you know, I've been in hospital. And that's no good 'cause this still happens with the non-MDS ones, you see. It still happens because we don't know they'd gone in hospital and it's amazing how many people do that. I think they just think everything's wonderful and it's all going to work so they wait 'til they're nearly running out and then they tell you which is not good.

I: Yes, 'cause that's actually not giving you that block of time to do things.

R: No, not at all, whereas with these MDS it's good and it's a shame they can't extend it, hopefully one day they will.

HP15

I: Good. We are recording. We are talking about…thinking about the introduction of the pharmacy e-referral service. What do you think it is there for? What do you think the purpose of having that e-referral is?

R: So, it gives a clearer discharge letter to us first of all. So, the handwritten ones, sometimes they are not very clear and it causes confusion. But when it's electronic, it's easier for us to go through and compare to the old medication and see if there are any changes. So, it's quicker for those purposes. Also, because it is electronic, we get to know quicker than we would usually if the patient has been admitted to the hospital or is discharged. Because sometimes we wouldn't even know if they had been admitted, until they come out and they ask for their medicine. So, we wouldn't know, yeah. But it's better when it's electronic now. So, we get to know that they are in the hospital now.

HP15

I: Yeah. So, you were getting informal conversations from people to let people know, but not before. And you said that the discharge was handwritten beforehand and it wasn't electronic. What information did you get from that before?

R: It's just the medication. So, what they were discharged with. What medication they were discharged with. So, you usually used to get faxes. So, they call us first to say that, what's your fax number? And they'll fax over the discharge letter. But sometimes when they do that, we still don't get the discharge letter until days after. And then the patient…at that point because it's not straight, it's not the same day they are discharged. It is probably two days after they've been discharged. So, there's a delay.

I: Yeah, they are already in the community.

R: Yeah. And then what happens the patient will need the medication. They usually get discharged with seven day's supply. So, if you have not received it by the fourth day…I mean when the doctors haven't received the discharge at that point, there's a big delay because they don't know, they can't do the prescriptions until they've seen the new discharge letter and everything.

I: And then you've only got three days left before.

R: Yeah. So, what happens we keep…we are continuously chasing up…can we get the prescription for…when they haven't got the discharge either. So, we usually get the discharge on the fifth day. So, it just leaves two days for them to look at everything, arrange a prescription. And the patient is getting very worried and saying, well I don't have medicine, I don't have medicine. Even though they have two days left but they're scared. So, they keep chasing us, that can I have the medicines?

HP16

R: I think there's multiple impact really, for the patient it's seamless care which is the aim of the project, it's about seamless transfer of care between the two sectors. For a patient that's what they need, they want to see the medicines on time, they want to see that actually they've not had to chase things up, they're not worried, they're not anxious about getting their medicines. Those sorts of things, actually will they come on time, did you know I was in hospital, did you know I've come out, did you know this has changed? All these things are avoided.

HP17

R: So, the sooner we get that information then you're reducing the delays to the patient getting their medication in community. The limitation of this system is the surgery. So, even though we get the discharge sheets I still can't do anything until the surgery accept the information at their end, process the discharge sheets, pass it to the GP, get the GP to then do new prescriptions, whether they be monthly,

whether they be weekly depending on the risk, and then those prescriptions have got to come to us. Some of the surgeries don't do anything. They'll just sit on that information, it'll just sit on their computer, and they'll do nothing, and then the patient…
I: Yeah. So, you're getting that information, which you didn't get before, but you can't act upon it until you've got the script, can you?
R: We can provisionally act upon it. We can make sure we've got the drugs in stock, we can put the patient on a sort of chasing up shelf and have the…make sure…see what's changed, look to see if we've got any existing scripts. So, if they've put on the discharge sheet, no changes, okay, no problem, we'll just continue the trays as they were. We might use existing scripts, we might request new scripts if we need them, but if the surgery…or we can…it prompts us then to phone the surgery and say, wait a minute, we've had notification this patient's come out of hospital, we need new prescriptions for everything, why have you not done it? Oh, it's sat in the doctor's room, or, oh, sorry about that, we've had it a week but we've not done anything.

HP17
R: Yeah. Then you get to the point where you say, well too much information. So…
I: And too much…yeah.
R: …because we manage tray patients in so much detail down to what time of day they take their medication and you need…it needs to be perfect, there's no room for error, you need the tray ones. Whether you need more than that at this moment in time I'd probably say, no, we've got enough to be going on with. But further down the line when you sort of integrate GP records with pharmacy, or some way that pharmacy can dial in and look, will be helpful, but then you've got the time issue that we're so pressured in pharmacy every second is important.

HP2
I: And do you think…what do you think that then is trying to achieve…that doing that?
R: Well, it's a quicker service but it's also letting the primary care know if there are any changes and then when the patient goes home or goes back to the nursing home it's done promptly.

HP2
I: Yeah. Oh, that's actually brought us perfectly seamlessly on to our next question, which is about how it was introduced into your workplace. So, what, sort of, things went well, when that went live, 17 February wasn't it? I think.
R: I think it was, yes. Well, it was just so easy to follow. Just the whole process was easy to follow. You know, you clicked on one thing and the next screen came up. So, it felt too easy. Yeah, it felt too easy to be right, but because we did that, we knew the people in the dispensary every evening who were faxing, faxing, faxing, had all that spare time to do something else. So, it took time off people down in the dispensary…a busy time of night, when they are faxing all of these prescriptions. So, they could carry on with something else, help out other staff, so…
I: Yeah. So, it freed up people.
R: It freed up people. Yeah.
I: Who had been, sort of, like, clogged down with this.
R: Yeah. Who were standing at the fax machine, just faxing for however long.

HP2
I: What would the benefits then of having that three-way communication be? In terms of…
R: Just the time. Everybody is on the same page at the same time. So, that's why.
I: So, it's clear what that patient…
R: It's clear what that patient's on and what they should be doing. Yeah.
I: Yeah. Yeah. Yeah. Precisely.
R: And if the patient cannot…if the patient is confused or not taking their medications as they should, then maybe the GP should get involved and more and find out why.

HP3
I: That's good. It just contextualises things nicely for me. So I want to start off really by talking about the e-referral service and when it was…the fact it has been introduced. Why do you think it was brought in?
R: I think probably to make things a bit more efficient and more convenient for us when we are doing medicines reconciliations. I think it takes a bit of time to ring the pharmacy, get through to them and say, this patient's in, put everything you've got on hold. Especially when we've already got the compliance aid there and it's literally just to ring them, to say, listen, this person's in right now, can you just put everything on hold? And then also, on discharge, like faxing all the information over, which, personally, I think it's old-fashioned to fax over, so it's just to make things more efficient, pretty much.
I: So do you think that the purpose of the service is that timeliness and efficiency then?
R: Yeah.
I: Are there any other reasons why you think it's there?
R: It's not even just efficiency for us, I think it's efficiency for the other pharmacy, for the patient, in terms of discharge. I think it's to make things clearer, things just being electronically sent over, instead of us actually writing down, oh, this is what's been changed, this is this. And also, if there are any changes, I think it updates when it sends to the pharmacy, so they can know straightaway if anything's actually changed.

HP3
I: So using e-referral, in what ways has it changed how you and the team around you work?
R: I'd say it's just a bit more efficient, in the sense that, again, I don't have to ring the pharmacy, I'll just send an e-referral through and that's done. And also for, I guess, transfer of care between us. So if a patient's been transferred from the emergency admissions unit where they've done the drug history there, I can see when... By the time they get up to the elderly care ward, oh, that pharmacist has already put in a referral, so I don't need to make any contact with the pharmacy because someone's already sent through that referral.
I: Yeah. So you said it's more efficient, and does it save time?
R: Yeah, I'd say slightly, yeah.

HP3
I: So have there been any hindrances or constraints to your work? Have there been any challenges and difficulties with the service?

R: I'd say probably when we first started using it, but then that was because we were just still learning how to use it, so it felt like, oh, this is just taking so much longer and why can't we just fax it? But now that we understand how it works and how to use it, it's fine. I can't really think of a bad thing about it, to be honest.

HP3

R: I think it should be used more widely, because, again, that's another thing that could cut down time. Me ringing the pharmacy to let them know that, oh, the substance abuse user patient that you guys have regularly is currently in, or if I could just send through a referral, that would be [voices overlap 0:28:36] it would just save so much time.
I: And as you say, they know not...
R: They can put that on hold.

HP3

I: Yeah, people knowing that you've got a community pharmacist knows what this patient's on, or a practice-based pharmacist or a GP, a hospital pharmacist, all these people know what medicines the patient is taking, and where does that have a benefit for the patient?
R: In terms of, I guess, like continuity, so if something has been stopped here and the community pharmacy doesn't know, because I've seen this happen before, they will just continue to put the same things back in the blister pack. The patient will continue to take it, not aware that it's back in there, and they'll end up back here because they were taking something that they shouldn't have been taking. Whereas if the community pharmacy is aware, the GP practice pharmacist is aware, I guess it's kind of like the Swiss cheese model, things will be picked up, so there are no errors that will fall through, it will all be picked up by people that are aware of the situation.
I: Yes, so you've got more...
R: Barriers to it, yeah.

HP4

R: To kind of I guess standardise communication, to make it more reliable, because before it relied on people kind of, you know, faxing, ringing, too many chains in the…too many links in the chain where it fell down routinely because we'd constantly get phone calls with problems where it didn't happen. And so safety I guess overall, I think the aim is probably to do with patient safety, improving communication from secondary care to primary care in terms of medication changes in hospital.
I: So that communication, how can that help with that safety or how is that going to help…?
R: Because inevitably I see it all the time when patients are discharged from hospital, we see so many problems with mistakes, errors, prescribing errors, when patients go from one setting to another, when they go from primary to secondary, secondary to primary, you're constantly facing that battle of mistakes in that transition, and the aim really is to make sure it's communicated reliably. So if the chemist are getting an electronic discharge of like a snapshot of exactly what they were discharged with, that takes out that kind of element of, you know, they've got the information there so they can then hopefully act on that quickly, much more quickly than relying on a fax two days, three days later where there used to be quite a significant delay, things can get changed quicker, so if it's blister packs they can get their new blister pack up and running, they can nag the GP to get the new prescriptions through. So hopefully the next time the blister pack comes out, it will have the correct medication in, not medication from like preadmission.
I: So it's about having that correct…making sure the patient is on the correct medication …
R: Yeah, exactly, yeah, making sure changes have been communicated accurately.

HP4

R: Yeah, so this week we've just recently had a new meds reconciliation document version, I don't know if you've heard of that, but that now links to the e-referral, which is really good, because…
I: Right, so this is an electronic document, yes.
R: It's an electronic document which pharmacists and technicians use to basically – it's like a stepwise document you kind of go through, and it's all about accurately documenting the drug history for a patient, so when they come in it says, which sources have you used, and it will be GP prescription, patient, patients on medicines for example, so you'd tick it through, you'd work your way through, it's, does the patient have any medicines with them. And then you document the drug history, so you write whatever they're on at home, and then it says, is this – I can't remember the exact kind of terminology, but it says something like, can you complete an e-referral, do they have a compliance aid basically, and if you tick yes, it automatically brings up the box for the e-referral, so you can click on that and it exports you to…
I: And then just takes you…
R: …yeah, takes you straight through to the e-referral, which is good because it means you're less likely to forget to do it.
I: Yes, so you haven't got to think, oh, that patient's got a Venalink…
R: No, so…
I: …I need to do…
R: …you did last week, but since this new kind of document has been introduced…

HP4

I: So until this week, that was happening, was that previously what you were doing to…?
R: So previously you'd kind of rely on the fact that you had to remember to do it, and I did nearly forget a couple of times, because it's easy to do when you've got a complicated drug history, kind of in and out of different documents, you know, looking around and documenting things, and you'd put like…you still have to record it in the significant event button that we use, we still put blister pack there or Venalink and the details of the chemist. So then you'd have to remember to go into the orders and type in community pharmacy referral, it's like a separate step that you'd do at the end…
I: Whereas now, it's an automatic dropdown, soon as you've said yes…
R: Yeah.
I: …they're on a Venalink…
R: Yeah, it takes you through to it, yeah, which is better because it reminds…it's almost like it's more reliable because it's a prompt really.
I: Yes, so there's less…
R: Yeah, less likely that you're going to forget to do it at the end, yeah.

HP4

I: No, no, I mean, and I haven't either yet, so yeah. So what happens then, so that's on admission…

R: Yeah, so that order stays on their current inpatient chart throughout their admission, then my understanding is on discharge basically that order will automatically ping off…

I: Right. So when you then…do you have to physically do anything?

R: You don't have to do anything – yeah, my understanding is once you've put that referral on, that electronic order, when they are discharged, that will automatically notify the community pharmacy that they've gone home and they'll get a copy of the discharge summary.

I: So you only ever go into the system once, really.

R: To the order, yes, to the referral. So on discharge it's a slightly different process, there are certain things we have to do on discharge, like in the [QRG or 15:56] the guidelines they kind of set up with this, we have to annotate the discharge summary with documentation to say who's clinically checked the discharge summary, the time, the fact that we've asked the patient's consent if they're able to, and details of what we've supplied. And as part of that we check that the discharge summary was accurate in terms of the stop-started amended medication. So we do annotate the discharge summary on discharge to make it clear to the community pharmacy what we've actually done. Because before I guess you would have ticked on the coversheet that we used to use, the paper sheet, we would have ticked on there, seven day supply, Venalink dispensed, 14 day supply, you know, it would have been in black and white on paper, so this is kind of another way of…

I: With the cover letter with the discharge summary handwritten just…

R: Yeah, basically, yeah, yeah.

I: Faxed.

R: Yeah.

I: So it's quite a change…

HP4

I: Is it therefore, you know, it's adapted the way you work then, has it then improved things…

R: Yeah.

I: …save time or…?

R: It does save time, definitely, I would say it probably doesn't save – well, it saves a little bit of time on admission because otherwise you would have had to ring the community pharmacy, so it's quite nice to not have to ring them in certain situations. You know, if you've got the blister pack in front of you and you've got all the information you need, you can just stick the e-referral on. Sometimes you'd be ringing all morning trying to get through to the chemist and it would waste a lot of time actually having to keep going back and ringing the same number and nobody is answering. So that definitely saves time. And then on discharge as well obviously with the whole, you know, it's just much easier on discharge.

I: One of the things when health professionals say to me that that saves time or this only takes so much time and now we've saved some time, one of the things I often say is then, so what do you do with that time?

R: Yeah. Well, we see more patients, which is good, or you have more time to do clinical things rather than just kind of, you know, desk…

I: So it takes away some of this administrative work…

R: Yeah, exactly.

I: …and brings you back into those patients.

R: Yeah, that's right, which helps, you know, so we can then have more time to correct mistakes and prescribe on the ward and things like that. So yeah, I would say definitely saves time. I still the odd time, the only downfall with having the e-referral sometimes is you still end up having to ring the chemist on admission to clarify certain things. So if for example like a patient doesn't have their medicines with them from home, you might be able to see from their GP records who their nominated pharmacy is, but you might still have to ring that pharmacy just to check, do you do blister pack for them or do they not, it looks like you do, but I always kind of ring just to check, because I think it's easier just to sort it out then than for someone to get a decline in like two days' time or whatever. So I kind of ring them or sometimes might want to clarify the timings of the drugs, so if it's not clear, if they're on like 20 things and they're all white and you can't work out which one is at night-time, which one is teatime, you might just need to go through the blister pack with them over the phone and say, is that night-time, or if there's a mistake sometimes as well in the dispensing, in the actual blister pack you might say, is that a mistake, the label is not there but looks like it's in or… So there's still times where we have to ring them, but I would say overall it definitely saves time.

HP4

I: Yeah. So those challenges, that's having the blister pack, but the e-referral, how is that going to get over some of those challenges?

R: I think like we said, it just makes it more reliable, that that communication is going to be actioned and quicker, so when a patient goes home, I can kind of assume that that e-referral is going to get picked up, the community pharmacy can see straight away that aspirin stopped or whatever. They can then prompt the GP to make the changes more quickly, it just speeds everything up, it makes it more reliable that they're going to then get the correct medication continued post-discharge I think.

HP4

R: Just as we've said, probably time, the saving the time, knowing that it's going to happen which was always kind of in the back of your mind that it might not happen, so reliability. It's just easier, we're not all running around trying to do faxes and stuff in the afternoon and cursing the fax machine when it doesn't work. I would say it's probably too early to really see if it's…it's difficult, I would say I've not really been able to assess yet the impact it's had to see for patients coming back in, because it's still quite early days. It will be interesting to watch over time and see the e-referral patients who then come back in and see maybe…it'll be quite an interesting thing to do actually, wouldn't it, just to see…if we had the baseline data actually, it would be nice to see if we had less problems, difficult to pick up because there's so many factors, but I can only imagine it's going to make our life easier in the long-term. But only if the discharge summary is right.

HP5

I: Still the same, right. And has there been any…has it taken any extra time to do this or has it saved time or what?

R: Oh, the e-referral itself has saved time. I mean, there is no joy going to a fax machine and having to wait for all those pages to go through and then at the end of it, it puts…it's not gone. And then you've got to send it again, you've got something else you want to get on with.

I: Yes, got to send it again.

R: And e-referral, in fact at the point of discharge, that's where the big difference is. I don't have to worry about sending any paperwork to the chemist once the e-referral is initially set.

HP6

R1: I think it's a great, great thing, it is very, very fruitful and a very efficient system because if you compare it with what I used to do, I still do that thing but actually I used to do faxing, we used to fax the sheets, the prescriptions to the pharmacists. I used to spend at least an hour and a half every day, on faxing.
I: Just standing by a fax machine?
R1: Yes, standing by the fax machine, waiting for reports and that time that I was spending on faxing, should be spent on returning, on saving money. So we are wasting time and wasting money. So, I used to average, let's say two hours and now it's no more than 15 minutes, average.

HP6
R1: Yes, so faxing is still there but the benefit out of it, is I don't like…what I want to say is, it goes like that, we don't even think about it, as long as the right information and everything is on the system properly, it goes by the system. The system does everything, which is really good, it makes a great, great difference and gives me chance to save more money.
I: Yes, to do the other bits of your job, rather than just standing by a fax machine.
R1: Two hours out of eight, is like 25 per cent of my work time…
I: Was doing that.
R1: Yes, a significant amount of savings, which means I can do different things, even if I walked a patient, directed him to his clinic or anything could be beneficial, more than stood next to the fax and chase up the…it's a nightmare actually. Nobody liked it but I volunteered to do it, it's not easy, nobody liked it, as I mentioned and it used to be my line manager's job and he was always bubbling under, because of that job.

HP7
I: Let's start then talking about the referral service, and one of the things I start off talking about with people is what you think it's there for, what do you think the point is, and what's it trying to do?
R: It's to increase patient safety and communication between secondary care and primary care, really. Because at the end of the day a fax machine is not very reliable, it's not the safest way to communicate. So, that e referral is a way to communicate to community pharmacy, that this patient has been admitted to hospital, to let them know. And, then on discharge it's a way to make sure that they know, no matter what, whether it be a weekend of out of hours, that they have been discharged and that they will get an automatic copy of the discharge. It's just a safer, more reliable way, and it's a way of us to communicate as well, what's going in the blister pack, what's not in the blister back. It's just more of an advanced way. More of an up to date way of doing it.

HP7
R: I mean, we were supposed to ring…when I went to speak to patients when they came in and they told me they were on a blister pack, we were supposed to ring and inform them and sometimes they don't answer the phone. Because they can be very very busy, if it's like a Monday morning, it's just busy; the lines are busy and busy.
I: Yeah, the pharmacists are busy checking…
R: So, we're trying to ring them back but sometimes you just don't have the time.
I: Yeah.
R: Whereas with an electronic referral you don't have to ring them, you can just do it electronically and you don't have to worry about whether they are answering the phone or not. No matter what they'll know, they are in hospital.

HP7
R: I think it's had quite a lot of benefits. Because it saves time ringing pharmacies, it's made us be a bit more careful; add more information, a bit more accurate on discharge. We've been able to communicate in a more timely manner to community pharmacies particularly out of hours.

HP8
R: Definitely, yeah. It doesn't take me a lot of time out of my day. It's fairly straightforward what I have to do with it and then when the NIPPS team chase it up they get quite quick answers about what's happened.
I: I think on that note, unless you've got anything...?
R: Just that in setting up the generic NIPPS email, that has then opened up more communication between the hospital and the NIPPS team, so (name) circulated the NIPPS email and said that you can send us non-urgent patient queries. So, again, I triage those and it's literally just this is the generic NIPPS email send something through. But we have had urgent patient queries sent through on them. So we need to work on our messaging.
I: Yeah, because if you're get anything urgent, because you're not looking at it regularly enough for...
R: I am looking at it regularly but we don't have the team in place that are in the patient surgeries. As I've said every...
I: Every day, no…yes, precisely.
R: ...like there's not a nominated pharmacy. It might be a week before a NIPPS team member works in a surgery due to...

HP9
I: That's recording nicely. So just to start off then, I want to start about the e-referral service more generally. What do you think it's there for?
R: So it's if you've got patients getting discharged from the hospital, then they inform the chemist, like if they're on a blister pack, they'll tell the chemist that the patient's been discharged so that the chemist can prepare the blister pack or can at least get in touch with the practice or know at least about the discharge 'cause where we've had problems in the past communicating between…from the hospital to the chemist. Yeah, sorry.
I: Is that what it's trying to achieve, that sort of communication?
R: Between – yeah – between the chemist and the hospital and possibly between the chemist and the GP practice as well, having that communication between them.

P3
I: …which is interesting. Have you used that chemists' all the time, have you always used (name of pharmacy),?
R: We've lived up here about 20, just over 20 years anyway, and I've always used that one. It's only the last year or so maybe, 18 months that I've had a blister pack, and…
I: Right, but it's always from there.

R:	It's always from there, they're usually great, you know, usually walk in, they say, (name), and they just, you know, and it's there in a bag all ready for me. It's only recently it's started to go downhill. But I put that down to this new system they had, and our surgery, because that's where the problem started initially…

4a 5. Code - Which patients should it or could it be used for - MDS patients or other

HP10.
I:	Yes, precisely. Yes, actually, when it started and they said, oh, we're just going to put patients who are on blister packs, and I thought, oh, that's not many. And then I thought, well, actually, that's the most complicated group of patients, isn't it? Because as you said, they're all multiple morbidity, many drugs and so on and so forth. Having said that, would you welcome any sort of extension of the service, to doing more than just patients on blister packs? Do you think it could be for lots of people?
R:	It's interesting, this, you know. I'm struggling to give you a good answer to that because I feel, or I'm under the impression, that the community pharmacy at the moment is underfunded, honestly. From my perspective as a community pharmacy manager, I think... I've managed to retain my staff so far, but there are a huge amount of problems getting someone, employing people for this money that we pay. So you don't necessarily have that many resources and it's hard to motivate people. And we're doing numerous things at the moment and I'm thinking, what will it look like? I'm not sure what we're talking about. I'm guessing that... Because someone said, maybe yourselves, someone said it to me, but there is a plan of rolling this out as all the patients who belong to you say, oh...
I:	Yeah, everyone with a nominated pharmacy.
R:	...I'll go to this community pharmacy, send it out. I think it would be great overall, it will improve things probably a lot, but are we able to take it on board, are we able to manage it? It's got to be well... That's my view. Well evaluated first before any next steps are made, in my opinion, because you've got to pick probably... I don't know who I'm kind of addressing that to, you need to pick up a few individual pharmacies and test it well, is it feasible or not? Because you will get a backlash from pharmacies, saying, give over, give us some money for it. I mean, I'm not sure. We don't get any funds for it, we don't get any money paid for it. You see, that's another thing. The money, and again, I'm not a business man, believe me, I don't run this business, this is not my business, I just get paid a salary. But I get loads of sort of, oh no, forget it, you can't get another member of staff on, no chance. We've got this, this. This pharmacy is not profitable, this is not profitable, we're going to shut this, we're going to sell this. So we get those constraints. If we had to do some more work with no reimbursement for it, I think my bosses would be: hold on a minute, give me a break, you know, and there will be, like, people just saying, can't do it. Pharmacists have got so much work to do, the pharmacy staff in my... I'm talking about my individual pharmacy, it's got so much stuff to do, so it's got to be very carefully evaluated and there's got to be feedback collected from a different kind of parties before any decision is made. That's how I see it. But again, I'm sure it will improve. I've got no data to prove it, but again, I can see it, it will improve overall safety, but is it feasible? That's the big question mark.

HP11
R:	But if we had other patients who were being discharged or anticoagulants and things like that, if we had that information and then when we'd…doing new scripts for them, we could tie that in more with doing a…
I:	Yeah, so if you had…where you've got patients who are on quite a lot of medicines, but they're not yet on a blister pack…?
R:	Yeah, so like patients who've had a heart attack or a DVT or…
I:	Yes. So where there's significant changes to new medicines or…yeah.
R:	Yeah, so we could tie that in with doing a NMS for them, which we'd probably tend to do anyway when we get the new script, but if we've got that information already… 'Cause if you just get a script for Rivaroxaban, you don't particularly know what's happened, but if we've got that information that says, this patient's had a pulmonary embolism, then when you go to speak to them about it, you're not like, oh, what's happened to you, sort of thing.

HP12
R:	Yeah. I don't know how you would define what groups of patients to do or how to, because I think if you did it for every single patient it would just be a nightmare to be fair.
I:	Yeah. And everyone… they have to have a nominated pharmacist.
R:	I was just going to say that. Patients use a variety of pharmacies, it's where would that information go, what would you do with it. I think it could be very useful, especially on patients who maybe have gone in hospital because they've had a myocardial infarction or something like that, or have been in hospital for a procedure and their medications may have changed a lot, but I don't know how you would decide what groups of patients to do it on. Maybe patients that have started on multi new medications would be somebody to do it for because then you've got that information, you can chase up how they are getting the new information, have they stopped what they should have stopped. I wouldn't like to be the person who has to make the decisions who to send it out for.
I:	Yes. It's interesting. In what way can you see that being of benefit, if it were in other patients?
R:	I am thinking of a couple of examples that we've had, so people that come out of hospital and they will come in and ask to speak to us and they will have like a big bag of medication, some of which they have got from the hospital, some of which they had at home, and I don't know what I'm supposed to be taking now, and they'll have like the hospital gave me these, I've got these. So, if we had that discharge summary, which sometimes they will bring in with them, 'cause they don't understand necessarily and things will look different and have different names and what have you. But if we routinely had that discharge summary, we could say, right those aren't needed, we'll keep hold of those, we will dispose of them, this is what you need. You haven't got enough of these, order some more of these or that kind of…
I:	So, it's patients who might be on multiple medicines but not yet using the blister packs?
R:	Yeah. So, they can manage the medication but it's just that confusion when you come out and you've got new things and old things. What do I get rid of, what do I…I'll just keep it just in case; no don't keep it just in case.

HP16
R:	It's also a relationship that we can leverage, we have a rapport with the patient and hospital pharmacy, it's an acute interaction as such. Yes, whereas for us it's a continuous interaction where we are seeing those patients or their family or their carers month on month.
I:	Yes, precisely.
R:	And we're having a different conversation with health promotion, other interventions but then the discharge becomes part of that regular conversation. And actually, do you know, I can see they've started something new, did they explain that to you in the hospital? Oh no, no, no, they didn't. Well, I'm pretty sure they did but you've just not absorbed it. That's what we're thinking but actually that's our opportunity to say, well, this is what it's for. Having that little conversation can make a big difference.

HP16

R: No, when we first scoped, even when we were talking about going live we talked about other patient cohorts it could go to, other messages that could be on there, other ticks boxes, but I think as a group they felt that they wanted it to be as simple as possible initially and then we could expand. But there's so many things we deal with, people who are new heart attack patients who need counselling on all the new medicines, they may have gone from zero to five medicines overnight and they need some significant support. There could be help intervention messages that could be done there, flu messaging, smoking, all those things they've done, they could have had smoking cessation initiated at the hospital that they need to continue in the community. Who's going to provide that otherwise, where are they going to go for it? They're going to come to the pharmacy where there's a patient service for it. And then the other part of it also is fluid restriction patients, patients from oral to liquid, can we get the liquids, what's the plan for them, are they going to be on long term, short term, patients who've started on a thickener possibly because of dysphasia. Does that impact on their other medicines because they might come in and tell us but we didn't know because you're on a monthly medicine, you've just had it three days before you've gone into hospital and you've been sent out with a load of stuff from hospital? So I don't find out that information until four weeks later when the next repeat's due and then it's all...those patients actually are all primed for information.

HP16

I: And a number of people have said to me patients on multiple medicines who aren't yet on blister picks would they be useful to be part of it?

R: Anybody is useful, if there is a medicine involved that needs some sort of intervention, that needs some sort of care advice, anything specific, and there's all sorts of things that could come up. And we've named a few just off the cuff but actually there are hundreds of different issues that we deal with that could impact on that patient.

HP17

I: Currently they just send out the e-referrals for people who are on blister packs, or Venalinks, or whatever. Do you think it would be of benefit if it were other patients as well, and, if so, which groups of patients do you think would benefit?

R: So, yes, it would be a benefit. We've just got to be careful. You don't want to flood the system. You don't want to log into PharmOutcomes and have 20 referrals and then you've got to do that. That's when you'd have the time wasting. If I turned on PharmOutcomes and logged in and I wouldn't know which was a tray and I've got another…I've got two trays in there and I've got 30 other patients that have been discharged but they're just normal patients and we don't manage their medication. So, we do have the repeat system where we order people's medication and they verbally, or physically written, confirm what medication they want in four weeks' time, or eight weeks' time. It would notify us, so we know we could cancel that and put a note on the computer, they're in hospital, but that's when you get to the limits as well, you're creating more workload. So, we're not…I think just doing trays is great. I think you'd need to think very carefully whether you'd want to do more than trays because that would then create more workload. I mean there might be…if there was another way to separate the referrals, if they're just normal community patients that are on medicines, and not mix that up with the trays and you could log into some sort of system and know that they've been in hospital. I mean you've got the summary care record but then you need to have permission from the patient to access the summary care record. So, I mean you've got to let…where they're talking about letting pharmacists look at GP records and sharing records with pharmacy. So, it's something for the future where everyone's got access to everyone's records, well community pharmacy can access GP records, and it's all streamlined and you're all on the one system, that will be great, but you have got summary care records but then you need permission to look at that, unless it's an emergency.

HP17

R: Yeah. Then you get to the point where you say, well too much information. So…

I: And too much…yeah.

R: …because we manage tray patients in so much detail down to what time of day they take their medication and you need…it needs to be perfect, there's no room for error, you need the tray ones. Whether you need more than that at this moment in time I'd probably say, no, we've got enough to be going on with. But further down the line when you sort of integrate GP records with pharmacy, or some way that pharmacy can dial in and look, will be helpful, but then you've got the time issue that we're so pressured in pharmacy every second is important.

HP3

R: Yeah, I'd say. And I'd say it's even been helpful in other cases where the patient doesn't even have a compliance aid, because there was a patient I had a couple of weeks ago who was a methadone user. We tried to get in touch with the pharmacy, but I guess their line was just busy or it was just going straight to voicemail. And that is an important case, to make them aware that this patient is in, so that no one else collects their prescription in the pharmacy.

I: Yeah, precisely.

R: And so I sent through a referral to the pharmacy after consenting with the patient, just to make them aware that they're currently in, even though it wasn't a compliance aid, it was just so that they were aware that the patient was currently in.

HP4

I: And we were talking about MDS and just the thing I wanted to come back to and about Venalinks and blister packs, MDS, compliance aids, whatever you want to call it, currently that's the only group of patients that the service is being used…is focusing on. Do you think it should focus just on that or do you think it could be used for discharges more generally?

R: Yeah, it's interesting, isn't it, because that section of the discharge summary is so poorly documented at the moment in terms of stop-start, change medication, I actually think it is also really useful sometimes just to notify the community pharmacy of changes generally. Because you've still got the same situation, imagine you've got your 80-odd year old male who's on lots of medication, we change everything in hospital, then we rely on the GP stopping that prescription say for the aspirin, you know, once they get the discharge summary, then say a secretary looks at it then passes it on to the GP, the GP then puts it in their pile of things to look through. A couple of days later eventually, maybe they'll get round to stopping the aspirin off the repeat prescription, but by that point, say like another lot of prescriptions has gone out or they've got one at the chemist, by the time the chemist gets the next lot of prescriptions, they could easily have got another supply of medication stopped. And if the patient's not totally with it with their medication, which lots of people aren't, they'll just take whatever arrives, then it definitely would, you know, you can see how it could work there as well. I sometimes have used it for stuff like that, so I know technically we're not meant to, but I have used it a couple of times for patients who aren't blister pack patients, and I've used it for more like a communication tool almost. So I think the two I use it

for are for nursing home patients, and I wasn't sure to begin with whether they had blister packs or not, I can't remember which nursing home it was, but I rang the chemist just to check and they said, they're not blister packs, but they said to us, if you make any changes, would you let us know because they're due another month's supply of medication to go out this week. And I was like, yeah, that's fine, so I put a referral on and I put like in the comments section, and b) this patient does not have blister packs, but this is just to highlight any changes on discharge. So in that situation, I've already kind of used it for that to some extent, because I don't see why you couldn't.

HP4
R: Yeah, that's right. I mean, it's like you could see it like being developed in loads of areas to be honest, we sometimes use it for methadone patients for example, if you want to let a community pharmacy know that they're in hospital, we've used it for that before. So they get notification on discharge, because again that relies on…we'll ring them on admission and say, this patient's in and they say, can you ring us on discharge, and we'll be like, well, I'll try and get someone to ring you, but they're going off to another ward and it relies on whoever's on that ward, and if they've got a pharmacist, and if they're discharged on a Sunday, probably no one's going to ring you.

HP6
R1: So it's a dramatic decrease in time-wasting. I'm not wasting time on faxing now but as you know, the e-referral does not cover the system, does not cover the whole pharmacies in (place) or in the UK, it just covers about 60-odd pharmacies, in (place). So still there is lots and lots of pharmacies, outside the area but luckily, we don't have patients, lots of patients coming in from…

Theme 4b. Nature of the Network - agents and how it operates

4b 1. Completeness, fragmentation or incompleteness of the network

HP1
R: Yeah, I mean I try not to leave it a week, but it depends on them, like (name of practice) are the largest in (name), so each of them are over 10,000, so there's many discharges a day, so yeah, so generally maximum a week, yeah, and they're getting it after two hours. So it's kind of, they're calling me, well I've not got to that yet, just one…so it's a bit of a waste of time.
I: So, because it's automatic, because that's the point of it being electronic, it's that quick?
R: Yeah, which is good, it's definitely good, but it would probably be better if we had it, because then we could then action it all, because the community pharmacy can't really do much, apart from tell us about the change, and then we make the change. Yes, they can amend the dosette, but without an actual prescription, it's risk for them, so some pharmacists, I know from working in community might not want to change anything from that, they want an actual prescription to see, even though…
I: So, even though [inaudible 00:14:19] doesn't need, or it needs x instead of y.
R: Yeah, I don't think many, especially the inexperienced pharmacists, the newly qualified pharmacists, I don't think they'd be comfortable to send out a Venalink, just from a discharge summary, 'cause they're still quite young and inexperienced, they would like to see a valid prescription, to say, you know, what the patient is having, and then they will send it out.
I: And that's not going to happen until general practice know?
R: We do it, yeah.
I: And then issue the scripts, and then the [voices overlap 00:14:57]?
R: Yeah, and it might happen by the community pharmacy calling the practice, and saying, by the way, do you know this has changed, but is really a useful way of…?
I: It's more work?
R: Yeah, it's more work, because we would have got to it eventually, so that phone call is probably just a waste of time, maybe, or just extra work that didn't need to…if we had it initially, and then we would prioritise those discharges and say, this is what we're going to be doing first, 'cause they take priority over clinic letters, we could have just done it there and then, and I don't know, yeah.

HP10
I: So they faxed it but then there was also some informal communication then between you and the GPs?
R: Yes, it was difficult. I think ever since the GP surgeries took on, they developed... Like the surgery next door to me, they developed this, what they call prescribing team. They will have a pharmacist or two pharmacists in charge of running it, they will have prescription clerks, all these people are doing all activities with regards to prescribing prescriptions, discharges, you name it, all the safety of meds. Where before, it was really... The next door doctors, unfortunately, it was all over the place, in my view. There was usually a doctor leading this, signing, but there was no one really taking full charge of every single person was issuing. It was utter chaos. Again, I'm talking about this particular surgery next door to my practice that we got 90 per cent of prescriptions from, so it wasn't very professional, it wasn't very sustainable, it was just chaos. So ever since that has been introduced, they very much...you know. We used to engage with whoever was answering the phone in the past. Now there are people that we can relate to, exactly. We can either ask for (name) or this that or the other. So this is the way now. In the past, it was just...

HP10
So we try to make sure that the next door pharmacist, doctors, whoever is managing the discharge, will know exactly when do we need that set of prescriptions, see if there are any changes for. And then we're looking out for scripts. Recently, it has become better and better. We will sometimes obviously chase things up or we will receive things: oh, there is an error here, there is an error there, we will question things, but sometimes realising they don't always necessarily always agree, the GPs, or with what's on that discharge, they will make their own changes, they will make their own little implementations, you know, whatever they do. We, ultimately, do what we're told, but at the same time, we watch their hands, we're making sure that this is going to be right. So we will be in the habit of still double-checking. We receive something that is in contrary with what the district was saying and we'll say, guys, is that definitely right, that furosemide, is it definitely...? It says there 40 and you just dropped it to 20. Oh yeah, because she's doing a blood test in a week's time so we'll then... So we always have that verbal communication, and once everything is cross-checked, then the preparation will start. We'll just prepare it. I always do an in initial check against the discharge. So, to be honest with you, my, (name) who does the blister packs, she's really good, she's so good that she always... I check but it's hardly ever that I pick up any errors or discrepancies, but I do it anyway, I'll always check the information on the script against the discharge initially, and then once I'm happy with that, then I will do whatever checks need doing, and then it's going out. So, yeah, I think that's the best way I can describe it really, giving you that kind of journey of that.

HP10

I: Who are the important people there, yourself or...?

R: […] I think myself, initially, and then everybody was involved. Obviously I briefed everyone. I will hold meeting with staff, so I either do one-to-one every month or so or like a group meeting. So just at one of the meetings I was saying to everyone, listen guys, this is the way they're going to be doing...letting us know about this, people coming out of hospital. So just keep your eyes and ears open for anything and let me know, let (name) know about it, because she is the main person. But if (name) is off, there's usually another lady who takes over, so she's been made aware of it. Everybody knows her. I've got a relatively small team, it's not like we've got hundreds of people. It's just a group of five people, so it's fairly easy to just... We communicate things on a daily basis that are happening and changing, so everybody is aware of it. But myself and (name) will be the main people who will be paying attention and looking out for anything coming through.

I: And wider, beyond that, are there any...?

R: Beyond that, obviously I will have to mention, I guess my head office. The superintendent office, they've got a group of... There's a team who will facilitate a lot of things for us in respect of professional services, patient safety, clinical governance, as a whole, a bunch of activities. So they will be the people, like I said to you initially, they were the ones letting us know about things, reminding us about things, telling us when it will all start. They always read emails and then they just make sure... We should receive them but they know very well that we're bcc'd, so they're kind of trying to see [inaudible 0:18:02]. Whatever is very important, we will be made aware with big red letters, emails, saying, this and that is happening. So they definitely played a role initially in it. And that's it. Within my organisation I cannot think of anyone else. Obviously if we're talking wider about it, it's the engagement of the people on the other end, for instance at the hospital, I think they are quite important. I had some little issues there and they will always immediately communicate back to me, things that happened within that referral scheme. And obviously next door, as I said to you, that prescribing team, they are aware of it, I spoke to them about it.

HP12

R: It's impacted on the way we work in the fact that we get the information much quicker and everything is done in less of a rush.

I: Yeah.

R: Which I think is quite a safety issue. So, we've got a lot more time to sort everything out. We've got time to sort the prescriptions with the surgery; they've got time to look at it properly, to work out everything. So, that's worked much better. And we're not bothering the surgery saying; please can you print me out a discharge summary.

I: Yeah, because you've already got it.

R: It sounds awful to say but if the surgery just do us a new lot of prescriptions, we prefer to check that against the discharge summary, just to make sure that everything is as it is on the discharge summary. I know the doctors have issued the prescription but we prefer, as a double check, to check it against the discharge summary. So, sometimes they were getting a bit…why don't you get one and blah blah…so, we've got that information and like I say, it's the other way round now, we are taking it through to them saying, this patient has been discharged, here is the discharge summary, please can we have prescriptions.

I: Which is really really interesting.

R: Yeah. And we've got quite a good relationship with the practice pharmacist that's working at the surgery now, the previous one we didn't have, but that's another story! But this current one we've got a really good relationship and he's just like, yeah, if you can print me off an extra copy that's great, it's much quicker.

HP13

I: In terms of communication, you talked a little bit about the communication with the doctors there, what about the communication back and forward to the hospital, is there any communication there at all?

R: No, 'cause you can't really get through to (name of hospital) much, the pharmacy, you wouldn't be able to actually ring them up, no, you don't really need to with this, to be fair, 'cause once they've told you what the patient's medicines, it's all there so you wouldn't need to, I've never had to phone them up.

I: Right, yeah, 'cause one of the things that I was thinking is, you know, it's a bit linear, the information is coming out of the hospital, coming to you but actually, once they provide you with that information, that's all they need to really do, isn't it, unless there's any queries that you…

R: Well, if there's a query, to be fair, I think actually I would pass that on to the GP, if there was a real query. Because at the end of the day, I'm not the clinical person producing the prescriptions, so you know what I mean, if there was an actual, real thing that I thought, I'm not happy with this, I'd put on PharmOutcomes that such and such doesn't look right or whatever and inform the GP and then I'd phone the GP. I mean, I don't rely on just the email on PharmOutcomes, I do actually phone them if there's a problem, I would throw that back to the GP, not the hospital and I'd let the GP deal with the hospital, yeah, really.

I: There is that process of talking.

R: But we…I don't think many pharmacists would ever really try to get through to the pharmacy at (name of hospital) about anything, I've never had that arise, really, no.

HP17

So, we're…one thing to tell you that is our branch here is probably a really good branch for you to pick for this study because we're a tray hub. We manage nearly 200 patients, or probably 200 plus the ones in hospital. So, it's a lot of pressure here. And we've got one girl, (name) that's spending…a full time member of staff, apart from…more or less full time, apart from on a Thursday, not that that's relevant, but she's constantly working on these trays, banging them out, constantly liaising, very good lines of communication with the surgery, with the hospital, with the Warfarin clinic. So, it's multidisciplinary, multiple different agencies, with the patients, with the carers, with the nursing homes. So, you've got to have good lines of communication.

HP17

I: Yes, and I'm thinking in terms of workload that has an impact on…

R: Yeah, it…and it delays things, and then you could just a patient turn up saying, I've got no medication. And so, well no one's told us you've come out. See, that's another issue. So, for me an action would be to involve nursing homes on this scheme as well. So, you've got the hospital, you've got the surgery being notified, you've got the chemist being notified. What you've not got is the middle man, which is…so there's the hospital, there's the respite people, whether it's a nursing home, whether it's some temporary NHS centre that's linked to the hospital that

they might be in for a week/two weeks/three weeks, and then they go back into the house. So, you're jumping from the hospital and you're missing that and you're jumping straight to the residential, but they need to be involved on the scheme as well.

HP2

Right. So, when a patient comes in, we will go through a medicines reconciliation. So, we will go through all their meds, what they have and we'll do the e-referral. On discharge, if a patient is going home, the nursing staff or the doctor will let us know. If they need anything…because a lot of the time they will bring medicines in with them, so, we don't have to re-prescribe and we don't have to send them home. But it's if they need antibiotics or pain relief for anything new. And then once that discharge summary is done, then that should go automatically to the chemist via e-referrals, by PharmOutcomes. Yeah.

I: So, in terms of, you know, what you then do, you do the meds rec in the beginning…

R: Yeah. We do the meds rec…

I: But then you do, any changes.

R: Yes. So, if there are any changes throughout their hospital stay, when the doctor writes the prescription or electronically writes a prescription, that will then be clinically checked by a pharmacist. So, they'll check that there's nothing wrong with it. If there is anything wrong, they will get them to change it straightaway. They should then do the changes electronically and then that can go to the chemist. And one is given to the patient, the chemist and the GP, I think. Yeah, all three. So, they all have a discharge summary.

I: Yeah. I think most of the information goes to the community pharmacist. Do you think it should be going to the GP there as well?

R: Definitely. Yeah.

I: Yeah. That's really interesting. Yeah.

R: Definitely. Because on the occasion…some occasions I'll ring the community pharmacy and they've not had…they have changed something, but it's not on the patient's SIR record. So, there's a discrepancy…why does he not know that but they know the changes. So, I think the GP definitely should.

HP3

I: And who are the important people in the using of the service? I mean, as well as thinking more broadly, who are the important...?

R: As in the hospital?

I: Yeah, in hospital or in the community or whatever.

R: I'd say it would be us, because when patients come in, we're the ones that would have to identify that they're on a blister pack and we would send the referral out to the pharmacy, it wouldn't be the community pharmacy letting us know that. So it would only be one way, I guess, the referral, yeah.

I: Yeah, so it's not... That's quite interesting, in so much as that this is purely you sending the information to the community pharmacy. Do you ever get anything coming back from them? […]

R: No.

I: So it's only...

R: A one-way.

I: It's a one-way street.

R: Yeah. And so I guess if they did have a concern...

I: They'd phone up.

R: ...they'd have to, yeah. So if there was something in the discharge summary that pharmacies aren't sure about, they will just ring us, pretty much, and there isn't anything that they can send through. But, to be fair, if they did send something through, the patient would still have to be here, they'd have to be reviewed, because I guess there isn't a system in place to see patients that have been discharged if the community pharmacy has an issue with it. Because when the patients are discharged, we move on to the next patients that are currently here. So I guess it should only work as a one-way system, to be honest, and then they would ring us if they had any queries.

HP4

I: So that communication, how can that help with that safety or how is that going to help…?

R: Because inevitably I see it all the time when patients are discharged from hospital, we see so many problems with mistakes, errors, prescribing errors, when patients go from one setting to another, when they go from primary to secondary, secondary to primary, you're constantly facing that battle of mistakes in that transition, and the aim really is to make sure it's communicated reliably. So if the chemist are getting an electronic discharge of like a snapshot of exactly what they were discharged with, that takes out that kind of element of, you know, they've got the information there so they can then hopefully act on that quickly, much more quickly than relying on a fax two days, three days later where there used to be quite a significant delay, things can get changed quicker, so if it's blister packs they can get their new blister pack up and running, they can nag the GP to get the new prescriptions through. So hopefully the next time the blister pack comes out, it will have the correct medication in, not medication from like preadmission.

HP4

R: It's a complex…it's really, you know, discharge, admissions and discharge is really complex business, isn't it, in terms of… And some of the factors, you've got carers, patients, relatives to throw into the mix on top of all the other stuff, it's just…

HP4

R: Yeah, that's right. I mean, it's like you could see it like being developed in loads of areas to be honest, we sometimes use it for methadone patients for example, if you want to let a community pharmacy know that they're in hospital, we've used it for that before. So they get notification on discharge, because again that relies on…we'll ring them on admission and say, this patient's in and they say, can you ring us on discharge, and we'll be like, well, I'll try and get someone to ring you, but they're going off to another ward and it relies on whoever's on that ward, and if they've got a pharmacist, and if they're discharged on a Sunday, probably no one's going to ring you.

HP6

R1: So let's start in order, so once I open that rejected Venalink prescription, I find out the reason, I write it on a log and that log goes to (name), the pharmacist whose in charge, to follow up and change something on the EPR, which I'm not allowed to, I'm not authorised to. Once I write it on that log, I try to follow the steps, if the patient is not with that pharmacy, he doesn't deal with the pharmacy, he or she, then I try to find out what the right pharmacy is. So sometimes, what happens, they pick up, on the system, the wrong pharmacy, so because the map, it's not very,

very clear and especially under work pressure, people tend to, when they see the area and see that sign of a pharmacy, they click on it, while there is one very close to it, carrying the same name but in a different lot and then mistakes happen. So I try to just make sure, go on EPR, check the right address, check the right telephone number, try to speak to them if there's any need for that and this is one of the easiest things to do, actually. So I just click on the right pharmacy and say that it's done. Well if it's the wrong pharmacy on the EPR, then I have to report that to (name) or the person who is dealing with the EPR thing, who have authorisation to go and change things over EPR. As I mentioned, I don't have that authorisation. So this is one scenario, there could be other reasons, like I come across today, a patient who has been rejected by (name of pharmacy). I went on the EPR, checked the records and I found out that this is the right pharmacy that is mentioned on the EPR, it's not the wrong pharmacy but the reason for rejection, that (name of pharmacy) is not doing Venalink for (name of care home) anymore. So the patient has been sent out, discharged from the hospital to (name of care home) but (name of pharmacy), who receive the information from the hospital, is not dealing with (name of care home) anymore, it is another pharmacy. So I spoke to them and I found out, at the beginning, because maybe they are under pressure, I've asked them, do you know who is dealing with (name of care home), which is a logical question. They said absolutely no idea and I was shocked, that was a prompt answer, which I didn't like and then I asked them, is anyone around you. No, she said I don't think so, but then she said, hang on, hang on, maybe someone was overhearing that conversation and said (name of pharmacy)Then, I went back to (name), because I don't know where that pharmacy is, (name) has much more experience than me and she said, we have a list of the pharmacists that do Venalink for care homes and yes, I found it on the computer and then I found the number for that pharmacy, I rang them and I confirmed that. It was listed on our PharmOutcomes anyway, but I just wanted to double-check that this is the right pharmacy before letting it go to that pharmacy. I called them and they confirmed the address that I mentioned on the PharmOutcomes and I saved it on our PharmOutcomes, so that it's gone to the right place.

HP8
I: What happened then? When it went live around about February or just before that, was there any further, were you involved in any further, you know, from there onwards, the development of the service at all?
R: Yeah. So after that initial conversation with (name) we had another one before the launch to say, well, to ask me whether I would be happy to basically triage any communication that came via the e-referral system from the community pharmacy back to primary care. So my manager, (name), asked me to set up a generic email address, an nhs.net address so that the reports, the e-referral reports, could be sent there and I could check those.

HP8
I: And then in some respects therefore, how does that then, that what that is for, fit in with what you're doing in your role?
R: So, yeah, it's that extra step that if there is a query about the patient's medicines, that doesn't have to go back to the hospital, that can go back to either the patient's GP or the NIPPS pharmacist who potentially has the time to do a proper in-depth examination of the patient's medicines and make sure that they are on the correct medicines. So, yeah, it's adding an extra layer of safety and an easier way to communicate, I think.

HP8
I: And then they get on to resolving whatever the issue is. Within that how does that impact upon the GPs, is that creating extra work in general practice generally or?
R: I think so. I think when the community pharmacy communicates with the GP surgery outside of the e-referral system, they might say, oh, yeah, the patient needs to see the GP, so the GP will then make an appointment. Whereas, actually one of the NIPPS team might be even better placed to do that review than the GP.

4b 2 Code - Different actors in the network and how workflow might be different for each of them.

HP10
I: So they faxed it but then there was also some informal communication then between you and the GPs?
R: Yes, it was difficult. I think ever since the GP surgeries took on, they developed... Like the surgery next door to me, they developed this, what they call prescribing team. They will have a pharmacist or two pharmacists in charge of running it, they will have prescription clerks, all these people are doing all activities with regards to prescribing prescriptions, discharges, you name it, all the safety of meds. Where before, it was really... The next door doctors, unfortunately, it was all over the place, in my view. There was usually a doctor leading this, signing, but there was no one really taking full charge of every single person was issuing. It was utter chaos. Again, I'm talking about this particular surgery next door to my practice that we got 90 per cent of prescriptions from, so it wasn't very professional, it wasn't very sustainable, it was just chaos. So ever since that has been introduced, they very much...you know. We used to engage with whoever was answering the phone in the past. Now there are people that we can relate to, exactly. We can either ask for (name) or this that or the other. So this is the way now. In the past, it was just...

HP10
I: And that surgery has pharmacists working in it as well?
R: They do. They do have a pharmacist, I'm pretty sure. I don't know how the project is called but employed by (name of NHS trust), and then there's another guy, this is a different kind of way, where he's more of an independent prescriber, but I think they work together. Both of them are happy to look at the... At the moment, when they get a discharge, they will look at the changes, they will implement whatever new meds need implementing and put them on prescriptions, put them on repeats, make sure that the blood tests are arranged for the patients to be done, with regards to certain medications that was asked by the hospital setting. So, yeah, it works much, much better and I'm already kind of going ahead, but in the past it wasn't the case.3MAJ29/03/2020 18:36
I: You said something which is quite usual in GP practice and is that just very... Do you think that's something specific to that practice?
R: No, no, no. In (name of place), you see, I know very well that lady called (name) who is employed by (name of NHS trust), she works...they call it a cluster, so she will work within a few pharmacies and pretty much...one, two, three, three surgeries now I work really close to, they all employ the pharmacist now. So, as you may know, you might have heard on BBC News or in the papers, that the GPs, the primary care, are now trying to get pharmacists involved to facilitate more time for GPs and then, like, physios, because apparently 20 per cent of problems that patients are coming through the door are to do with musculoskeletal problems, so...
I: Is that widening of general practice isn't it

R: Yeah, exactly, so pharmacists are very much now an essential part of the surgery, I think, there's no exception that this one is, I think it's overall. And they all engage with... Again, to me, my understanding is they engage with all kinds of activities to do with prescribing, every single thing I'll be talking through pharmacists. Very rarely these days I will speak to the GP directly unless they've got something, you know.

HP11

I: So what sort of…who uses it here then? Who's involved in doing things here, I think, 'cause you mentioned somebody else who…

R: So (name), and I and possibly the pre-reg, we've just got a new pre-reg…we tend to be checking the emails and then we've got the PharmAlarm thing now. So we'll tend to go onto PharmOutcomes and print the information off and then pass that onto the lady who's physically doing them and she tends to liaise with the surgery about the scripts. She'll go and say to (name),, this patient's been discharged, here's the discharge, when can I have the scripts? And we need them for this…the [inaudible 11:12] needs to be sent out.

I: Are most of your patients at that surgery next door?

R: Yes.

I: That makes life…must be very…makes it…

R: Especially our Venalink patients. Yeah, we do have a lot of patients who are from elsewhere, but most of our Venalink patients are…yeah, we've got…

I: And it being such a big practice.

R: Yeah, we've got the odd one like…it tends to be like parents of staff who are a different one…who are at different practices that we've taken on. But yeah, most of our…yeah, I would say probably 98 per cent of the Venalink patients are next door. 'Cause it's like…you're right, it just makes it so much easier.

HP12

I: Who are the different people who are using the e referral service do you think?

R: So, there is obviously myself and (name), the pharmacists, our pre-registered that we had, she has just actually finished so our pre-registration pharmacist, she has just qualified; so I've got a new one but I don't think he's had any. He only started last Thursday so I've not had any since then. Although we did have one this morning but he is on a training day this morning. And then (name), who is the lady that deals with the venalinks. So, she knows how to do all the things and get all the information.

I: Right.

R: She can log on and look at it. She would probably never accept or complete any of them without our say so, but she knows how to go on, view the discharge letters, print those off.

I: Right, that's interesting. And beyond and outside of that, who are the important players in the system, beyond here as it were, do you think?

R: So, obviously the pharmacy staff at the hospital that are preparing the discharge quickly and getting it to us. The staff in the surgery who now ask us to take a copy of the discharge letter in, because we get it quicker than they do now, so that works quite well. And obviously the patients and families.

HP13

I: Well, yeah, precisely, whereas with the fax, a piece of paper, no one knows what you've done.

R: And I think…as far as I understand it, that feedback that I do, saying that we need another prescription or whatever, all that gets emailed straight through to the GP so they've also got another record, you see, at their end…I Right, that's interesting…R …which is good because if I had to ask them for a prescription and they're just not playing ball and they're not providing it, it's, there in a record and they can see that I have asked. They can't say three days later you've not asked us for a prescription. I can say, well it's logged when I phoned you on da da da and they have the email of that.

I: So they get emailed from that?

R: Yeah, and I do them promptly, I do them if…well, before the PharmAlarm thing I might've done it…it might've been the next day but now we do them more or less as soon as we get them.

HP13

R: Well, it's not hugely busy but it's sort of, medium-ish ones, 'cause we've only got the one, it's not hugely busy.

I: Yeah, who do you think are the important people to the pharmacy referral service, who do you think are the most…the crucial people to make it work, as it were?

R: Probably in some ways it would be the dispensing staff, you know, because as the pharmacist you may see that a notification has come through but you maybe haven't got the time just then to open it up and really view it; it's possible that you get interrupted, you're checking something, whatever. I don't know, possibly but then I have to oversee it anyway as a pharmacist so that I'm still involved. It's a team thing, really, isn't it.

I: Yeah, that's interesting.

R: But in a way I probably rely on my staff to alert me that something has happened in the first instance and then we open it up and have a look at it.

HP16

I: Do you think it would be better if it was more of a triangle…?

R: I think it would be better because what we would do is we would see inter-referrals, so actually what we want to see is the discharge MRU come to us but then the practice pharmacist could see I've done a discharge MRU and a quick bit of note on it, these are the couple of issues that have come up, can you deal with those, please? And vice versa, they could say, actually I've just started somebody on an inhaler, can you MNS them for me? And the same way the GP (inaudible) goes why can't they refer to us?

I: Yes, so there would be a referring back and forward between?

R: Yes, but it would be one...[…] joined up approach so that if that patient then went back in the hospital pharmacist

HP17

So, we're…one thing to tell you that is our branch here is probably a really good branch for you to pick for this study because we're a tray hub. We manage nearly 200 patients, or probably 200 plus the ones in hospital. So, it's a lot of pressure here. And we've got one girl, (name) that's spending…a full time member of staff, apart from…more or less full time, apart from on a Thursday, not that that's relevant, but she's constantly

working on these trays, banging them out, constantly liaising, very good lines of communication with the surgery, with the hospital, with the Warfarin clinic. So, it's multidisciplinary, multiple different agencies, with the patients, with the carers, with the nursing homes. So, you've got to have good lines of communication.

HP2

I: And do you think it should be…is there a potential to extend it beyond just MDS or beyond dosette boxes, beyond Venalinks to discharge more generally.

R: Every patient? I think it would be a really good idea. Yeah. For us it would be a really good idea just to let the chemist know when a particular patient is in. But for the chemists it will be…basically we are passing our work on to them. And I've had this discussion with a particular pharmacy here in (place), who said to me, I don't like this system at all. So, I said, why? It's great, do you know, she said, yes, but you've passed your duties on to us. Because now they have to go to the computer every morning to look, to see if they've got an e-referral…a referral and it will say, oh, we're coming to have a look to see who's in hospital. Oh, right, sort all their…what we're going to do there…blister packs today, no, so should we do that. She said, but then all through the day, we are constantly looking. So, it's taking time out for our staff to keep looking to see if there's been any referral to say somebody is in. So, we don't have to do the blister pack. Whereas beforehand, we were waiting, until the end of the day to fax them, they got hard copies, so, they didn't have to keep going looking at the computer. So, they were relying on the paper appearing on a fax, now they have to keep going to look at the computer, to logon, to put their code in or their password. So, it's causing them more time.

HP3

I: Yeah, precisely, so they know it's not going to be collected, and so on, which is really important. Beyond then just those sorts of things, are there any other reasons why it would be good to have it used more widely, or are there any challenges in using it more widely, do you think?

R: I'm sure there are challenges using it more widely. I guess one of the challenges would be, because we're inputting into the system, this is the pharmacy, and then we send through the referral, if we send it to... Like I said before, there are so many (name of pharmacy). Say I send it to the (name of pharmacy) on the other side of town and they've got all of this patient's information. I'm not sure, I think on their side they have to accept or reject, they can accept or reject.

I: Yeah, I think there is something, but I think if they reject, I think they then get phoned up by somebody, or something.

R: Oh, okay.

I: Something happens; I'm not sure.

R: So I'm not sure how it works on their side but I guess it is a case of patient confidentiality, because I've sent it now to the wrong pharmacy, and is that an issue with that? Say it was as pharmacy that the patient used to be at but they no longer want to go to, and they don't want them knowing any of their business.

HP4

I: Yeah, right. And who do you think should be using it, should it be restricted to just community pharmacy and hospital pharmacy or are there wider people or do you think that's about right?

R: To be honest, I'd not thought about…do you mean in terms of like GP practices and things like that?

I: Yeah, yeah.

R: We have a lot of…so it's kind of really exploding, the whole community interface at the moment with all our practice-based pharmacists, NIPPS pharmacists, it's really taken off into this new area which we'd not really delved into before. It's quite interesting to think how it could be used. In some ways actually if you had a practice-based pharmacist thinking about it, it would be really useful for them to know that that patient's in hospital, but they would be notified on discharge anyway I guess because they would get the discharge summary, wouldn't they, so I'm not sure how it would work in…currently we just use it for blister pack, for Venalink patients, don't we?

I: Yes, yeah, precisely.

R: So I don't know if it would have any particular benefits, I'm not sure. I think for what it's used for, I think the right people kind of are involved. It's quite a specific thing we use it for currently.

HP4

I: So how often are you doing it, daily?

R: Yeah, oh yeah, because me and my colleague, we tend to work on the elderly care zone of EAU, or we work on the whole ward but we prioritise the elderly care patients…

I: Yeah, it's been explained to me that you've basically got an elderly care part of that ward.

R: That's right, yeah, yeah, that's right, so we tend to prioritise those kind of patients, mainly because they're high risk, they're polypharmacy, they're on lots and lots of medicines, and they're also high risk of medicines-related admissions, because they're in and out of hospital, they have lots of changes to medication. So these are the kind of patients who really benefit from this e-referral system, so because we kind of work on there, we use it a lot because 80 per cent of them have blister packs, so I'd say I probably do, I don't know, maybe four, five a day, quite a few.

HP4

R: Are continued, yeah, that's right. The only…I guess the only downfall slightly is that our system doesn't necessarily show that it's had a pharmacist clinical check. So if a patient's discharged but the nurses or the doctors don't tell pharmacy about the discharge summary, that discharge will automatically still ping off to the community pharmacy, but it might not have had a clinical check, so there might still be errors on that. That's one of the big risks I think in our kind of system…

I: Because unless they make any changes, they don't need to involve you, is that what…?

R: So we try to encourage doctors and nurses to inform us of all discharges basically so we can cast our eye over it, because a lot of the time you do see doctors writing, no changes to medicines, or… We did a recent audit on EAU where we found we changed nine out of ten prescriptions in terms of documentation and prescribing. So if you imagine that and if you imagine that say out of hours overnight if pharmacy aren't open or if a nurse just says, oh, there's no changes, I'm not going to bother telling pharmacy, we'll just discharge the patient, and then that automatically goes off and those errors are continued.

I: Errors can carry on, yeah.

R: That is still a risk, but it's difficult to get around that, so other than encouraging staff to inform pharmacy of all discharges, problem is pharmacy aren't there 24/7, at the moment we just have 9:00-5:00 really, 9:00-7:00 on EAU, so that is still a risk. So the problem with this e-referral I guess is that you can't guarantee it's necessarily going to be correct. If a pharmacist has been involved, you'd hope it was correct, but you

don’t necessarily… So that’s why we annotate I guess if we’ve seen it or not, if we’ve clinically checked it, it puts our electronic stamp on it to say, we have clinically checked this. If it doesn’t have that and there’s errors, I guess the community pharmacy could then ring and say, is this right, has anyone actually seen it from pharmacy, is it…

I: Because you do the order at admission, if you then don’t get involved in the discharge, it goes without…

R: Yeah, that’s it, it goes. It goes, yeah.

I: …you having anything…

R: That’s right, yeah.

I: And it’s about keeping people…

R: It really is, yeah, so there is still an element of communication and unreliability to some extent. It’s just better than it was. Yeah.

HP5

I: Yes, yes, that's really interesting. In fact, that brings us back to these other points about who you think should be involved in the e-referral service, who should be using it?

R: I really think pharmacists, pharmacy technicians, I'm not sure. I think primarily the two of them, only because the e-referral information is for the community pharmacy and most of the liaising will be with the community pharmacy. However, we do get instances where perhaps doctors, especially out of hours will need to know about certain information with regards to patients. And they are told when perhaps we've gone home that a chemist might be open a little bit later and they might need that information. If they are competent, they can put an e-referral on. But I do believe primarily the pharmacy staff because they are understanding of the e-referral system, how it works, so it's better.

HP8

I: And the e-referral... Well, actually let’s move on to the e-referral. Let’s go back to, you came into your present role in November, so presumably that was when you first heard about the e-referral? What was the first thing you, take me back to that time when you first heard it?

R: So I met (name) at the hospital who was on the NIPPS team but went back to the hospital fulltime and she mentioned it, so that was the very first I’d heard about it. And she said that it was a way for the hospital to communicate with community pharmacy. So when a patient is discharged, their information about their medicines will be sent to their local pharmacy that they use, whether that’s their (name of pharmacy) or their (name of pharmacy) or one that’s at their GP surgery. And she just mentioned a brief sentence or two saying that the NIPPS team might then be a part of it at a later stage and it would be, like, a three way communication between the hospital, community pharmacy and the NIPPS team.

HP8

I: Right, I’m with you, yes. So in terms of the people who are using the e-referral service, you’ve got a group of hospital pharmacists, you’ve got yourselves. Who do you think should be using it?

R: I don’t know. I suppose, really it’s the community pharmacy that’s been involved in making that clinical decision whether the patient needs to have the correct, yeah, whether the patient has got the correct prescription or whether they need to go and see their GP, and I think that’s appropriate. I do think community pharmacists have that level of expertise and knowledge. And I think it’s good that they are being encouraged to, I suppose, question what’s happened in hospital because we know in primary care that sometimes discharges aren’t correct. As I said earlier, they’ll add something on but their patient doesn’t need it or, you know, something may appear to have been discontinued when it shouldn’t be, but I don’t know whether community pharmacy know that. So I think it’s good that...

HP8

I: Yeah, who are the important players, who matters, without whom it wouldn’t work?

R: Well, I think everyone. I think everyone has got a slightly different role. Obviously, the pharmacists that see patients in hospital are usually dealing with something that’s happened and have to make decisions on what the patient needs at that moment in time. But I think it’s important that then the patient is supported by their community pharmacy. And, as I said, the NIPPS service can offer some really in-depth, you know, they’ve got the time to see a patient for 20 minutes, 30 minutes and look at all their medicines in some detail to make sure that they are on the optimal combination. So I think everyone has got an equal role to play.

HP8

R: And I’m not sure that we have that with community even though some of our pharmacists currently work as a split job in community pharmacy. But I certainly haven’t met any community pharmacists since I started, which is bizarre when you think about it.

I: Yeah, there is communication but it’s, sort of, like... And some of the communication may well be, sort of, ad hoc or informal, e-referral is trying to make that more...

R: Standardised, yeah. I don’t know if I should say this because it’s just an impression that I get, but some of the NIPPS team I think are a bit confused about why we’re getting these from community pharmacy and it’s been, you know... I’ve been sending this email saying, oh, can you just check on this patient, here’s all the details that I’ve got. I go, okay, but I don’t understand why, you know, where’s this come from. Even though we’ve told them about the service many times and in the lead up to it and preparing them. And then obviously if I’m on annual leave or I’m sick, we’ve got a system in place for someone else checking the email, so everyone is aware of the system, but they just don’t quite see the value. And I get the impression that that’s because it’s coming from community pharmacy.

HP8

R: Because obviously they are experts in medicine. They have got the time to do long appointment times. So, yeah, I think the fact that there’s initial communication that, sort of, precedes the e-referrals communication, potentially if the GP was aware that the NIPPS pharmacy team could see them it would save them.

HP9

I: So the e-referral work, how does that fit in with the broader work of being a practice pharmacist within the NIPPS service then?

R: Well, it fits in in terms of the chemist…what I’ve come across so far I think, it’s the chemist referring people to us if they have any issues like, they’ll say, can you look at this? This is what I’ve come across so far if they have a problem with the…yeah, if there’s a discharge that they’ve got a problem with like their medications don’t match up with the ones on the repeat then they’ll flag it up to us and they’ll get us to review.

HP9

R: It varies…sometimes it could arrive within the next day and sometimes it could be…depends I guess…if it's a weekend maybe it will take a bit longer. Depends where it's coming from, so from (name) I think it does come quite quickly. I've seen ones that come the next day which I was shocked about 'cause I think they usually take at least a few days.
I: And they come elect…do they come electronically?
R: Electronically, yeah.
I: So you see…what would the normal…who goes through those?
R: I think the admin staff go through them. So in this practice I've set up a system because they used to send a lot of discharges and a lot of letters, so we've set up a system where the admin obviously will get the letters that are, I think into one box and then they would send them over to the doctors and if the doctors think it's complex…the ones that are complex, they'll send them over to me, so they don't send letters, they don't tend to send just letters, they send discharges to me. So this is the system we've got in place, so the doctors might get them now before me and they most of the time send it over to me, especially Venalinks, because they are classed as complex. So they come across to me, yeah.
I: Yeah, 'cause [voices overlap 06:15], yeah.
R: Yeah, so they'll send them over, so…
I: But that's going to be a few days after the patient's left hospital as opposed to…
R: Yeah, 'cause it doesn't come – like I say – it doesn't always come straight away, it's just some instances it does come straight away and the doctor's seen it straight away and sent it over to me. This is an odd instance that I've seen…they've been discharged and then I'd got the discharge the next day which I was a bit surprised about 'cause I'm sure they have a lot of discharges, but you do get those instances.

HP9
I: Yeah. It's just…'cause I think – going round the houses here but it does really matter – I think when things started that…well, before, when things were being planned, it was thoughts of you lot being more involved. And maybe that's not necessarily…that's not happening but it's maybe…it's useful for me to know what the…
R: Yeah, it is different…
I: …the timescales on it.
R: …for different practices, it's different. I know some of my colleagues probably do only meds…the discharges and have loads and loads to do in a day. I don't know how their system works. I think every practice has got its own set system. I don't do any discharges in the other practice because the doctors do them, but in this one – like I say – they'll come across to the doctors or to me. Sometimes it will come to me but usually the GP will see it and then send it across to me. So it might be a few days. I tend to tell them if it is urgent and it's a blister pack and they're going to run out then you need to obviously do them, because I only work three days a week, so I'm not here then. I don't want a patient to get left without medications, yeah…
I: …yeah, because…you know…
R: …they'll run out, yeah. But I think this chemist next door is really on the ball, especially now like, they just flag things up which is really good, so…

HP9
I: Yeah, yeah indeed. Who do you think are the important people in that?
R: The right answer I guess is everybody.
I: That's what everyone is saying.
R: 'Cause everybody needs to do their bit, so the hospital needs to like obviously do the referral and then the chemist needs to act on that referral. And then when they've forward the issues I guess, if it's to me or to the practice staff, then there'll be, there, everyone's got I guess their important and role. Do I have to specify who's the most important?
I: Yeah. Well if you think there's someone most important?
R: I mean, they're all really important. I guess to start that chain you'd need the people at the pharmacist, at the hospital or the technician if they can do it as well, to start the referral process because otherwise the rest of it will not…
I: …not happen.
R: …well, just not happen, yeah. So I guess yeah, I would say the person at the hospital, so it's the pharmacist or the pharmacy tech that does the referral to the chemist.

P4_C4_C5
I: So, did they get sorted, those problems with those medicines?
R2: Well, I phoned and she said, what was it, I'm sorry there's nothing come through. I said, well I'll tell you what, I said, it better be through for Thursday, I said, or my husband's got no tablets.
I: Yeah.
R2: So she said, well just a minute. Oh yeah, they've found it.
I: Yeah.
R2: They've never passed it through. And I know he takes his time for signing them off.
R3: There has been one occasion, where I have been chasing a prescription from morning, that should have gone through to him, and I've waited and waited, to the point where I've gone down to the…I've phoned all morning, oh, he's in clinic now, he'll do it when he's finished clinic.
R1: Yeah.
R3: And I've gone into the surgery, and asked for him. Oh, he's on his lunch. And I've waited in the surgery, and he's walked through and I've gone, is that the doctor? Doctor, I forget his name now, but at the time I went, Dr (name) And he turned round, and he's going, and he's walking, and I went, Dr (name). And he's turned round, and I said, it's my…(name of patient) daughter, I said, have you got his prescription please, I said, only he's desperate for it? And he's looked at the girls and he's gone, oh has it come through yet? I said, yes, it come through at such a time this morning, and I said, he's urgent for it, the girls passed it through to you at such a time today. And he's just walked off, gone in his room and then sent it, I'm sat there, and he's sent it through to the pharmacy, and then the girls have said, oh, he's sent it through to the pharmacy…
R2: Won't come out.
R3: ..cause I was like that, I thought five more minutes, and I'm going to knock on his door, because I think they just…you was brought up with respect, my Mam and Dad always brought me up with respect, and now I have a few friends who are GPs and pharmacists and stuff like that, and they're ordinary people, but some of them get this chip on their shoulder, and you think, no, you treat people with respect. And to me, that GP, he's just taken on too much and can't cope, but he won't pay for the added locums and the extra staff to take on the workload he's got, and he's there for a service not to make money. If he's in that, he's in the wrong job.

4.2.3 Movement of information in the network - How is information exchanged between actors

C2_C3

I: Right. Thinking about the medicines before that hospital, what sort of information was given about the medicines, who talked to you or to your mum about the medicines?

R1: Well, the last time she was in they noticed that the pregabalin, the one for the clot, wasn't working properly, so that's when they put my mum on the injections, so she has injections daily now for those. And then they informed us in the hospital that they'd be giving her injections from then on and she has the injections now instead of the tablet form. But they come round and…the chemist in the hospital pharmacy phoned us up and came to speak to us and went through all the meds and went through them with us and explained that they didn't have the sleeping tablet, the [inaudible 03:36], asked would I bring some down, the packet down while she was in, and then they just prescribed a slightly different one. Exactly the same sort of thing.

I: Right, so they did make some changes.

R1: Yeah, they changed the sleeping tablet, more or less the same sort, just a bit different manufacturer, and the injection she's on. She has injections now and she came out with some antibiotics for infection just to finish off, which she's stopped taking now. But they're pretty good actually at explaining and that. They phoned us up at home.

I: This is the pharmacy at the hospital?

R1: The pharmacy in the hospital, yeah. There's only the doctor really that changes anything when she's at home. The pharmacy just takes word off what they say. The doctor never has to come out when they want to give her something else or knock something down, they do…it all goes through the surgery then.

HP1

I: So, we'll start off really with some of the background to the Pharmacy Referral service. Why do you think it was introduced?

R: So I attended some of the initial meetings, with, I think yourself and (name) So, it's to improve the communication across three, initially, it was the three sectors, community, practice and hospital. And obviously, the prime goal is to reduce hospital admission, via reducing errors and that could be prevented by communication amongst ourselves.

I: Yeah, to join that communication up, yeah?

R: Yeah, so just simple communication between, maybe the community pharmacy and the practice, will then reduce an error, minimise an error and, yeah.

HP1

I: No, no, I think that does, because it's sort of like, you know, basically the more information you're getting therefore, and the faster you're getting it, and if it's directly saying, you know, look at this?

R: Yeah, and also, exactly the faster you get the information, because what my experience was, we kind of just went around in circles, when we didn't really need to. If we were involved from the beginning, we would have just got that information, we didn't have to waste our time finding out where the information's come from, what we need…so it would have just been better, if we just had the information as well, and we just tackled it there and then.

I: As the community pharmacists do?

R: Yeah.

I: Precisely, yeah. And you've got then that triangle as it were.

R: Yeah, and the practice are who's issuing the medication, so surely we should have a prime role in having that information first, because well, if it was a query about this patient's whatever, Ramipril was removed on discharge, but it's still not removed, it probably wasn't removed, 'cause we don't know that it was removed yet, because we haven't looked at that discharge yet, because its [voices overlap 00:13:11].

HP1

I: Yeah, that's really interesting actually, getting some understanding of how that triangle might have worked, is really helpful. Just going back a little bit. So, what you see is this notification by email, can you describe how it's used, what happens in you work with it?

R: Okay, so I hate the notification, 'cause the first time I had it, and I'm quite computer savvy, my mum was a computer…anyway, we used to do everything on computer from the day we were born, so I know how to use computers. But, when I looked at it, I thought, oh God, what the hell is this? So, what it is, is they send you an Excel, I'm trying to open it, but it's not really going to be of benefit, so I can describe it, so they send you an Excel spreadsheet, with…this has actually been, so I've actually changed it so you can see the words, but initially when I got it, there were just codes on the Excel spreadsheet. So, you have to then change the format to work like Word, so text, and then you'd see it. So it's quite simple but someone that doesn't really know, they'd kind of be looking at it for about ten minutes, thinking, what is this, I don't really know what this is? So, I think that's now been fixed, because I raised it with (name), and I think (name) now will change it, before she sends it out. And that was my initial experience. The other thing was, there's no NHS number, so what happens in general practice is, I receive this email via my NHS email, and then I then, there is a column that said, where I eventually found, there is a column that says what is the issue. And so, for example, the first one I got, the issue was medicines stopped in hospital, still on repeat. I don't actually know what the medicines were, that's it.

I: It's not told you which of them, or all of them?

R: Not told me which, yeah, but I just guessed that another reason, going back to having training, it would be nice to actually see how it looks on the hospital side, so we have an idea what that has come from. Was it actually someone free texting it, or was it just a tick box?

I: Yes, a drop tick box.

R: If it was a drop tick box, then it would make me think, okay, it might just be a general thing, it might not even be an issue. So, that just helps with our thoughts. So, I knew that there was an issue, but I didn't actually know, there was only a patient ID. Now, in EMIS, and Vision, you can't get the patient's records via their hospital ID, that's only for the hospital system. So, what I had to do, was log on to Sunrise, now you wouldn't appreciate it if you haven't done it, it is easier now, but some practices still have some block on the computer, so sometimes it's difficult to log onto sunrise from a practice, which is the hospital EPR. So, what I had to do, was log on to sunrise, type in the hospital ID, find the patient, go onto the patient information to find out what the NHS number is, so I've got the NHS number, go back to the practice system, find out who…and this is just to find out who the patient is.

I: Yeah, before you do anything else.

R: So, the first day I got this, I couldn't log onto EPR, which is sods law but they had updated the system at the practice, and I couldn't log on to EPR. So, it was very stressful for me, because I knew that something wasn't right, because we were issuing something on the repeat, but it had

been stopped in hospital. I didn't know whether it had just been stopped, or is this like, has the patient been having it for about two weeks? And I didn't know any other information. I didn't know who the patient was. I didn't know what to do. Didn't know what pharmacy it was, because there's no information about who the pharmacy is, which pharmacy it is, so I had to guess and just called, yeah, there's no information on what pharmacy it's come from. So, I didn't like it. And, I did speak to (name) about it, and said, why isn't the NHS number in there, just so it's fast? And she said, that the way that it's sent via the PharmOutcomes, is they can't generate the NHS number, so it can only be this way for some reason, I don't know. So, yeah, in fact, it had got a pharmacy number, (name of pharmacy), but there's hundreds of (name of pharmacy), pharmacies in (place), so I didn't know which, so I just called the one next door, and luckily it was them. So, that's how it looks like in practice.

HP1
I: And also, then, having to do further things, as you…I mean, do you think, what's the sort of, how's this going to move forward then, in terms of, is this going to stay the same like this, or is this going to be, do you think this'll be…?
R: I mean, I think that there's a lot of things that don't need to be in this report that we get sent. I mean, this is assuming that we're not going to be in the e-referral system, and it's going to be a two-way, if it was a three-way, it would be a different story, 'cause we could actually see the system, and yeah, but currently, as it stands, we're just getting these reports as and when we need to. And the reports are really, there's information that we don't really need, like DISWA B3, ah, that's probably Discharge Ward.
I: Discharge Ward, yeah.
R: I mean, yeah, that's useful, because if we wanted to call the ward, and we know where they got discharged, yeah. But there are things that, I think there's information that we don't really need, and the information that we do need, probably needs highlighting more. We also definitely need an NHS number, or an easier way to get to know who the patient is, because not everyone can access EPR unfortunately, which I think they should be able to, but even if they can access it, it takes time. And the way that we access EPR in general practice, we have to log on via … and then we have to go on to our hospital system, and then we have to select Sunrise, and then we have to log onto Sunrise, so already there's three log ons, yeah, just to get onto the system, so it's not really that easy to do, yeah, and it takes time.

HP1
I: Yeah, precisely, yeah. So, some limited information coming through, information is difficult to unpick, unravel, difficult to act upon. How is that then fitting into the rest of what you're doing, in terms of your work? Is it slowing the rest of your work down, is it changing the way you work, or what?
R: I mean, the numbers that we're receiving, it's not really changing the way that I work, it's just something, as and when I get a notification, I just need to deal with it. I think that is part of my job role, so I don't think it's affecting me. I'm quite chatty and just speak to anyone, and anything anyway, but some pharmacists, some of our inexperienced band 7s, might be less chatty, so I think it's good, in that it promotes them to pick up the phone and call their local pharmacy and say, oh hi, I'm the pharmacist, what's happened, I've just received this notification? Which is the whole point in the whole three way communication, so I think it is good to have these every now and then. It's just, if it was a little bit more slick, it would be nicer.
I: Yeah. So, the information you're getting through is the issue, or how it's presented to you.
R: Yeah, how it's presented, yeah. And whether it needed to be presented, because if it has been dealt with, or if it was something that, one occasion as well, because the pharmacy, and again I raised this with (name), the community pharmacy get it after 72 hours, and we receive a discharge later, maybe within the week, so they might have sent us something, and by the time we've looked at it, we probably are coming to it, so sometimes it's a bit of, I feel, it maybe was just unnecessary, maybe just highlight the things that needed doing asap.

HP1
So, a lot of pharmacists would be in the position anyway, it wouldn't be an unfamiliar system to…presumably. So, if you had that three, if you were seeing as the discharge or e-referral went to the community pharmacist, if it came to the practice pharmacist at the same time, how do you think that would be of benefit then?
R: I mean, it depends on what needs changing. If it was something to change on a repeat, we'd just do it there and then. I think also, the issue is that the community pharmacists, there's been two occasions where they've said something, and actually it didn't need reporting, because it would have been removed anyway. So, if it was a, they had a repeat, and they said, it's still on the repeat, it was still on the repeat because that's an old prescription, an old repeat. So, sometimes I think that the selection of boxes, they also need to understand what that would look like for us, so I think the importance of the community pharmacy selecting the right boxes, and the hospital pharmacy selecting the right boxes, because if they select the wrong box, it just means something different to us. So, for example, I'll try to make sense of what I've just said, if it was about, like if we take this example, so, medicines stopped in hospital, still on repeat. This is what the pharmacy had told us. And again, it's difficult, 'cause I haven't seen the system, I don't even know how they do it. But, do they know that that is actually the warning for us, the issue that's been highlighted for us to action, or are they just ticking this, assuming that it's just information, just saying that the hospital's stopped this, and it's on repeat, but it's fine, it's nothing we want anyone to do anything about it, we're just stating a fact. But I don't think that they realise that that, to us, is, okay, it's stopped in hospital, but it's still on repeat, there's a problem, why is it still on repeat? But then going back to the community pharmacy, when I called them, they said, oh it's fine, it's just that it had been stopped, but I don't think they realised that that was something that's been highlighted…

HP10.
I: There's more quality information?
R: Yeah, yeah, where before it was handwritten very often and sent via the fax, it got even worse in terms of quality, you couldn't really read properly what that information was. I've got a feeling this digital we get now and it's even more...there's more in it, there's more information. I don't think it's the same as the doctors get, I think they get an even bigger one, but we, for example, now can find out about what the patient was admitted with. I always scan through it, I always find time to, and I read, oh, this and that and the other, I read the little history. I'm not an expert, I'm not a GP, I'm not a doctor, so I don't... I mean, I understand most of it but I don't understand everything, but it's helpful to know that, say... I'll give an example. If someone was...it's happening quite often, I think 20, even 30, per cent of my patients will be admitted due to falls, they'll be falling, they fell in the house, they hurt themselves, taken to hospital. And I'm thinking, oh God, looking at what are they on. Oh, they take alendronic, they take this, this and this, and I'm thinking, Christ, how did I not pick this up earlier? Oh gosh, so many strong painkillers, bloody hell. Maybe we should be more conscious about it. You've got to get a better and bigger picture. You can understand this patient, oh, hold on a minute, oh Christ, I should maybe make sure that she... Doctors often don't think about those things, they will issue another painkiller that could make them drowsy, and I'm thinking, hold on a second, she was admitted to hospital with a fall not so long ago. Just think about that. Not so long ago, where a lady was on very strong pain relief and their doctor must have given...went for a home visit. So I just picked it up thinking, oh, Christ, she's

given her this. Oh, no, no, she shouldn't be on it, she's already on this, she had this fall. And I just rang: listen, this and this happened not so long ago. Oh, yeah, yeah, thanks (name) I will change it, so that was good.

HP10

R: Yes, you're right. You're right, yes, it does. I'm not sure whether you are trying to refer to the fact that I've just said about communication with the patient.

I: Yeah.

R: That's what you meant. Yeah, that's right. True, I didn't think about that initially, it just came out in the conversation. Yeah, you're right. It's kind of like you're looking at it, when you're dealing with that on PharmaOutcomes it says, what have you done? Commenced a blister pack, phone them post discharge, MUR, NMS, this, that and the other. So you're thinking, oh, maybe I should do that, you know, like even that or just... It's just basic communication and those people often, you know, they don't... You're not trying to patronise them in any way, but where I work, there are a lot of working class people, so they're just people that don't necessarily understand things very well but they appreciate, like, you know, like you look after them, you tell them, you... To be honest with you, if you ring them, give them a ring and reassure them, you save yourself phone calls later, oh, where's this, where's that? It's just you do it and you know everybody is happy, it's much better. And again, you know, as I said to you, maybe it's me, but I always enjoy doing clinical stuff, I always like to kind of have that broader picture overall. Communication makes them safe and makes the drugs they use safer, in my view.

HP11

R: So I assume, well they said that is the reason. So also, you get the whole information, which is so much better than just the medicines discharge. At one point, we had…the surgery would give us… If we didn't get the sheet from the hospital, 'cause that was always a bit hit and miss as well, and the surgery would only give us the sheet with the medicines on and then they wanted to cut the name off the top, which is not very…it's just not safe, is it?

I: Yeah.

R: So this is, we have all the information that the GPs have. And then we can then check. Because obviously, they have that information but then they're using that to generate scripts. But if we can also check properly as well, it's just safer, isn't it?

HP11

I: Yeah. So is it that information that's empowering you to do?

R: Well, I think it will, yeah. Well, I'm…I don't think it is yet, because I don't think it particularly applies to those Venalink patients.

I: Right.

R: But…

I: Because they don't come in, obviously.

R: Because they don't come in, but it is good actually to look at that and I do always try to make a point of reading this is what's happened to this patient. This is why they ended up in hospital, which we never used to…we didn't always know that. So it is…

I: Yeah, precisely.

R: Yeah, so that is extra information, which is useful…

I: Yeah, particularly since…

R: …from a pharmacist's point of view.

I: …yeah, and also because by nature of the fact they're on a Venalink, they're going to have multiple drugs.

R: Yeah, I mean you might see somebody's relative who comes in and will…they'll tell you that, but if we already know that then that's useful, isn't it?

HP11

R: Yeah, I don't think I…yeah, I think it'd probably just progress like…but I think (name)'s planning to do that anyway. I think that's…yeah. Yeah, I think for what we can do now with the services we have, yeah, I think we're getting all the information we need.

I: Yeah. So do you think it's…I mean, broadly, do you think it's a positive thing?

R: Oh, definitely. Yeah, in every way, yeah. I can't see that it could possibly be a negative thing, can it?

I: What are the main things that make it a positive thing then, I wonder?

R: I think there's the security, the extra information, the speed. Just communication really, it's just like, knowing more is always a positive thing, I think.

HP12

I: Yeah. Has the service changed the way…I mean really what I'm looking for here is if you can tell me how the service fits in with what you are doing at the moment or what you were doing in your existing work. So, has it changed the way you work or impacted the way you work?

R: It's impacted on the way we work in the fact that we get the information much quicker and everything is done in less of a rush.

I: Yeah.

R: Which I think is quite a safety issue. So, we've got a lot more time to sort everything out. We've got time to sort the prescriptions with the surgery; they've got time to look at it properly, to work out everything. So, that's worked much better. And we're not bothering the surgery saying; please can you print me out a discharge summary.

I: Yeah, because you've already got it.

R: It sounds awful to say but if the surgery just do us a new lot of prescriptions, we prefer to check that against the discharge summary, just to make sure that everything is as it is on the discharge summary. I know the doctors have issued the prescription but we prefer, as a double check, to check it against the discharge summary. So, sometimes they were getting a bit…why don't you get one and blah blah…so, we've got that information and like I say, it's the other way round now, we are taking it through to them saying, this patient has been discharged, here is the discharge summary, please can we have prescriptions.

HP12

I: Yeah. What about the discharge summaries, how useful are they then.

R: Really useful because you've got all the information there about the medication. And it's very straightforward how it's presented and it's easy to compare that against our control charts that we use.

I: So, before you just got a list and now you get everything about that patient.

R: Yeah. So, obviously we get the list of medications which is easy to check against ours but there is also a section where it says, what's stopped, what's started, I don't know whether to say it's interesting or nosey to know why they were admitted, but sometimes it might be something that we can prevent happening in the future, so I don't know, if they have gone in with acute kidney injury because they've had sickness and diarrhoea and they've carried on with all the medications, or anything like that really; or they've taken too many of something and they've ended up in.
I: Yeah. So, there's been a medication related admission.
R: That's handy for us to know, just to keep an eye on in the future really.

HP12
I: And having that extra information on the discharge summary, how useful is that around those conversations?
R: To be fair, probably the ones that we see personally and have the conversations with are probably not the ones that have gone in with medication related issues. So, probably not so much.
I: Yeah. But is it useful when you are having that conversation with someone to be able to say, oh I see you went in for whatever?
R: Yeah, definitely or I'm trying to think what we can say, you know, you'd had a fall, are you alright now, is there anything you need help with, is there…you know, it's just conversation openers sometimes, rather than the actually doing anything about what's happened, but it's just that bit…because some patients tell you absolutely everything about themselves, and some patients tell you absolutely nothing. But what we are getting is the bare bones of what's happened and they can either elaborate on it or not, as they wish.
I: Yeah. And that can help you to help them as it were?
R: Yeah.

HP12
I: Do you think the service has, actually or potentially benefitted your work then?
R: Yeah, definitely. I know there has been a couple of occasions previously where we've had discharge prescriptions from the surgery, we haven't actually seen the discharge summary, we've just been told, right this patient is out of hospital this is the new scripts, done them, sent them out to the patient and the patient says, I've not got everything here, I was on a lot more medication and then you'll look at the discharge summary and half of the stuff hasn't been done for some reason. Whereas now there is that extra check because we are seeing the discharge summary, the doctor is seeing the discharge summary, the patient and family, depending on how able they are to deal with it. But they can look at it and say, I usually have eight things in a morning, I've only got three, what's going on, sort of thing. They might not know what they are but they know that it doesn't look right. But now we've got that extra step in that we can check that what's happening is right.

HP12
I: So, the improvement in relationship is because you are not rushed. You aren't rushed because?
R: I think because we are initiating the request rather than waiting for the information to be sent to us. Because we've got the information, we can start acting on it straight away. Whereas I think at the doctor's surgery there is that much to be going through, that it takes a few days before that information would necessarily be acted on.

HP12
I: Yes, but you are still empowered with that information to have that…
R: Yeah, because we got the initial information, we spoke to them, sent them the information so he's then rung and said, I've dealt with it all but I've not done this because of…you know.
I: Yeah, precisely. Is there any communication, apart from the little notification you can put in there as to why you are accepting or rejecting, is there any communication back and forward to the hospital?
R: Not an awful lot. We do find if we are ringing up now it's much easier to say, I've had a discharge summary from such a person, and this is…we did have some referrals the other day, so they were discharge referrals, no they weren't they were inpatient referrals, but they were patients we didn't do a blister pack for. So, we sent it back saying, we rejected it, we don't do a blister pack for this patient, and then somebody from the hospital rang us and said, they had been assessed, we feel they need a blister pack that's why we've referred them to you.
I: Okay.
R: And then when I did look at it more closely it did sort of say, referred for new compliance aid. So, it's all still a learning process. We kind of, just looked at the name and went, no we don't do her, reject, you've picked the wrong pharmacy kind of thing.
I: Yeah.
R: So, it's all still learning and the fact that we'd had that phone call, now makes me think, right when we get one that I don't know, I'll check that it doesn't say.

HP12
I: Yeah, that it's a new one. Do you think the service is improving care or has the potential to improve care?
R: Yeah, definitely.
I: In what ways?
R: From a safety point of view that we are knowing that they are getting what they should be getting on the discharge summary. From a safety point of view in terms of not delivering stuff when the patient is in hospital, from the fact that there's hopefully no missed days of medication because we didn't know they were out of hospital, we didn't know there were changes, you know, so hopefully there is no missed days of medication. So, yeah, I think it's definitely improving.

HP13
I: You could carry on supplying and not know.
R: Yeah, just, yeah, [inaudible 02:32], then I can inform the GP as well. The GPs…I think they're so busy that they didn't always pick up on the discharge or act on them, whereas I think we are doing that and we, kind of, did anyway. You know, it used to be that I would phone the GP once the hospital had phoned me to make sure that the prescription was coming and that they knew about about the changes. Half the time they hadn't read them, they hadn't got round to reading them, you know.
I: Yes, and having knowledge now that the patient is in, is still admitted, what's the value of that?
R: Well, that means it saves time because if we're just preparing a dosette for that person and the thing's gone orange and I realise and I look at it because I was getting a little message 'til we found out that person's gone to hospital, we don't prepare the dosette, there's no point

because they're in hospital. And then if their medication does change, then you've saved a waste of time, haven't you, and a waste of drugs, a waste of all kinds of things because we just write a note now to them saying the patient appears to be an in-patient as of what date and we'll leave it until we get the discharge, it's marvellous really.

HP13

R: Yeah, go through it. I like the PharmOutcomes thing because this actually makes you go through it in a systematic way. See, because when you've got the discharge now, you have to actually say what you've done and tick boxes, you see, which is marvellous because before, I did it but I wasn't acknowledging to myself or anyone else that I did it.You know and it's saying, the first question is have you reconciled all the medicines and that's the first thing you actually do which we always did but now it's making you do it, it's making you declare that you did it and it's asking you have you got a repeat prescription, which is good. You have to look and see have you and then they ask you is it correct. And then they ask you have you had to refer anything back to the GP which again, if I do, which I often do, then I have to obviously press a yes.

I: Is that all within PharmOutcomes?

R: Yeah, it's all in one page, it's excellent and there's notes to say…you can put what exactly you had to refer to the GP and why you had to and what the GPs done about it. This is obviously marvellous.

HP13

R: It's better than, yes, it's better than, yeah. It's not only that, I've forgot about the actual patient, which is important. I'm involving the patients a lot more than I used to.

I: In what ways?

R: Because I know I've got the discharge, I will then…I've got it early, I've now…because it's the PharmOutcomes, you have to tick things that you've done. One of the things I will do now which I wouldn't have actually done necessarily, I will phone the patient, I'll let them know that we know they've been in hospital, we've got a discharge, they're not to worry if they will be coming later in the week and blah de blah, you know, if it's the same I tell them. And then also when I go to the patient with it or whoever goes, it's me, usually, if they've been in hospital I will go and if there are changes I do a medicine review with them all about the changes to let them know.

I: In their home?

R: Yeah, in their home, yeah, what it is about and I would not have done that before. I didn't take it as…just because PharmOutcomes is very well designed 'cause it draws your attention to the fact that that's what you should be doing, so that's what I do. I've found that that's helpful, very helpful and I think the patients find it helpful, they've all been very grateful because maybe they haven't understood what the doctor had said to them, maybe they didn't know that their medication was changing or they weren't listening.

I: That new medicine or counselling bit that you're doing there, is that new or is that something….?

R: No, I really didn't do that much before. Now and again I would've done but not with every discharge at all whereas now I am doing, if it's changed. If it's not changed, I just phone them and say there are no changes, we'll be along with your medicines on Thursday as usual, end of story. But if it's changed, it's making me do things I would not have done.

I: That's fascinating, actually.

R: Whether everyone is, I don't know but I feel that it's definitely made a big impact that way.

HP13

I: I was going to say is it the system itself, you said that check…be able to having those checks through it or is it the fact that you've got that information itself that drives that?

R: It's both those things; it's having more information and also the PharmOutcomes, making you, as I say, go through it in a logical way and making you realise what should actually and what could be done.

I: It's the steps of what needs to be done, yeah.

R: I think it's those two things really, yeah.

HP13

I: And is there a cost saving on waste or stuff like that involved as well?

R: Well, there would be because in the past if the GP did the wrong prescription, you'd take it to them and then you might still have had to get, the patient wouldn't have had to be using that one. You'd have to go along a day later with the correct thing 'cause somebody realised somewhere, you know. Yeah, there would be a saving and also the fact that because of the quick notification, it saves us making a pack up. So that prescription can just be returned to the [inaudible 28:14] or something, it can be… It means you're saving, you're not even having to dispense that prescription. And if it's incorrect, you just destroy it, it's not a waste at all.

HP15

R: But at least we can start our process and check with the GP surgery the next day. Well usually the day, because we are not always sure when they receive the discharge letter either. So, we leave a day and then we check with the surgery if they've received the discharge letter, if there are any changes. And then we request the medication accordingly. According to the supply that they have had. Yeah. But we just then check with the current medication if there are any changes when we receive it. And then after two weeks, if there are any more changes, again we complete the online request and say, yeah, everything is fine and we have received the request. We have checked with the patient, their MUR or whatever needs doing

HP15

I: Just to confirm with you that they are right. That's good. There's another part as well. We talked there just about the extra communication with the patients. And we talked a bit earlier before about that communicating extra…you know, your communication with the GP practices and so on. In what ways has this improved, you know, communication with the hospital? Or has it improved in communication with the hospital? Has it not?

R: It has improved. So, they don't necessarily need to call us and tell us if there are not any major changes. We just read and…yeah. Unless there's something very confusing or something then, we will have to call them, but we've never had any such issues, once it started, yeah.

I: Yeah. Were there before? Were there times when the hospital were calling you or you always check back with the hospital about a patient or something?

R: There were a couple, yeah. So, what would happen sometimes, is the patient would say, oh I've been started on this one or my dose has been increased. But maybe a page has been missed out in the discharge they've sent to us. So, we have not seen that, yeah. So, we have to check

with the GP surgery if they received a full discharge and was it in their discharge. So yeah, there was a lot of time that was used up in that chasing up to see what's happening. Or we ask the patient if they have a copy with them, they can bring it in and we can have a look. So, if a discharge has not been sent properly, we can at least use what they've been given by the hospital, and tell the doctors that we have seen the paper from here, and we take a copy and send it to them. Yeah. But that's reduced a lot now.

HP16

R: It makes things easier for us, to be honest with you, now I'm a very big data person so I love having information, I'd love to be able to see the GP record, I'd love to be able to make our contacts count even more because we have more information to deal with at our disposal. So that would be great for us because that impacts on clinical care but actually trying to get hold of a fax, trying to phone a GP about, have you got a discharge, can you send it over, what's changed, what hasn't, that whole workflow for us is reduced because I've got a copy of the discharge there to utilise. So that means again our conversations are more technical, they're more about the clinical, the issues rather than actually trying to do, in essence, admin work, trying to just get the information so that we can try and do something with it.

I: Yeah, precisely, so before it's phoning the GP, oh, what's happening with this patient, what's going on, now it's I've got it...

R: That's it, what's going on, have you got a copy of discharge?

I: ...so what can we do about this?

R: Yes, that's it, and that's a much more meaningful conversation not only for the practice, for us but for the patient also, they may not necessarily see that conversation happening either way but for us that means our time is spent more efficiently.

HP16

R: To be honest with you, I think we would welcome it because what we wouldn't want to see is actually somebody's gone in and they've got codeine and paracetamol because they've got a cut on their leg. Something very routine like that, we don't need to see...

I: But they're not likely to have a nominated pharmacy, well, they might, they may do, yes.

R: They may do, you see, that's it, they may do, for example, if you have a nominated pharmacy, you fell, you graze yourself, you want to get it dressed and cleaned and they gave you some paracetamol is that relevant to me?

I: No, it's not.

R: Probably not.

I: So it's about relevance?

R: It is, it's about relevance so actually if there's anything that could impact, so if it's somebody who's on four or more medicines but they've gone in with something, they could be in with, I don't know, a UTI, for example, but actually the link could be, oh, I've seen them three times with thrush or they've come in twice for thrush treatment recently. That's the beauty of it, that's the continuing care impact because they choose to come to us to get their OTC medicines as well as their regular medicines.

HP16

I: And presumably you are in this area, you're fairly central for the people around here in (name of place) this is where they come.

R: We are central, we are and we have a massive, for example, Jewish population who don't like going to the doctors, who don't like their ailments being sort of like aired, they want the confidentiality, they want to know about kosher status of medicines, we can offer them all those advice so they know they can come here, get the high quality advice that's relevant to them. If a Muslim person comes in we advise about alcohol content, we'll advise about gelatine status, all those things we will check because we understand that's an impact on somebody's values.

I: Yes, precisely.

R: And that's why people come back. So there are scenarios, yes, where people will go here, there and everywhere to buy their paracetamol and whatever it is but we do have a cohort of patients where...and even if it's just one patient who we can make that impact for to stop a readmission, to make a difference, that's what we're here for.

I: That just improves their knowledge.

R: That's it, but the actual...there's an intangible and tangible benefit to the system from that, not only the patient, the GP and everybody in it but the overall system.

HP16

I: Do you think it would be better if it was more of a triangle…?

R: I think it would be better because what we would do is we would see inter-referrals, so actually what we want to see is the discharge MRU come to us but then the practice pharmacist could see I've done a discharge MRU and a quick bit of note on it, these are the couple of issues that have come up, can you deal with those, please? And vice versa, they could say, actually I've just started somebody on an inhaler, can you MNS them for me? And the same way the GP (inaudible) goes why can't they refer to us?

I: Yes, so there would be a referring back and forward between?

R: Yes, but it would be one...[…] One joined up approach so that if that patient then went back in the hospital pharmacist could see, oh, there've been some conversations here, so I can see the community pharmacist talked about your inhalers, I can see, oh, the practice pharmacist has started something new and asked the community pharmacist to do this as well, I can see that's happened. Brilliant, so you're really good on your inhalers so actually do I need to counsel you about your inhalers or do I need to counsel you about allergen avoidance or do I need to counsel you about something else which is causing you to have that problem? Do we need to think about changing the inhalers because obviously your inhaler's already optimised? So it's a better conversation for the patient, for the pharmacist, it's more valuable but actually, again, for the overall outcome.

HP17

I: Yeah, we were saying about communication with the GPs. Has it…having all this information has it improved the communication with the GPs, or has it made it more difficult, has it made it…or is it the same, or what? What changes have happened there, if any?

R: So, what I would say is it's reduced my stress to the point that you're not stressing about getting the information, so you're not mithering the GP as much. So, we're not…I wouldn't say wasting the GP's time but they could be doing more important things, dealing with their patients rather than us asking them to send this email. Fax machines are being phased out. Previously we would get stuff faxed or picking up the physical paper. So, there's less mither for them. So, we're not…it frees up their time because we're not on the phone chasing discharge sheets. It improves the data transfer. So, you know, it's…it just streamlines the whole thing really, it simplifies things. We've got the same data that they've got. We're protecting the GP in a way because we now have the full information rather than bits of it. So, we can get…because we're getting this full picture you're not missing stuff. So, you can look at the full picture of the patient, look…think in your head as a pharmacist, does it look right, is

it okay, is there any risk here, are there any clinical issues to discuss, do I need to query this with the doctor? Whereas before we might get no information at all. So, overall there's less risk to the patient, less risk to the doctor of prescribing something wrong, or, say…no, say he does prescribe something wrong, or miss something, which is exactly what happened a few days ago.

HP17
R: So, how many patients have I personally prevented from dying, being re-hospitalised by GP prescribing errors before versus after the new electronic notification system? So, I mean I'm getting old now, I've got some [inaudible 00:00:28]…I don't need to tell you that, but I've been doing it 15/16 years but… Yeah, so getting the full information is great, it reduces prescribing errors.
I: Because you have more information to check for the GP?
R: Yeah. So, something might have been stopped but because the GP's maybe not noticed or was rushing and he's stressed in his job, or her job, and they've not noticed that this has been stopped, or that's been started. So, you could get multiple…that one that I just told you about was two diuretics together. You could get all sorts of different changes, it could be anything, and you could get something stopped that the GP's still continuing. So, anything I notice that doesn't match the discharge sheet I'll query. But my dispenser here, when I came, she was a bit reluctant to query and go against the GP and say, well why's this on, or, why's that not on, because once the GP went mad at her and that then changed her attitude. So, he said…this GP said, 'why are you questioning me, I'm the GP, I prescribe this?' Well it's not on there.

HP17
I: Yeah. So, you…it sounds actually that the…you having the extra information does mean you've got more…you're more empowered when you're talking to those GP…the GP?
R: Yeah. So, you're questioning, well I've got this information that says they're on this medication, or, they're not on this medication, why have you done it, or, why have you not done it, or, where is it, you know? And then you've got…and your answer is, well it's on the discharge sheet, can you pass this as a query to the GP? Okay, the receptionist says, I'll send the GP an email and we'll get back to you, or the GP will phone you later in the day, or…

HP17
No, that's interesting and that sort of like leads onwards to sort of other questions I've been asking people, which is around sort of like what…where you see the benefits of this, you know, and there're various strands we can talk about there, one of which is obviously what's the benefit to patients?
R: So, based on the patient the other day the benefit to that patient is that they're still alive, they've not been…
I: This is the one that had the two diuretics?
R: The one…the two…double diuretic dose. So, one, they're still alive, two, they've not been readmitted to hospital, three, they've not taken a double dose of a diuretic, or it could be all sorts of queries over the years, it depends how far back you go. If you're just talking about now we've got the new system in place, or the worst…
I: Yeah, with the new system.
R: …case scenario incidents that I've seen in my history.
I: Yeah. No, now with the new system what…how much is that…the new system, how much of that…
R: Reduced.
I: …is helping, you know, yeah…
R: So, you…I'd say constantly.
I: …patient impact?
R: So, you've got the one from two days ago, two or three days ago. That patient's avoided a major incident by me having the discharge sheet and by me reading the discharge sheet in detail.
I: Is that…that's the one with the two diuretics?
R: Yeah, that's what I'm talking about, yeah.

HP17
R: Yeah. So, I disagree. The more information the better. The more information we have then we can reduce the risk to the patients. Yes, you've got to log on to PharmOutcomes, yes, you've got to spend how many minutes accepting it and printing it out, but overall you've got the information. So, the time that you're spending downloading the information, printing it out, yes, it takes a few minutes, maybe five minutes, but that time you spend is valuable time which will then benefit you because of the knowledge that you've gained.
I: About that patient?
R: About the patient and the time that it's saving so that you're not in a situation where somebody just walks in and says they've got no medication because you've already pre-empted that because you've had this all planned out since the day they got discharged.

HP2
Right. So, when a patient comes in, we will go through a medicines reconciliation. So, we will go through all their meds, what they have and we'll do the e-referral. On discharge, if a patient is going home, the nursing staff or the doctor will let us know. If they need anything…because a lot of the time they will bring medicines in with them, so, we don't have to re-prescribe and we don't have to send them home. But it's if they need antibiotics or pain relief for anything new. And then once that discharge summary is done, then that should go automatically to the chemist via e-referrals, by PharmOutcomes. Yeah.
I: So, in terms of, you know, what you then do, you do the meds rec in the beginning…
R: Yeah. We do the meds rec…
I: But then you do, any changes.
R: Yes. So, if there are any changes throughout their hospital stay, when the doctor writes the prescription or electronically writes a prescription, that will then be clinically checked by a pharmacist. So, they'll check that there's nothing wrong with it. If there is anything wrong, they will get them to change it straightaway. They should then do the changes electronically and then that can go to the chemist. And one is given to the patient, the chemist and the GP, I think. Yeah, all three. So, they all have a discharge summary.
I: Yeah. I think most of the information goes to the community pharmacist. Do you think it should be going to the GP there as well?
R: Definitely. Yeah.
I: Yeah. That's really interesting. Yeah.

R:	Definitely. Because on the occasion…some occasions I'll ring the community pharmacy and they've not had…they have changed something, but it's not on the patient's SIR record. So, there's a discrepancy…why does he not know that but they know the changes. So, I think the GP definitely should.

HP2
So, yeah, that's really, really interesting. In terms of you, what the consequences of the e-referral service might be, let's look at that in a few different ways. Firstly, do you think it, you know, has actual value or benefit for…you know, you talked there it might have some negative effects to the pharmacy. Has it benefited things here in the hospital?
R:	The pharmacy department, yes, because of the time it saves us. I think it will benefit a patient if it carries on and it's up and running, because if the chemists and GPs surgery get together…if this patient is coming in and out, in and out all the time, they can look into that, you know, get that patient referred back to the doctors surgery and say, why is this happening? You know is there is somewhere that this patient…are we failing the patient somewhere where the medications are? Is it a medication issue or is it a social issue? If it's medication we can sort that out.
I:	Yes. Precisely. And is it because then that information is being shared or….?
R:	It's been shared. Yeah. It's being shared by everybody. So, we all know about this patient when they are discharged.

HP2
I:	Yeah. Yeah. Absolutely. Do you think therefore there is an impact…a potential for the service to improve care? Or in what ways can the service improve care?
R:	Well, it has got to be in primary care, once it has left here, I think. And I don't know how it can, but I can't…I don't know how it can, unless other multidisciplinary teams get involved, are made aware. I don't know if that will help.

HP2
I:	Yeah. Precisely. And if you were to make any changes to the service, what would you do?
R:	I don't know. I don't know if this…I don't know if there is a facility to send a message to the chemist when we are doing a referral, when we're letting them know they are in. You know, maybe…no, I don't know.
I:	Are there? Yeah. Maybe. I don't know.
R:	So, if there is…which I've not even looked. I suppose if there was…and I will check when I go back, we could, you know, mention to the chemist, the patient didn't bring any medication in. Said, that they don't have any at home. Can you confirm or whatever?
I:	Yes. Yes. So, on admission you could ask for some feedback from the community pharmacist. Yeah.
R:	From the chemist. Yeah. From the community pharmacy. Whereas at the moment, you're speaking to a patient, who is ill and so, you know, you might be asking them and they might not know. And in that moment in time, don't want to say. So, it's not the correct time really.
I:	Yeah. Yeah. And it's obviously a difficult one, isn't it? Yeah
R:	If there is a communication where you can ask a question or send written information to the chemist, rather than phoning them up. But we always phone them anyway. So, we do the referral but then when it's a patient on a Venalink, then we have to phone the chemist, just to confirm that there aren't any changes or…to the one that the patient had brought in or the one that they've most recently supplied.
I:	Right. Because the patient might not have brought in the most recent one

HP3
I:	And who are the important people in the using of the service? I mean, as well as thinking more broadly, who are the important...?
R:	As in the hospital?
I:	Yeah, in hospital or in the community or whatever.
R:	I'd say it would be us, because when patients come in, we're the ones that would have to identify that they're on a blister pack and we would send the referral out to the pharmacy, it wouldn't be the community pharmacy letting us know that. So it would only be one way, I guess, the referral, yeah.
I:	Yeah, so it's not... That's quite interesting, in so much as that this is purely you sending the information to the community pharmacy. Do you ever get anything coming back from them?
R:	No.
I:	Have you ever seen anything coming back from them?
R:	No.
I:	So it's only...
R:	A one-way.
I:	It's a one-way street.
R:	Yeah. And so I guess if they did have a concern...
I:	They'd phone up.
R:	...they'd have to, yeah. So if there was something in the discharge summary that pharmacies aren't sure about, they will just ring us, pretty much, and there isn't anything that they can send through. But, to be fair, if they did send something through, the patient would still have to be here, they'd have to be reviewed, because I guess there isn't a system in place to see patients that have been discharged if the community pharmacy has an issue with it. Because when the patients are discharged, we move on to the next patients that are currently here. So I guess it should only work as a one-way system, to be honest, and then they would ring us if they had any queries.

HP3
R:	I didn't really have any expectations, to be honest. I think because I didn't really fully understand... I didn't understand what was going to happen. All I knew was...because (name) said it will just make things a lot more efficient. I remember at one of the meetings that I went to some of the other pharmacists weren't really happy about the whole e-referral situation. I think for them... But then I think it was mainly the senior pharmacists that weren't happy about it. I'm not sure if it's because they're so used to the system that we had before, but I know one of them said it's because the pharmacies will now be aware of why the patient's in and what's happened and it's too much information. But I feel like the more information the better, to be honest.
I:	Why do you think that?
R:	I feel like it's just better for them to understand why this patient's been in. If they're left in the dark, it will just be... I don't know what the word is. I think them having more information will help them with their work as much as it helps with us knowing fully what's happening in the community for the patient. So it will help them understand, okay, this patient obviously hasn't been adherent with their medication and maybe we

can do something better to help them be more compliant, I guess. Because, say, it's a case of their blood sugars are ridiculously high because they haven't been taking any of their medication, if the pharmacy's not aware of that they're just going to keep doing the same thing because they think nothing's wrong.
I: Yes, precisely.
R: Or they could, I guess, maybe have more consultations with the patient, make them understand this is why it's important to take your medication. I think it's just a much better handover and transfer of care for the pharmacy to be aware of what's going on in secondary care.

HP4
I: So that communication, how can that help with that safety or how is that going to help…?
R: Because inevitably I see it all the time when patients are discharged from hospital, we see so many problems with mistakes, errors, prescribing errors, when patients go from one setting to another, when they go from primary to secondary, secondary to primary, you're constantly facing that battle of mistakes in that transition, and the aim really is to make sure it's communicated reliably. So if the chemist are getting an electronic discharge of like a snapshot of exactly what they were discharged with, that takes out that kind of element of, you know, they've got the information there so they can then hopefully act on that quickly, much more quickly than relying on a fax two days, three days later where there used to be quite a significant delay, things can get changed quicker, so if it's blister packs they can get their new blister pack up and running, they can nag the GP to get the new prescriptions through. So hopefully the next time the blister pack comes out, it will have the correct medication in, not medication from like preadmission.

HP4
R: Are continued, yeah, that's right. The only…I guess the only downfall slightly is that our system doesn't necessarily show that it's had a pharmacist clinical check. So if a patient's discharged but the nurses or the doctors don't tell pharmacy about the discharge summary, that discharge will automatically still ping off to the community pharmacy, but it might not have had a clinical check, so there might still be errors on that. That's one of the big risks I think in our kind of system…
I: Because unless they make any changes, they don't need to involve you, is that what…?
R: So we try to encourage doctors and nurses to inform us of all discharges basically so we can cast our eye over it, because a lot of the time you do see doctors writing, no changes to medicines, or… We did a recent audit on EAU where we found we changed nine out of ten prescriptions in terms of documentation and prescribing. So if you imagine that and if you imagine that say out of hours overnight if pharmacy aren't open or if a nurse just says, oh, there's no changes, I'm not going to bother telling pharmacy, we'll just discharge the patient, and then that automatically goes off and those errors are continued.
I: Errors can carry on, yeah.
R: That is still a risk, but it's difficult to get around that, so other than encouraging staff to inform pharmacy of all discharges, problem is pharmacy aren't there 24/7, at the moment we just have 9:00-5:00 really, 9:00-7:00 on EAU, so that is still a risk. So the problem with this e-referral I guess is that you can't guarantee it's necessarily going to be correct. If a pharmacist has been involved, you'd hope it was correct, but you don't necessarily… So that's why we annotate I guess if we've seen it or not, if we've clinically checked it, it puts our electronic stamp on it to say, we have clinically checked this. If it doesn't have that and there's errors, I guess the community pharmacy could then ring and say, is this right, has anyone actually seen it from pharmacy, is it…
I: Because you do the order at admission, if you then don't get involved in the discharge, it goes without…[…]
R It really is, yeah, so there is still an element of communication and unreliability to some extent. It's just better than it was.

HP5
I: Right, okay. And what do you know about the pharmacy e-referral service that's been introduced here, what do you think it's there for?
R: Well, I'll tell you what, I was very excited when I first heard of e-referrals. Only because within the unit where I work, the units, if a patient is discharged or if they arrive, one, is I don't know if they've been contacted by the hospital prior to transfer to my facility, that's the first thing. That information is not necessarily in the medical history, the medicines history or medicines reconciliation, that's the first one. And the second thing is, is because I work between units my contact with the community pharmacy that if they had a blister pack, for example, would be via fax, via fax. But quite often they would get discharged and I wouldn't be at that facility and I missed the opportunity to actually inform the community pharmacy of what the patient has actually been discharged with. So, the beauty of the e-referral, I'm not being funny, but the beauty of it, is that firstly we get to inform the community pharmacy that the patient is admitted into hospital, they get to know that electronically. And the second thing is whether or not I'm at the facility, once an e-referral has been setup they get a discharge summary once the patient goes. So, it takes the pressure off of me, trying to remember whether or not we've informed community pharmacists, et cetera. I've got a visualisation, I can see now whether or not a patient has had an e-referral, and I know that's going to go once they get discharged, so that takes the pressure off of me, I love it, I absolutely love it.

HP5
I: Yes, so do you think that the purpose of the service, what it's trying to achieve then is that sort of joined up in communication…?
R: It links everybody, it absolutely it links everybody, there's not a break in the care of the patient so to speak. The community pharmacy know that the patient is no longer under their care when an e-referral is sent, so it means that they will withhold any blister packs, for example, or sending them because changes could be made. So, if they withhold it that means there's not the risk of the patient going home, say they go home with a week, and then going back into a blister pack, perhaps with a drug that perhaps caused their admission, the admission in the first place, that they still have at home, that has been sent to us as they've been in hospital. So, it really does complete the circle of communication between all disciplines involved.

HP5
I: So, some of the, for some of your patients because they've come, they are in that sort of stepping stone out of hospital, some of them would have already had the e-referral happen in hospital.
R: Yes, the majority would have.
I: Right, but you check that that's the case.
R: Yes, but I check to make sure that that's the case.
I: That's interesting isn't it.
R: Because, you know, even though in their records, in the medicines reconciliation or drug history might have that they use a blister pack, there are occasions where whoever has done the meds rec previously might not have the opportunity to do community pharmacy referral. And the

reason why that might happen is, because the patient might not know which chemist they actually get a compliance aid from or anything. So, it's got that they use it, but nobody, they might not know. Because some patients come in and obviously, they are confused at times or whatever and it's a traumatic event, they might not be able to give that information. But they are more relaxed by the time they get to my facility and therefore, I make sure that I get that information, or I get it from the family. And it's important to have that information because at the point of discharge not all of my patients go back to where they came from initially.

HP5

I: Yes, that's really interesting actually and you talk there a lot about that continuity of care, because of actually one…and you also talked there about the…in terms of that, in terms of patients because one of the questions there is, you know, do you think the service can improve care, will it improve care?

R: Yes, definitely.

I: In what, why will it do that?

R: Because the information received is accurate. It really is accurate; everybody is getting the same information.

I: Yes, precisely.

R: And you've not got a break in information. Because a patient, a scenario we've had in the past is a patient, we might send a summary to a patient, they might go to community pharmacy to get a supply of the drugs, community pharmacy isn't aware they've been sent into the hospital and gives them something, a new blister pack. Perhaps they might have had a couple of prescriptions left from prior to the admission and then they get drugs that have been, that has been stopped that caused hospital admission.

I: And could have caused a hospital admission.

R: Exactly, so yes, so definitely because there is communication between all services involved and you've got now exactly the same information moving between all of them. The risk of having errors like that made is minimised.

HP6

So this is one scenario, there could be other reasons, like I come across today, a patient who has been rejected by (name of pharmacy). I went on the EPR, checked the records and I found out that this is the right pharmacy that is mentioned on the EPR, it's not the wrong pharmacy but the reason for rejection, that (name of pharmacy) is not doing Venalink for (name of care home) anymore. So the patient has been sent out, discharged from the hospital to (name of care home) but (name of pharmacy), who receive the information from the hospital, is not dealing with (name of care home) anymore, it is another pharmacy. So I spoke to them and I found out, at the beginning, because maybe they are under pressure, I've asked them, do you know who is dealing with (name of care home), which is a logical question. They said absolutely no idea and I was shocked, that was a prompt answer, which I didn't like and then I asked them, is anyone around you. No, she said I don't think so, but then she said, hang on, hang on, maybe someone was overhearing that conversation and said (name of pharmacy)Then, I went back to (name), because I don't know where that pharmacy is, (name) has much more experience than me and she said, we have a list of the pharmacists that do Venalink for care homes and yes, I found it on the computer and then I found the number for that pharmacy, I rang them and I confirmed that. It was listed on our PharmOutcomes anyway, but I just wanted to double-check that this is the right pharmacy before letting it go to that pharmacy. I called them and they confirmed the address that I mentioned on the PharmOutcomes and I saved it on our PharmOutcomes, so that it's gone to the right place.

HP6

R1: Actually, there shouldn't, if the faxing, if it goes from the first time, there shouldn't be a communication between myself and the pharmacy. So the e-referral does, there's no need for communication at all, it's just because we are trying to send the information in the right way, it has to be sent like that because other ways of sending information could be, not effective. Or could carry mistakes, could end up with mistakes, like with the drugs. Because some drugs carry very similar names, like promethazine or promazine, sometimes a spelling mistake might change something to something else. Because e-referral or faxing, send the information printed clearly, not every time by fax it goes clearly but most of the time, it goes clearly, with all the details and it ends up with zero mistakes, if it's sent properly. But the faxing is time-consuming, while the e-referral, it's not at all.

HP7

R: So, when I speak to a patient, when they are admitted. I find out if they have got a blister pack, I'll find out what pharmacy it is and if it's a (place) Pharmacy, I will go on the electronic system; there is a little link to get to it.

I: Right.

R: There is a link so you just click it, you type in – I always type in, the postcode of the pharmacy, because if you type in the name it's not going to map it to the map, so it's better to just type the name and the postcode, find it on the map. And, then I'll send it. Sometimes if the patients don't bring in their blister packs, and I don't know what time slots things go in, I might have to ring the pharmacy, or I will ask the family to bring the blister pack in.

I: Right. That's interesting because you've got, you are telling the pharmacist at that point of admission to stop making up the blister pack, and effectively that's the point of that notification.

R: Yeah, don't send it out for delivery, yeah.

HP7

R: So, they will get an email from [Pharma 12:46] pharmacy outcomes to basically tell them that they are in hospital. I have actually also used it for patients who are on methadone.

I: Right, okay.

R: Because there was an incident where the patient was admitted and I just, you are supposed to ring the community pharmacy and tell them that the patient has been admitted to hospital when they are on methadone.

I: Yes.

R: And, the line was busy all day, I couldn't get through. So, I used the e referral to communicate to them. So, the next day when I rang them, they were like, yeah we received your e-referral. So, that was good.

I: Yeah. And, that's useful.

R: Yeah, it cannot just be for compliance aids it can be used for that.

I: Yeah, and that can be useful because they can…

R: They'll know no matter, yeah if they are not answering the line or the phone has gone or something.

HP8
I: I think in some respects, until I was talking to you today, I have been saying to people, this is very linear. This is very, sort of, one way. It's very, sort of, like, hospital - community pharmacy communication. Where's the communication back from that. But actually, your bit is, sort of, it's a little hook at the end of it, isn't it, coming back to practice. Do you think there should be more of sort of a rounded communication going on?
R: Oh, definitely, yeah. I think it's part of integrated care that everyone should talk to each other about the patient, if possible, rather than just say, right, I've done my bit, that's it. You know, I'll wash my hands of the patient now, and they're on to the next thing. Because a lot of the patients that we are seeing have these chronic long-term conditions and they are probably going to be back in hospital and they're probably going to be going to their community pharmacy for a very long period of time. You know, it's not like healthy people who might have a prescription for a month and then, you know.

HP9
I: …on the discharge summary. And they might not match up. The discharge summary list should have been sent to the community pharmacist. What I'm trying to think of there is then if these things are happening, in what ways then is the referral going to help that?
R: Help it. Well, because I think the issue that…from working in (name of place) and now, the issue is with the discharges being sent to the chemist, it doesn't always happen because it is done by fax and it relies on the pharmacist actually calling the chemist, which they don't always, but their number could be busy for hours and hours. It doesn't sometimes…either the discharge doesn't arrive via the fax because there's a problem with the fax machine.
I: So you get discharges here by fax?
R: No, electronically…
I: Electronically.
R: …but it used to…
I: …it used to be by fax, yeah.
R: …it used to be…to the chemist it…
I: …by fax.
R: …used to be by fax until this new system came, and that used to be a nightmare. I know from working in the hospital and here, it just…it's a nightmare for both ends because it doesn't…fax is not really reliable and doesn't always work and the number's busy and sometimes they don't receive the full fax. So that causes a lot of issues and they always used to complain to the hospital that they'd not received it but then it's not always the hospital because they might have done their part but it's just not got through, so it's just completely not a reliable way of sending something that contains confidential information as well on the patient.

HP9
I: Yeah, and that was one of the other things that people were talking about last year with the service, was that this would…the potential for this to lead to patients better-informed about the medicines. Do you think that's likely?
R: Yeah. I think so. I think that's the potential with it, yeah, 'cause again, when you say, rolling it out to all patients, because there's people not on a Venalink but are on ten medications and something would have changed in hospital but then if nobody tells them about it then they don't know. They might have a box of Ramipril at home and it's been stopped at the hospital and if someone briefly mentioned that to them or not mentioned it at all then they'll go home and start taking that again. And if the chemist is not informed of that then they might have a box on the shelf waiting for them that repeat prescription or they'll just give them that and then this will just carry on. It's like a – what's it called – the Swiss cheese and when it's like an error, just yeah….
I: Just going through the hole, yeah.
R: …gets passed, yeah…
I: Precisely.
R: …basically. Yeah, and this is a potential…if something stopped they could tell them, this has stopped, they can maybe take it out of the bag and not give them that obviously medication…

P1_C1
R1: But the GP, Dr (name) she explains everything. She is really good.
R2: Well she does and she doesn't. She doesn't explain what the tablets actually do.
I: All do.
R2: And what interacts with them.
I: Yeah.
R2: She's good in the fact that she's aware of what mum's conditions are and why the tablets have been given.
I: So, basically you would like, more information would be better?
R2: Yeah. Even with the venapack, even if, obviously if it was going to be regularly changed, then that would defeat the object, but if you've got regular doses and you are on them for however many, even for 12 months, if one piece of information, like a piece of paper of the all the drugs that's on and what the interactions would be, what to avoid, what not to avoid, if you got that information, with the pack, just the once, so you've got them here. And then when it's changed, that information changes with it.

4.2.4 Patient preference in relationships with health professionals

C2_C3
I: Yeah, yeah. So you're pretty involved in everything, aren't you, but I'm interested in how you've got that information about her medicines. Who are the people you've been talking to? Is it the pharmacist, the doctors?
R1: Well, before it was doctor.
I: Oh yeah, you said.
R1: He's retired. He was really good, him. You only had to phone him.
R2: He knew my gran.
R1: He knew my mum because he'd been a doctor for years and anything, any problem he'd come right out and if he thought her blood levels were down, really spot on, him, he's really like old-fashioned doctor sort of thing. You know him and he's really helpful and any problems with the meds he'd right away sort it out. There was one time a few years ago they changed your blood pressure, didn't they, and must have been cutting

down on costs and my mum had a bit of a reaction, but right away he just put on a note under no circumstances, this has got to be changed. She stays on that medicine, that blood pressure tablet. He's really spot on. But like I say he's retired now.

C2_C3
R1: Yeah, communication with doctors is really good.
R2: I think it's good when like my gran, like my mum said, he was a family doctor, so the doctor knew my gran, knows his patients, and I think that benefited him, that, and benefited the surgery.
R1: Yeah, you weren't just a face like.
R2: My doctor's. I used to have one doctor all the time and when I book now I'm seeing all different kinds of doctors, because it's just the way it is now, isn't it?
R1: That's it, yeah.
R2: But I think that [voices overlap 14:58] like my mum said with the tablets, if she would have phoned and spoken to a different doctor, she would have had to explain it all and why she's on these.3MAJ11/02/2020 13:29
I: So you phone them rather than the chemist?
R1: Well, if it's something minor I'll phone the chemist, if it's not turned up. But obviously if it's something that's changed in the meds, if it happened where they hadn't told me or they put another one in, I'd phone the doctor to say, well, have I got to give her this, just double check like that.

HP15
R: So, we can do an MUR up to eight weeks after they have been discharged, maximum. So that's why we don't do it straight away. We leave it for three, four weeks when they are much better.
I: For that same reason. Yeah.
R: Yeah. Where we ask them to come in, when they are feeling better. But they still won't want to come, because they feel, oh they've already had all this in the hospital, we don't want to. So even when we check on the phone, and they understand what the new medicines are for, if not they can just come in and we can speak to them quickly about it, see if everything's okay. So, they say, oh no no I've had my GP check this, and they don't want to come in, some patients. But some people happily come in. Yeah.
I: Yeah. Well that's interesting, isn't it?
R: Yeah. For some of them you can clearly tell they don't understand their medication. They don't know what they are taking, why they are they taking. But they still don't want to come in to talk about it, no matter what you try. We try different ways to get them in. But no, if they don't want to come, they won't come.

HP16
R: So for us that's the key thing really is we want to make a difference to patients, we want to make a difference to the community and to the patients that we serve, if any new patients come up they will get the same treatment as anybody else, do you know what I mean? But we're not in it to poach patients and all these things, we're in it for the care of our patients, whoever we serve. And the same way GP practice won't go out and start going...stood outside another surgery going, come to us, come to us, we're not going to stand outside another pharmacy or the GP practice going, come to us, come to us. It's just absurd. And I think the vision, the view is that we are going to get monetary benefit for doing this, which we're not, it's about the care and if somebody wants to choose us they're welcome to choose us, if they want to go somewhere else they're more than welcome to.

P3
I: Right, but it's always from there.
R: It's always from there, they're usually great, you know, usually walk in, they say, (name), and they just, you know, and it's there in a bag all ready for me. It's only recently it's started to go downhill. But I put that down to this new system they had, and our surgery, because that's where the problem started initially…
I: So the communication between the two.
R: Yeah.
I: Yeah, that's interesting, yeah.
R: Yeah. I find the surgery sometimes can be a bit lax because there are so many different doctors, you never know who you're going to see when you make an appointment.

P3
R: I prefer face-to-face, but you can't get an appointment with our pharmacist at the surgery, you can have a phone call but you can't come in and talk to them. And if you ask the doctors what's that tablet for then they'll tell you, you know, but they don't just sit down and tell you what, you know, they're going to put you on this now and that should help with so-and-so. But then as I say, the two blood pressure tablets I was on that they stopped, the consultant in the EAU that time said, you've medication here that's working against each other, he said, and you've also got some that's working too well.

P4_C4_C5
I: Right. And you seem to have a relationship with the pharmacist?
R2: Yeah.
R1: Oh, we've known her for donkey's years.
I: Does she talk to you a lot about the medicines, do you…?
R2: No usually it's the girls who bring it to the door for us. You know, it's delivered.
I: Right.
R1: There's (name) and…
R2: (name) and… (name) and that, yeah.
R1: There's your tablets (name of patient), and they give you a bag of tablets.
R2: Yeah, and they'll still talk, but they'll tell me if I want to ask anything.
I: Right.
R2: You know, I'll say, what's this for? And she'll say, oh that's for thing, his legs.

P4_C4_C5
I: Yeah.
R3: He's bad on his feet, he's also not in the best of health.
R2: He can't see proper.
R3: And it's like, what you're doing to him, what are you doing to his body? If you change it, change it, but keep up with the… 'cause there was one time, where he had to lose weight, and he was told to be seen in four weeks' time.
R1: Oh yeah, that's right.
R3: That was ridiculous. So he's trying to lose weight, he couldn't even get an appointment for four weeks' time, then they changed his doctor and he was under two consultants, and they're contradicting each other, and you just don't know…
R2: What's the best.
R1: One was about the weight wasn't he?
R2: Yeah.
R3: Yeah.
R1: Get rid of your weight, and the other one said…
R2: That's when they upped your water tablets.
R3: Who's right and who's wrong? Do you take that tablet, or do you take that tablet?
R2: Antibiotics - have a lot of fluid and drink a lot of water. Don't drink a lot of water with his legs.

4b 5 Code - Relationships between different health professionals and what this achieves

HP1
I: Yeah, precisely, yeah. So, some limited information coming through, information is difficult to unpick, unravel, difficult to act upon. How is that then fitting into the rest of what you're doing, in terms of your work? Is it slowing the rest of your work down, is it changing the way you work, or what?
R: I mean, the numbers that we're receiving, it's not really changing the way that I work, it's just something, as and when I get a notification, I just need to deal with it. I think that is part of my job role, so I don't think it's affecting me. I'm quite chatty and just speak to anyone, and anything anyway, but some pharmacists, some of our inexperienced band 7s, might be less chatty, so I think it's good, in that it promotes them to pick up the phone and call their local pharmacy and say, oh hi, I'm the pharmacist, what's happened, I've just received this notification? Which is the whole point in the whole three way communication, so I think it is good to have these every now and then. It's just, if it was a little bit more slick, it would be nicer.

HP1
I: What do you think the impact upon, well a couple of things, the impact upon working relationships across those transitions, between yourselves and community pharmacists, yourselves and hospital pharmacists. What sort of changes do you think the service is going to make to those sorts of working relationships?
R: I think it will help with knowing who's who. 'Cause there's so many of us now, and all over the place, we're like ants. So, I think it will help us know who is in what team, and what their role is. And it will also, it's more streamlining, sending information across, so again, assuming it's three way communication, we could then just send a quick notification via pharm outcomes, to the community pharmacy, and they will then send one back, instead of being on the phone for endless hours waiting for someone to pick up, or…I think definitely, and also it's a formal way of communicating. Sometimes, there have been occasions where it's been, oh you didn't tell me that, oh I did tell you, why didn't you…you know? So, having it written down, formally, is also safer and a better way of communicating I think. And also, the hospital, definitely, it's useful knowing what ward they're in, and kind of…
I: Why does that help?
R: Because, sometimes, if there was a query, so on the discharge, they might not have put something, and we just wanted to know, it's easier to go straight to the ward, and say, do you remember this patient that you've just discharged? Yeah, so that was useful. Yeah.

HP10.
I: And that surgery has pharmacists working in it as well?
R: They do. They do have a pharmacist, I'm pretty sure. I don't know how the project is called but employed by (name of NHS trust), and then there's another guy, this is a different kind of way, where he's more of an independent prescriber, but I think they work together. Both of them are happy to look at the... At the moment, when they get a discharge, they will look at the changes, they will implement whatever new meds need implementing and put them on prescriptions, put them on repeats, make sure that the blood tests are arranged for the patients to be done, with regards to certain medications that was asked by the hospital setting. So, yeah, it works much, much better and I'm already kind of going ahead, but in the past it wasn't the case.

HP10
I: Yeah, that's the sense of the journey through, because that's really interesting, because you sort of like go from the note, putting it on hold, through to the checking of the discharge summary. But what I'm really interested in there is that communication with the GP. Is that something that's new or has that always been like that? Or is that the sort of because you've now got this extra information coming with the discharge summary through the e-referral system?
R: I think it's a number of things. I definitely think this discharge has improved it enormously. We've got this meaningful... It's kind of... You know, on a discharge sent through the fax, you could sometimes hardly see what's on it. It was hand-written very often, it was just things coming through the fax. There was, like, ink covering certain bits, the information wasn't very good. If you had to pass that on to someone else, fax it again, or scan it and send it via the email, it wasn't very clear, where this is I've got a PDF, so I can easily just save it on my PC and send it as an email to my doctors, which I do, but they prefer the fax. For some reason they prefer that, they pick it up quicker than an email, but I'm talking about next door. But, overall, this has improved it. The other thing I would say is the fact that I've been there for a bit, I know all the main doctors, I know everybody there, I'm quite a proactive guy, I engage a lot with them. I used to attend more often the meetings, so we know each other well, so they don't fob me off, they respect me, they know I'm not wasting their time, they know there is some meaning there. I think we've got

HP10

I: Is that widening of general practice isn't it
R: Yeah, exactly, so pharmacists are very much now an essential part of the surgery, I think, there's no exception that this one is, I think it's overall. And they all engage with... Again, to me, my understanding is they engage with all kinds of activities to do with prescribing, every single thing I'll be talking through pharmacists. Very rarely these days I will speak to the GP directly unless they've got something, you know.
I: Well, that in itself is quite interesting, isn't it, that you use them...that's their role in some respects...
R: Yeah.
I: ...in that sort of middle ground between yourselves.
R: Yeah, like a bridge.
I: And the GP.
R: Bridging, yeah.

HP11
I: …yeah, once that discharge information comes through, 'cause it's quite a lot of checking and you're checking against what was previously there, making the changes, getting the script generated from the... What helps make that all work?
R: Well, having the information, doesn't it?
I: Yeah.
R: Having the information and having the relationship with the…we had a previous practice pharmacist who was really unco-operative, who have moved elsewhere. But she was just really awkward and it just made our life so difficult. But she left. So that's good.
I: Yeah.
R: Unfortunately she's gone to one of our other pharmacists. Well, she's gone to a practice where we have another pharmacy, unfortunately for them.

HP12
R: Which I think is quite a safety issue. So, we've got a lot more time to sort everything out. We've got time to sort the prescriptions with the surgery; they've got time to look at it properly, to work out everything. So, that's worked much better. And we're not bothering the surgery saying; please can you print me out a discharge summary.
I: Yeah, because you've already got it.
R: It sounds awful to say but if the surgery just do us a new lot of prescriptions, we prefer to check that against the discharge summary, just to make sure that everything is as it is on the discharge summary. I know the doctors have issued the prescription but we prefer, as a double check, to check it against the discharge summary. So, sometimes they were getting a bit…why don't you get one and blah blah…so, we've got that information and like I say, it's the other way round now, we are taking it through to them saying, this patient has been discharged, here is the discharge summary, please can we have prescriptions.

HP12
R: Yeah. And we've got quite a good relationship with the practice pharmacist that's working at the surgery now, the previous one we didn't have, but that's another story! But this current one we've got a really good relationship and he's just like, yeah, if you can print me off an extra copy that's great, it's much quicker.

HP12
I: Another question is about working relationships. One of the things I wanted to expand that to is, has this changed the relationships you have with the – I mean by large I presume most of your patients are from next door.
R: The vast majority are from next door, yeah.
I: So, has it changed the relationship with the general practice?
R: Yeah. I mean the relationship has changed a lot anyway because of this change of practice pharmacist. We have always had a very very good relationship with the practice next door, which deteriorated when we had the previous practice pharmacist. There became quite a lot of issues.
I: Right.
R: And now we've got the new one the relationship is great again now. And I think from their point of view it's probably benefitted the relationship because we're not hassling them, we need these scripts by today, we need them now, you know, the patient's no medication.
I: Because you've got more time?
R: Because we've got time to sort it out.
I: Because if you are seeing the discharge summary within a couple of hours, you have got seven days before that patient has run out.

HP12
R: But I think providing…I mean we're lucky because we are directly next to the surgery, so we can sort everything really quickly and I think if you were a high street pharmacy and dealing with a lot of different surgeries, the whole process might be more difficult.
I: Yeah.
R: And you might need that extra communication. We get away with it really because of the relationship we have with them and the close proximity we are to them.
I: Presumably even with a one off acute, scripts just come, walks out of there walks straight in, because it's convenient.
R: Yeah. We've got the proximity and we've got the relationship with them. And in our particular situation, we probably don't need that third thing between…[voices overlap 35:40] because it's kind of working as it is. The vast majority of our venalink patients are from the surgery next door; we have a few that aren't. But that's quite interesting this week, so we had a patient that was from a different surgery from (name of GP practice), who's been discharged. So, we'd had the discharge summary, we'd spoken to the surgery who said, send us the information over, and then on Monday the practice pharmacists from that practice rang me up and said right we've gone through the discharge summary, I've issued the prescriptions, we've not issued this for such a reason, but there was that conversation there so then when we got the prescription through electronically it wasn't like, why have you not done. It was good to have that communication.

HP13
I: In terms of communication, you talked a little bit about the communication with the doctors there, what about the communication back and forward to the hospital, is there any communication there at all?

R: No, 'cause you can't really get through to (name of hospital) much, the pharmacy, you wouldn't be able to actually ring them up, no, you don't really need to with this, to be fair, 'cause once they've told you what the patient's medicines, it's all there so you wouldn't need to, I've never had to phone them up.

I: Right, yeah, 'cause one of the things that I was thinking is, you know, it's a bit linear, the information is coming out of the hospital, coming to you but actually, once they provide you with that information, that's all they need to really do, isn't it, unless there's any queries that you…

R: Well, if there's a query, to be fair, I think actually I would pass that on to the GP, if there was a real query. Because at the end of the day, I'm not the clinical person producing the prescriptions, so you know what I mean, if there was an actual, real thing that I thought, I'm not happy with this, I'd put on PharmOutcomes that such and such doesn't look right or whatever and inform the GP and then I'd phone the GP. I mean, I don't rely on just the email on PharmOutcomes, I do actually phone them if there's a problem, I would throw that back to the GP, not the hospital and I'd let the GP deal with the hospital, yeah, really.

HP13

R: It's better than, yes, it's better than, yeah. It's not only that, I've forgot about the actual patient, which is important. I'm involving the patients a lot more than I used to.

I: In what ways?

R: Because I know I've got the discharge, I will then…I've got it early, I've now…because it's the PharmOutcomes, you have to tick things that you've done. One of the things I will do now which I wouldn't have actually done necessarily, I will phone the patient, I'll let them know that we know they've been in hospital, we've got a discharge, they're not to worry if they will be coming later in the week and blah de blah, you know, if it's the same I tell them. And then also when I go to the patient with it or whoever goes, it's me, usually, if they've been in hospital I will go and if there are changes I do a medicine review with them all about the changes to let them know.

I: In their home?

R: Yeah, in their home, yeah, what it is about and I would not have done that before. I didn't take it as…just because PharmOutcomes is very well designed 'cause it draws your attention to the fact that that's what you should be doing, so that's what I do. I've found that that's helpful, very helpful and I think the patients find it helpful, they've all been very grateful because maybe they haven't understood what the doctor had said to them, maybe they didn't know that their medication was changing or they weren't listening.

I: That new medicine or counselling bit that you're doing there, is that new or is that something….?

R: No, I really didn't do that much before. Now and again I would've done but not with every discharge at all whereas now I am doing, if it's changed. If it's not changed, I just phone them and say there are no changes, we'll be along with your medicines on Thursday as usual, end of story. But if it's changed, it's making me do things I would not have done.

I: That's fascinating, actually.

R: Whether everyone is, I don't know but I feel that it's definitely made a big impact that way.

HP13

I: There's a change in terms of your relationship with those patients, your communication with those patients, which is actually something I think the service was looking to do in the first place.

R: It sounds a bit bad actually but I think before, I just used to think, oh they've been in hospital, that's the GP's responsibility but clearly in the way I'm seeing it now that no, it's slightly my responsibility 'cause we're getting this thing so therefore I've got to put input into it. I think that's the difference was a fax thing, we weren't really…I don't think we were taking them on board as much as we should've done.

I: Yes, and you were pulling the patient into that as well.

R: Yeah, exactly so.

HP13

R: I just feel I'm giving those patients a much better service because of this than I used to. I just feel that and I'm sure they feel that too, you know.

I: Because of that conversation?

R: Yeah, I really did used to think it was always the doctors' responsibility whereas now I had to be dragged into it and I prefer that. I feel as if it's a good…you're giving them a good service and making sure there's no errors, yeah.

I: And do you feel the patients value that?

R: Oh yeah they do, definitely, 'cause that's another thing, when I call at their house, I'll take the old packs away 'cause that's another issue if the medicines have changed, you don't want them picking up two weeks' ago, you don't know how long they've been in hospital, they might've been in hospital two months, they might have loads of packs there so I'll clear them out when I take the new pack. And I will write on the new pack so I know to tell them that this is the new, up-to-date pack and I get rid of all the others and take them away.

I: Right, yeah, and so the patient, yeah.

R: I didn't used to do that, I don't think I did used to do that, I just used to deliver it and say, that's your new medicines, you see. It's just a safer service, really, it's made it safer.

HP13

I: One of the things that was thought of to start with, this would be a triangular communication between practice-based pharmacies, hospital pharmacies and community pharmacies. Do you think that would be…do you have anything to do with practice-based pharmacies at GPs?

R: Oh yes, yeah.

I: What are relationships like or do you talk to them about these?

R: They're not great, strangely, they're not great always, no. But yeah, we'll have to talk to them, yeah. Although I find actually a lot of them, the reason I'm saying it's not great at first, a lot of them are fine but I think now they won't talk to you directly, which I don't like.

I: That's interesting, yeah.

R: You have to pass the message to some receptionist. I don't like passing messages to someone to pass a message. If it's complicated I say no, I don't want to pass a message, just tell them I need to speak to them directly. I don't know why that is, whether they're busier or whether they feel that they don't want to be interrupted.

HP16

I: One of the things, as you well know, at the beginning there was this idea that this would be triangle between the NIPPS pharmacists, yourselves - community pharmacy and the hospital, that's not really happening.
R: No.
I: It's quite a linear thing.
R: Yes.
I: Do you think it would be better if it was more of a triangle…?
R: I think it would be better because what we would do is we would see inter-referrals, so actually what we want to see is the discharge MRU come to us but then the practice pharmacist could see I've done a discharge MRU and a quick bit of note on it, these are the couple of issues that have come up, can you deal with those, please? And vice versa, they could say, actually I've just started somebody on an inhaler, can you MNS them for me? And the same way the GP (inaudible) goes why can't they refer to us?
I: Yes, so there would be a referring back and forward between?
R: Yes, but it would be one...
I: Joined up.
R: One joined up approach so that if that patient then went back in the hospital pharmacist could see, oh, there've been some conversations here, so I can see the community pharmacist talked about your inhalers, I can see, oh, the practice pharmacist has started something new and asked the community pharmacist to do this as well, I can see that's happened. Brilliant, so you're really good on your inhalers so actually do I need to counsel you about your inhalers or do I need to counsel you about allergen avoidance or do I need to counsel you about something else which is causing you to have that problem? Do we need to think about changing the inhalers because obviously your inhaler's already optimised? So it's a better conversation for the patient, for the pharmacist, it's more valuable but actually, again, for the overall outcome.

HP16
R: I think we've not seen it in our area, our area's probably slightly unique in that, candidly, our GPs are quite archaic.
I: There's quite a few single-hander GPs here.
R: We've got a couple of single-handers but the big practices also don't...they're too worried about, well, we can't talk to one pharmacy and not the other and all these things, and we're getting into all this debate all the time, it's like, well, actually if I've come to you with a question...
I: About a patient.
R: Not even a patient but actually a service or something then we're doing it for the benefits of patients, that's our patient cohort, it doesn't need to be the whole of the...
I: Yes, it's the patient cohort.
R: It's the patient cohort that we serve and there are a significant number at your practice. So I think in other areas I've seen it work very, very well, for us probably not as much but it is getting better.
I: Yeah, because I know a while back, I think, there were some single handers hanging on, as it were, and near to retirement.
R: Yes, we've got three or four actually in this area who are single handed but the issue is that's all great but actually you've got a NIPPs network there but the NIPPs network aren't necessarily linking in with the pharmacies in this area essentially, it could be because we have so many practices, I don't know whether it's because they don't get around to the pharmacies or what but we've never had a NIPP come in here to say hello.

HP16
R: It's down to the NIPP I think.
I: Yeah, and it's down to that and what's happening with that.
R: And I've worked equivalent to a NIPP before and my first job was to go and say hello to all the pharmacies in the area because to me with the issues that we have in the market, stock availability, all those things, we're an invaluable resource for them. Is this available? No. Is this available? No. What's the alternative? Here you go.
I: And just being able to know what issues there are.
R: But it's also the medicine queries, they're meant to be there to support us with our medicine queries as well as the practices, so to resolve issues around medicines so if you have a complex patient who has issues then they need to be resolved and we want to have a point of contact for those.

HP17
But I mean you've got summary care records, which is better now, but from past experience this new system of getting electronic referral is brilliant because you know instantly…you know when they go in hospital, you get an email for that and you can bring it up on PharmOutcomes, and you know when they come out, and you get…all the information that the doctors get you get, I'm assuming the same, or very similar, information. So, I've had problems in the past with one particular surgery, not mentioning any names, that wouldn't even give me a discharge sheet from the doctors, they refused, the practice manager refused to give me a discharge sheet, and this is what she said to me, not mentioning any names, was, 'well the doctor's done the prescription, it'll be right because the doctor's done it, and we're not releasing a discharge sheet.' But what happens if it's not right? What happens if the doctor's made a mistake? What happens if it's been transcribed wrong? It's my job as a pharmacist to check what they've come out of hospital matches what the GP's prescribed and I can't do that if I've not got a discharge sheet.2MAJ17/04/2020 18:37
R: Yeah. So, something might have been stopped but because the GP's maybe not noticed or was rushing and he's stressed in his job, or her job, and they've not noticed that this has been stopped, or that's been started. So, you could get multiple…that one that I just told you about was two diuretics together. You could get all sorts of different changes, it could be anything, and you could get something stopped that the GP's still continuing. So, anything I notice that doesn't match the discharge sheet I'll query. But my dispenser here, when I came, she was a bit reluctant to query and go against the GP and say, well why's this on, or, why's that not on, because once the GP went mad at her and that then changed her attitude. So, he said…this GP said, 'why are you questioning me, I'm the GP, I prescribe this?' Well it's not on there. So, then this surgery manages their own prescriptions. So, then the other thing is…so one surgery, they do all their own prescriptions and we don't do any ordering, they have somebody that's employed full time just to do all the prescriptions and work out when they're due and print them all off. All we get is prescriptions and we just ask for a name. Not mentioning who this surgery is, of course, but I said, well if something's not there or something's not thingy you don't just assume it's right, you need to question everything; so why, for example, with this patient that was on 30 milligram of Citalopram and then all of a sudden it drops to 20, where's the extra 10 one that they'd only just started two weeks ago? Don't know, it's not arrived, they ordered the wrong prescription so maybe they've not done it. Well let's question it and let's ask them. Well we don't question this surgery. Yes, we do, we'll get back on the phone now and find out what's going on.

HP2

I: What happens once the patient has left is going to maybe have an impact or whether they come back at all.

R: If, you know…once that patient goes home, if somebody then can have contact with that patient, maybe in their own home, or the patient goes into the GP surgery and speaks to pharmacists or technicians in there, just to keep up-to-date with that patient to say, are you fine with your blister pack? How can we help you? Are you finding any problems? That kind of thing could work in the community and I don't know if people are doing that kind of thing now. So, I don't know how it can help…like the hospital pharmacy, once we've done that referral and they've left, I don't know what benefit we can…

I: When you do the referral, do ever put an action on for the…right.

R: No. I don't.

I: So, you only tell them what's happened.

R: Yeah. We let them know that the patient's in or they've gone, and then they'll receive the discharge summary, which has…the doctor will write why the patient has been in, what actions they've taken, medication they came in on, if anything was added, if anything was stopped. Yeah. So, that's it then, we've…it's finished from the pharmacy department then.

I: Yes, then so then it's…

R: Then it is up to the community…

I: To deal with…

R: …to deal with medications, but then maybe social issues, that brought the patient in, whether it is medication-wise or not, I don't know. If it could help, it could benefit somebody in that way. Another team.

HP4

R: Are continued, yeah, that's right. The only…I guess the only downfall slightly is that our system doesn't necessarily show that it's had a pharmacist clinical check. So if a patient's discharged but the nurses or the doctors don't tell pharmacy about the discharge summary, that discharge will automatically still ping off to the community pharmacy, but it might not have had a clinical check, so there might still be errors on that. That's one of the big risks I think in our kind of system…

I: Because unless they make any changes, they don't need to involve you, is that what…?

R: So we try to encourage doctors and nurses to inform us of all discharges basically so we can cast our eye over it, because a lot of the time you do see doctors writing, no changes to medicines, or… We did a recent audit on EAU where we found we changed nine out of ten prescriptions in terms of documentation and prescribing. So if you imagine that and if you imagine that say out of hours overnight if pharmacy aren't open or if a nurse just says, oh, there's no changes, I'm not going to bother telling pharmacy, we'll just discharge the patient, and then that automatically goes off and those errors are continued.

I: Errors can carry on, yeah.

R: That is still a risk, but it's difficult to get around that, so other than encouraging staff to inform pharmacy of all discharges, problem is pharmacy aren't there 24/7, at the moment we just have 9:00-5:00 really, 9:00-7:00 on EAU, so that is still a risk. So the problem with this e-referral I guess is that you can't guarantee it's necessarily going to be correct. If a pharmacist has been involved, you'd hope it was correct, but you don't necessarily… So that's why we annotate I guess if we've seen it or not, if we've clinically checked it, it puts our electronic stamp on it to say, we have clinically checked this. If it doesn't have that and there's errors, I guess the community pharmacy could then ring and say, is this right, has anyone actually seen it from pharmacy, is it…

I: Because you do the order at admission, if you then don't get involved in the discharge, it goes without…

R: Yeah, that's it, it goes. It goes, yeah.

I: …you having anything…

R: That's right, yeah.

I: And it's about keeping people…

R: It really is, yeah, so there is still an element of communication and unreliability to some extent. It's just better than it was.

HP8

I: Right, okay. So then that was devised that that was what was going to happen. We can go on as to how it's happened, whether it's worked, as we move on. So when it went live was there any initial, sort of, like, response from your end what was happening? Did you see that, oh, gosh, suddenly it's all happened?

R: Not really. It was difficult. So initially I don't think the email address had actually been set up in time because we had to contact (name of hospital) IT to get them to set that up, making no comment on (name of hospital) IT. So I was set up instead with the log in for PharmOutcomes, which is a portal, I'm not too sure, I think it's mainly used by community pharmacy. I was set up with a log on so that I could go and manually download the spreadsheets. So that's what I was doing for the first week or so, I believe, of the service, which was difficult because there was quite a lot of different reports I could have downloaded. So I was, sort of, downloading them, sending them to (name) and going is this the right one and she was, like, oh, I don't think it is, no, it's here. So there was a lot of screen shots back and forth in emails before I realised what the right one was, so that was in the initial couple of weeks. And then once I'd gotten the right report, it was then reading that report because when you open it, you just got a massive Excel spreadsheet, loads of columns, loads of rows, mostly numerical data. So, like, MRN numbers, ages, a few, I guess, multiple choice, like, male or female, and then there's some free text boxes as well. So locating where it was that I had to look to see whether a referral was made back to the GP surgery was the next big challenge. Eventually we found the column and, yeah, it would either say yes or no referral back to GP. And then there was some more free text boxes, which would say a little bit more about the problem that you can then read.

HP8

I: So it's not, like, it's with people with acute.... What are the issues around those patients then and how can this service help that, if you like?

R: I think medicines optimisation is definitely the biggest issue when people are, when you've got people who have polypharmacy. A lot of people, obviously they are not started on all the same medicines by the same people at the same time. So historically they've maybe got some things that have stayed on there for a long period of time that need to be reviewed. New things are being added that might interfere with old things, and people aren't sure, you know, what they're already taking. So that's one of the biggest challenges, I think. Another one is just integrated care. So knowing if someone has gone into hospital and when they are being discharged and what they will need then from a medicines perspective and when they'll need it. So we had one recently where the pharmacy wasn't happy with giving them a repeat of something they've been put on in hospital and asked us to book them in with their GP or the pharmacist to have a review of that before they would.

HP9_

I: So the e-referral work, how does that fit in with the broader work of being a practice pharmacist within the NIPPS service then?

R: Well, it fits in in terms of the chemist…what I've come across so far I think, it's the chemist referring people to us if they have any issues like, they'll say, can you look at this? This is what I've come across so far if they have a problem with the…yeah, if there's a discharge that they've got a problem with like their medications don't match up with the ones on the repeat then they'll flag it up to us and they'll get us to review.

P3

I: Have you had any problems with them not being ready on time, things like that, or have they always been ready when you've gone up to collect them, you collect them yourself?

R: Yeah, yeah. Normally they've always been ready. Lately, over the last few weeks, are you doing your shopping, they're not ready yet or they've not been checked, or on Monday, Tuesday when we went in just to see them and ask if they've got all the paperwork sorted, and they said they hadn't had anything, they had a phone call from the hospital, but that was all to say I was coming out. So I said, oh, so you haven't got a list of the meds…

I: Yeah, that's…

R: Yeah. But as I say, (name), one of the girls, she's very reliable and she's the one that deals with the surgeries, she's not been in for a couple of weeks, I think she had a breakdown that day. But no, she had an operation on her foot, but she is usually on the ball, anything going wrong, she spots it right away, yeah.

P4_C4_C5

I: Right. And you seem to have a relationship with the pharmacist?

R2: Yeah.

R1: Oh, we've known her for donkey's years.

I: Does she talk to you a lot about the medicines, do you…?

R2: No usually it's the girls who bring it to the door for us. You know, it's delivered.

I: Right.

R1: There's (name) and…

R2: (name) and… (name) and that, yeah.

R1: There's your tablets (name of patient), and they give you a bag of tablets.

R2: Yeah, and they'll still talk, but they'll tell me if I want to ask anything.

I: Right.

R2: You know, I'll say, what's this for? And she'll say, oh that's for thing, his legs.

Theme 5. Perceived impact and potential benefits - Hoped for, anticipated realised and unintended changes
5.1 Aspirations and expectations

C2_C3

R1: Well, she has four carers that come every day. They give her the first, the morning, lunch and tea. I give her the evening ones, because the simple reason, when they come at teatime…at about eight o'clock, don't they, and it's too early for her to take it, because they're sleeping tablets as well, so I give it her about half past ten, isn't it?

R2: Yeah.

R1: Yeah, about ten thirty, eleven o'clock.

I: So you're giving those medicines.

R1: Yeah, and she has these blister packs where it's all…

I: Blister packs, yeah, so all you're doing is you're popping out the stuff in the blister packs and then…

R1: The only thing I give her separately is her sleeping tablet, plus if she has anything extra like an antibiotic or anything. But they're more or less in that packet except for the sleeping tablet she has at night, so just add that to the…yeah.

C2_C3

I: How did those problems get sorted out then? Were you…

R1: I just phoned them up.

I: You phoned…

R1: And then said to them, like, this has been ordered, it should have been sent.

I: So are you pretty aware of what your mum's on and what she needs to have?

R1: Yeah, because I've been doing it coming up to three years now, so you get used to it.

R2: Been doing it longer than that, before the stroke. Not the same medication, but you're quite aware of everything.

R1: My mum's had bad arthritis for years anyway, haven't you, mum, so I've kind of been calling down before work and doing things and then my mum's like done the meals when she was…so when I'm work and that, so I've been more or less calling here every day anyway before the stroke.

HP10

This is far better in terms of we know what's going on, where before, we didn't have a clue, I'm afraid, at times, and there was just no system in place, where this is, in contrary, this is a system that is in place. We can talk about individual consciousness and letting people know this, that and the other, but this is just...and that's how it should be in such a big organisation and NHS systems are vital. So this is a good system, or looks like to me, from my perspective, it looks good and much better than what it was before. There was nothing there really. And improving the overall safety and efficiency and how things are...communication. I don't know what else to say, so that's it really.

HP10

I: Yes, precisely. Yes, actually, when it started and they said, oh, we're just going to put patients who are on blister packs, and I thought, oh, that's not many. And then I thought, well, actually, that's the most complicated group of patients, isn't it? Because as you said, they're all multiple morbidity, many drugs and so on and so forth. Having said that, would you welcome any sort of extension of the service, to doing more than just patients on blister packs? Do you think it could be for lots of people?

R:	It's interesting, this, you know. I'm struggling to give you a good answer to that because I feel, or I'm under the impression, that the community pharmacy at the moment is underfunded, honestly. From my perspective as a community pharmacy manager, I think... I've managed to retain my staff so far, but there are a huge amount of problems getting someone, employing people for this money that we pay. So you don't necessarily have that many resources and it's hard to motivate people. And we're doing numerous things at the moment and I'm thinking, what will it look like? I'm not sure what we're talking about. I'm guessing that... Because someone said, maybe yourselves, someone said it to me, but there is a plan of rolling this out as all the patients who belong to you say, oh...
I:	Yeah, everyone with a nominated pharmacy.
R:	...I'll go to this community pharmacy, send it out. I think it would be great overall, it will improve things probably a lot, but are we able to take it on board, are we able to manage it? It's got to be well... That's my view. Well evaluated first before any next steps are made, in my opinion, because you've got to pick probably... I don't know who I'm kind of addressing that to, you need to pick up a few individual pharmacies and test it well, is it feasible or not? Because you will get a backlash from pharmacies, saying, give over, give us some money for it. I mean, I'm not sure. We don't get any funds for it, we don't get any money paid for it. You see, that's another thing. The money, and again, I'm not a business man, believe me, I don't run this business, this is not my business, I just get paid a salary. But I get loads of sort of, oh no, forget it, you can't get another member of staff on, no chance. We've got this, this. This pharmacy is not profitable, this is not profitable, we're going to shut this, we're going to sell this. So we get those constraints. If we had to do some more work with no reimbursement for it, I think my bosses would be: hold on a minute, give me a break, you know, and there will be, like, people just saying, can't do it. Pharmacists have got so much work to do, the pharmacy staff in my... I'm talking about my individual pharmacy, it's got so much stuff to do, so it's got to be very carefully evaluated and there's got to be feedback collected from a different kind of parties before any decision is made. That's how I see it. But again, I'm sure it will improve. I've got no data to prove it, but again, I can see it, it will improve overall safety, but is it feasible? That's the big question mark.

HP10
R:	Is it improving care?
I:	Yes, is it improving care?
R:	I think I've already said it does. I think my answers before speak for themselves, very much so. And again, I'm not sort of...I'm a bit negative now, saying that it needs to be tested well, but again, I'm a realist as well. It's just that we can talk about our dreams but you've got to be realistic about what is, on the ground, possible and doable, what is it? But yeah, definitely listen. Any communication and the broader picture always helps, always improves overall safety of patients, most definitely. But I think maybe if... The rollout of the service, a little fee will be attached and I'm sure... As an example, I don't know what else to say. As an example now, it will be probably seen as a positive move that, yeah, we're doing an extra bit of work but we're getting something and paid for it. So like PharmaOutcomes facilitate this for us now.

HP16
R:	No, when we first scoped, even when we were talking about going live we talked about other patient cohorts it could go to, other messages that could be on there, other ticks boxes, but I think as a group they felt that they wanted it to be as simple as possible initially and then we could expand. But there's so many things we deal with, people who are new heart attack patients who need counselling on all the new medicines, they may have gone from zero to five medicines overnight and they need some significant support. There could be help intervention messages that could be done there, flu messaging, smoking, all those things they've done, they could have had smoking cessation initiated at the hospital that they need to continue in the community. Who's going to provide that otherwise, where are they going to go for it? They're going to come to the pharmacy where there's a patient service for it. And then the other part of it also is fluid restriction patients, patients from oral to liquid, can we get the liquids, what's the plan for them, are they going to be on long term, short term, patients who've started on a thickener possibly because of dysphasia. Does that impact on their other medicines because they might come in and tell us but we didn't know because you're on a monthly medicine, you've just had it three days before you've gone into hospital and you've been sent out with a load of stuff from hospital? So I don't find out that information until four weeks later when the next repeat's due and then it's all...those patients actually are all primed for information.

HP2
R:	I think you've got peace of mind knowing once that discharge summary has been written and is sent, you know, the chemist is going to…it's going to receive documentation. Because if we…we had failed in the pharmacy previously by faxing everything, then I think that would make me feel that was my fault.
I:	Right. Right. Yeah.
R:	Do you know what I mean? So, with e-referral you know it's going to get there.
I:	So, it's more…a confidence.
R:	So, it's more of a confidence, yeah, beforehand if there was…if somebody even forgot to put a cover note on the top of the prescription to say, please fax, that may at some stage not have gone to the chemist.
I:	Right.
R:	It may never have got there.
I:	So, the previous system was one which involved paper and…
R:	Paperwork and human error. Yeah.
I:	And now it's just…
R:	And now it's electronic it goes…
I:	Goes to the computer terminal.
R:	Yeah. Click a button and it's gone.

HP2
I:	So, it might change the medicine, or it might change the dose?
R:	They might change the dose. So, still we still have to do a new blister pack. But then once the patient goes home, the blood pressure might go back and therefore the GP might change that blister pack again…might change the dose again. So, it's a little bit of to-ing and fro-ing.
I:	Because once they are out of hospital, presumably, I mean, tell me if I'm wrong, and they're back into home and perhaps, more of a routine and they're eating differently, that might have an impact on things.
R:	Yeah. That might have an effect. Yeah. But there are changes but sometimes, you know, when somebody comes in, we will say…a lot of older patients aren't compliant with their medication, the older patients. And they might not take them because they can't swallow them or they're having problems and so, these are the type of things now that we are starting to ask. Why aren't you taking your medication? Do you have

swallowing difficulties? And, you know, some of the most vital medication they don't take for one reason or another and that's why they are coming back in and out. Yeah.

HP3
R:	I didn't really have any expectations, to be honest. I think because I didn't really fully understand... I didn't understand what was going to happen. All I knew was...because (name) said it will just make things a lot more efficient. I remember at one of the meetings that I went to some of the other pharmacists weren't really happy about the whole e-referral situation. I think for them... But then I think it was mainly the senior pharmacists that weren't happy about it. I'm not sure if it's because they're so used to the system that we had before, but I know one of them said it's because the pharmacies will now be aware of why the patient's in and what's happened and it's too much information. But I feel like the more information the better, to be honest.
I:	Why do you think that?
R:	I feel like it's just better for them to understand why this patient's been in. If they're left in the dark, it will just be... I don't know what the word is. I think them having more information will help them with their work as much as it helps with us knowing fully what's happening in the community for the patient. So it will help them understand, okay, this patient obviously hasn't been adherent with their medication and maybe we can do something better to help them be more compliant, I guess. Because, say, it's a case of their blood sugars are ridiculously high because they haven't been taking any of their medication, if the pharmacy's not aware of that they're just going to keep doing the same thing because they think nothing's wrong.
I:	Yes, precisely.
R:	Or they could, I guess, maybe have more consultations with the patient, make them understand this is why it's important to take your medication. I think it's just a much better handover and transfer of care for the pharmacy to be aware of what's going on in secondary care.

HP3
I:	At the moment, it's just a two-way thing between the hospital and the community pharmacy, although there is notification given to some of the practice-based pharmacists. The practice-based pharmacists and the GPs aren't particularly involved in the actual sort of receiving referrals and so on. Do you think that would be a good thing, that as well as when you press that button, discharge summary or the admission notification, notification of admission, as well as all that information going to the community pharmacy, if that information automatically went to a practice-based pharmacist in the GPs, do you think that would be a good thing or do you think that would help?
R:	I think it would be a good thing, so they're aware. I'm not too sure how...like what the roles are of the practice pharmacists, but I think it would still be helpful, especially on discharge. I think it would be more helpful on discharge, probably, if they had that.
I:	[Voices overlap 0:22:26]
R:	Yeah, if they had that information.
I:	What would they be able to do with that information?
R:	So, again, I think they can follow up with counselling for the patient. If something new has been started, they can prepare, I guess, for when the patient gets back and make appointments. I think it's just really good transfer of care, pretty much.
I:	Yeah, and having that seamless thing so that, as you said before, everybody knows what's going on.
R:	Because I don't think patients will ever say... Well, I don't know why a patient wouldn't want other healthcare professionals that are looking after them not to know what is happening. Because if I were a patient, I would want everyone to know...well, all the healthcare professionals to know this is going on, because they're looking after me. I don't think there's anything that I'd want them not to know. Especially because they understand that we're healthcare professionals, we're registered, we have to abide by rules, so it's not like we're going to be talking to random people about what's going on.

HP5
So, I met up with one of the pharmacists here, the pharmacist who is heading it and I had a little chat with her about, you know, in what circumstances do I use this et cetera. And to be honest the minute I read about the e-referral I was, like, I want to do it now, if that makes sense. Because of the circumstances under which I work, obviously because I'm off site I've had a lot of challenges with chemists phoning days later to say we didn't know this patient was discharged for example. Even though I would say that I've sent the e-referral, I've not seen the fax, or maybe they sent me say several faxes that day and it was probably not the information that they have received and it's been chucked away. You know, so it's the minute I saw e-referral I thought, oh thank goodness for that. And the other thing, of course, in my head that was going on was that when it was explained that once you put an e-referral on at the point of discharge you get a discharge summary sent to the GP, but you refer, the minute you refer it is sent to the chemist. I thought thank goodness for that. 2MJ17/04/2020 18:20
R:	Yes, and you want when they get discharged, that perhaps they get discharged into the same situation or environment that they were used to. Patients are a little bit funny; you see if they get…funny things happen. If they get a blister pack with certain drugs in it, but then certain drugs are not put into it and then from the hospital side of things we put that drug that doesn't normally go in the blister pack, back into the blister pack, they'll assume it's missing and then they'll get frantic about it, and then they'll get worried about it and so on.
I:	Yes, precisely, yes.
R:	Whereas, whereas if we have the information electronically, we could probably see what the norm is and stick with what the norm is and hence that patient will not be as uneasy. Or we'd see a situation where we can explain we are going to put this in because of this, this, this, this, this, you know. So, it may work from community pharmacy if we get e-referral from them, or contact that way.

P1_C1
I:	This is interesting isn't it? So, did you said there was no apology; did anybody help put in getting that sorted?
R2:	MDS helped out, they were the dispensers.
I:	Right.
R2:	Because I said my concern is that mum's got no meds and they said, right we can get it done, are you able to come and pick them up yourself, so I said yes absolutely.
I:	Right.
R2:	But if I wasn't able to then mum would have been without…

P1_C1
I:	About your medicines, about your medicine taking, which is interesting. So, let's move on to the recent visit to (name of hospital) and talk about that. When you were in the hospital, did any medicines get changed?

R2: Yes. In fact the, my mum's on flucloxacillin because we thought there was an infection which was penicillin based.
I: Right.
R2: Unfortunately, she had a reaction to that, which didn't start 'til the second lot of medication kicked in.
I: Right.
R2: So, we were looking for the signs and symptoms on the first…which we did get the information in the pack, none of that related to what was happening to mum because nothing happened in that first prescription of five days' worth. The second lot of prescription when we went to the GP, so we were looking for the signs and symptoms but a rash appeared which wasn't part of…
I: So, this is before going into hospital.
R2: Yes. And, then we ended up going into hospital.

P1_C1
I: Yes. Have you had a review of those medicines with the local pharmacist at all? Have you ever talked to them?
R2: No. Not to the local pharmacist, no.
I: At the general practice?
R2: I questioned a couple of them with the pharmacist and that's only recently..
I: Right.
R2: …to say, can we not reduce one of the particular tablets that mum's on. The doctor had said that she was going to put them up before we went into hospital, that she was going to up her water tablet to 40mg, that never got sent through to the pharmacy, for whatever reason, there was a communication…
I: Right.
R2: It wouldn't have made any difference in all fairness, but again there was a difference in that.

P1_C1
I: That's good. So, but there is no like, can we come in and chat to you about…do you think it would be useful to have either from the practice, maybe with the pharmacist, or maybe at the chemist, do you think it would be useful to have a conversation about your medicines?
R1: It would be, yes.
R2: I think so.
R1: I think so.
R2: Because like I said, there must be certain food types that trigger things that you can have a reaction to and I only found this out the other day by speaking to my niece who has, she does suffer with epilepsy, and the medication she is on she's not allowed to eat nuts with it.
I: Right, yeah.
R2: Now you wouldn't know that.
I: Yeah, unless it was told to you.
R2: Unless it was…the only thing, like I say, grapefruit and alcohol are the only things that's on mum's blister pack.
R1: So, I like a glass of port but I can't have a drink now with having so many tablets.

P1_C1
I: Right, okay. So, and in terms of, one of the things with the e-referral service that we've been talking about, is that it was thought that actually, if the community pharmacist gets this information, they could then call the patient in and do a review of their medicines.
R2: Yeah.
I: Do you think that's something that would actually be really helpful then?
R2: I think.
I: Or would it not be helpful.
R2: In Utopia, it would be absolutely spot on, but in reality, they haven't got the time and capacity to be able to do that. But equally, like you say, there is that many leaflets that come with all meds.
I: Yes, if there was something.
R2: If there was something in the pack.
I: Some written information.
R2: And, then as the person that's taking it, they could ring, and speak to the pharmacist about what it is.
I: Yes.
R2: That would probably be a better than every patient whose having meds go and have a sit down with the pharmacist.
I: Yes.
R2: Probably that would be the status quo, if you like, or the compromise.

P2
I: No one at all? Did anyone at the hospital talk you through what your medicines were for?
R: No, it was just changed, and they just kept saying, oh we'll change this and change the dosage and see if it works, but if it isn't a heart problem, why are they treating me for a heart problem? You know with the medication.
I: Right, so you think they've given you medicines for your heart?
R: Yeah.
I: But they've also said to you, that there isn't a problem with your heart?
R: Yeah, you know, I mean, with all them papers, me daughter's read them, and they're sort of saying, well it's not your heart. But how can your heart rate go so high and then stop and that's it, and they say that it is your heart rate but then, now, with all them papers they're saying it isn't the heart but yet they're still treating me with the medication.

P2
I: Right, so ...Before you went into hospital, apart from not knowing things about why you were on these medicines, did you ever have any problems with the medicines? For instance, you know, did you used to get them on time and things like that? Did they always used to come from the pharmacist at the time?

R: Yeah, like I say, I was forgetful and that's why I had the blister pack because, you know, there's that many and I used to count them, and I used to think, I don't know whether I've taken it, and I don't…you know, I can't remember because this is what they said on Monday what happened, I've not taken me medication. But I don't remember, and I've got to make sure that…

I: So, they think you'd gone in because you hadn't taken the medicines?

R: Yeah, and now I have to remember that I've to take it, you know, so I leave it on the side now, so I can think right I've got to take that now, and I have been taking it when it says. But of course, I'm taking all that in the morning, and I'm looking and I'm thinking well, what's that one for because I haven't got a clue, and me daughter really wanted to talk to somebody about it, but nobody bothered.

I: So, no one has talked to her either about it?

R: No, nobody bothered.

P2

I: Do you think it's better having the blister pack then?

R: Yeah, because…but I just take it, I don't know what it's for, but I just take it.

I: Keep taking the tablets.

R: I sort of think well, it's up to them to make sure the doctors and the chemist, to make sure that they're giving me the right medication. I don't know what I'm taking.

I: Do you think they are…do you think…how…are you sure they are giving you the right stuff, I mean is that…or do you worry that they are giving you the right things?

R: I don't worry about it because as far as I'm concerned, they should know what I need. I don't know what I need but now…

I: So, you don't worry about that?

R: I don't worry about it, because to me, it's not my responsibility to take the right medication, its up to the doctors and the chemist to do the right thing, and I just take it and I think, oh well, and then I look at all them and I think, what are they all for. You know, I mean it's got the names on.

I: But the names don't mean much to you.

R: The names don't mean anything to me. You know, oh swallow it whole, take it in the morning, you know, so that's what I do.

P3

R: Yeah. Now, with those, I had to take…that pink one, I have to take that out.

I: Oh right.

R: Right, and I have to put these two in, and that statin, I have to break it in half.

I: So currently this has been made up by…

R: My local chemist.

I: Your chemist.

R: Yeah.

I: And this is…so currently some of these bits and pieces are wrong.

R: It's pretty much as it was before I went in hospital.

I: Oh, so they haven't made the changes yet?

R: When I left hospital, the day after I went in to the pharmacy and asked them if they got all the information and they said no, they said, we've had a phone call from the hospital pharmacy and they have told us that there's some changes, so I said, so you've not had the discharge papers, so they said no, I said, have you had the new list of medication, no, have you heard from the surgery, no. So I said, right, I'll ring them, she said, well I'll ring them and have a word. So when I rang the surgery said, we've already sorted it out with (name of pharmacy), it's all done and everything, so I thought, right, that's fine, showed (name of pharmacy) the blister pack that I'd got from the hospital and they said that we haven't had time to get you one ready, your next one, she said, so the one that we've got ready has got all your old meds on and there's no changes, so we'll have to give you that and the boxes of the additional tablets and you'll have to take the Ranexa out and break the statin in half because they'd reduced…

I: They'd reduced the dose, yeah.

R: …dose. So that's the point I'm at at the moment, and my pharmacy said…I said, well I always used to pick it up on a Thursday because my first dose was Friday morning on the new one, so now I have to pick it up on Monday because they've rehashed everything, so I have to pick my next one up next Monday and that should be up-to-date…

P3

I: You've just got boxes and packs.

R: Yeah, so what I've got to do now as I say is I've got to make the adjustments in the pack…

I: Which sort of defeats the object of the pack?

R: Exactly, yeah, yeah, yeah. So as I say, I have these two additional ones that I have to put in. The other problem is with that, one of these is a water tablet that…so that is a stronger one than the water tablet I've got, but I don't know which one it is, because I'd have to take that one out and replace it with this one. So all I can assume is – they've only given me seven days of these, so all I can assume is just take the one that's in, you know, because I could take the wrong one out.

P3

R: Yeah. I find the surgery sometimes can be a bit lax because there are so many different doctors, you never know who you're going to see when you make an appointment. I've been lucky this last period, this all started in May with the first hospital, and they changed tablets then and the day I came home I got a massive headache and I've had it since then, this headache, every day, all night, and they were tweaking the meds and doing this, that, the other and nothing helped it, Paracetamol, Co-codamol, nothing, the only thing they did was what I shouldn't take and that was Anadin because of the Aspirin content.

I: Right, yeah, you're already…

R: I have all Aspirin every day, yeah. So every time I mention it I get told off at the GPs', but it was the only thing that would take the edge off and give me a bit of peace. This last stay in hospital there was this change of tablets again and they stopped Ranexa as I call it, they stopped that because they said that could induce headaches, and anything that's angina-related normally gives me a headache. So the headaches seemed to fade a bit on this occasion, and they're not bad at the moment, I can't complain now, but that's when it all started with the medication and the changes and the ups and downs.

P3

R: But as I say, it's just with the case…I mean, when we went in last week for the blister pack she said, oh, so-and-so has just redone it, it's not been checked yet, said, okay, we'll go and have a coffee, you know, no problem. When we went back, they still hadn't checked it, so we hung around and they checked it and came back and that's when she explained then that she'd put these…

I: Extra boxes.

R: But she didn't tell me I had to take the Ranexa out, which they've stopped, you know, so…

I: How did you know you'd got to take that out, who had told you that?

R: They told me at the hospital the Ranexa was stopped, yeah.

I: Right, and they hadn't taken it out the blister pack.

R: No, because they say they've not had the information.

I: Right, so they're working on the old prescription still.

R: Yeah. But when I rang the surgery, they said they'd sorted it all out and they'll send all the new stuff to (name of pharmacy).

I: So when you collected…do you collect it weekly?

R: Yeah.

I: Yeah, so when you collect it this Thursday…

R: Well, it'll be Monday next week.

P3

R: Yeah, last Monday, late Monday I came out, yeah. So we went in on Tuesday to see them and nothing. And I asked them – when did we go in again? We went in yesterday and I said, have you had all the paperwork yet, he said no, not had anything. I said, what, not a list of the new medicine…

I: You'd finished your seven day supply that the hospital gave you…

R: Mm?

I: The ones that you got as you left hospital.

R: Yeah.

I: That's gone, isn't it?

R: That's gone, yeah, which is why I've got to physically make the changes to this, but I'm not going to use that one, the water tablet, because I can't identify…

I: Which one it is.

R: …which one it is.

I: You're supposed to be taking, yeah…

R: So I'll leave that and just work on that one till they change it.

P4_C4_C5

R2: When he comes out now, I phone (name of pharmacist), that's our pharmacist, and I ask her if she's got the prescription, and she'll say, no, there's nothing come through.

I: Right, okay.

R2: So, then I phoned the doctor. Oh, we've got nothing. I did it this time, when he came out on the Friday I phoned (name of pharmacist), because…

R3: That was after speaking with (name of pharmacist) wasn't it?

R2: Yeah, 'cause she said it goes from here, the day he comes out.

I: Yes, I mean it should be, yes.

R2: So, I phoned her, on the [Saturday 00:08:40] I phoned her, I said, right, there should be a prescription for Monday comes through, or it's going to the doctor, and you should get it Tuesday.

I: Yes.

R2: So, I phoned, she said, well, I've got nothing, so I phoned on the Tuesday, she'd still heard nothing.

I: Right.

R2: So, I phoned the doctor, but you get the receptionist, oh, there's nothing here.

P4_C4_C5

I: Yeah, I know which one it is. So the pharmacist should have known what the medicines were, but it's about getting the prescription from the doctors.

R2: The doctor, that's right, she said, I can't do anything.

I: They've actually got to write the script haven't they?

R3: Yeah. I have, in the past, had to go into the doctor with his discharge notes.

I: His discharge summary, yeah?

R3: Yeah, over a week past him being discharged.

I: Yeah, because you come out with a week's supply, and then when that's gone…

R1: We got some this morning.

P4_C4_C5

I: So, did they get sorted, those problems with those medicines?

R2: Well, I phoned and she said, what was it, I'm sorry there's nothing come through. I said, well I'll tell you what, I said, it better be through for Thursday, I said, or my husband's got no tablets.

I: Yeah.

R2: So she said, well just a minute. Oh yeah, they've found it.

I: Yeah.

R2: They've never passed it through. And I know he takes his time for signing them off.

R3: There has been one occasion, where I have been chasing a prescription from morning, that should have gone through to him, and I've waited and waited, to the point where I've gone down to the…I've phoned all morning, oh, he's in clinic now, he'll do it when he's finished clinic.

R1:	Yeah.
R3:	And I've gone into the surgery, and asked for him. Oh, he's on his lunch. And I've waited in the surgery, and he's walked through and I've gone, is that the doctor? Doctor, I forget his name now, but at the time I went, Dr (name) And he turned round, and he's going, and he's walking, and I went, Dr (name). And he's turned round, and I said, it's my…(name of patient) daughter, I said, have you got his prescription please, I said, only he's desperate for it? And he's looked at the girls and he's gone, oh has it come through yet? I said, yes, it come through at such a time this morning, and I said, he's urgent for it, the girls passed it through to you at such a time today. And he's just walked off, gone in his room and then sent it, I'm sat there, and he's sent it through to the pharmacy, and then the girls have said, oh, he's sent it through to the pharmacy…
R2:	Won't come out.
R3:	..cause I was like that, I thought five more minutes, and I'm going to knock on his door, because I think they just…you was brought up with respect, my Mam and Dad always brought me up with respect, and now I have a few friends who are GPs and pharmacists and stuff like that, and they're ordinary people, but some of them get this chip on their shoulder, and you think, no, you treat people with respect. And to me, that GP, he's just taken on too much and can't cope, but he won't pay for the added locums and the extra staff to take on the workload he's got, and he's there for a service not to make money. If he's in that, he's in the wrong job.
I:	Yeah, yeah.
R3:	He really is. The run ins we've had with him, I'm sorry, that is why…

P4_C4_C5
I:	Has there been any problems there?
R3:	There's certain items that you've required, that you've struggled with, 'cause they go through Clinic Direct.
R2:	Oh yeah, and they take longer.
R3:	And that is hectic, trying to get, 'cause you'll ring Clinic Direct, and they need the prescription, the doctor won't release it.
R2:	They phone the doctor, the doctor sends it to them, then they can release it.
R3:	But my mam, you spend a lot of time don't you, trying to chase round for stuff that he needs.

P4_C4_C5
R2:	Most of it's the same, so I can look at this list, what I've got, and it'll tell me what they're called
R1:	I think, the old saying is (name) could give them a run for their money. As of, identifying tablets, they haven't come and told us what they are, this, that and the other, and she's had the pack from my old pack, and she's looked at them, and she's said, they've changed that.
R2:	And what about when they left one out?
R1:	And they left one out.
I:	Right, so what happened when they left one out, what was that, was that recent or was that…?
R2:	No, it's a bit ago.
I:	A bit ago?
R1:	You went chasing it up didn't you?
R2:	I went chasing it, and said to (name of pharmacist), how come there's none in this?
I:	So, there was something wrong with the actual prescrip…it was one not in there?
R2:	Yeah, she said if you ever see that, you must phone me, she said, you must phone me right away.
I:	Yeah.
R2:	And when he comes out of hospital, she says, phone me and let me know he's out.

P4_C4_C5
R3:	So we was here, and giving him the packs, I was looking at the, you have the pack, when you used to get it, you had the pack, and it identified or try to identify the tablet, and I noticed on what (name of community pharmacist) had handwritten, it didn't always fill out what shape and style the tablet was. So, in some instances, it was difficult to pick out what that tablet was.
I:	Right.
R3:	So, where you've got quite a few in the blister, you'd have a white one, or a white oval, and there might be two white oval ones in there, which one is large oval, small…?
R2:	[inaudible 00:29:59].
I:	Do you…is it useful having the blister pack, do you think?
R2:	Oh yeah.
I:	Does that help?
R2:	Yeah.
I:	In what way does that help?
R2:	Well, I'd be opening boxes all the time.
I:	Yeah, because it's all organised, it's…four time a day isn't it?
R2:	Yeah, what's not in there, his is for his moods.
I:	Right.
R2:	I've got that in a separate, and…
I:	Why's that separate, is it recent or?
R2:	It's 'cause they changed it.
I:	They've changed it.
R2:	And they didn't put it in the first week.
I:	Right.
R1:	It's not got in the system yet.
I:	Yeah.
R2:	So, it's in the system now, and she's sent it, before we got it off the daughter, when we went.
I:	Right, yeah.
R2:	Now I've got two lots.
I:	Oh right.
R3:	Yeah, for my mam to stand there every morning, sorting them out, or every week, it would be an absolute nightmare.

P4_C4_C5
R3: That is when Alexa comes in really good isn't it?
R2: Yeah.
I: Oh really? Right.
R2: Yeah.
R3: Oh yeah.
I: Tell me about that.
R1: Alexa, aye.
R2: Echo.
R3: Echo.
I: Echo?
R1: She'll talk to you, so watch out.
R3: Have you got it switched on?
R2: You've turned it off (name of patient)
R3: Yeah, we've had Echo, yeah.
R1: It'll give you the time of day and remind you what you're doing.
R3: It my mam has to nip out, like for five, ten minutes, well I say five, ten minutes, she might nip over and see my sister, or whatever, and if it falls in line with him needing his tablets, she'll put them there for him, and, I'll be back whatever, and she'll get Echo to shout, (name of patient), take your tablets.

5.2 Code - Good communication leads to timeliness and promotes good relationships

HP12
R: Which I think is quite a safety issue. So, we've got a lot more time to sort everything out. We've got time to sort the prescriptions with the surgery; they've got time to look at it properly, to work out everything. So, that's worked much better. And we're not bothering the surgery saying; please can you print me out a discharge summary.
I: Yeah, because you've already got it.
R: It sounds awful to say but if the surgery just do us a new lot of prescriptions, we prefer to check that against the discharge summary, just to make sure that everything is as it is on the discharge summary. I know the doctors have issued the prescription but we prefer, as a double check, to check it against the discharge summary. So, sometimes they were getting a bit…why don't you get one and blah blah…so, we've got that information and like I say, it's the other way round now, we are taking it through to them saying, this patient has been discharged, here is the discharge summary, please can we have prescriptions.

HP13
I: Well, yeah, precisely, whereas with the fax, a piece of paper, no one knows what you've done.
R: And I think…as far as I understand it, that feedback that I do, saying that we need another prescription or whatever, all that gets emailed straight through to the GP so they've also got another record, you see, at their end…I Right, that's interesting…R …which is good because if I had to ask them for a prescription and they're just not playing ball and they're not providing it, it's, there in a record and they can see that I have asked. They can't say three days later you've not asked us for a prescription. I can say, well it's logged when I phoned you on da da da and they have the email of that.
I: So they get emailed from that?
R: Yeah, and I do them promptly, I do them if…well, before the PharmAlarm thing I might've done it…it might've been the next day but now we do them more or less as soon as we get them.

HP17
I: Yes, and I'm thinking in terms of workload that has an impact on…
R: Yeah, it…and it delays things, and then you could just a patient turn up saying, I've got no medication. And so, well no one's told us you've come out. See, that's another issue. So, for me an action would be to involve nursing homes on this scheme as well. So, you've got the hospital, you've got the surgery being notified, you've got the chemist being notified. What you've not got is the middle man, which is…so there's the hospital, there's the respite people, whether it's a nursing home, whether it's some temporary NHS centre that's linked to the hospital that they might be in for a week/two weeks/three weeks, and then they go back into the house. So, you're jumping from the hospital and you're missing that and you're jumping straight to the residential, but they need to be involved on the scheme as well.

HP4
I: And who are the important people in using that, who are the important people in that?
R: Well, everyone I guess, because it's like a chain, isn't it, I guess, it's like if one part is not working and the rest isn't, so if you don't put the referral in the first place which would be the responsibility of whoever is doing that medicines reconciliation, so that would be the pharmacist or the technician, if you don't do the referral in the first place, community pharmacy is not going to know they're in hospital. But if they don't pick it up, if they're not proactive in picking up the referrals, then it's not going to work either. So I would say probably equally important.

HP5
Basically, what it does it strengthens the relationship between all the carers whether they do primary or secondary or whatever, between the community and the hospital. And for me personally I do feel that patients are getting better care as a result or continuity of care.

HP9
I: Yeah, and that was one of the other things that people were talking about last year with the service, was that this would…the potential for this to lead to patients better-informed about the medicines. Do you think that's likely?
R: Yeah. I think so. I think that's the potential with it, yeah, 'cause again, when you say, rolling it out to all patients, because there's people not on a Venalink but are on ten medications and something would have changed in hospital but then if nobody tells them about it then they don't know. They might have a box of Ramipril at home and it's been stopped at the hospital and if someone briefly mentioned that to them or not

mentioned it at all then they'll go home and start taking that again. And if the chemist is not informed of that then they might have a box on the shelf waiting for them that repeat prescription or they'll just give them that and then this will just carry on. It's like a – what's it called – the Swiss cheese and when it's like an error, just yeah….
I: Just going through the hole, yeah.
R: …gets passed, yeah…
I: Precisely.
R: …basically. Yeah, and this is a potential…if something stopped they could tell them, this has stopped, they can maybe take it out of the bag and not give them that obviously medication…

5.3. Code - Potential benefits - Capacity to change, capacity to improve care

HP1
I: So, we'll start off really with some of the background to the Pharmacy Referral service. Why do you think it was introduced?
R: So I attended some of the initial meetings, with, I think yourself and (name) So, it's to improve the communication across three, initially, it was the three sectors, community, practice and hospital. And obviously, the prime goal is to reduce hospital admission, via reducing errors and that could be prevented by communication amongst ourselves.
I: Yeah, to join that communication up, yeah?
R: Yeah, so just simple communication between, maybe the community pharmacy and the practice, will then reduce an error, minimise an error and, yeah.

HP1
I: Yeah. I mean in terms of sustainable, I mean is this sustainable?
R: I don't think so, no. I mean, when I get a notification I think, oh, not again. And especially if the actual, and this is just assuming that the issue was something important, and that we needed to change immediately. This particular example was resolved already, so I kind of did all the work, and logged on, and called the community pharmacy, and they said, oh no, it's fine, it's done.
I: But there ought to be something to say to you that that's actioned, done.
R: Yeah, well I can't see anything that said actioned, there, but I don't know again, how it's used in primary care, so it may be if I understood how they do it, then I'd know that a particular word means that it's fine.

HP1
I: Do you think, sort of moving on a little bit, do you think this is something which has potential value or benefit going forward, the service as a whole?
R: Yeah, I think so definitely. As a three way communication, and highlighting important changes that really will affect the patient, that will harm the patient if it hadn't been changed, or communicated, I think definitely, and I'm positive to change, I'm not usually negative about something, I think this is great, and it's definitely something that's needed, I think in time it will be more slick and I think, once we've used it, and everyone knows how to deal with it, then definitely it's very positive, yeah, definitely a move forward.

HP1
I: Do you think the potential impact upon patients, or the potential to improve care, has that been realised yet, or do you think…?
R: Yeah, I think it's more, rather than having an impact on the patient, I think the benefit is the communication. I think that's what's great, and kind of having that three way communication, and knowing that an issue is going to be tackled in three of the different…
I: Yeah, because that has a time saving thing, or it makes work easier or more streamlined?
R: Yeah, makes work easier yeah, definitely more streamlined. So, I think that's the benefit. I don't think, and it changes the way we work, and that's what's going to benefit the patient. I don't think particularly specific, I don't know where I'm getting at this but…

HP10.
I: That's something, isn't it? Because one of the things I think that was said to me way back, sort of last year sometime, around community pharmacy, and I know that there were people really pushing this agenda forward and saying, well, actually, this is going to give value to community pharmacy. Do you think it has that potential to do that?
R: Most definitely. Honestly, most definitely. We, as a pharmacist, believe me, we do, apart from making accuracy checks, we clinically check things. And if you're clinical checking, it's not only about interactions and side-effects of meds, it's about the broader picture, so you're looking at a number of things. As a clinical check, you're making sure that an elderly lady, can she open it, can she apply that drop, has she had any falls, what's her history? When I do a Medicine Use Review, I will ask them, let them speak for a minute. I say, how are you doing, how are things, how are you managing your meds, how is your life to do with your meds? I listen to them, so that gives you an understanding and picture and I always make myself notes, so, oh, this is this patient. If you try... I always call it I'm trying to do a proper job today. So if you do a proper job, that's how it should be, so as much information as you get, you're able to apply the information later in order to improve the overall safety of patients, and that's it.

HP10
R: Yes, you're right. You're right, yes, it does. I'm not sure whether you are trying to refer to the fact that I've just said about communication with the patient.
I: Yeah.
R: That's what you meant. Yeah, that's right. True, I didn't think about that initially, it just came out in the conversation. Yeah, you're right. It's kind of like you're looking at it, when you're dealing with that on PharmaOutcomes it says, what have you done? Commenced a blister pack, phone them post discharge, MUR, NMS, this, that and the other. So you're thinking, oh, maybe I should do that, you know, like even that or just... It's just basic communication and those people often, you know, they don't... You're not trying to patronise them in any way, but where I work, there are a lot of working class people, so they're just people that don't necessarily understand things very well but they appreciate, like, you know, like you look after them, you tell them, you... To be honest with you, if you ring them, give them a ring and reassure them, you save yourself phone calls later, oh, where's this, where's that? It's just you do it and you know everybody is happy, it's much better. And again, you know, as I said to you, maybe it's me, but I always enjoy doing clinical stuff, I always like to kind of have that broader picture overall. Communication makes them safe and makes the drugs they use safer, in my view.

HP10

R: Is it improving care?

I: Yes, is it improving care?

R: I think I've already said it does. I think my answers before speak for themselves, very much so. And again, I'm not sort of...I'm a bit negative now, saying that it needs to be tested well, but again, I'm a realist as well. It's just that we can talk about our dreams but you've got to be realistic about what is, on the ground, possible and doable, what is it? But yeah, definitely listen. Any communication and the broader picture always helps, always improves overall safety of patients, most definitely. But I think maybe if... The rollout of the service, a little fee will be attached and I'm sure... As an example, I don't know what else to say. As an example now, it will be probably seen as a positive move that, yeah, we're doing an extra bit of work but we're getting something and paid for it. So like PharmaOutcomes facilitate this for us now.

HP11

I: Yeah, that's fine, yeah, absolutely. No, that's perfect. So thinking about the pharmacy referral service, why do you think it was introduced? What do you think was the point of it, if you like?

R: Well, I think it's always been particularly good in (name of place) anyway because they've always let community pharmacists know directly, which a lot of places don't do. So (name of hospital) has always been a bit ahead of its time with that. And then they've always communicated directly with the GPs, where I know, like my in-laws live in the Midlands and they get a scrappy piece of paper when they come out of hospital, to take themselves to the GP. Presumably they then get a letter at some point but there isn't the direct contact that they have in (name of place). But obviously, before they were just faxing it, it's a bit hit and miss and sometimes they'd fax our phone number rather than our fax number and it's not secure, is it?

HP12

I: Do you think the service has, actually or potentially benefitted your work then?

R: Yeah, definitely. I know there has been a couple of occasions previously where we've had discharge prescriptions from the surgery, we haven't actually seen the discharge summary, we've just been told, right this patient is out of hospital this is the new scripts, done them, sent them out to the patient and the patient says, I've not got everything here, I was on a lot more medication and then you'll look at the discharge summary and half of the stuff hasn't been done for some reason. Whereas now there is that extra check because we are seeing the discharge summary, the doctor is seeing the discharge summary, the patient and family, depending on how able they are to deal with it. But they can look at it and say, I usually have eight things in a morning, I've only got three, what's going on, sort of thing. They might not know what they are but they know that it doesn't look right. But now we've got that extra step in that we can check that what's happening is right.

HP12

R: From a safety point of view that we are knowing that they are getting what they should be getting on the discharge summary. From a safety point of view in terms of not delivering stuff when the patient is in hospital, from the fact that there's hopefully no missed days of medication because we didn't know they were out of hospital, we didn't know there were changes, you know, so hopefully there is no missed days of medication. So, yeah, I think it's definitely improving.

HP15

I: Right. Why is that useful to know why they've been in?

R: It's just so we know exactly what actually was happening with them and then…it gets us a better understanding of the medications as well. […] So, we know why it was stopped…why something was stopped, reduced or increased or…it gives us a better understanding.

HP16

R: I think there's multiple impact really, for the patient it's seamless care, which is the aim of the project, it's about seamless transfer of care between the two sectors. For a patient that's what they need, they want to see the medicines on time, they want to see that actually they've not had to chase things up, they're not worried, they're not anxious about getting their medicines. Those sorts of things, actually will they come on time, did you know I was in hospital, did you know I've come out, did you know this has changed? All these things are avoided. But actually, from a GP perspective they know that we're getting the same information now so actually we're not phoning up, our phone calls are not, have you got a copy of the discharge, has there been any changes? Those questions which are more, I would say, logistical, we're moving to clinical conversations, we're actually doing the technical conversation rather than just all the admin stuff that actually this sorts out because we've got the information there. It's what do you do with the information rather than actually have we got the information in the first place? And for us as a community pharmacy it means we have a deeper impact on the patient. Now that might not be for every patient, it might be for every patient in a different way but we are making an impact on that patient's care. And that's the value for us. And then if you talk about the system, if we can reduce readmission, if we can make every contact count, if we can make sure that that appropriate care is there with the appropriate practitioner, all these things are...we're the experts in medicines so actually we should be dealing with the medicine side for the patient. But, ideally, for us we should be remunerated for spending that time with the patient in whichever way that is. And for us it's about making those meaningful impacts and then the system benefits from all those things, reduced waste, reduced medicines burden, actually having expert patients with the right things, safety improves, readmissions could reduce. The benefits are there.

HP17

R: So, how many patients have I personally prevented from dying, being re-hospitalised by GP prescribing errors before versus after the new electronic notification system? So, I mean I'm getting old now, I've got some [inaudible 00:00:28]…I don't need to tell you that, but I've been doing it 15/16 years but… Yeah, so getting the full information is great, it reduces prescribing errors.

I: Because you have more information to check for the GP?

R: Yeah. So, something might have been stopped but because the GP's maybe not noticed or was rushing and he's stressed in his job, or her job, and they've not noticed that this has been stopped, or that's been started. So, you could get multiple…that one that I just told you about was two diuretics together. You could get all sorts of different changes, it could be anything, and you could get something stopped that the GP's still continuing. So, anything I notice that doesn't match the discharge sheet I'll query. But my dispenser here, when I came, she was a bit reluctant to query and go against the GP and say, well why's this on, or, why's that not on, because once the GP went mad at her and that then changed her attitude. So, he said…this GP said, 'why are you questioning me, I'm the GP, I prescribe this?' Well it's not on there.

HP17

No, that's interesting and that sort of like leads onwards to sort of other questions I've been asking people, which is around sort of like what…where you see the benefits of this, you know, and there're various strands we can talk about there, one of which is obviously what's the benefit to patients?

R: So, based on the patient the other day the benefit to that patient is that they're still alive, they've not been…

I: This is the one that had the two diuretics?

R: The one…the two…double diuretic dose. So, one, they're still alive, two, they've not been readmitted to hospital, three, they've not taken a double dose of a diuretic, or it could be all sorts of queries over the years, it depends how far back you go. If you're just talking about now we've got the new system in place, or the worst…

I: Yeah, with the new system.

R: …case scenario incidents that I've seen in my history.

I: Yeah. No, now with the new system what…how much is that…the new system, how much of that…

R: Reduced.

I: …is helping, you know, yeah…

R: So, you…I'd say constantly.

I: …patient impact?

R: So, you've got the one from two days ago, two or three days ago. That patient has avoided a major incident by me having the discharge sheet and by me reading the discharge sheet in detail.

I: Is that…that's the one with the two diuretics?

R: Yeah, that's what I'm talking about, yeah.

HP2

I: What would the benefits then of having that three-way communication be? In terms of…

R: Just the time. Everybody is on the same page at the same time. So, that's why.

I: So, it's clear what that patient…

R: It's clear what that patient's on and what they should be doing. Yeah.

I: Yeah. Yeah. Yeah. Precisely.

R: And if the patient cannot…if the patient is confused or not taking their medications as they should, then maybe the GP should get involved and more and find out why.

HP2

I: And do you think it should be…is there a potential to extend it beyond just MDS or beyond dosette boxes, beyond Venalinks to discharge more generally.

R: Every patient? I think it would be a really good idea. Yeah. For us it would be a really good idea just to let the chemist know when a particular patient is in. But for the chemists it will be…basically we are passing our work on to them. And I've had this discussion with a particular pharmacy here in (place), who said to me, I don't like this system at all. So, I said, why? It's great, do you know, she said, yes, but you've passed your duties on to us. Because now they have to go to the computer every morning to look, to see if they've got an e-referral…a referral and it will say, oh, we're coming to have a look to see who's in hospital. Oh, right, sort all their…what we're going to do there…blister packs today, no, so should we do that. She said, but then all through the day, we are constantly looking. So, it's taking time out for our staff to keep looking to see if there's been any referral to say somebody is in. So, we don't have to do the blister pack. Whereas beforehand, we were waiting, until the end of the day to fax them, they got hard copies, so, they didn't have to keep going looking at the computer. So, they were relying on the paper appearing on a fax, now they have to keep going to look at the computer, to logon, to put their code in or their password. So, it's causing them more time.

HP2

I: You talked earlier about the potential for, you know, discrepancies between the GP list and the pharmacist's list and the patient…what the patient thinks they are on, you know, which, you know, can often happen. Do you think this can help?

R: I'd hope so. The thing is with the patients, they'll come in with a particular blister pack and we'll say, you can't use that one, it's been changed. We will provide them with a new one, which we do for a week, but then they may go home and they have a blister pack lying there from a couple of weeks ago…

I: Yes, they got a box.

R: …and carry on using that. So, there's got to be…somehow, we've got to get through to that patient, where if it is not in hospital, it has got to be in primary care, where they are up to with the medications. And I think if we are all on the ball, the chemists, the doctors and the pharmacists here…the hospital pharmacy here, we can bet that patient can benefit, but they…I don't know.

I: Yeah. Because, yeah, what you are saying is that that if you change the Venalink and they end up…they go home and they've got a different one or there's…

R: They've got a different one at home, they are going to carry on taking the other one until that one is finished.

I: Or the GP isn't aware that things have been changed.

R: The GP isn't aware that things are changed, so they will do in three weeks' time, you know, when the letter comes.

I: Yeah, when the letter comes through.

R: But like with the e-referral, the chemist should know straight away. Well, they'll get to know straight away and should prepare that next time.

HP3

R: I didn't really have any expectations, to be honest. I think because I didn't really fully understand... I didn't understand what was going to happen. All I knew was...because (name) said it will just make things a lot more efficient. I remember at one of the meetings that I went to some of the other pharmacists weren't really happy about the whole e-referral situation. I think for them... But then I think it was mainly the senior pharmacists that weren't happy about it. I'm not sure if it's because they're so used to the system that we had before, but I know one of them said it's because the pharmacies will now be aware of why the patient's in and what's happened and it's too much information. But I feel like the more information the better, to be honest.

I: Why do you think that?

R: I feel like it's just better for them to understand why this patient's been in. If they're left in the dark, it will just be... I don't know what the word is. I think them having more information will help them with their work as much as it helps with us knowing fully what's happening in the community for the patient. So it will help them understand, okay, this patient obviously hasn't been adherent with their medication and maybe we can do something better to help them be more compliant, I guess. Because, say, it's a case of their blood sugars are ridiculously high because they haven't been taking any of their medication, if the pharmacy's not aware of that they're just going to keep doing the same thing because they think nothing's wrong.
I: Yes, precisely.
R: Or they could, I guess, maybe have more consultations with the patient, make them understand this is why it's important to take your medication. I think it's just a much better handover and transfer of care for the pharmacy to be aware of what's going on in secondary care

HP3
I: Yeah, people knowing that you've got a community pharmacist knows what this patient's on, or a practice-based pharmacist or a GP, a hospital pharmacist, all these people know what medicines the patient is taking, and where does that have a benefit for the patient?
R: In terms of, I guess, like continuity, so if something has been stopped here and the community pharmacy doesn't know, because I've seen this happen before, they will just continue to put the same things back in the blister pack. The patient will continue to take it, not aware that it's back in there, and they'll end up back here because they were taking something that they shouldn't have been taking. Whereas if the community pharmacy is aware, the GP practice pharmacist is aware, I guess it's kind of like the Swiss cheese model, things will be picked up, so there are no errors that will fall through, it will all be picked up by people that are aware of the situation.
I: Yes, so you've got more...
R: Barriers to it, yeah.

HP3
I: And especially, as you say, if you don't particularly know. And I suppose the final thing to finish up now is, do you think this can/will/will not improve care?
R: I think it will improve care.
I: In what ways?
R: I think just moving more towards... I feel like just overall we're moving more towards more technology. I love the fact that this is an electronic prescribing hospital. I think the paper charts are old, I think faxing is old. I prefer... I think it's just more efficient and more convenient to have things like e-referral, have things like emailing and just sending things electronically. So I think it's just progressive, pretty much.

HP4
I: Yeah, right. And who do you think should be using it, should it be restricted to just community pharmacy and hospital pharmacy or are there wider people or do you think that's about right?
R: To be honest, I'd not thought about…do you mean in terms of like GP practices and things like that?
I: Yeah, yeah.
R: We have a lot of…so it's kind of really exploding, the whole community interface at the moment with all our practice-based pharmacists, NIPPS pharmacists, it's really taken off into this new area which we'd not really delved into before. It's quite interesting to think how it could be used. In some ways actually if you had a practice-based pharmacist thinking about it, it would be really useful for them to know that that patient's in hospital, but they would be notified on discharge anyway I guess because they would get the discharge summary, wouldn't they, so I'm not sure how it would work in…currently we just use it for blister pack, for Venalink patients, don't we?
I: Yes, yeah, precisely.
R: So I don't know if it would have any particular benefits, I'm not sure. I think for what it's used for, I think the right people kind of are involved. It's quite a specific thing we use it for currently.

HP4
I: Yeah. So those challenges, that's having the blister pack, but the e-referral, how is that going to get over some of those challenges?
R: I think like we said, it just makes it more reliable, that that communication is going to be actioned and quicker, so when a patient goes home, I can kind of assume that that e-referral is going to get picked up, the community pharmacy can see straight away that aspirin stopped or whatever. They can then prompt the GP to make the changes more quickly, it just speeds everything up, it makes it more reliable that they're going to then get the correct medication continued post-discharge I think.

HP4
I: And we were talking about MDS and just the thing I wanted to come back to and about Venalinks and blister packs, MDS, compliance aids, whatever you want to call it, currently that's the only group of patients that the service is being used…is focusing on. Do you think it should focus just on that or do you think it could be used for discharges more generally?
R: Yeah, it's interesting, isn't it, because that section of the discharge summary is so poorly documented at the moment in terms of stop-start, change medication, I actually think it is also really useful sometimes just to notify the community pharmacy of changes generally. Because you've still got the same situation, imagine you've got your 80-odd year old male who's on lots of medication, we change everything in hospital, then we rely on the GP stopping that prescription say for the aspirin, you know, once they get the discharge summary, then say a secretary looks at it then passes it on to the GP, the GP then puts it in their pile of things to look through. A couple of days later eventually, maybe they'll get round to stopping the aspirin off the repeat prescription, but by that point, say like another lot of prescriptions has gone out or they've got one at the chemist, by the time the chemist gets the next lot of prescriptions, they could easily have got another supply of medication stopped. And if the patient's not totally with it with their medication, which lots of people aren't, they'll just take whatever arrives, then it definitely would, you know, you can see how it could work there as well. I sometimes have used it for stuff like that, so I know technically we're not meant to, but I have used it a couple of times for patients who aren't blister pack patients, and I've used it for more like a communication tool almost. So I think the two I use it for are for nursing home patients, and I wasn't sure to begin with whether they had blister packs or not, I can't remember which nursing home it was, but I rang the chemist just to check and they said, they're not blister packs, but they said to us, if you make any changes, would you let us know because they're due another month's supply of medication to go out this week. And I was like, yeah, that's fine, so I put a referral on and I put like in the comments section, and b) this patient does not have blister packs, but this is just to highlight any changes on discharge. So in that situation, I've already kind of used it for that to some extent, because I don't see why you couldn't.

HP4

I: And do you think it has the potential to improve care or…?

R: Yeah, definitely, yeah, I just think the whole kind of…just making sure those medications are out quicker impacts on patient care, doesn't it, it's patient safety, it's reducing adverse effects from medication, making sure they don't have things that we've stopped in hospital, it can only improve things really from a patient safety perspective.

I: And hoping they don't come back in.

R: Yeah, yeah, that's right, yeah – well, yeah, longer-term, or thinking about it, we have so many readmissions because of medication errors, it could hopefully help to reduce readmissions.

HP5

I: Right, okay. And what do you know about the pharmacy e-referral service that's been introduced here, what do you think it's there for?

R: Well, I'll tell you what, I was very excited when I first heard of e-referrals. Only because within the unit where I work, the units, if a patient is discharged or if they arrive, one, is I don't know if they've been contacted by the hospital prior to transfer to my facility, that's the first thing. That information is not necessarily in the medical history, the medicines history or medicines reconciliation, that's the first one. And the second thing is, is because I work between units my contact with the community pharmacy that if they had a blister pack, for example, would be via fax, via fax. But quite often they would get discharged and I wouldn't be at that facility and I missed the opportunity to actually inform the community pharmacy of what the patient has actually been discharged with. So, the beauty of the e-referral, I'm not being funny, but the beauty of it, is that firstly we get to inform the community pharmacy that the patient is admitted into hospital, they get to know that electronically. And the second thing is whether or not I'm at the facility, once an e-referral has been setup they get a discharge summary once the patient goes. So, it takes the pressure off of me, trying to remember whether or not we've informed community pharmacists, et cetera. I've got a visualisation, I can see now whether or not a patient has had an e-referral, and I know that's going to go once they get discharged, so that takes the pressure off of me, I love it, I absolutely love it.

HP5

I: Yes, that's really interesting actually and you talk there a lot about that continuity of care, because of actually one…and you also talked there about the…in terms of that, in terms of patients because one of the questions there is, you know, do you think the service can improve care, will it improve care?

R: Yes, definitely.

I: In what, why will it do that?

R: Because the information received is accurate. It really is accurate; everybody is getting the same information.

I: Yes, precisely.

R: And you've not got a break in information. Because a patient, a scenario we've had in the past is a patient, we might send a summary to a patient, they might go to community pharmacy to get a supply of the drugs, community pharmacy isn't aware they've been sent into the hospital and gives them something, a new blister pack. Perhaps they might have had a couple of prescriptions left from prior to the admission and then they get drugs that have been, that has been stopped that caused hospital admission.

I: And could have caused a hospital admission.

R: Exactly, so yes, so definitely because there is communication between all services involved and you've got now exactly the same information moving between all of them. The risk of having errors like that made is minimised.

HP5

R: Less, you have less, right you get less admissions, hospital admissions due to drugs for a start. Patient educated as well, patient education, they'll know exactly what they are supposed to be taking now because they get the same information from all the disciplines involved. Patients can get confused if they get one information from here and another set of information from here, so it's less confusion with regards to their care. And they are less, obviously they are less distraught and traumatised by people not talking to each other.

I: No, knowing what that…yes.

R: About what they should be getting.

HP6

R1: I think it's a great, great thing, it is very, very fruitful and a very efficient system because if you compare it with what I used to do, I still do that thing but actually I used to do faxing, we used to fax the sheets, the prescriptions to the pharmacists. I used to spend at least an hour and a half every day, on faxing.

I: Just standing by a fax machine?

R1: Yes, standing by the fax machine, waiting for reports and that time that I was spending on faxing, should be spent on returning, on saving money. So we are wasting time and wasting money. So, I used to average, let's say two hours and now it's no more than 15 minutes, average.

HP7

I: Looking more broadly then at benefits, what about patients.

R: Yeah, I guess patients, as in it been better, you know, like logistically deliveries and things like that. So, they don't get failed deliveries and then on discharge things have had to be sorted. Like we've had to redo one, something like that. So, I guess it's just a bit better in that sort of way and their information has just been, you know, it's just a bit more safer for patients.

I: Because?

R: Because there is better communication in the community.

I: Yeah.

R: So, the community pharmacies will have more of an idea of when they are being discharged, when they are admitted, what medicines they are on at the moment, things can change quite a lot in hospital. Especially if patients are in for quite some time. So, yeah.

HP8

I: Yes. So basically, as a patient they are referred, there is this referral, it's a referral for the patient to go and visit the community pharmacy rather than just notification as it is.

R: No, I do think that would be a good way to relieve maybe some of the pressure on GP practices in primary care. I think they are trying to get the message out there that you can see your pharmacist for advice on many, sort of, minor ailments and conditions. And that actually, you

know, you don't just automatically book an appointment in with your GP. I think the trouble would be is where people have more serious long-term conditions, maybe they've got COPD, they feel, like, they are having an exacerbation, obviously don't go to (name of pharmacy) about that. So, yeah, I think it's a fine line.

HP8
I: I suppose, with some of these patients it's going to be... There are people who will have their medicines delivered and they won't actually go into the...
R: Go into pharmacy, yeah.
I: And that could be... Is there therefore something, is that the role the NIPPS could pick up in terms of...?
R: Yeah. I mean, we do, do home visits for patients who are house bound and unable to come out to the GP. And certainly, providing advice on long-term chronic conditions, it's not just about medicines. So we ask for, using the COPD example, we'll refer patients to pulmonary rehab and smoking cessation and all those, sort of, lifestyle things that will help and try and reduce that medicines burden as much as possible.

HP8
R: No, I think definitely safety. It's good to have that safety net and it feels good when you go, has this patient been seen and, yes, they are already getting the care that they need. I think it's really important to have a system that can catch people who might have otherwise slipped through the cracks. I think also integrated care is very important in any patient centred system. Patients see an awful lot of people and those people have to communicate with one another otherwise we know things can go wrong. So, yeah, the communication is really good.
I: You said things can go wrong, what things go wrong?
R: I've, sort of, alluded to it before but if patients are on the wrong medicines or the wrong combination of medicines, if primary care aren't aware, for example, that they need monitoring regularly, if they are on certain drugs, there can be very serious patient harms. And I remember just after I started, there was a case where someone was put on, I think it was atypical antipsychotic, I'm not sure, but they had very severe kidney damage from it. They had not had any monitoring. Primary care thought secondary care were doing it. Secondary care thought primary care were doing it. And their kidneys were shutting down and they were extremely poorly, and I think they passed away. And that happened as I was starting and, you know, the lead in the area was talking to (name) about it and they were... Yeah, it was really upsetting for everyone basically. So, yeah, very serious potential harms if there's not that communication.

HP9
I: Yes, we'll come onto that actually because having that proximity of them is quite interesting, isn't it? When the e-referral service started, how were you told about it? How was it introduced? What did you know about it to start with?
R: I think there was emails from (name) and from maybe (name) in the past that they've just told us about it. I think it might have been discussed in meetings, just briefly what it's about. That it's basically to try and improve the three-way communication because at a certain point I think the chemist will be able to refer to the GP other problems, not just…maybe that's my idea of it, that there'll be three-way communication or I can refer to the chemist through that service. I don't know if that's…this is like, future. I know for now I think it's to communicate with the chemist that the patient's been discharged and give them the discharge summary. And then they will obviously modify it on the system. I'm not really seeing it in my own eyes to say…I've seen the table, the spreadsheet and I know they can…if it's pending or if it's been received, if it's been dealt with and things like that. So I think that's for the time being but I think they've got more plans for it in the future as far as I have heard. But this is just like the trial, isn't it? There to see how it's going.

HP9
I: Yeah. I think…that's really interesting actually. You're saying there about that three-way communication, 'cause I think that was… Do you think that that would be…if you were seeing electronically, immediately, like they're referring straight away, these patients who are on these blister packs, if you were seeing that straight away, what would the benefits be of that?
R: I guess it's having somebody…as in to get the discharges straight away?
I: Yeah, to get it in a w…the [inaudible 10:29], they get it in an hour…
R: The way they get it.
I: …or two or whatever, pretty instant.
R: I guess it depends who will…'cause I'm…most people on my team are not here full-time so I guess it's who gets this. I think that would be my only concern, would it be the admin staff that would get it and flag it up to us? But I think that would be really useful because then sometimes when you…like I say, what you were saying before? It takes a few days to come across and sometimes you do end up with patients on a blister pack maybe that…if the chemist is not as pro-active about it, that have got to the last day and the chemist has got to sort out the Venalink a day before, which is not…as you can probably anticipate, they don't particularly like that, but yeah, I think that would be useful because then you'd get the discharge straight away and then you'd be able to action it straight away and change the medications and send it over.

HP9
I: But is it going to improve? Where is…in some respects, I suppose, what is the potential for the system, or…
R: …to improve…?
I: …to improve care.
R: Yeah, so because obviously if it's sending the discharge summary to them and I guess with the blister packs, they will then have a look, the chemists that have been doing that where they have a look at their sheets for their compliance aid blister pack and they'll just compare it and that's when they flag up to see that, oh, actually, they've got Furosemide for example on this. Oh, but this is not already in the blister pack, so that must be a new thing. That's thinking of the fact that the discharge doesn't document anything accurate in terms of what changes have been done. So they will check the list of medication against their list and see if there's anything, any changes. At least that's the hope with that, and I have had obviously instances where they have flagged things up and I was like, oh, yeah, you're right, I'm just trying to sort it now and…which has been quite good.

HP9
I: Hopefully it's not going to be different, yeah.
R: Yeah. You hope it's not, but yeah, that in terms of…I guess in terms of errors as well. You've got somebody else checking it as well at that point. 'Cause I don't always get the discharges, like I said. So if there's a chemist there as well who's getting and checking the discharge, then that's another step as well in making sure the patient gets it at the right time 'cause they'll all go, oh, well, we usually send it on…they've been

discharged for nearly a week now, can you sort this out? So they'll flag it up even before, sometimes before we've even seen it, so they'll go, patient's going to run out probably, can you sort it out? Yeah.

HP9

I: Which is I'm sure, there's a lot of people that would really like that. So just thinking about then, some things more around the service, at the moment, as we've mentioned a lot through the interview, it's currently just used for what I would call multiple dosage systems other people would call compliance aids or blister packs, whatever. Do you think that's a good thing to be using it for or do you think it could be broadened to other people, other patients?

R: You could roll it like I said, I think you could roll it potentially for other areas. So if the hospital wants the community pharmacy maybe to follow up on something or if the community pharmacy want us to follow up on something or flag an issue to us, they could maybe do it through that system. Again, we could send something to the chemist to tell them when the patient picks up this prescription, make sure you tell them this or do this rather than well, we could still do that by telephone but again, but you could use that sort of thing through it. So I don't know about non-blister pack patients in terms of discharges 'cause I guess they're not as…I mean, they are familiar with them but it's very different 'cause they might not have a set pharmacy, yeah.

I: And if it was used for all patients it would be…

R: Yeah, it would be a bit…I think it would be a bit difficult unless they have a 100 per cent set pharmacy and they pick up everything from there 'cause it's not always the case with people who are not on a blister pack, they might get their medication from…

I: Yeah, 'cause you could be just discharging anybody, I mean, literally every single…

R: Yeah, and they wouldn't really need to amend the medication as such 'cause it's not a blister pack. They'll just get a prescription.

HP9

R: Yeah. Maybe people are on a big list of medic…on lots of medications it might be useful I suppose and you could tell them to enforce…I ring patients but again, because like I say, not all discharges come to all pharmacists, it might be that you could…the hospital pharmacist can say, could you reinforce some advice on this medication that they've been discharged with? 'Cause I do come across patients who maybe have been discharged over the weekend and they might not have a lot of information about the new medications that they've been started on. So when I ring them, they find that quite useful, so they could potentially do that with the community pharmacy, say, can you counsel them.

HP9

I: Yeah, and that was one of the other things that people were talking about last year with the service, was that this would…the potential for this to lead to patients better-informed about the medicines. Do you think that's likely?

R: Yeah. I think so. I think that's the potential with it, yeah, 'cause again, when you say, rolling it out to all patients, because there's people not on a Venalink but are on ten medications and something would have changed in hospital but then if nobody tells them about it then they don't know. They might have a box of Ramipril at home and it's been stopped at the hospital and if someone briefly mentioned that to them or not mentioned it at all then they'll go home and start taking that again. And if the chemist is not informed of that then they might have a box on the shelf waiting for them that repeat prescription or they'll just give them that and then this will just carry on. It's like a – what's it called – the Swiss cheese and when it's like an error, just yeah….

I: Just going through the hole, yeah.

R: …gets passed, yeah…

I: Precisely.

R: …basically. Yeah, and this is a potential…if something stopped they could tell them, this has stopped, they can maybe take it out of the bag and not give them that obviously medication…

HP9

R: Knowing more about their medicines. I mean, I don't know if the chemist…I think they do call the patient and do speak to them as well when they get discharged, just like to make sure that they've got enough supply. I don't know about informing them about their medications but they could potentially have a role in this because, like I say, if not all the discharge…when the discharges come to me I call the patient, go through their medications but if they don't come to me and go to a GP they obviously won't have that conversation.

I: So if you get a dis…when you get a discharge note you generally call the patient?

R: I generally call patients unless there's a reason that I can't get in touch with them or they…I'll just confuse matters if I call them if they've got dementia or something like that and then I'd speak to their carers but that might not always be possible as well. They might have carers coming four times…

I: So that call you're making is to check that they've got what they need?

R: That they've got what they need, but also I go through the list and make sure that they're aware what's changed and what's new, what's stopped and usually give them some advice about any new tablets, if they get started on a lot of medications, I give them advice about it as well, what to look out for. And if they've got any monitoring I make sure that they know about it as well and that they need to book in.

5.4 Code - Value, trust and confidence in health professionals

HP10.

I: That's something, isn't it? Because one of the things I think that was said to me way back, sort of last year sometime, around community pharmacy, and I know that there were people really pushing this agenda forward and saying, well, actually, this is going to give value to community pharmacy. Do you think it has that potential to do that?

R: Most definitely. Honestly, most definitely. We, as a pharmacist, believe me, we do, apart from making accuracy checks, we clinically check things. And if you're clinical checking, it's not only about interactions and side-effects of meds, it's about the broader picture, so you're looking at a number of things. As a clinical check, you're making sure that an elderly lady, can she open it, can she apply that drop, has she had any falls, what's her history? When I do a Medicine Use Review, I will ask them, let them speak for a minute. I say, how are you doing, how are things, how are you managing your meds, how is your life to do with your meds? I listen to them, so that gives you an understanding and picture and I always make myself notes, so, oh, this is this patient. If you try... I always call it I'm trying to do a proper job today. So if you do a proper job, that's how it should be, so as much information as you get, you're able to apply the information later in order to improve the overall safety of patients, and that's it.

HP10.

I: I know also that when they refer, when they do the...and I think they do the referral at the point of admission and then the discharge summary automatically comes to you, but I think there is a potential for them to say, oh, by the way, would you like...can you look…counsel this patient, do an NMS, MUR, or something like that. Has any of that happened? Have you had sent …through the service?
R: Yes, it did, but it's not always that easy. So, normally, I will probably speak to the patient or my staff will speak, saying, how are you, are you out now? Okay, cool, what's happening? Have you got a week's worth? And we always kind of say, okay, cool, listen, we're just working on it so don't worry about it, or we speak to a family member. We will be trying our best, the surgery is working on your script now. When we get the script, we'll get it ready and we'll send it out. Oh, okay, thanks. So this is kind of happening, so the patients are reassured that someone is actually doing something about it.
I: Yeah, precisely.
R: So we normally do that. And then with one lady... You see, a lot of our blister pack patients are patients who wouldn't necessarily come down to see me. We don't have all this... It's really not feasible for us to go out to their houses and do and MUR there, a Medicine Use Review or any other service, but mainly that communication happens over the phone. I had one through that, one lady who was started with a new inhaler, because I do a service called Inhaler Technique Service and she came post-discharge with a new inhaler in her hands, she didn't have a clue what to do with it, which was just surprising. I think she was told, shown and told in the hospital, but I think she... I don't thinks he's diagnosed with dementia but she clearly had got some problems with cognition, she was saying, oh, I don't know what I'm doing, I don't know what I'm doing. So I said, yeah, come on in, have a look, this, that and the other, shown her again. I always give...I think it's called Safety Net. I told her, listen, any time you want, come back. So I kind of did another service, which we get paid a tenner for, showing them how to use their inhaler. And I think what I did as well, I've ordered her like a chamber to put...to get a new inhaler for her, she's not doing it right, she needs something else to make sure that works. So we'll always make sure or explore other avenues of communication: everything alright? You know, let me know if you experience any problems with it, and that's what happens really.

HP12
R: Yes. I think we feel more of an included part of it rather than just the person who provides the meds, who does the blister pack. I feel like we do feel more, and I think we probably feel more valued by the patients when they do that thing where they ring up and say I'm out of hospital and you can say, yes I knew. You feel like, to the patient they feel that you're more involved in their care and that you are aware of what's going on. So, yeah, in terms of that kind of way, it has definitely improved the value.

HP13
I: And do you feel the patients value that?
R: Oh yeah they do, definitely, 'cause that's another thing, when I call at their house, I'll take the old packs away 'cause that's another issue if the medicines have changed, you don't want them picking up two weeks' ago, you don't know how long they've been in hospital, they might've been in hospital two months, they might have loads of packs there so I'll clear them out when I take the new pack. And I will write on the new pack so I know to tell them that this is the new, up-to-date pack and I get rid of all the others and take them away.
I: Right, yeah, and so the patient, yeah.
R: I didn't used to do that, I don't think I did used to do that, I just used to deliver it and say, that's your new medicines, you see. It's just a safer service, really, it's made it safer.

HP13
R: Just makes you feel that you've helped people more and it's making you more professional and more involved in the, I don't know, the process of them being in hospital, I suppose.
I: Yes, precisely, yeah. One of the things that was talked about right at the beginning of…when they were planning it last year and I was going to some meetings, one of the things that was said was this may well enhance the value of community pharmacy.
R: I think it will with time, yeah, I think you've got to give it a bit more time, that's the only thing. But I think eventually the GPs will realise that we're actually saving them from making errors, you see, this is what I think but that is going to take a while before they realise.
I: Because they've got to go through…there's more volume.
R: Yeah, you need more people doing it and this, kind of, thing, you know, but eventually they probably are going to realise, gosh this is useful.

HP15
I: Yeah. Yeah. One of the things, you know, we say here is, what are the potential benefits for your work? Which I think, we've, sort of like, covered in some respects, because we are talking about, you know, time particularly. You also talked about that in, sort of, now having more knowledge about the patients. In what way is that from, sort of, you know, community pharmacy angle, why is it better that community pharmacists learn more about patients?
R: Because we see the patients face-to-face and it gives us a better understanding of their medicines. So, we can speak to the them better about it. Yeah. Because patients don't necessarily understand everything they are told in the hospital, because they are not in that state of mind anyway, when they are not well. So, they wouldn't understand everything they are told in the hospital.

HP15
R: Yeah. They know that they've got the right medication, they don't have to worry about it. Yeah. Before I think…for the people who had the constant problem of not having the right medication on discharge. So, what happens, they might take the medicine home before, and then they realise, oh there's one thing missing on it. That's when we realise that there's something else on the discharge that we didn't have. So, the people who have had this problem, they always check their medicine, no matter when we send it, even if normally, they keep checking, because they know that they have had problems before. But now that is much reduced. So, people don't have that problem anymore.

HP15
R: They just need to know that we've had the prescription with us. Then they know that they will still get the medicine on time. So, if you tell them we have the prescription, they are happy with that, that's fine. And we tell them, we will deliver it tomorrow, even though they are running out, tomorrow evening is fine for them. As long as they know we are getting it to them tomorrow.
I: Yeah. Precisely. That's [inaudible 32:50].
R: But if we say we don't have the prescriptions until now, they won't be happy. Because they know that it's with the doctors. And if we don't have it in our hands, we can't do anything about the medicine. That's why, yeah.

HP16
R: So I think for us it would raise the profile in terms if it's done properly, then patients see the benefit that actually, do you know what, I didn't know you knew I was in hospital, for example, or actually that we're taking that step to have a different conversation. So the conversation isn't, are you out of hospital, do you need medicines, have there been any changes? Again, it's the same conversation that we're having with everybody, the conversation is around, we can see there's been changes do you need any advice on this, did the pharmacy speak to you about it, do you want to come in, we can try and do discharge MUR for you if there's been changes? All those things, this has been started, do you know what it's for, did they explain to you? And they may well have done but actually the patient's state of mind hasn't taken that information in. And that's very, very typical for us so we're able to make a much better impact.

HP2
R: I think you've got peace of mind knowing once that discharge summary has been written and is sent, you know, the chemist is going to…it's going to receive documentation. Because if we…we had failed in the pharmacy previously by faxing everything, then I think that would make me feel that was my fault.
I: Right. Right. Yeah.
R: Do you know what I mean? So, with e-referral you know it's going to get there.
I: So, it's more…a confidence.
R: So, it's more of a confidence, yeah, beforehand if there was…if somebody even forgot to put a cover note on the top of the prescription to say, please fax, that may at some stage not have gone to the chemist.
I: Right.
R: It may never have got there.
I: So, the previous system was one which involved paper and…
R: Paperwork and human error. Yeah.
I: And now it's just…
R: And now it's electronic it goes…
I: Goes to the computer terminal.
R: Yeah. Click a button and it's gone.

HP5
R: Peace of mind for those of us who are doing drugs history, definitely. Peace of mind for patients knowing that their chemist is now going to know of changes that have been made. It also will…I was going to say improve relationships, but the communication between GP surgery and pharmacy is going to be strengthened because they both receive the same information. So, a community pharmacy can phone a GP surgery, confident that the GP surgery has received the same information. Because there's no way if you do an e-referral that one or the other is going to be, both going to receive that, so, it strengthens that. Basically, what it does it strengthens the relationship between all the carers whether they do primary or secondary or whatever, between the community and the hospital. And for me personally I do feel that patients are getting better care as a result or continuity of care. I'm not, because of working between sites, I'm not going to go to another site and scratch my head and think did I send this, did I send that, this and that, or you know. So, you know, it's a job done here and I can concentrate on this other facility here without worrying about back pedalling as to what I've done here. We call it…if one is not concentrating on the job they are doing, somebody else is going to be affected by this.

HP5
R: Less, you have less, right you get less admissions, hospital admissions due to drugs for a start. Patient educated as well, patient education, they'll know exactly what they are supposed to be taking now because they get the same information from all the disciplines involved. Patients can get confused if they get one information from here and another set of information from here, so it's less confusion with regards to their care. And they are less, obviously they are less distraught and traumatised by people not talking to each other.
I: No, knowing what that…yes.
R: About what they should be getting.

5.5 Code What changes should be made - How can the service be improved.

HP10.
I: Yes, precisely. Yes, actually, when it started and they said, oh, we're just going to put patients who are on blister packs, and I thought, oh, that's not many. And then I thought, well, actually, that's the most complicated group of patients, isn't it? Because as you said, they're all multiple morbidity, many drugs and so on and so forth. Having said that, would you welcome any sort of extension of the service, to doing more than just patients on blister packs? Do you think it could be for lots of people?
R: It's interesting, this, you know. I'm struggling to give you a good answer to that because I feel, or I'm under the impression, that the community pharmacy at the moment is underfunded, honestly. From my perspective as a community pharmacy manager, I think... I've managed to retain my staff so far, but there are a huge amount of problems getting someone, employing people for this money that we pay. So you don't necessarily have that many resources and it's hard to motivate people. And we're doing numerous things at the moment and I'm thinking, what will it look like? I'm not sure what we're talking about. I'm guessing that... Because someone said, maybe yourselves, someone said it to me, but there is a plan of rolling this out as all the patients who belong to you say, oh...
I: Yeah, everyone with a nominated pharmacy.
R: ...I'll go to this community pharmacy, send it out. I think it would be great overall, it will improve things probably a lot, but are we able to take it on board, are we able to manage it? It's got to be well... That's my view. Well evaluated first before any next steps are made, in my opinion, because you've got to pick probably... I don't know who I'm kind of addressing that to, you need to pick up a few individual pharmacies and test it well, is it feasible or not? Because you will get a backlash from pharmacies, saying, give over, give us some money for it. I mean, I'm not sure. We don't get any funds for it, we don't get any money paid for it. You see, that's another thing. The money, and again, I'm not a business man, believe me, I don't run this business, this is not my business, I just get paid a salary. But I get loads of sort of, oh no, forget it, you can't get another member of staff on, no chance. We've got this, this. This pharmacy is not profitable, this is not profitable, we're going to shut this, we're going to sell this. So we get those constraints. If we had to do some more work with no reimbursement for it, I think my bosses would be: hold on a minute, give me a break, you know, and there will be, like, people just saying, can't do it. Pharmacists have got so much work to do, the pharmacy staff in

my... I'm talking about my individual pharmacy, it's got so much stuff to do, so it's got to be very carefully evaluated and there's got to be feedback collected from a different kind of parties before any decision is made. That's how I see it. But again, I'm sure it will improve. I've got no data to prove it, but again, I can see it, it will improve overall safety, but is it feasible? That's the big question mark.

HP13
I: Yeah, I mean that's one of the questions we've got here is actually looking at that, whether it should be more broad.
R: It really should. I even…I told them at one of the meetings I had nearly a very serious error because of that, kind of, thing on a lady who wasn't an MDS because she had been in hospital, we didn't know, then she got out and we had packets there for her. They were only about a week or ten days' old, things that we were ready to deliver to her but because we didn't have a discharge, we didn't know what she was on and some blood thinning tablets got delivered to her by my staff who didn't realise; some of them didn't know she'd been in hospital, at that point I actually had done. But they'd gone before I realised so they were delivered to the lady's house and she wasn't on them anymore and that's quite a serious…whereas if I'd have had a discharge for that lady it would've been much, you know, it would've all been organised in a much safer fashion. Nothing happened 'cause the lady was on the ball and she knew she wasn't on them anymore but I didn't.
I: Yeah, and if she hadn't have been.
R: Yeah, exactly, she could've been taking things that were quite dangerous, but what can you do, if they can't roll it out…but it would be useful.

HP13
R: I think there was a little bit on the form that I thought could've been more clear because I didn't know 'til the second meeting about. I mean, it's there but I didnt' see it and it's quite an important little bit. And it was only at the second meeting that one of the ladies from Hope Hospital actually said, oh look, this is when they went in, this is where they're going, which residence they're going to and this is very important. I just think that could be highlighted more, maybe, in case other people haven't noticed it 'cause I'm sure they haven't.
I: Is knowing where they're going really important?
R: Well, it is really because if they're going to residential care, I'm not doing their prescription anymore and the GP needs to know that too because often their GP changes so they won't be doing it either and something needs to be sorted out for them and that, kind of, thing. Or you might want to be saying to the GP, shall we do a couple of weeks 'til they get sorted or whatever, I don't know.

HP8
I: So (name) or whoever comes to you and says, (name), we've decided that e-referrals service needs changing and we think you're the person to change it, so what would you do? You're in charge now, what are you going to do?
R: So I know that one of my pharmacist said to me that it was very difficult to identify the patients from the information given. I don't know what information they need but I would find out and see if we could add that information. Maybe it's their NHS number, which I don't think is included in the spreadsheet, so it's hard for them to identify patients. So that would be the first thing I'd do to improve it. See if I could fix the lag that (name) was talking about as well because then maybe we could action things a little bit quicker and it wouldn't automatically go...Or the community pharmacy wouldn't send the communication to the GP in the first instance, so the GP wouldn't then book appointments in with patients who could maybe see the pharmacist for a better outcome.

HP8
I: So you getting the information earlier basically.
R: Yeah. And then maybe in an ideal world, we could get a spreadsheet with less of the information we don't need, just the information we do need.
I: So streamlining things really?

HP9
I: I'm just seeing if we've covered…so just…that question about discharge information being…so it's timely, relevant and useful. We've definitely covered that one. That's perhaps the last bit. I've asked this to a number of people. So tomorrow morning, someone very important, (name) comes and says, right, you're in charge, and we're doing this e-referral service, and we want you to take over and you can do what you like. Money's no option can do exactly what you like. What changes would you make to the way it's done at the moment? What would you do?
R: First, I'd get rid of that spreadsheet and make it a bit more like…somehow make it possible translate a bit better to us. 'Cause it's just not useful at all. At the moment it's just useful to highlight the hospital number and then we can ….yeah, and that's the other thing as well, maybe have an NHS number, that would really help because I've have to go back to the system of the hospital to try and retrieve that patient and find out who it is.
I: Yeah. 'Cause it's got the hospital number, not an NHS number…
R: Yeah, not the NHS number.
I: …and you do NHS numbers, yeah.
R: So I'd add that on. I would probably make it a bit more simpler to…and more useful information translated from that system 'cause I think at the moment it just doesn't really…it's just a lot of codes and numbers that are…
I: And would you like it to be pinging you as well as pinging to…?
R: I would say to the practice, because again, I'm not here full-time so I wouldn't…
I: Yes, to the practice.
R: …and the same with other pharmacists. They probably would say, to the practice because if you're not here then somebody else needs to take on that…all those discharges that are coming across, I would say to come into the practice but that would be better because it'll be just flagging up straight away, won't it, so there'll be able to sort out, medicate, hopefully send the discharges across a lot quicker to the doctors. So that would be…or then the pharmacist so that would be a bit better in that aspect. And I guess, like we said, make it, expand it. You can always expand this service and include other things like us able to refer to the chemist, they're able to refer to us in a way through that system. I know they can do that now but other things as well, we could do that. And then the hospital as well, being able to…the counselling bit I think would be quite useful to say that we'd counselled them on this and us receiving that information, the chemists receiving that information. I think that would be the way forward.
